# “Click” formation of the imine bond in water

**DOI:** 10.1039/d6sc06874j

**Published:** 2026-10-02

**Authors:** Deepak, Tommaso Marchetti, Federico Rastrelli, Luca Gabrielli, Leonard J. Prins

**Affiliations:** a Department of Chemical Sciences, University of Padua Via Marzolo 1 35131 Padua Italy leonard.prins@unipd.it

## Abstract

The imine bond is a cornerstone of supramolecular chemistry because it combines stability and reversibility with ease of formation and the availability of a large number of precursors. Yet, the low hydrolytic stability of imines poses an important limitation as it prevents their application in water. Here, we show imine bond formation in water with the characteristics of a ‘click’ reaction: fast, quantitative and selective using reactants in a stoichiometric ratio at sub millimolar concentrations. Our strategy relies on the presence of a Zn(ii)-metal complex in one of the precursors that binds and preorganizes the other reactant, activates the carbonyl for nucleophilic attack and stabilizes the imine through coordination. The reaction proceeds with fast kinetics (*k* ∼5 M^−1^ s^−1^) and yields a highly stable imine (*K* > 10^5^ M^−1^) at neutral pH. Imine formation remains effective in a complex chemical matrix such as *E. coli* cell lysate. It is further shown that structural tuning of the building blocks enables efficient imine bond formation using either aromatic or aliphatic amines without requiring any other functional groups. The imines obtained following this strategy enable dynamic combinatorial chemistry at submillimolar concentrations in water.

## Abstract 2

Imine bond formation occurs in water with the characteristics of a ‘click’ reaction: fast, quantitative and selective using reactants in a stoichiometric ratio at sub millimolar concentrations.

## Graphical abstract

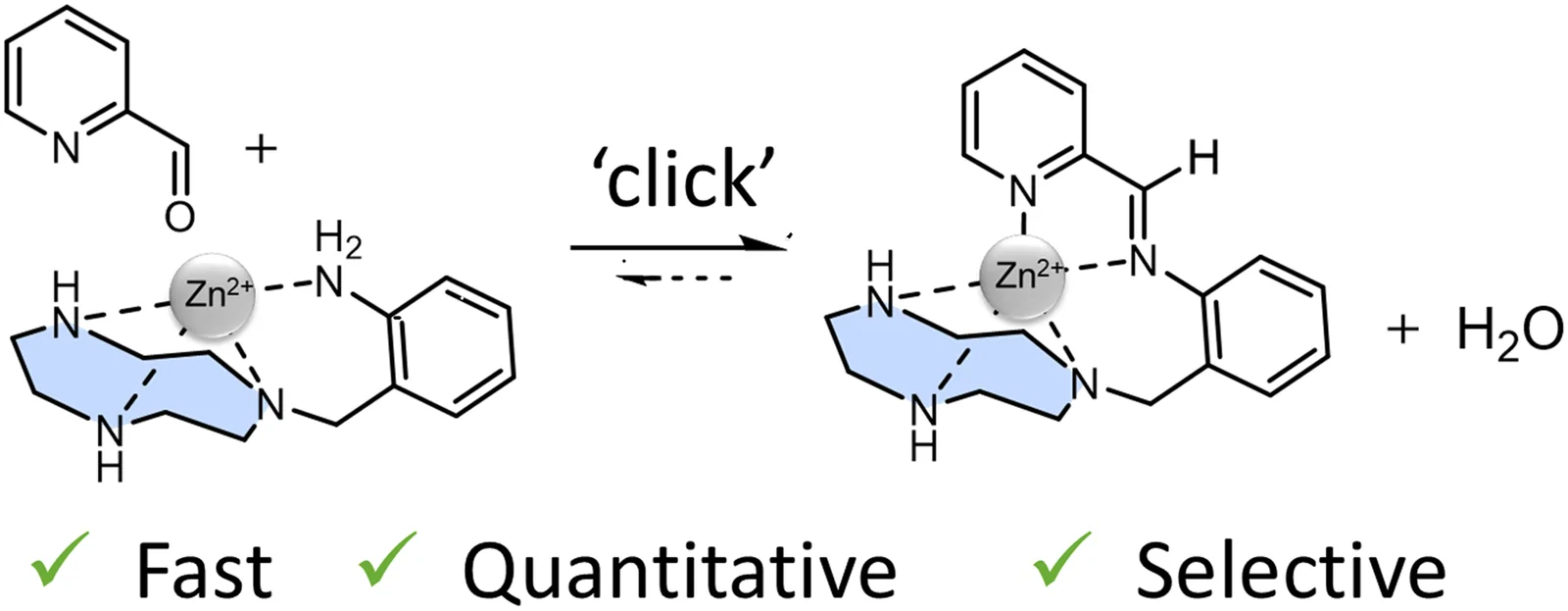

## Introduction

Imine bond formation between an aldehyde and amine is one of the most fundamental reactions in dynamic covalent chemistry owing to the ease of formation under mild conditions and the availability of a wide range of precursors.^1,2^ The reversible nature of imine bond formation and the possibility of amine exchange through a transimination reaction have enabled broad applications in dynamic chemical systems, such as the self-assembly of supramolecular structures, dynamic combinatorial chemistry, molecular machines, and responsive materials.^3–7^ However, an unresolved issue is the practical use of the imine bond in aqueous environments, which is severely limited by the low hydrolytic stability.^8–10^ This issue has been addressed by creating hydrophobic domains in water through surfactant self-assembly,^11–18^ the use of cooperativity in multivalent structures or the exploitation of templates.^19–22^ In particular, the simultaneous coordination of aldehyde and amine to metal ions has proven to be an effective strategy for the self-assembly of molecular cages containing multiple imine bonds.^23–25^ Despite the success of these approaches, the abiotic synthesis of a single imine bond in water has remained elusive. An important strategy to resolve the issue is provided by nature. The cofactor pyridoxal phosphate (PLP) forms an imine bond with amino-residues in the active site of enzymes which is stabilized through intramolecular hydrogen-bonding with the *ortho*-hydroxyl group adjacent to the aldehyde group of PLP.^26–28^ The use of neighbouring groups to stabilize imine bonds has also been successfully applied in a synthetic context enabling the formation of a single imine bond in water with yields up to 80%.^29,30^ Despite these advances, the formation of a single imine bond in water remains challenging, as existing systems often require high precursor concentrations, exhibit slow kinetics, or lead to kinetically trapped products. Recently, we reported a metal ion-assisted imine bond formation strategy in which a Zn(ii)-metal ion complexed by the amine precursor plays a pivotal role in stabilizing the imine through coordination to the imine nitrogen together with a complementary phenoxide–Zn(ii) interaction.^31^ This dynamic covalent capture approach enabled the near-quantitative formation of imines in water using stoichiometric amounts of aldehyde and amine.^32,33^ Yet, the reaction proceeded very slowly, requiring a few days to reach near-quantitative conversion for the most reactive aldehyde (2-hydroxy-5-nitrobenzaldehyde), making the reaction less attractive from a practical point of view. Furthermore, imines were obtained as kinetically stable products that could not be subjected to dynamic exchange reactions. These limitations hinder the application of imine bond formation as a ‘click’ reaction to develop dynamic chemical systems in water, which demands high selectivity, fast kinetics, and quantitative conversion.^34–37^

Here, we show that these limitations can be resolved by exploiting a pyridine-based aldehyde rather than salicylaldehyde or derivatives (Fig. 1). The pyridine unit enhances the electrophilicity of the carbonyl group while simultaneously enabling coordination to the Zn(ii)-metal center. The resulting enhancement in reaction kinetics and high thermodynamic stability of the imine enable fast and quantitative imine bond formation upon reaction with a 1,4,7-triazacyclononane (TACN)·Zn(ii) functionalized aniline at submillimolar concentrations in water at room temperature (Fig. 1, strategy I). Furthermore, we show that structural tuning of the building blocks allows efficient formation of imines in water using either aromatic or aliphatic amines that do not require any additional modification (Fig. 1, strategy II). The obtained imines are subject to fast exchange reactions implying their potential to be used in dynamic chemical systems.

**Fig. 1:**
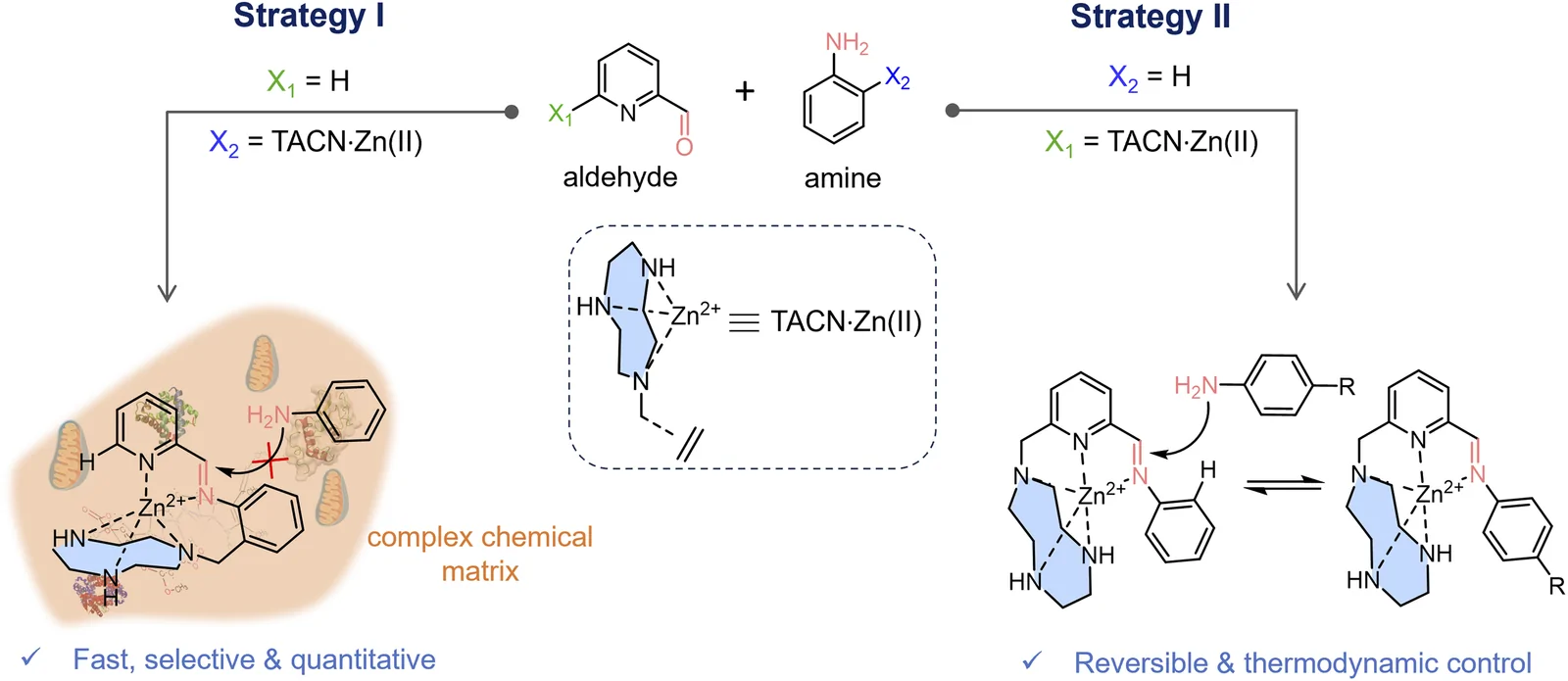
Strategies for imine bond formation in water and chemical structures of the precursors. The first strategy leads to the fast and highly selective ‘click’ formation of an imine with high kinetic and thermodynamic stability. The second strategy permits the use of simple amine precursors and yields imines that undergo rapid transimination reactions.

## Results and discussion

### Imine bond formation in water

Building on the strategy developed earlier, here we introduce pyridine-based aldehydes to provide a complementary interaction to imine bond formation *via* coordination of the pyridine nitrogen to Zn(ii) present in TACN·Zn(ii)-functionalized aniline A. Density functional theory (DFT) calculations suggested that imine bond formation between pyridine-2-carboxaldehyde (1) and A positions the pyridine nitrogen in an optimal spatial orientation for coordination to the Zn(ii)-center in 1-A (Fig. 2c and SI-Section 6). DFT calculations were performed with Gaussian 16 (ref. 38) at a wb97xd/6-31g(d,p) level of theory. Solvent effects (water) were accounted for in the optimization step using the polarizable continuum model as implemented in Gaussian 16.^39^ All geometries were checked to be true minima by a frequency calculation. Experimentally, we indeed observed that an equimolar mixture of 1 and A at 0.5 mM each in aqueous buffer (pD = 7.4, [HEPES] = 10 mM, *T* = 25 °C) resulted in the rapid formation of 1-A, achieving a conversion of around 90% within 1 h and eventually reaching 95% (Fig. 2a and S1). It is noted that a near-quantitative conversion is achieved even though 1 is present as an equilibrium between aldehyde and hydrated aldehyde, with an equilibrium constant (*K*_eq._) of approximately 0.64 (Fig. S1). The resulting imine 1-A remained stable for months at room temperature (Fig. S2). Quantitative imine formation (within detection limits) was achieved within 20 minutes using 2 equivalents of either 1 or A with respect to the other component. (Fig. S3 and S4). The structural characterization of imine 1-A by ^1^H NMR, ^13^C NMR, HR-MS, and ^1^H-^13^C heteronuclear correlation spectroscopy is provided in Fig. S6–S9. The rapid reaction kinetics required monitoring of product formation by UV-vis rather than ^1^H-NMR spectroscopy (Fig. S5). Although just a lower limit for the (apparent) binding constant (*K*_1-A_ > 10^5^ M^−1^) could be determined at this concentration, the rate constant of formation, *k*_f_, could be determined accurately. The obtained value of 5.3 M^−1^ s^−1^ implies a ∼30-fold increase in rate compared to the reaction of A with 2-hydroxy-5-nitrobenzaldehyde (2), which was the most active salicylaldehyde derivative in our previous studies.^31^ The enhanced performance is attributed to multiple cooperative effects originating from the presence of the pyridine ring in 1. The pyridine ring serves as electron withdrawing element that enhances the electrophilicity of the carbonyl group. Chelated coordination of the pyridine nitrogen and carbonyl oxygen to Zn(ii) in A leads to a strong increase in effective molarity of aldehyde and amine and a further increase in carbonyl electrophilicity caused by coordination to Zn(ii). Finally, coordination to Zn(ii) of the pyridine nitrogen in concert with the imine-nitrogen stabilizes the product.

**Fig. 2:**
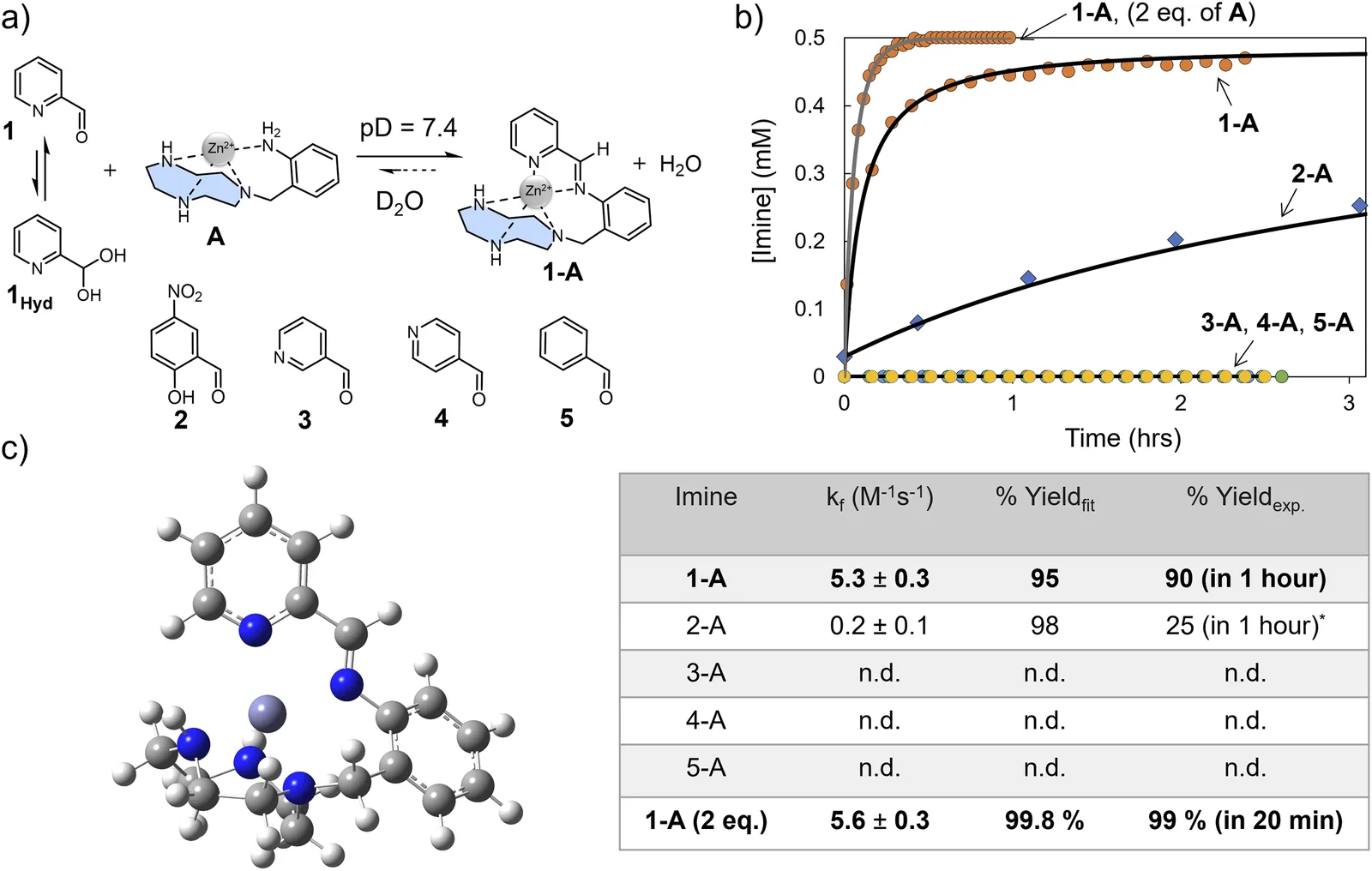
(a) Imine bond formation between pyridine-2-carboxaldehyde 1 (partially hydrated as 1_hyd_ with *K*_Hyd_ ∼0.63) and TACN·Zn(ii)-functionalized aniline A, chemical structures of alternative aldehydes 2–5. (b) Imine-concentration as a function of time upon mixing A and aldehydes 1–5 with rate constants and yields, comparison table for the kinetic parameters of present Zn(ii)-templated imine formation system with pyridine aldehyde regioisomers and reported salicylaldehyde derivative. (c) DFT-optimized geometry of imine 1-A. Hydrogen atoms are shown in white, carbon in grey, nitrogen in blue, and Zn(ii) in light blue. Conditions: [aldehyde] = [A] = 0.5 mM, HEPES = 10 mM, [pD = 7.4, D_2_O, 25 °C]. *Previously reported.^31^

No trace of imine was observed using either pyridine-carboxaldehyde isomers 3, 4 or benzaldehyde 5 under identical conditions (Fig. 2a and S10–S12). The absence of imine formation for these aldehydes highlights that carbonyl activation by the pyridine ring alone is insufficient, and that a precise spatial arrangement of donor atoms for coordination to Zn(ii) is crucial for efficient reactivity and stability. The importance of the TACN-Zn(ii) complex for the rapid and quantitative formation of 1-A was demonstrated by the absence of any imine formation when 1 was mixed with aniline in a 1 : 1 ratio under otherwise identical conditions, even after several days (Fig. S13). The essential role of Zn(ii) in activating the aldehyde, preorganizing the reactants, and stabilizing the imine product was confirmed by the complete absence of imine formation when 1 and A were mixed in the absence of Zn(ii) (Fig. S14). Upon addition of 1 equivalent of Zn(NO_3_)_2_, product formation initiated instantly, reaching ∼90% conversion within 1 h. Replacing Zn(NO_3_)_2_ with ZnCl_2_ had no significant effect on the reaction kinetics, indicating that the Zn(ii) counterion has a negligible influence on the efficiency of imine-bond formation (Fig. S15 and S16). The strong complexation of Zn(ii) by imine 1-A was confirmed by the observation that the addition of an excess of TPEN, a strong chelator for Zn(ii), did not lead to the dissociation of Zn(ii) from imine 1-A over a time course of 26 hours (Fig. S17). On the other hand, Zn(ii) ions were easily sequestered from the precursor A using 1 equivalent of TPEN, indicating that strong Zn(ii) chelation is significantly enhanced only after the formation of 1-A (Fig. S18). The addition of aldehydes or amines to 1-A did not lead to any significant change even after 48 hours suggesting that 1-A has a high kinetic and thermodynamic stability (Fig. S19). The stability of 1-A was put to the test through competition experiments with Girard's reagent T (T). Generally, acyl hydrazones, such as 1–T, have a much higher thermodynamic stability than imines because of delocalization of the π-electrons of the C=N bond. Mixing 1, A, and T in a 1 : 1 : 1 ratio at an 0.5 mM concentration each showed that initial formation of 1-A occurred as if T was not present, reaching ∼90% conversion of 1 into 1-A after 1 hour. Only at a much longer timescale a gradual exchange between A and T occurred, but even after 40 hours the presence of 1–T still amounted to just 20%. Heating the mixture to 70 °C accelerated exchange and after around 1 day an equilibrium mixture with 1-A and 1–T in a 1 : 1 ratio was obtained (Fig. S20 and S21). The same distribution was obtained when T was added to preformed 1-A, confirming that this distribution reflects the relative thermodynamic stabilities (Fig. S22 and S23). These studies confirm the high thermodynamic stability of imine 1-A, but, most strikingly, also the high rate of formation. Imine 1-A has a high kinetic stability resulting in a very low exchange rate.

### Boundaries and biocompatibility

We explored the boundaries of imine bond formation in water by studying the effect of pH, concentration, and compatibility with the presence of other functional groups. pH-Dependent reaction kinetics were measured using UV-vis spectroscopy by monitoring an increase in absorbance at 375 nm originating from imine 1-A (Fig. S24–26). The formation of 1-A was independently confirmed by ^1^H NMR spectroscopy, which also enabled determination of the molar extinction coefficient (Fig. S27–S31). Increasing the pD significantly accelerated the reaction between 1 and A, with the forward rate constant increasing over 3 orders of magnitude from 1.4 M^−1^ s^−1^ at pD 5.9 to 1.1 × 10^3^ M^−1^ s^−1^ at pD 10.4 (Fig. 3a, inset). Indeed, at pD 10.4 the reaction was so rapid that quantitative product formation had occurred within approximately 1 minute after mixing (Fig. S26A). Correspondingly, the conversion after 1 h increased from 90% at pD 7.4 to approximately 96% at all pD-values higher than 7.9. The pH-effect is against the trend typically observed for imine formation in water. Generally, a bell-shaped curve is obtained when plotting imine formation as a function of pH. A slightly acidic pH is required to facilitate dehydration of the hemiaminal intermediate, but at too acidic pH values rates drop due to amine protonation. We attribute the different trend observed for the formation of imine 1-A to a stronger coordination between the pyridine nitrogen and Zn(ii) at higher pH. Furthermore, we expect that Zn(ii) takes over the role of H^+^ in facilitating dehydration of the intermediate hemi-aminal. Coherent with a second-order reaction, a lower rate of imine 1-A formation was observed when the initial concentrations of both building blocks 1 and A were first reduced from 0.5 mM to 0.25 mM and then further to 0.05 mM at pD = 7.4 (Fig. S32). Yet, even at an initial concentration of 50 µM a conversion of ∼36% was still achieved within 1 h (Fig. 3b). Even at this concentration a quantitative conversion of 1 could be achieved within 1 hour when 7 equivalents of A were used, illustrating the efficacy of the reaction (Fig. S33).

**Fig. 3:**
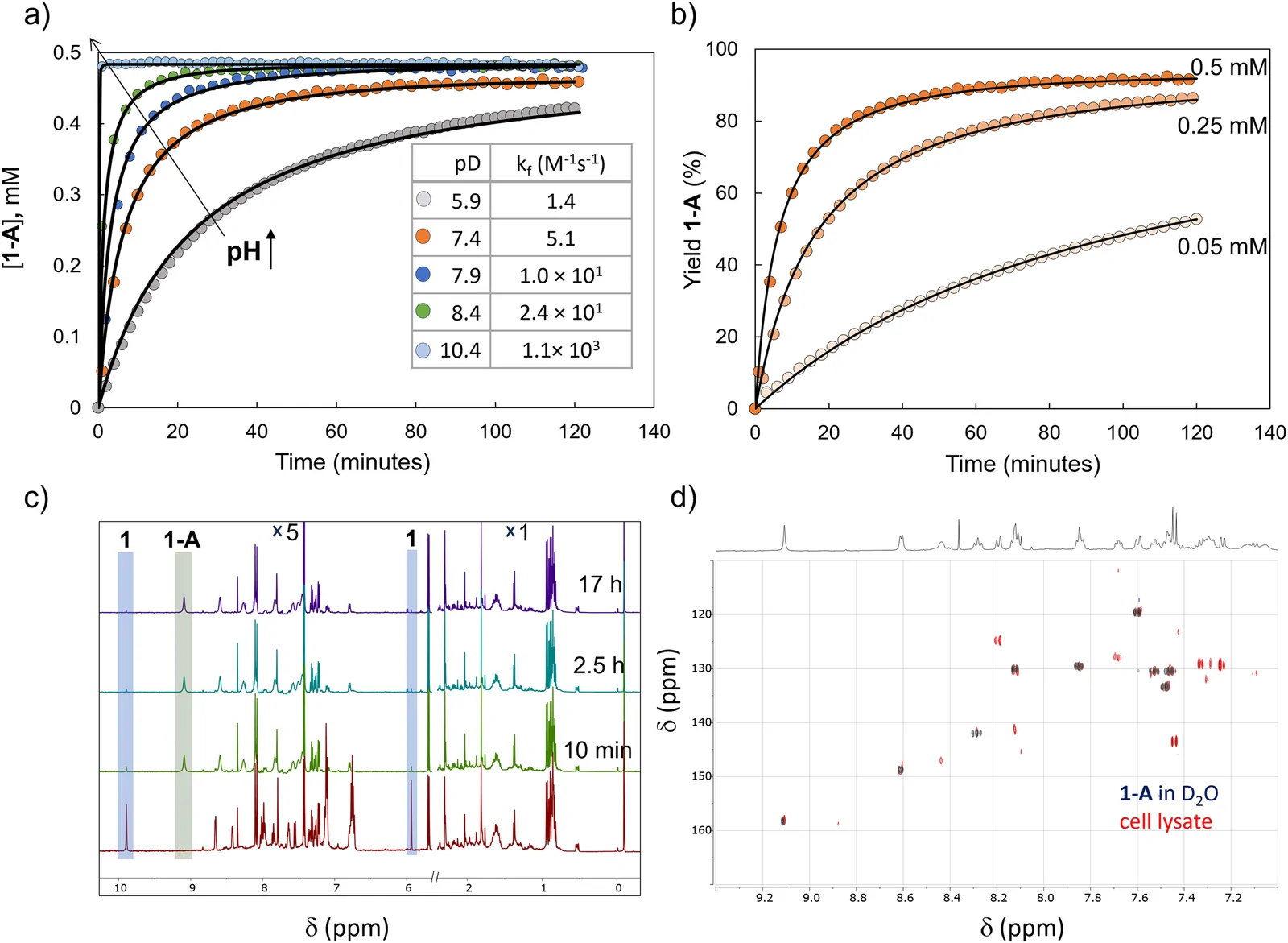
(a) Concentration of 1-A as a function of time upon mixing 1 and A at different pH. The inset table shows the measured rate constant of formation with pH, [1] = [A] = 0.5 mM. (b) Percentage of imine formation upon mixing 1 and A at different concentrations at pD 7.4. (c) Parts of the ^1^H spectra taken at various times after mixing 1 and A in in cell lysate demonstrating the selectivity and compatibility of imine bond formation in a complex biological mixture, [1] = [A] = 1.0 mM at pH = 7.0. (d) Overlapping ^1^H-^13^C HSQC spectra (partial) of the cell lysate containing 1-A (red) and 1-A in D_2_O (blue).

A final important feature of ‘click’-reactions is their excellent specificity which enables bond formation in complex chemical matrices. The tolerance of imine bond formation between 1 and A to the presence of chemical functionalities, including potentially competing amino-groups, was investigated by studying imine bond formation in *E. coli* cell lysate. The cell lysate was obtained through mechanical rupture of an *E. coli* cell culture which was only subjected to centrifugation to remove polymeric material, mostly consisting of nucleic acids, that caused line broadening in the ^1^H NMR spectrum (Fig. S34). The presence of numerous signals in the ^1^H NMR spectrum of the cell lysate confirmed the chemical complexity of the mixture. Upon mixing equimolar amounts of 1 and A (1.0 mM each) the imine signals corresponding to 1-A appeared within minutes, and an 89% conversion was achieved in 10 minutes (Fig. 3c and S35). An ^1^H-^13^C HSQC-spectrum was recorded to unequivocally assign the signals to imine 1-A and formation was also confirmed using HR-MS (Fig. 3d, S36 and S37). This result demonstrates that the functional group patterns present in 1 and A are specific enough to enable fast and selective imine bond formation in a complex biological environment.

### Reversible imine bond formation

The synthesis of 1-A illustrates that imines can be rapidly and quantitatively obtained at low concentrations in a complex chemical matrix in water. The high kinetic stability of the imine bond further increases the potential for use as a ‘click’ bond for bioconjugation. Yet, the high kinetic stability, reflected by the observed very low rate for amine exchange, poses a limitation for application in the field of supramolecular chemistry, which requires dynamicity of the imine bond to enable adaptive behaviour of imine-based chemical systems under thermodynamic control. To resolve this issue, we redesigned the aldehyde component by installing a TACN·Zn(ii) unit directly onto the pyridine-carboxaldehyde scaffold (6). This way, after imine bond formation, the amine building block would be coordinated to the Zn(ii)-metal ion just through the imine-nitrogen and therefore avoiding kinetic locking through chelation. In addition, both aldehyde and imine activation through Zn(ii)-coordination would be maintained to kinetically favour both imine bond formation and, potentially, transimination. A final important advantage of this design is that in principle any amine can be exploited for imine formation which significantly enhances the reaction scope (Fig. 4a).

**Fig. 4:**
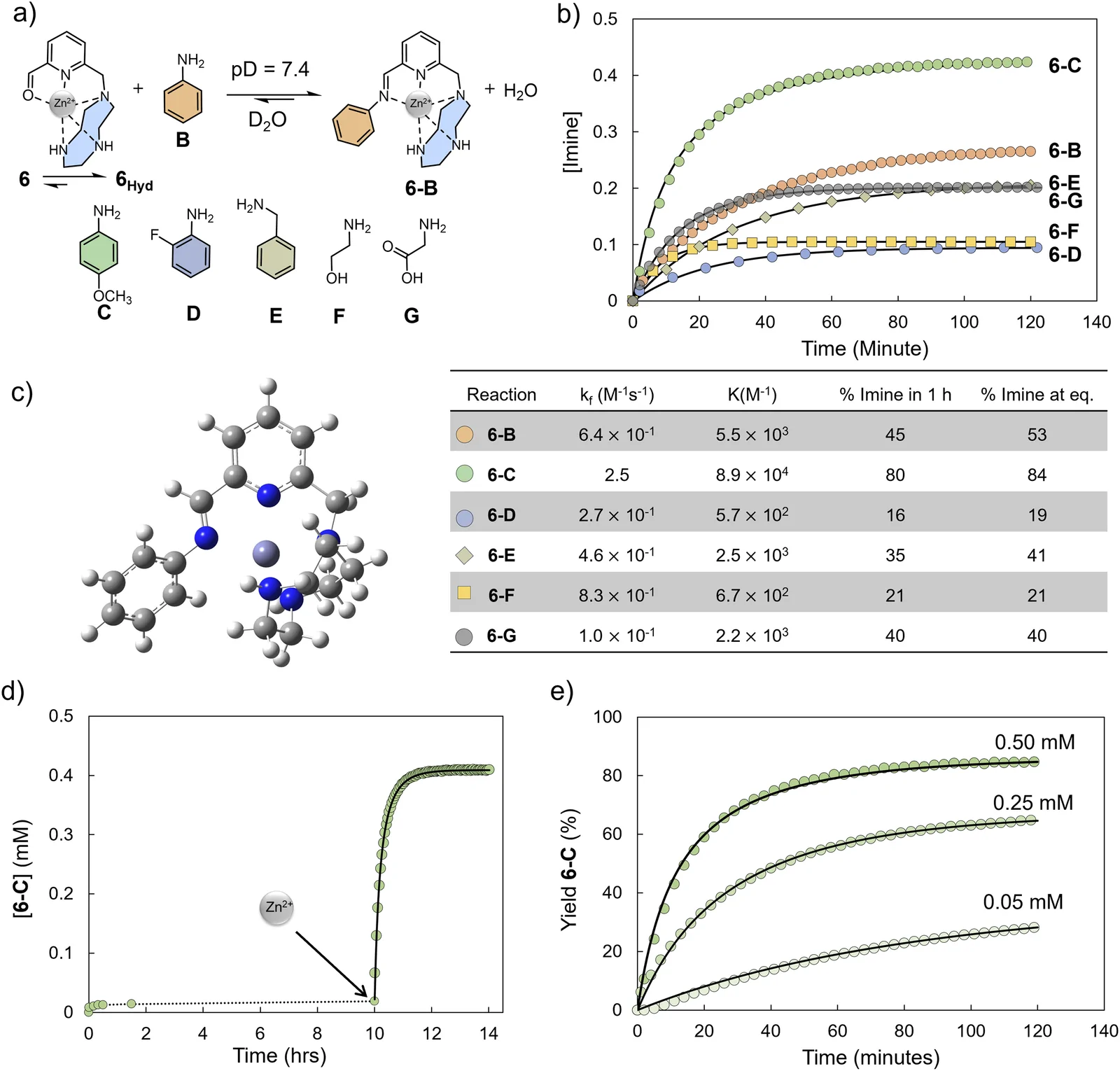
(a) Imine bond formation between aldehyde 6 (fully hydrated as 6_Hyd_) and aniline B and the chemical structures of alternative amines C–G. (b) Imine concentration as a function of time upon mixing 6 with amines B–G with rate constants of formation (*k*_f_), apparent equilibrium constants (*K*) and yields, [6] = [B–G] = 0.5 mM, 10 mM HEPES pD = 7.4. (c) DFT-optimized structure of imine 6-B imine highlighting coordination of the imine nitrogen to Zn(ii). (d) Control experiment demonstrating negligible imine formation in the absence of Zn(ii), followed by instant formation upon Zn(ii) addition, [6] = [C] = [Zn(NO_3_)_2_] = 0.5 mM. (e) Percentage of imine formation upon mixing 6 and C at different concentrations at pD 7.4.

Imine bond formation was initially investigated by mixing 6 with aniline (B) in an equimolar ratio under the same conditions used for the synthesis of 1-A (*i.e.* [6] = [B] = 0.5 mM, pD = 7.4, [HEPES] = 10 mM, *T* = 25 °C). It is noted that under these conditions the aldehyde group in compound 6 is fully hydrated, which, however, does not appear to affect imine bond formation. Indeed, we were pleased to observe the rapid formation of imine 6-B as evidenced by the appearance of the signal originating from the imine-proton at *δ* = 8.90 ppm and concomitant disappearance of the methine-signal from the hydrated aldehyde at *δ* = 6.17 ppm (Fig. S38 and S39). The correspondence between the measured and calculated ^1^H-^13^C HSQC spectra, together with data obtained from standard characterization techniques, confirmed the structure of 6-B (Fig. S59–S62). The measured rate constant for formation, *k*_f_, of 0.64 M^−1^ s^−1^ is lower than the one determined for 1-A, but still sufficient to account for 45% conversion after 1 hour (Fig. 4b). Evidence that 6-B formed under thermodynamic control was obtained by separately synthesizing 6-B and studying dissociation of an 0.5 mM solution under the same conditions (Fig. S40). Both mixtures converged to the same composition containing ∼53% of 6-B at thermodynamic equilibrium, which corresponds to an equilibrium constant, *K*_eq.,__6-B_, of 5.5 × 10^3^ M^−1^. Density functional theory (DFT) calculations performed on 6-B at the same level of theory as before demonstrated that the coordination of imine-nitrogen to Zn(ii) ions helps in stabilization of 6-B (Fig. 4c and SI Section S6). We explored the reaction scope using various amines, initially focusing on the importance of electronic effects on imine formation rate and stability. The use of electron-rich aniline-derivative C resulted in a 4-fold increase in rate of formation (*k*_f_ = 2.6 M^−1^ s^−1^) and a 16-fold increase in thermodynamic stability (*K*_eq.,__6-C_ = 9.0 × 10^4^ M^−1^), resulting in an 80% conversion after 1 hour at room temperature (Fig. 4b, S41, S42 and S55–58). These latter values fall just short of those determined for imine 1-A, which illustrates the validity of the new design. Coherently, the presence of the electron-withdrawing fluorine-substituent in D led to a decrease in both the rate of formation (*k*_f_ = 0.3 M^−1^ s^−1^) and stability (*K*_eq.,__6-D_ = 5.7 × 10^2^ M^−1^) (Fig. 4b, S43 and S44). Imine bond formation with aromatic amines, such as B–D, is thermodynamically favoured because delocalization of the π-electrons of the C=N bond through conjugation stabilizes the imines thermodynamically. Yet, we found that also aliphatic amines E, F, and G form imines upon reaction with 6 although with lower, but still appreciable, conversions of 35, 21, 40% after 1 hour, respectively (Fig. 4b and S45–S50). The essential role of Zn(ii) emerged from the complete absence of imine formation up to 10 hours after mixing 6 and C under the same experimental conditions as before, but in the absence of Zn(ii). Addition of Zn(NO_3_)_2_ (1 equiv.) immediately triggered formation of imine 6-C with the same kinetic and thermodynamic parameters as determined before (Fig. 4d, S51 and S52). We also explored the efficiency at lower concentrations by mixing 6 and C at either 0.25 mM or 0.05 mM each. Inevitably, dilution resulted in increased equilibration times and less product at equilibrium, but even at 0.05 mM a conversion of ∼30% was still observed after 2 hours (Fig. 4e and S53). By increasing the number of equivalents of C relative to 6 the equilibration could be accelerated and the equilibrium composition shifted towards product 6-C. Using 3 equivalents of C, a near-quantitative yield (96%) was obtained within 1 hour at 0.5 mM (Fig. S54).

### Dynamic combinatorial library of imines in water

Dynamic combinatorial libraries (DCL) consist of interconverting species formed through reversible reactions under thermodynamic control.^40–44^ Such systems are widely used to rapidly identify molecular receptors and catalysts and to develop responsive materials.^45–50^ To illustrate that our approach allows for the formation of DCLs based on single imine bond formation at submillimolar concentrations in water, we prepared a small DCL in which amines B–D compete for binding to aldehyde 6. We first employed a stepwise approach in which equimolar amounts of amines B–D (0.5 mM each) were added sequentially to 6 following the order of increasing thermodynamic stability of the corresponding imines (D followed by B, then C) as illustrated in Fig. 5a. Each subsequent amine was added to the mixture after equilibrium had been reached and changes in the composition of the mixture were determined by integrating the signals in the ^1^H NMR spectrum corresponding to hydrated 6 (6.17 ppm), and imines 6-B (8.90 ppm), 6-C (8.84 ppm), and 6-D (9.01 ppm). The ^1^H NMR spectra clearly show the dynamic reshuffling of imines after each addition (Fig. 5b, c, S63 and S64). A mixture with the same identical composition was obtained when amines B–D were added simultaneously to aldehyde 6, which unequivocally demonstrates that the imine-based DCL is under thermodynamical control and that the composition is determined by the relative thermodynamic stabilities (Fig. 5a, b, S65 and S66). The equilibrium is reached within 2 hours, which is an acceptable time frame for practical applications, especially considering that reactions were carried out at submillimolar concentrations at room temperature. The distributions of 6-B, 6-C, and 6-D at thermodynamic equilibrium obtained from both the one-pot and stepwise approaches are in excellent agreement with the distributions calculated based on the relative thermodynamic stabilities of the imines (Fig. 5b and S67). The observation that in the sequential addition experiment the concentrations of imines upon addition of a new amine decrease at a rate faster than permitted based on the hydrolysis rate suggests that the imines are subject to transimination reactions (Fig. S68).

**Fig. 5:**
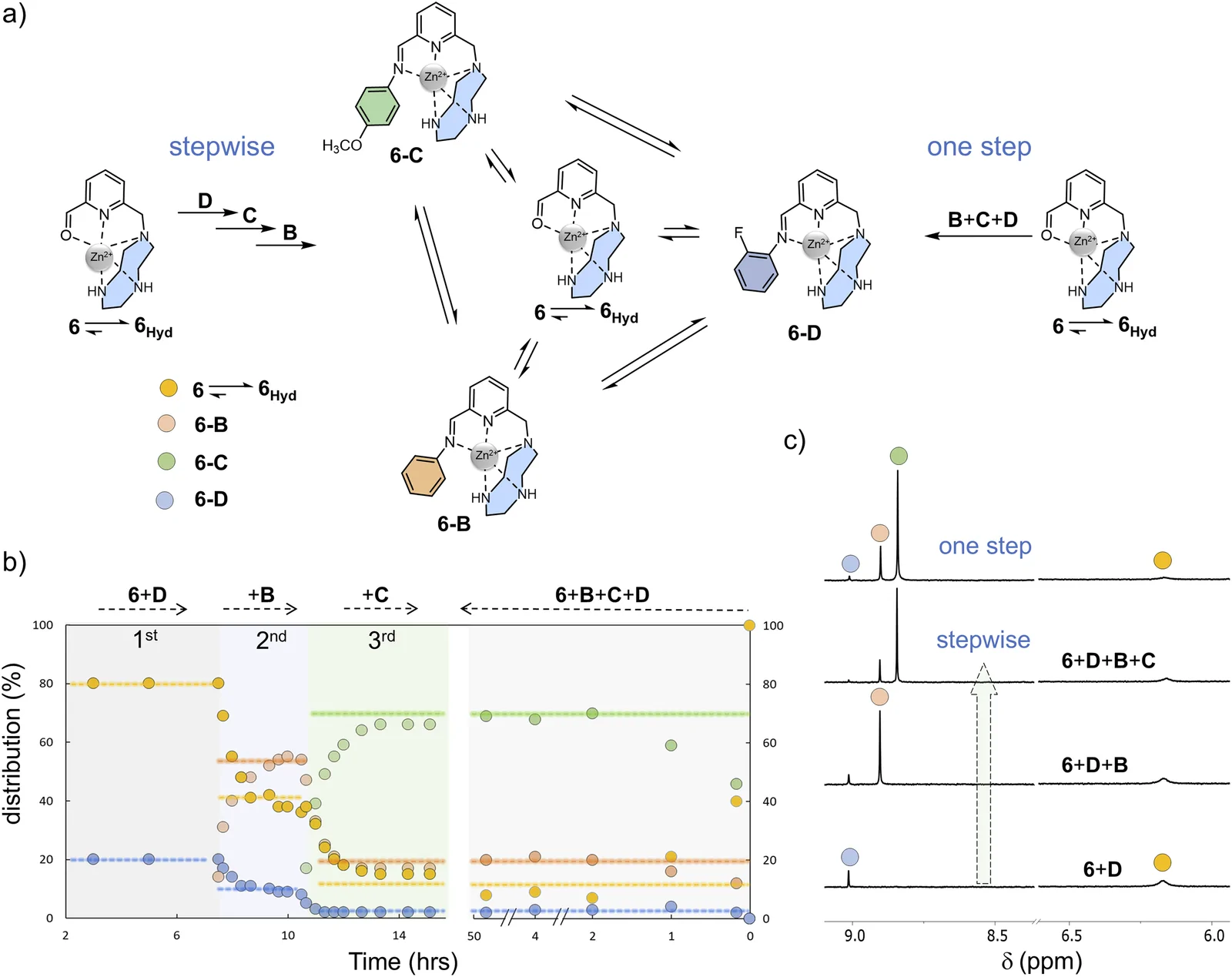
(a) Formation of a dynamic combinatorial library of aldehyde 6 (fully hydrated as 6_hyd_) and imines 6-B, 6-C, and 6-D obtained either by the stepwise addition of amines D, C, and B to 6 (left) or by the one-pot addition of amines B, C, D to 6 (right). (b) Composition of the dynamic combinatorial library upon the stepwise (left) or one-pot (right) addition of amines B, C, D to aldehyde 6. The dotted lines refer to the calculated composition based on the equilibrium constants (Fig. S67). (c) Evolution of product distribution monitored by ^1^H NMR during sequential addition of amines highlighting dynamic reshuffling of imines after each addition. Conditions: [6] = [D] = [B] = [C] = 0.5 mM, 10 mM HEPES, pD = 7.4, 25 °C.

## Conclusions

We have reported a strategy for rapid and quantitative imine bond formation in water. Cooperative interactions between TACN·Zn(ii) functionalized aniline and pyridine-based aldehydes enable carbonyl activation, reactive group preorganization, and imine stabilization, resulting in fast reaction kinetics and high conversions under neutral conditions, even at micromolar concentrations. Imine bond formation occurs with high selectivity and remains effective in a complex chemical matrix, such as *E. coli* cell lysate, this fulfilling key criteria of click chemistry and highlighting its potential for bioorthogonal applications.

The disadvantage of this ‘click’ reaction is the high kinetic stability of the imines which makes it less suitable for applications in supramolecular chemistry that exploit the reversibility of imine-bond formation. This issue is addressed in a second strategy in which we show that structural tuning of the building blocks gives access to the formation of imines in water exploiting aromatic or aliphatic amines. These imines still have a high thermodynamic stability – albeit less than the ones obtained using the ‘click’ reaction –, but the lower kinetic stability enables efficient exchange reactions. It is shown that a dynamic combinatorial library of imines reaches thermodynamic equilibrium within 2 hours at submillimolar concentrations in water. In both systems, the reaction kinetics and thermodynamic stability of imine formation rely critically on Zn(ii)-mediated coordination, which implies that Zn(ii) can be used as control element to trigger imine bond formation. The versatility of the TACN macrocycle as a complexing agent suggests that variation of the metal ions provides an additional attractive tool for tuning the performance of the system. Overall, this study unlocks imine chemistry in water for potential applications in bioconjugation and responsive molecular systems.

## Author contributions

D. and L. J. P. designed the experiments; T. M. carried out preparatory studies; D. carried out all the experiments; F. R. carried out the DFT calculations; L. G. carried out data analysis; L. J. P. supervised the research; D. and L. J. P. wrote the manuscript with input from all other authors.

## Conflicts of interest

The authors declare no conflicts of interest.

## Supplementary Material

SC-OLF-D6SC06874J-s001

## Data Availability

All experimental procedures and characterization data supporting the findings of this study are available within the article and its supplementary information (SI). The authors have cited additional references within the SI.^51^ Supplementary information is available. See DOI: https://doi.org/10.1039/d6sc06874j.
