# Supplementary material for: “Click” formation of the imine bond in water
Source: Chem Sci. 2026 Oct 2. Online ahead of print. doi: 10.1039/d6sc06874j (PMC13647122; doi:10.1039/d6sc06874j)
Supplement: Supplementary file 1 [file SC-OLF-D6SC06874J-s001.pdf]

Supplementary Information (SI) for Chemical Science.
This journal is © The Royal Society of Chemistry

Supplementary Information

"Click" Formation of the Imine Bond in Water

Deepak, Tommaso Marchetti, Federico Rastrelli, Luca Gabrielli, Leonard J. Prins*

Department of Chemical Sciences, University of Padua

Via Marzolo 1, 35131 Padua, Italy

Contents

S1. General Methods 4

S2. Imine formation of aniline-derivative A with aldehydes 1-5 6

S2.1. Kinetics for the formation of **1-A** (equimolar, 0.5 mM) 6

S2.2. Stability of **1-A** at room Temperature 7

S2.3. Reaction kinetics of **1-A** formation (2 equivalent of 1, [A] = 0.5 mM) 8

S2.4. Reaction kinetics of **1-A** formation (2 equivalent of A, [1] = 0.5 mM) 9

S2.5. Reaction kinetics of **1-A** formation (2 equivalent of A, [1] = 0.5 mM) by UV-vis spectroscopy 10

S2.6. Characterization of imine **1-A** using ^{1}H NMR, ^{13}C NMR, ^{1}H-^{13}C HSQC, and HRMS 11

S2.7. Reaction kinetics for the formation of **3-A**, **4-A**, and **5-A** using ^{1}H NMR 13

S2.8. Role of Zn(II) in the formation of **1-A** 17

S2.9. Role of counter ion (Cl^-) of Zn(II) in the formation of **1-A** 18

S2.10. Stability of **1-A** Imine 20

S2.10.1. Stability of **1-A** with excess of TPEN (4 eq.) 20

S2.10.2. Sequestering of Zn(II) ion from **A** using TPEN 21

S2.10.3. Imine and aldehyde exchange kinetics with **1-A** 22

S2.10.4. Equilibration kinetics with a mixture of **1**, **A**, and **T** 23

S2.10.5. Equilibration kinetics between **1-A** and Girard's reagent **T** 25

S2.11. Role of pH in the kinetic for the formation of **1-A** 27

S2.12. Formation of imine **1-A** at lower concentrations 34

S2.13. Formation of imine **1-A** in a complex biochemical matrix 36

S3. Reversible imine bond formation in water using aldehyde 6 39

S3.1. Reaction kinetics for the formation of **6-B** 39

S3.2. Thermodynamic control in the formation of **6-B** 41

S3.3. Reaction kinetics for the formation of **6-C** 42

S3.4. Reaction kinetics for the formation of **6-D** 44

S3.5. Reaction kinetics for the formation of **6-E** 46

S3.6. Reaction kinetics for the formation of **6-F** 48

S3.7. Reaction kinetics for the formation of **6-G** 50

S3.8. Role of Zn(II) in the formation of **6-C** 52

S3.9. Kinetics for the formation of **6-C** at lower concentrations 54

S3.10. Kinetics of **6-C** formation with 3 equivalents of **C** 55

S3.11. Characterization of imines **6-B** and **6-C** 56

S4. Dynamic combinatorial library of imines in water 60

S4.1. Dynamic imine formation upon the stepwise addition of **B**, **C**, and **D** to aldehyde **6** 60

S4.2. Dynamic imine formation upon the simultaneous addition of **B**, **C**, and **D** to aldehyde **6** 62

S4.3. Distribution of dynamic combinatorial library composition based on equilibrium constants. 64
S4.4. Kinetic analysis of the dynamic exchange reactions regarding imines **6-B**, **6-C** and **6-D** 65
S5. Determination of rate constants and equilibrium constants 66
S6. DFT optimized geometry of 1-A and 6-B 69
S7. Synthesis of compounds 1 and 6 69
S8. Formation of 1-A imine in PBS buffer 81
S9. References 82

S1. General Methods

All commercially available reagents, including aniline, p-methoxyaniline, 2-fluoroaniline, benzylamine, 2-aminoethanol, pyridine-2-carboxaldehyde, pyridine-3-carboxaldehyde, pyridine-4-carboxaldehyde, benzaldehyde, Girard's reagent T, and tris(2-pyridylmethyl)amine (TPEN), were purchased from Merck or Tokyo Chemical Industry and used as received without further purification. HEPES buffer (4-(2-hydroxyethyl)-1-piperazineethanesulfonic acid), MES (2-(N-morpholino)ethanesulfonic acid, and CAPS (3-(cyclohexylamino)-1-propanesulfonic acid) buffer was obtained from Merck and used without additional purification. Zinc nitrate ($Zn(NO_3)_2$) was purchased as an analytical-grade reagent, and the concentration of its stock solution was determined by inductively coupled plasma (ICP) analysis. Stock solutions of compounds **A** and **6** (in the absence of Zn(II)) were prepared in D_2O by weight. Their effective concentrations were determined using sodium trimethylsilylpropanesulfonate (DSS) as an internal standard. Stock solutions of pyridine regioisomers and aniline derivatives were also prepared in D_2O at an effective concentration of 50 mM. For poorly soluble compounds in D_2O such as p-methoxyaniline and TPEN, one equivalent of deuterium chloride was added solely to improve solubility.

NMR spectroscopy: ^1H and ^{13}C NMR spectra were recorded on a Bruker Avance Neo 500 spectrometer (500 MHz for ^1H and 125 MHz for ^{13}C). Spectra acquired in D_2O were recorded with water suppression using a presaturation pulse sequence (zgpr) when required. Chemical shifts (δ) are reported in ppm and referenced to the residual solvent signal (D_2O: δ = 4.79 ppm). Data processing was performed using MestReNova (version 14.2). Signal multiplicities are reported as singlet (s), doublet (d), triplet (t), doublet of doublets (dd), and multiplet (m).

NMR Kinetic Experiments: Stock solutions of all components were prepared in D_2O and combined to a final volume of 600 µL in an NMR tube. The reaction mixture consisted of HEPES buffer (60 µL, 100 mM stock solution, pH_{obs} = 7.0, pD = 7.4), $Zn(NO_3)_2$ (6 µL, 50 mM stock solution), aldehyde **1**–**5** (6 µL, 50 mM stock solution), and aniline **A** (6 µL, 50 mM stock solution). Aniline **A** was added as the final component to define the starting point of the reaction. DSS (sodium 3-(trimethylsilyl) propane-1-sulfonate) was used as an internal standard at a concentration of 0.5 mM. For kinetic measurements, the initial time point was approximated as 10 min, accounting for ~7 minute acquisition time for the first spectrum and ~3 minute required for instrument locking and shimming. Subsequent spectra were recorded using the Bruker "multizg" acquisition mode, where only the first experiment required locking and shimming. Each subsequent spectrum was acquired with a fixed time interval of ~ 7 min, enabling the monitoring of reaction progress**.**

Kinetics with UV-visible spectroscopy: Stock solutions of all components were prepared in D_2O and directly taken in a 1 mL quartz cuvette of path length 1 cm to a final volume of 600 µL. The reaction conditions were matched to those used in the corresponding NMR experiments. Imine formation was monitored by recording time-dependent UV-vis absorption spectra. Following completion of each kinetic experiment, ^1H NMR measurements were performed to confirm the extent of imine formation. Correlation of UV-vis absorbance with NMR-derived concentrations enabled determination of the molar extinction coefficients (ε). For kinetic measurements, aldehyde was first introduced into the cuvette, and the reaction was initiated by addition of aniline **A** immediately before the starting of the experiment. In the control experiment aimed at examining the role of Zn(II), initial spectra were recorded in the absence of metal ions. Subsequently, $Zn(NO_3)_2$ was added in equimolar amounts during the experiment, and the reaction progress was further monitored under identical conditions.

pH Measurements: The apparent pH of buffer solutions in D_2O was determined at room temperature using a Metrohm-632 pH-meter equipped with an Ag/AgCl/KCl reference electrode and corrected for the isotope following $pD = pH_{obs}+0.4$.

DFT calculations: All DFT calculations were run using the software package Gaussian16.[1] A first geometry optimization was run at the b3lyp/6-31g(d,p) level of theory, followed by a refinement at the wb97xd/6-31g(d,p) level of theory. The resulting geometry of imines **1-A** and **6-B** was checked to be a true minimum by a frequency calculation. The long-range solvent effects (water) were included in the optimization step by using the Polarizable Continuum Model (PCM).[2] NMR calculations on the optimized geometry were run at the B3LYP/cc-pVTZ level of theory. ^{1}H chemical shifts were referenced against the shielding constants of tetramethylsilane protons calculated at the same level of theory.

S2. Imine formation of aniline-derivative A with aldehydes 1-5

S2.1. Kinetics for the formation of 1-A (equimolar, 0.5 mM)

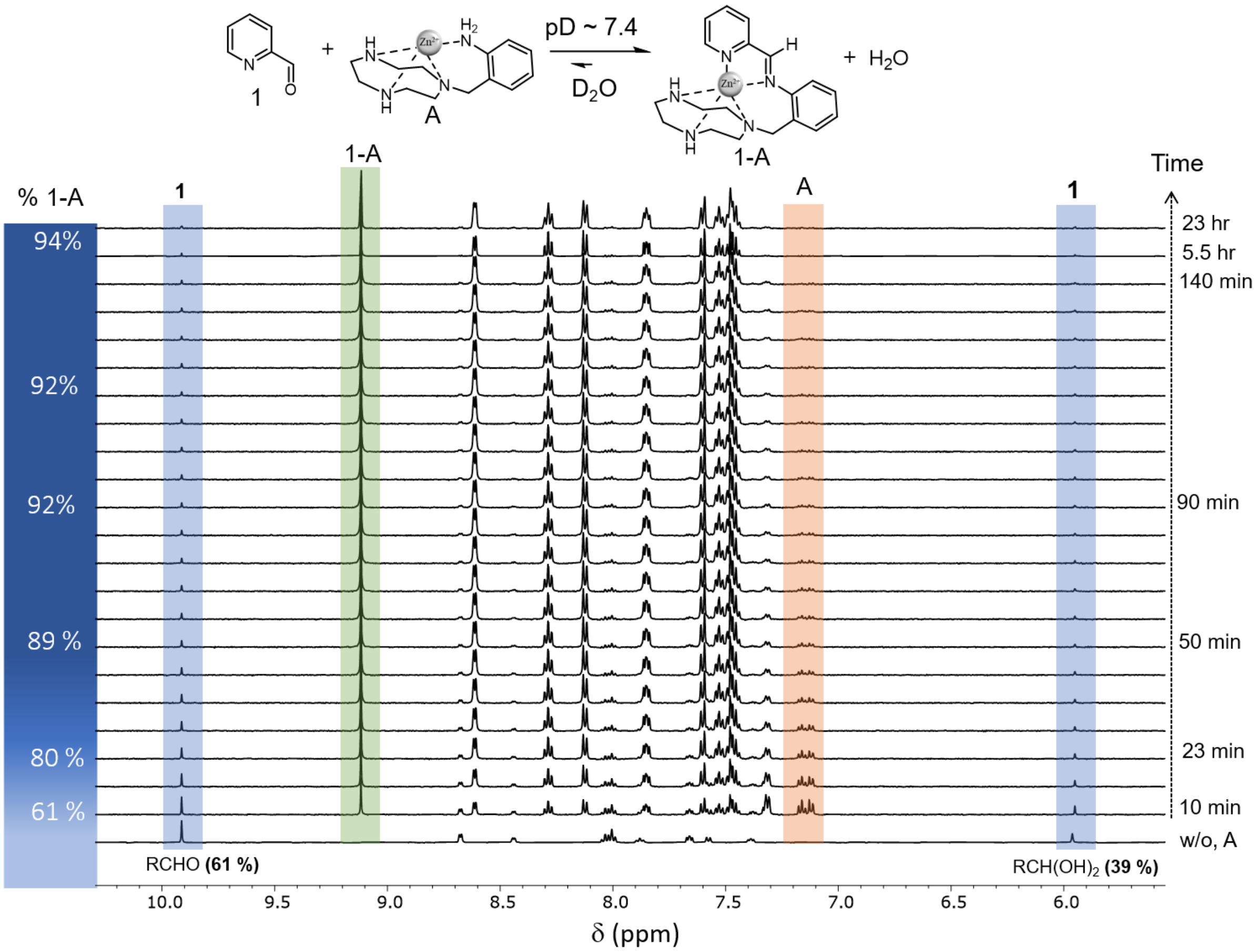

Figure S1. Parts of the ^{1}H spectra (500 MHz, D_2O) recorded between the indicated time intervals after mixing **1** and **A** (25 °C, pD = 7.4, [C] = 0.5 mM) in HEPES buffer (10 mM). The blue rectangles highlight the aldehyde (69 %) and hydrated aldehyde (39 %) peaks of **1**, the red rectangle highlights the peak corresponds to **A**, and the green rectangle highlights the imine **1-A**. Percentage conversion is determined using the combined integration of aldehyde, hydrated aldehyde and imine.

S2.2. Stability of 1-A at room Temperature

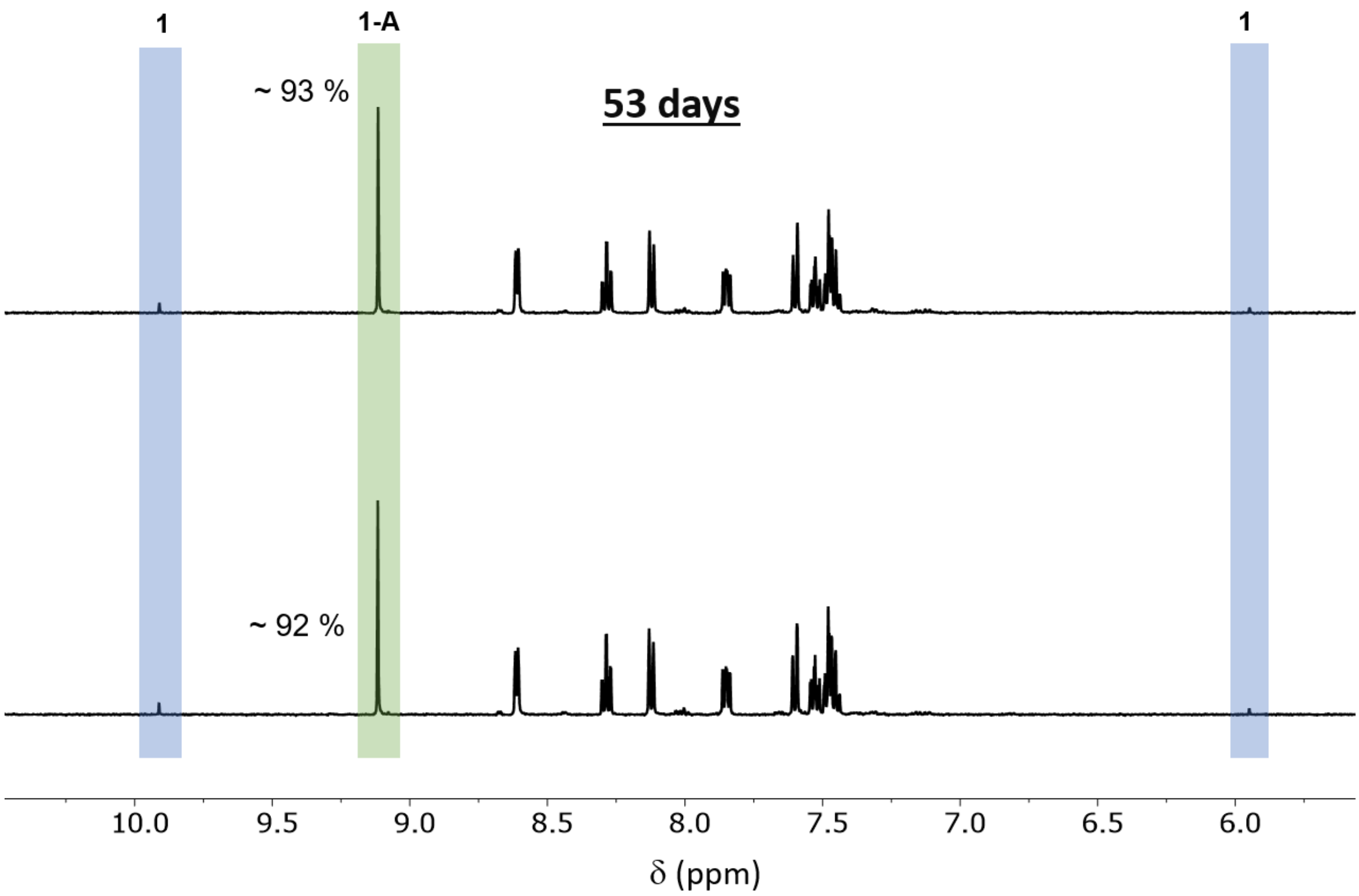

Figure S2. Parts of the ^{1}H NMR spectra (500 MHz, D_2O) recorded 53 days after formation of imine **1-A** ([**1**] = [**A**] = 0.5 mM, 10 mM HEPES buffer, pD = 7.4). The blue rectangles correspond to the aldehyde peak and hydrated aldehyde peak of **1.** The green highlighted region corresponds to the **1-A** imine. No significant changes are observed, indicating long-term stability of the imine **1-A**.

S2.3. Reaction kinetics of 1-A formation (2 equivalent of 1, [A] = 0.5 mM)

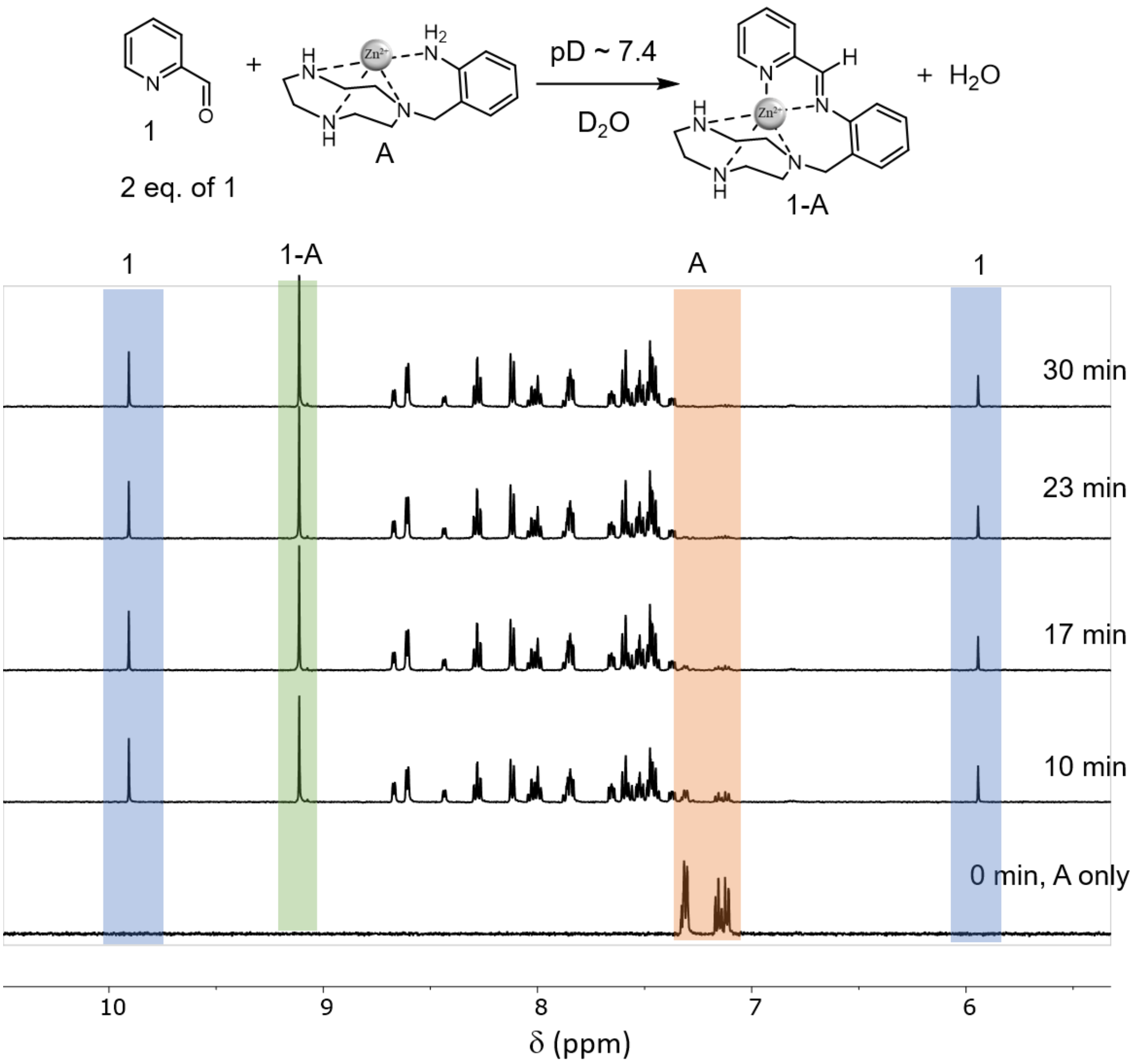

Figure S3. Parts of the ^1H spectra (500 MHz, D_2O) recorded between the indicated time intervals after mixing **1** (1.0 mM) and **A** (0.5 mM) (25 °C, pD=7.4, [C] = 0.5 mM) with 10 mM of HEPES buffer. The blue rectangles highlight the aldehyde and hydrated aldehyde peaks, red rectangle correspond to **A** and the green rectangles highlights the imine peak corresponding to **1-A**.

S2.4. Reaction kinetics of 1-A formation (2 equivalent of A, [1] = 0.5 mM)

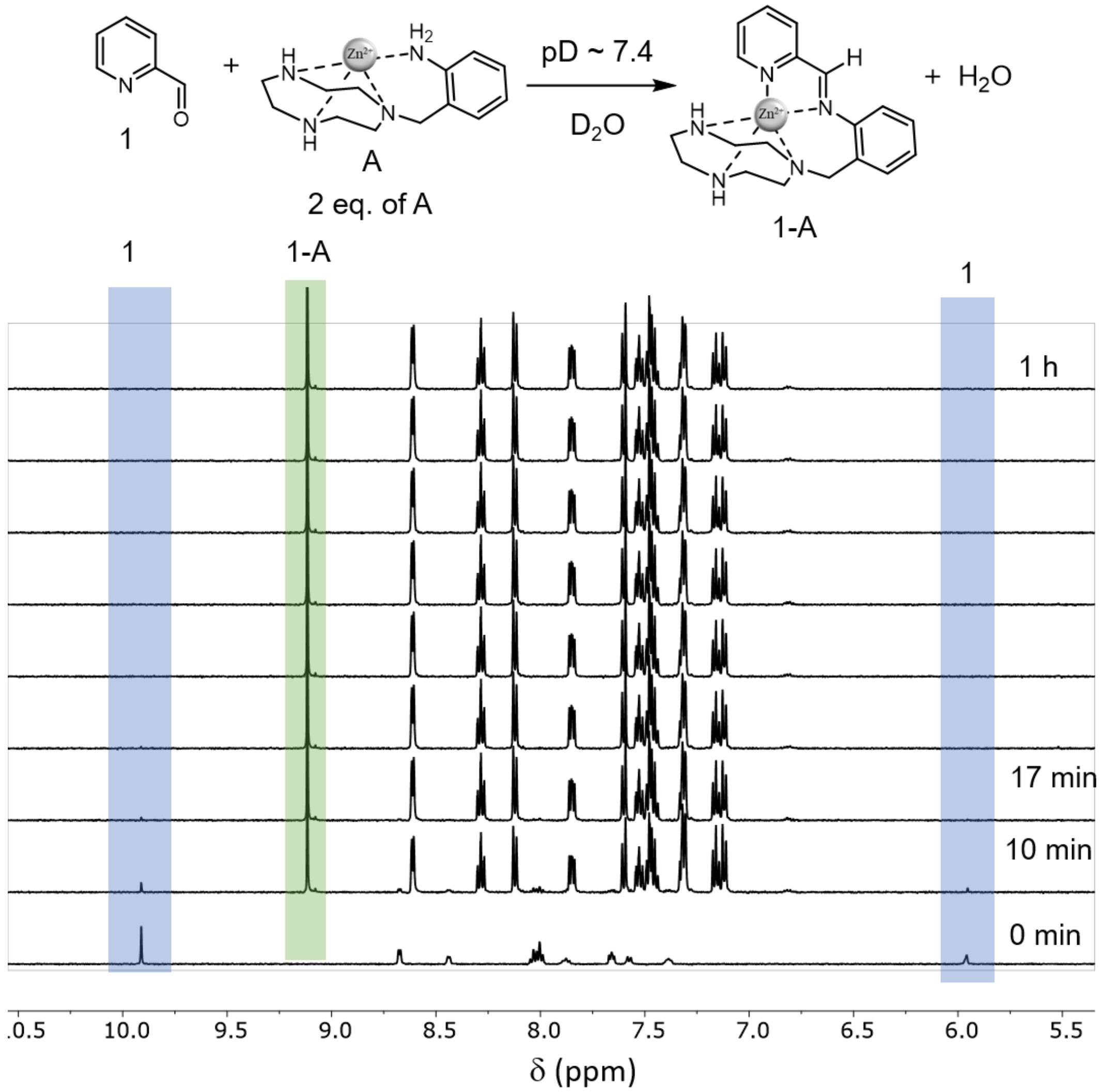

Figure S4. Parts of the ^{1}H spectra (500 MHz, D_2O) recorded between the indicated time intervals after mixing **1** (0.5 mM) and **A** (1.0 mM) (25 °C, pD = 7.4, [C] = 0.5 mM) with 10 mM of HEPES buffer. The blue rectangles highlight the aldehyde and hydrated aldehyde peaks and the green rectangles highlights the imine peak corresponding to **1-A**.

S2.5. Reaction kinetics of 1-A formation (2 equivalent of A, [1] = 0.5 mM) by UV-vis spectroscopy

When 2 equivalents of **A** were used the kinetics of imine formation by ^{1}H NMR spectroscopy proved challenging due to the relatively long acquisition time required to obtain spectra with sufficient signal to noise (~10 min for the first spectrum and ~7 min for subsequent spectra). Complete consumption of **1** was observed within approximately 20 minutes corresponding to only a few measurable data points. For that reason, UV-vis spectroscopy was employed under identical concentration conditions ([**1**] = 0.5 mM). Imine formation was immediately observed upon mixing aldehyde **1** and **A** in the cuvette, with a characteristic absorption band appearing at 350 nm. However, due to the high absorbance at this wavelength, signal saturation occurred rapidly. Therefore, kinetic measurements were performed by monitoring the absorbance at 375 nm, which allowed reliable monitoring of the formation of the **1-A** imine complex. The molar extinction coefficient (ε) at 375 nm was determined by correlating the absorbance with imine concentrations obtained from ^{1}H NMR (Figure S2).

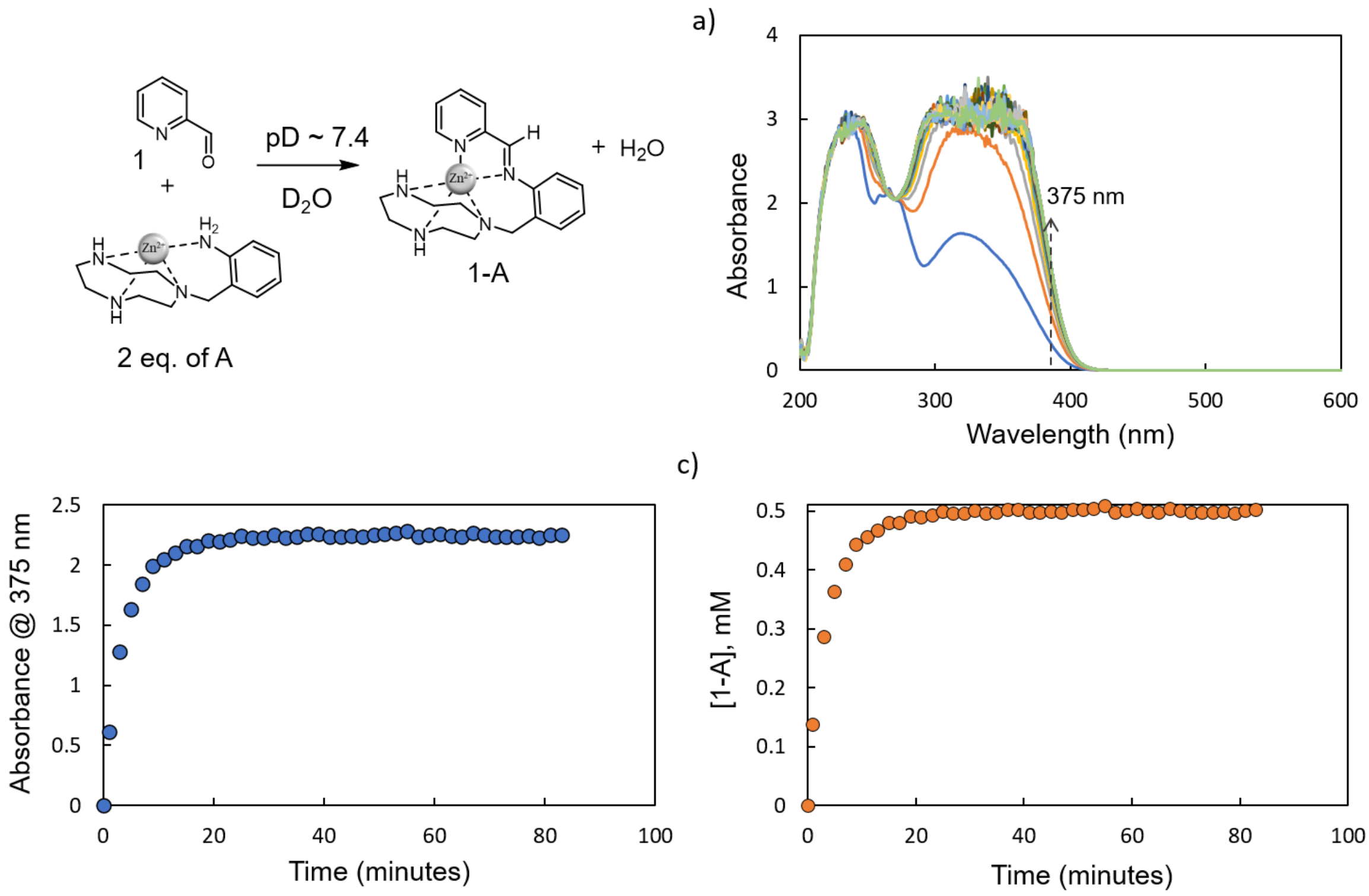

Figure S5. Kinetic measurment of **1-A** formation, monitored by UV-vis spectroscopy in aqueous conditions ([**1**] = 0.5 mM, [A] = 1.0 mM, 10 mM HEPES buffer in D_2O, pD = 7.4). (a) Changing in the UV-vis spectra showing the formation of imine. (b) Absorbance at 375 nm as a function of time, corresponding to **1-A**. (c) Concentration of **1-A** versus time, determined from UV-vis data using the molar extinction coefficient (ε) obtained from ^{1}H NMR (Figure S4).

S2.6. Characterization of imine 1-A using ^{1}H NMR, ^{13}C NMR, ^{1}H-^{13}C HSQC, and HRMS

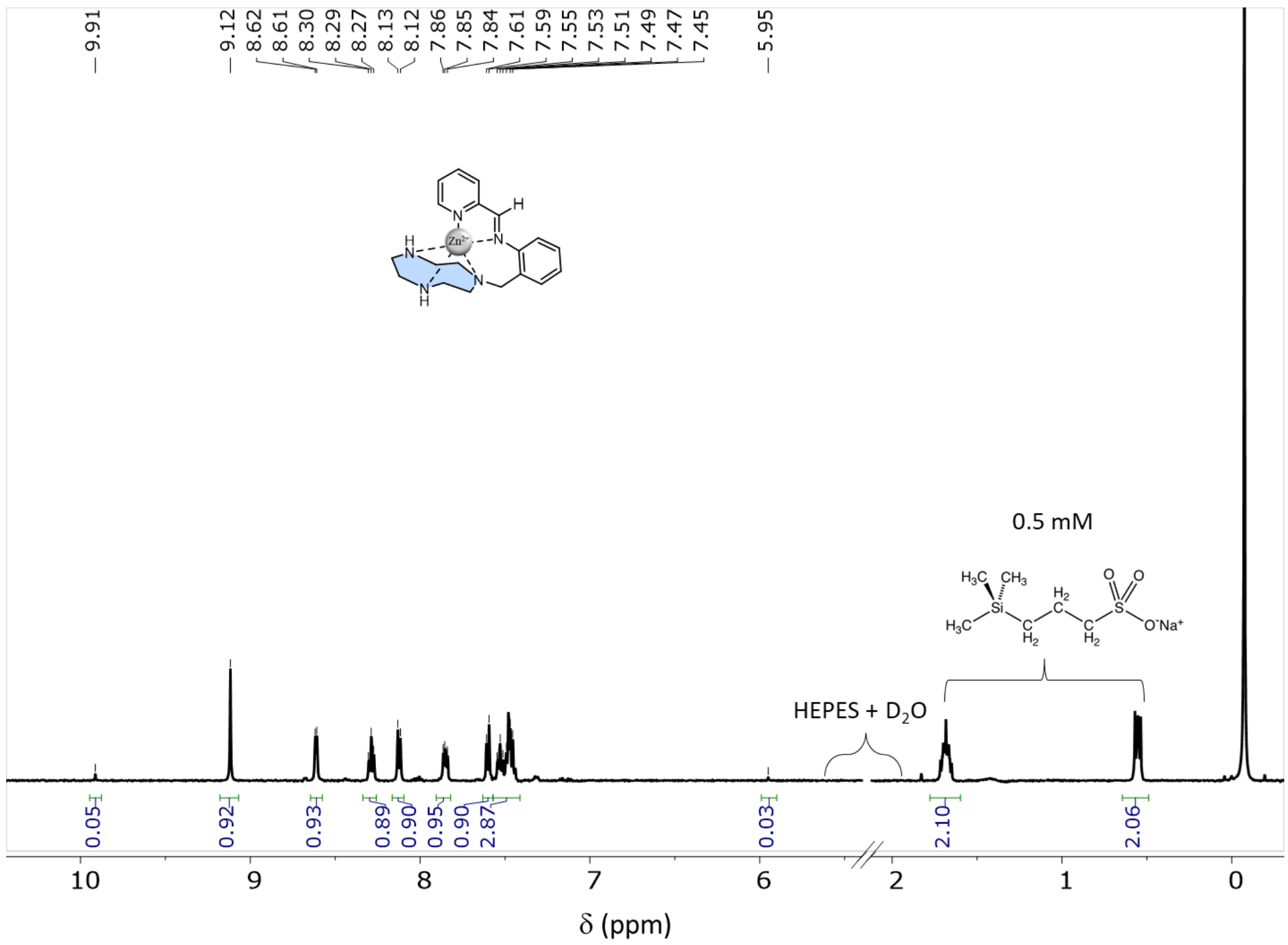

Figure S6. ^{1}H NMR spectrum of imine **1-A** imine, [1-A] = 0.5 mM, 10 mM HEPES, D_2O and 25 °C.

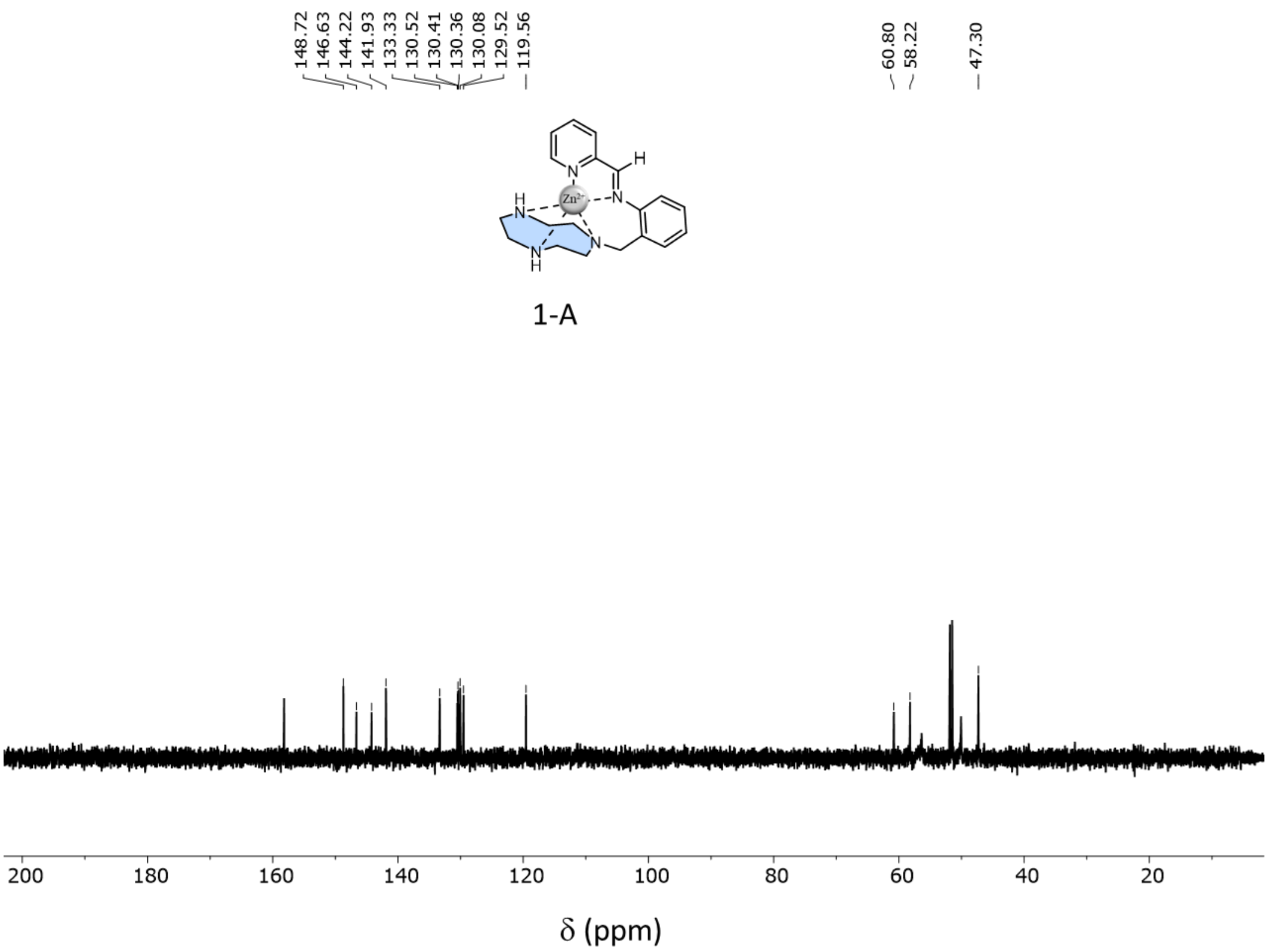

Figure S7. ^{13}C NMR spectrum of **1-A** imine complex, [1-A] = 5 mM, 10 mM HEPES, D_2O and 25 °C.

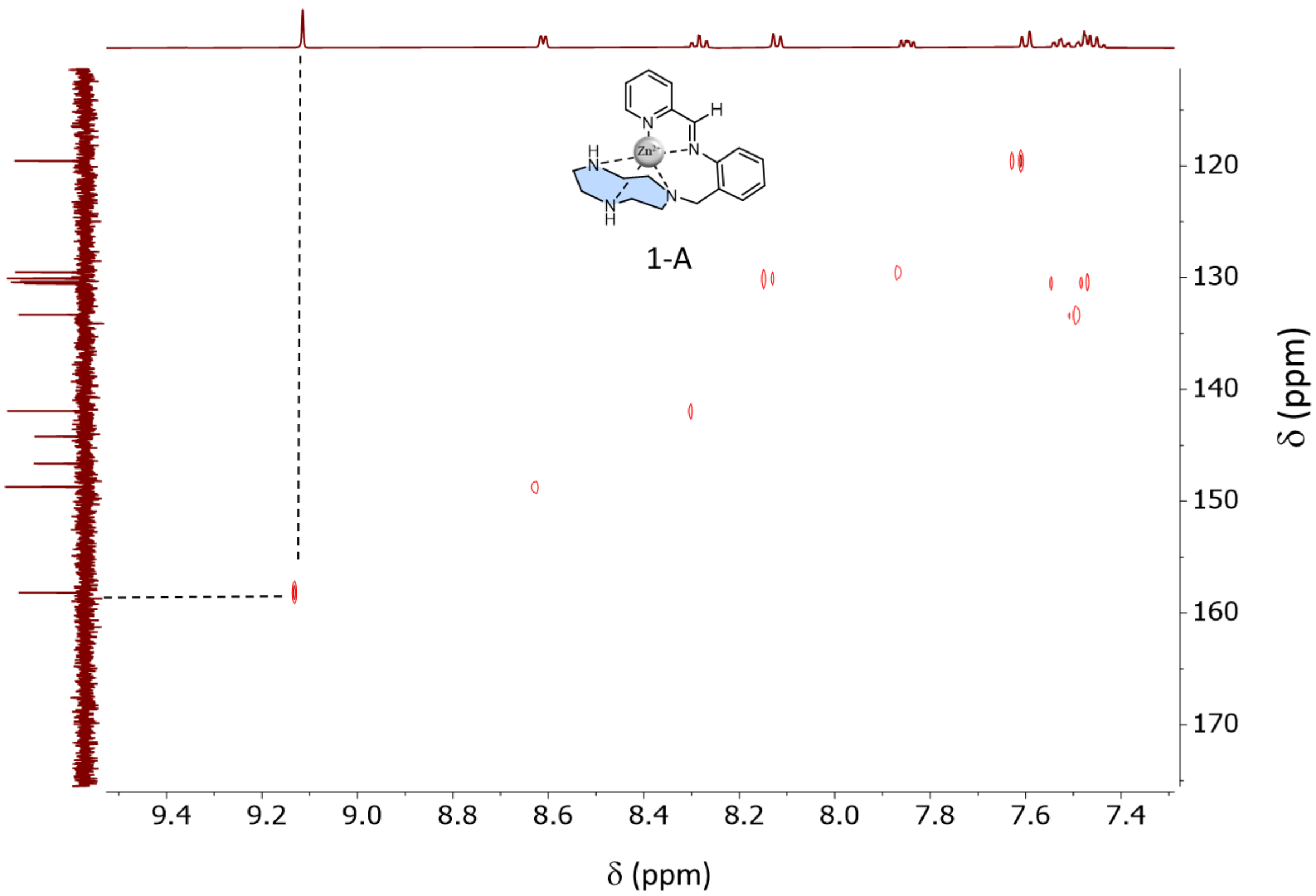

Figure S8. ^{1}H-^{13}C HSQC spectrum of imine **1-A**, [1-A] = 2.5 mM, 10 mM HEPES, D_2O and 25 °C.

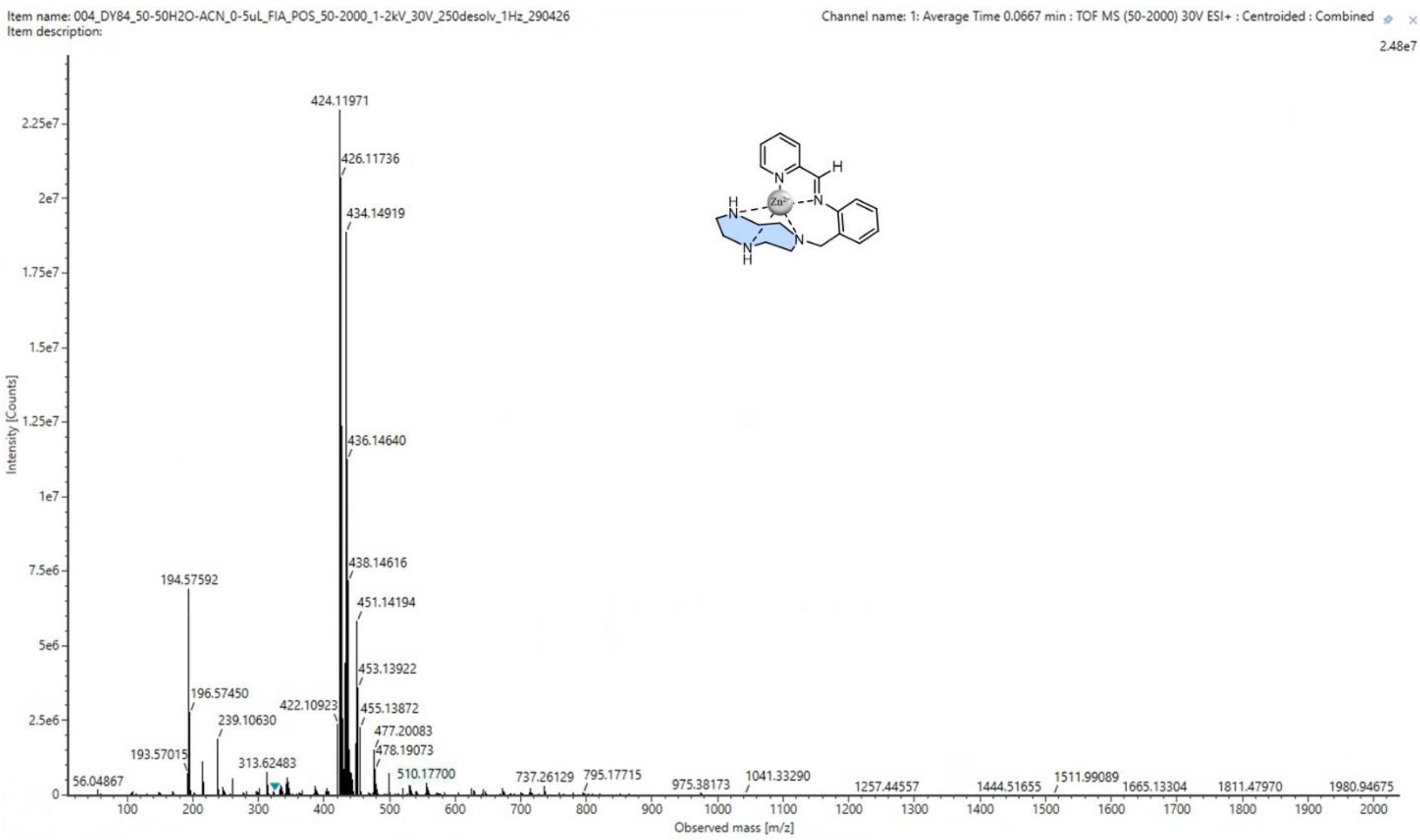

Figure S9. HRMS spectrum of imine **1-A**. Calculated Mass, [M+Cl] = 422.1084

S2.7. Reaction kinetics for the formation of 3-A, 4-A, and 5-A using ^{1}H NMR

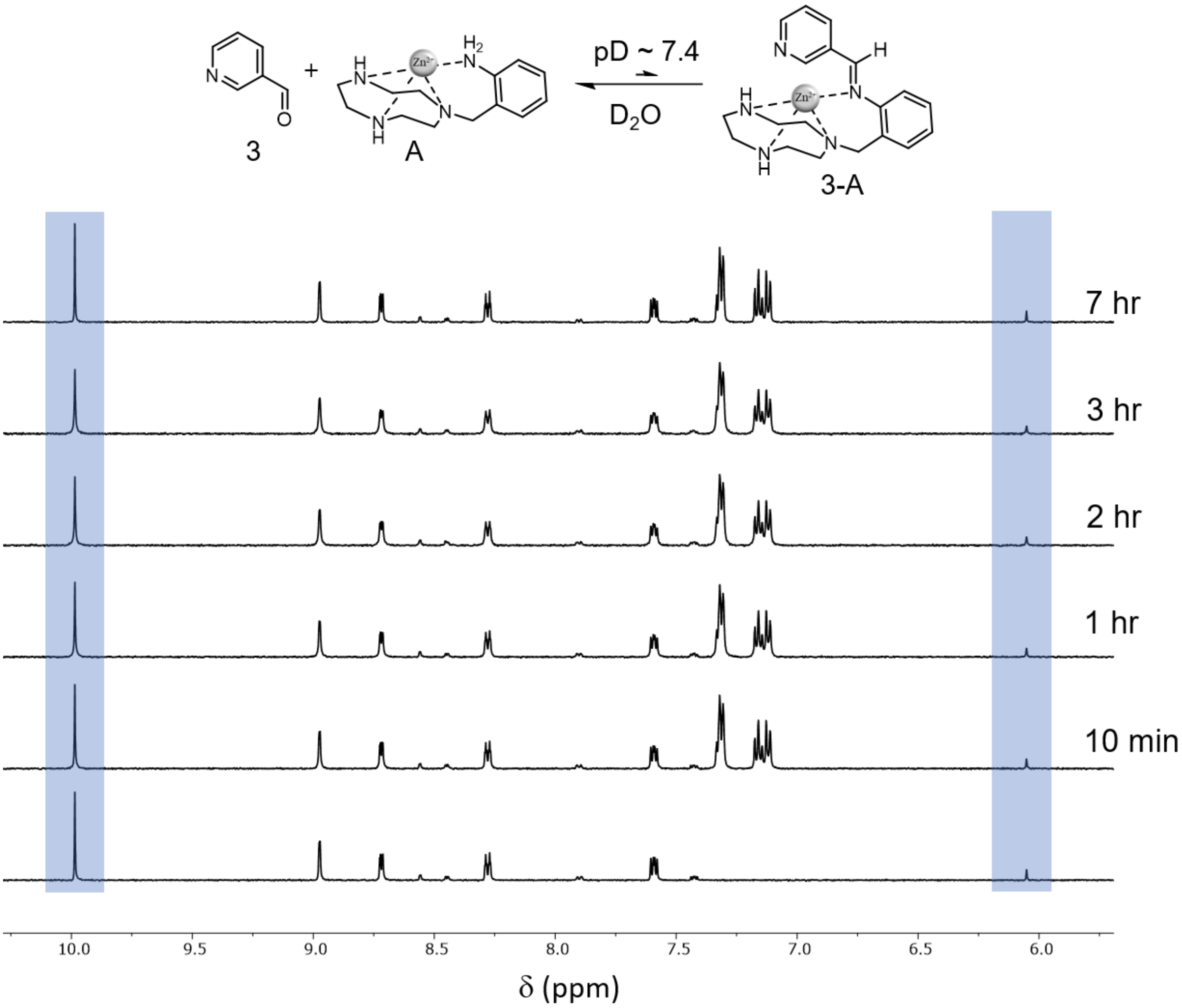

Figure S10. Parts of the ^{1}H NMR spectra (500 MHz, D_2O) recorded with the indicated time intervals following mixing of **3** and **A** ([**3**] = [**A**] = 0.5 mM, pD = 7.4) in 10 mM HEPES buffer. The blue rectangles highlight the aldehyde and hydrated aldehyde signals. No traces of imine **3-A** were observed under these conditions.

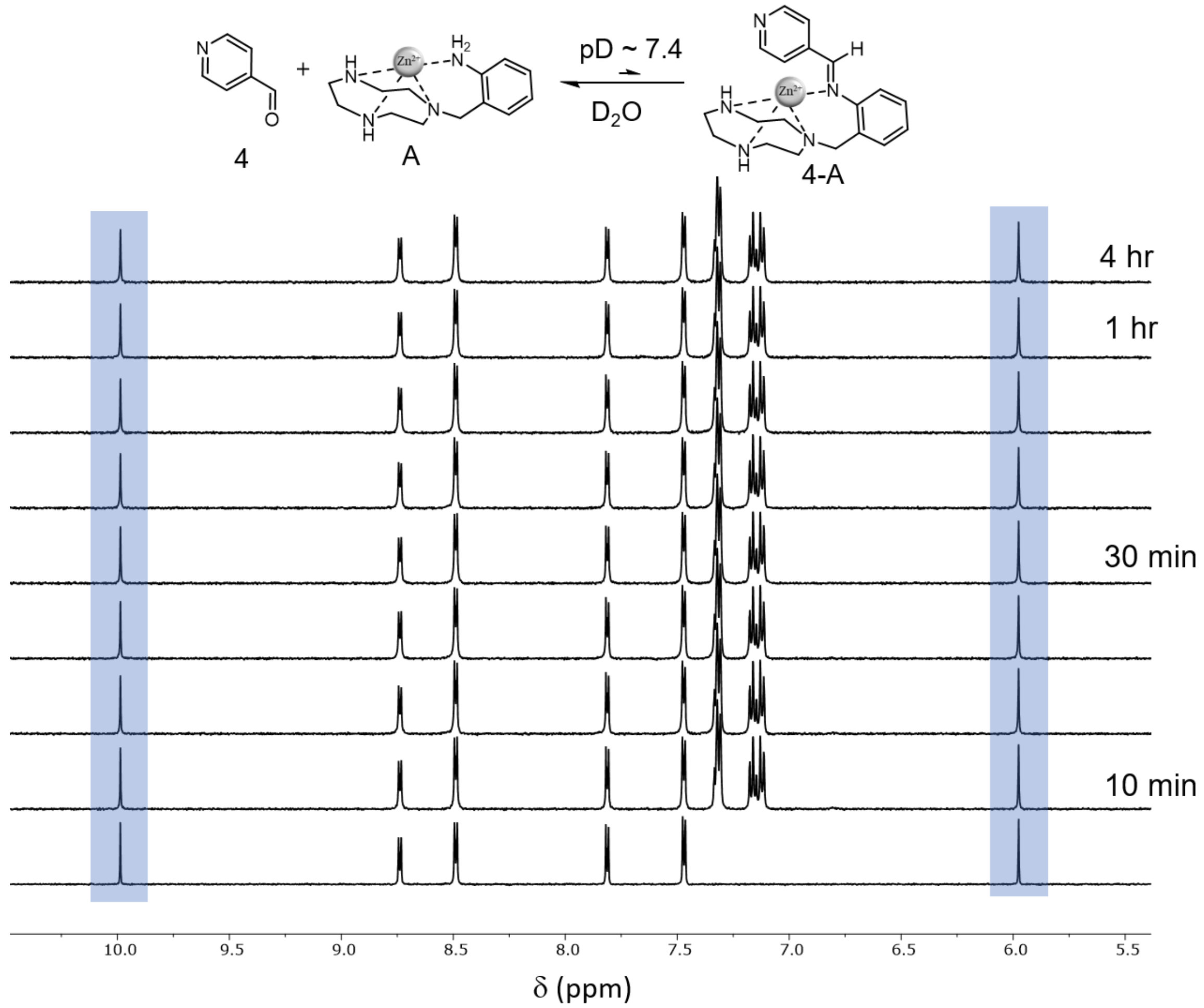

Figure S11. Parts of ^{1}H NMR spectra (500 MHz, D_2O) recorded with the indicated time intervals following mixing of **4** and **A** ([**4**] = [**A**] = 0.5 mM, pD = 7.4) in 10 mM HEPES buffer. The blue rectangles highlight the aldehyde and hydrated aldehyde signals. No traces of imine **4-A** were observed under these conditions.

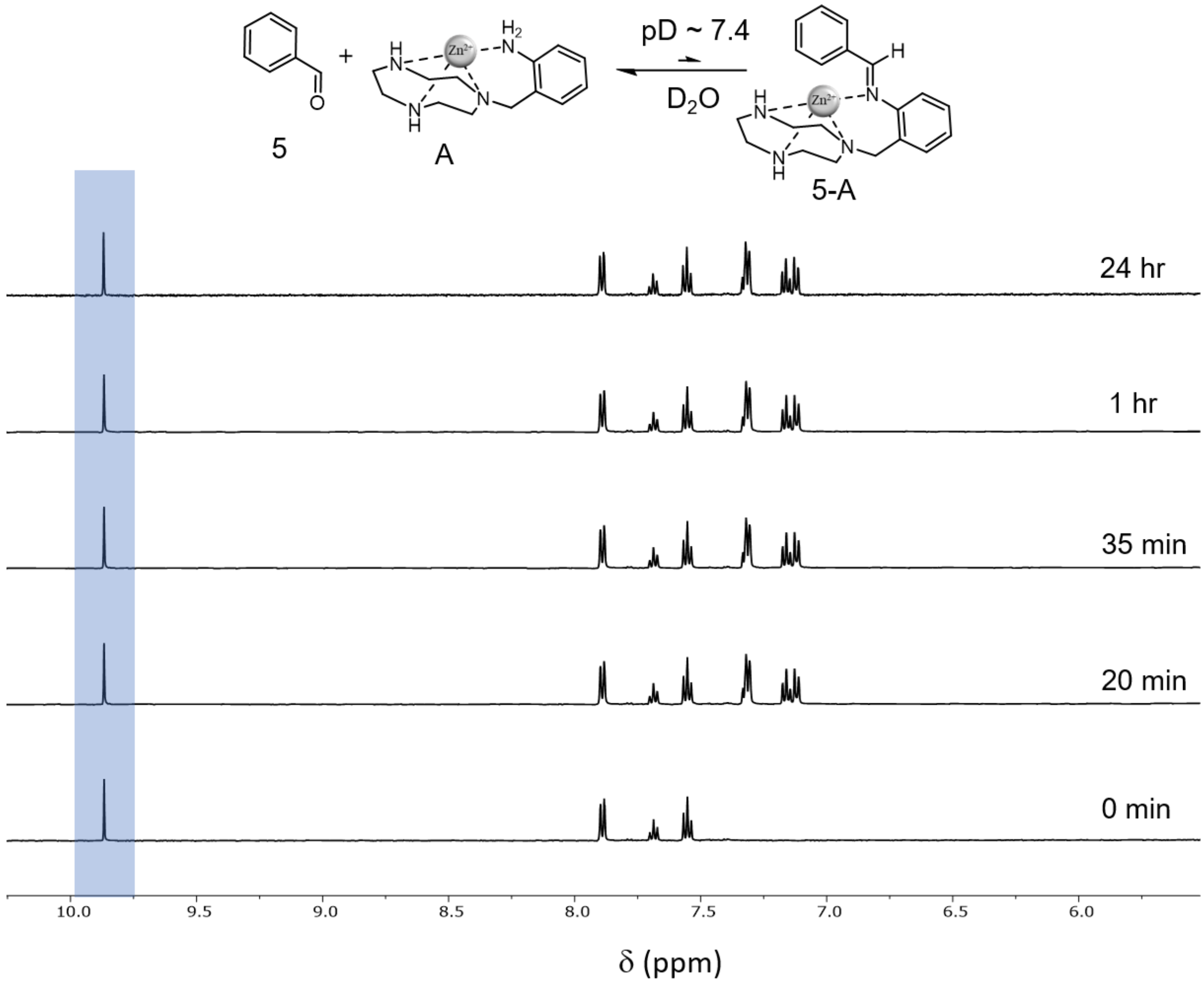

Figure S12. Parts of ^{1}H NMR spectra (500 MHz, D_2O) recorded with the indicated time intervals following mixing of **5** and **A** ([**5**] = [**A**] = 0.5 mM, pD = 7.4) in 10 mM HEPES buffer. The blue rectangle highlights the aldehyde signal. No traces of the imine **5-A** were observed under these conditions.

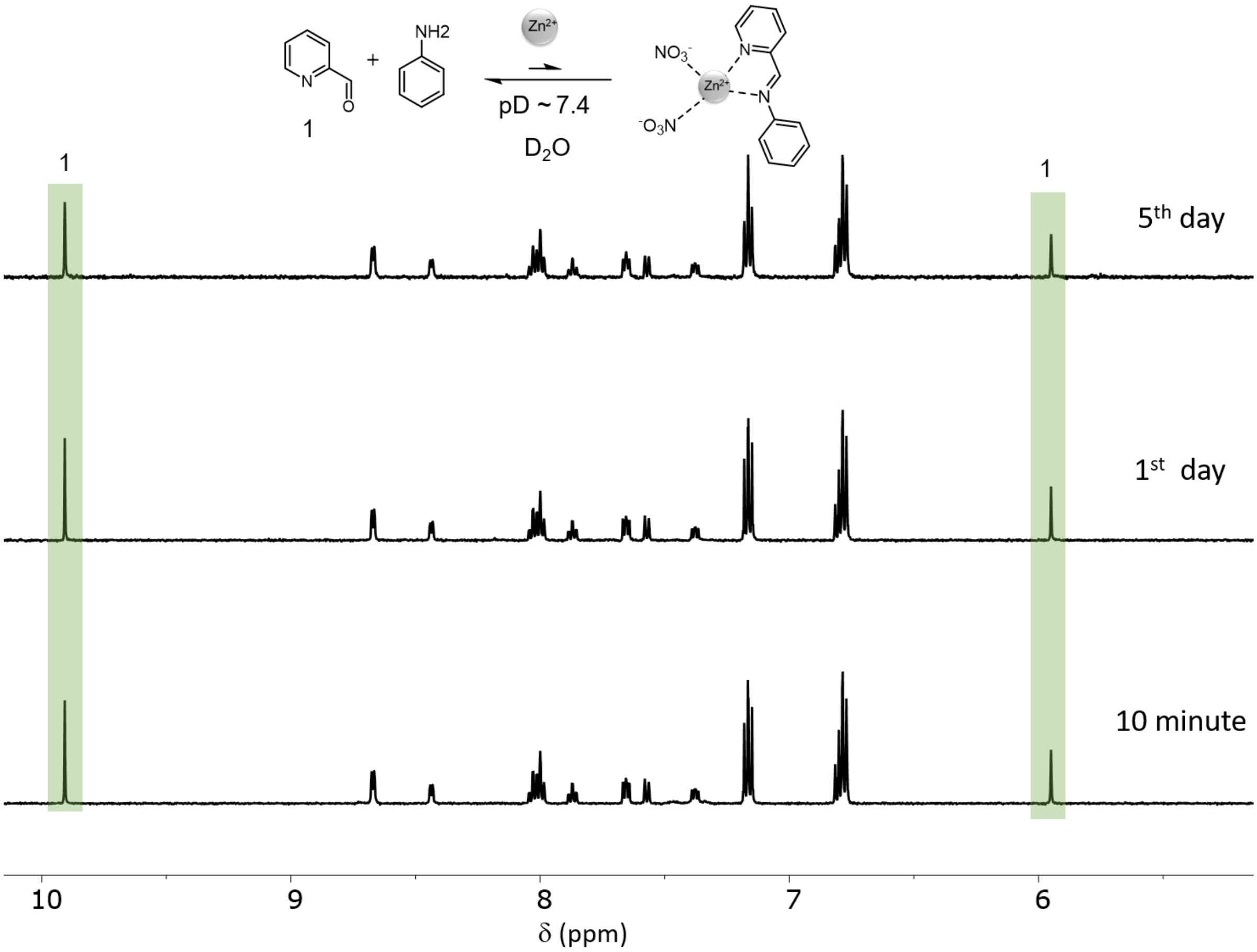

Figure S13. Parts of ^{1}H NMR spectra (500 MHz, D_2O) recorded with the indicated time intervals following mixing of **1** and **aniline** ([**1**] = [**Aniline**] = [$Zn(NO_3)_2$] = 0.5 mM, pD = 7.4) in 10 mM HEPES buffer. The green rectangle highlights the aldehyde signal. No traces of the imine were observed under these conditions.

S2.8. Role of Zn(II) in the formation of 1-A

The experiment was conducted using UV-vis spectroscopy to evaluate imine formation in the absence and presence of Zn(II). Initially, no metal ion was added, and no characteristic absorption band around 350 nm corresponding to the formation of imine **1-A** was observed After 10 min, an equimolar amount of $Zn(NO_3)_2$ was introduced, resulting in the immediate appearance of the imine absorption band which saturated almost completely within 1 h.

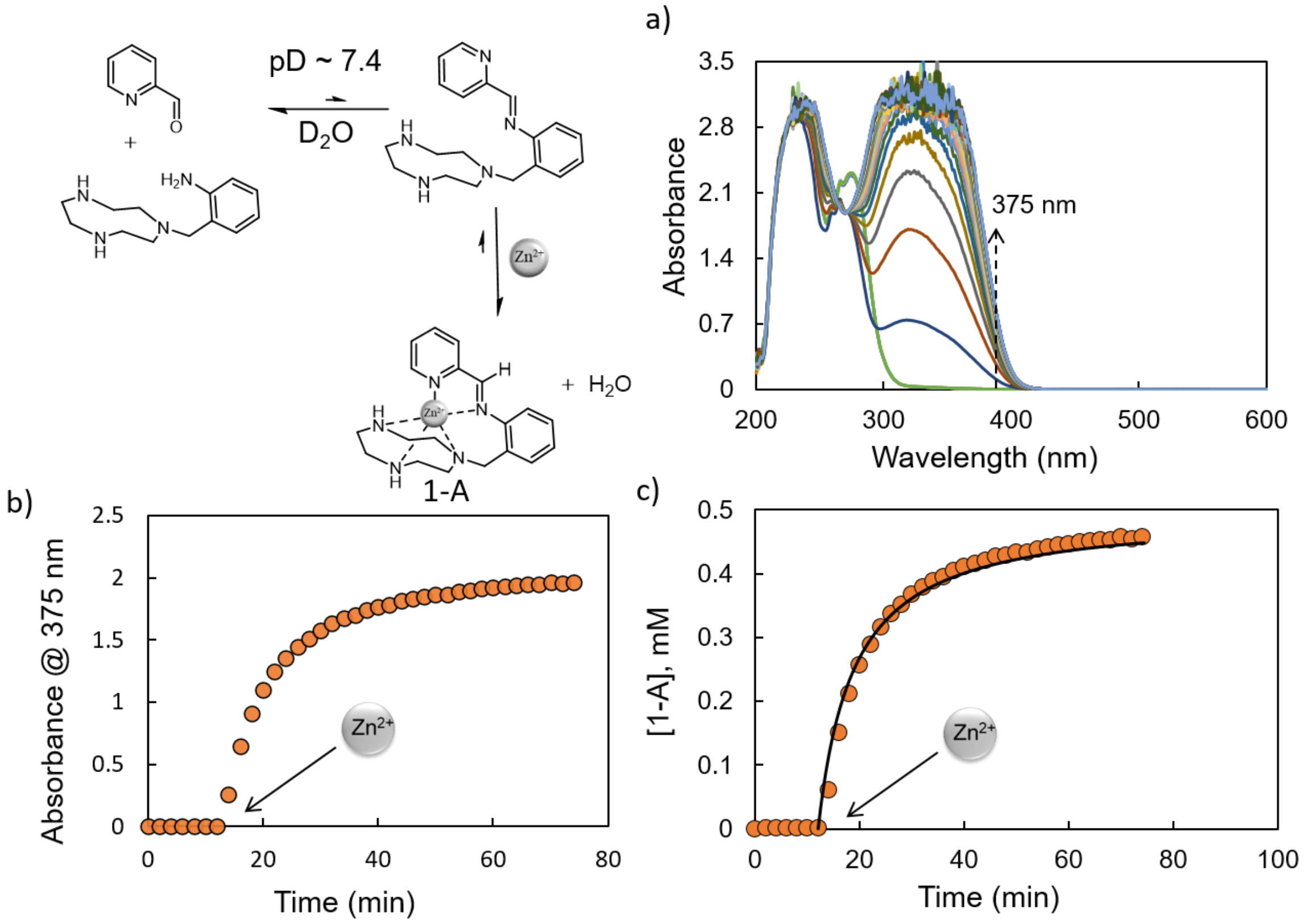

Figure S14. Role of Zn(II) in the activation and preorganization during **1-A** formation monitored by UV-vis spectroscopy. a) Changes in the UV-vis spectra with the formation of **1-A**. b) Absorbance at 375 nm as a function of time in the presence and absence of Zn(II). c) Concentration of imine **1-A** versus time, obtained from UV-vis absorption data using the molar extinction coefficient determined by correlating ^{1}H NMR kinetics (Figure S1).

S2.9. Role of counter ion (Cl^-) of Zn(II) in the formation of 1-A

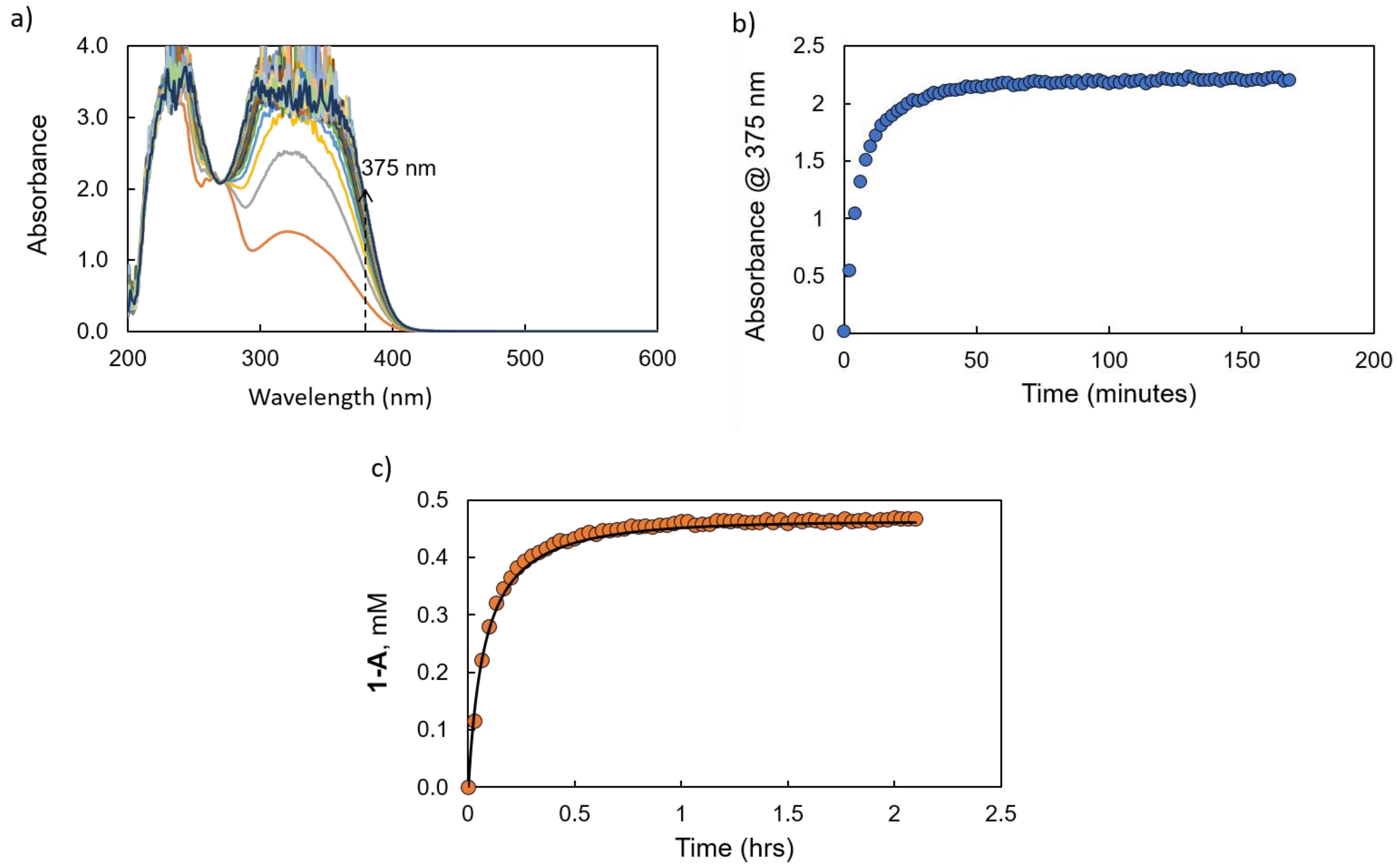

Figure S15. Kinetic measurment of **1-A** formation, monitored by UV-vis spectroscopy in aqueous conditions ([**1**] = **[A]** = $ZnCl_2$ = 0.5 mM, 10 mM HEPES buffer in D_2O, pD = 7.4). (a) Changing in the UV-vis spectra showing the formation of imine. (b) Absorbance at 375 nm as a function of time, corresponding to **1-A**. (c) Concentration of **1-A** versus time (k_f = 6.8 $M^{-1}s^{-1}$), determined from UV-vis data using the molar extinction coefficient (ε) obtained from ^{1}H NMR (Figure S16).

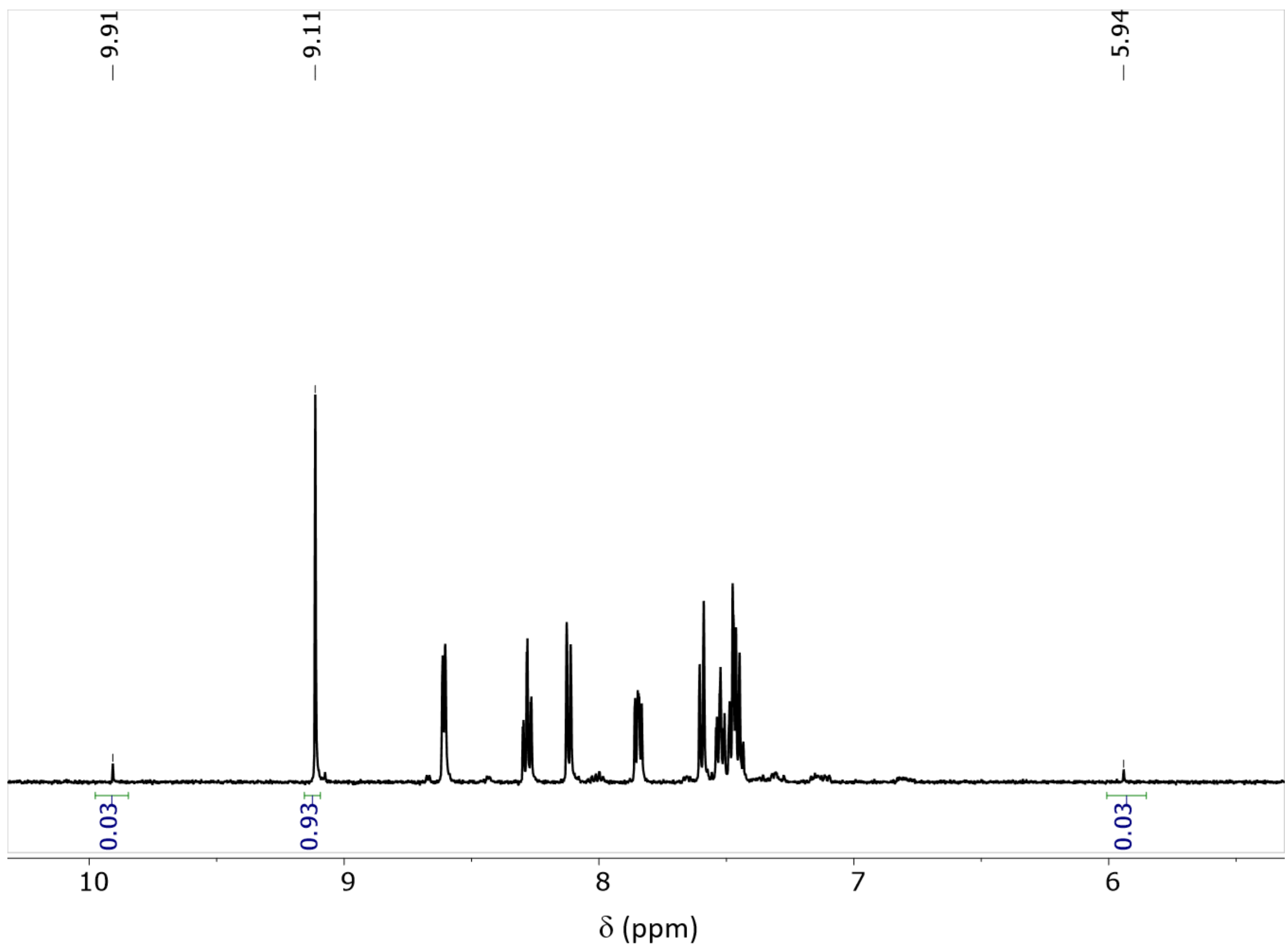

Figure S16. Part of ^{1}H NMR spectra (500 MHz, pD = 7.4, 10 mM HEPES in D_2O) recorded after the UV-visible kinetic experiment (Figure S15).

S2.10. Stability of 1-A Imine

S2.10.1. Stability of **1-A** with excess of TPEN (4 eq.)

N,N,N′,N′-tetrakis(2-pyridylmethyl)ethylenediamine (TPEN), a high-affinity Zn(II) chelating ligand, was employed to probe the stability of the **1-A** imine complex. Excess TPEN (2 mM, 4 equivalents) was added to a preformed solution of **1-A** (0.5 mM). Chelation of Zn(II) by TPEN would results in dissociation of the imine into precursors **1** and **A**. However, only 10 % imine dissociation were observed over 26 h, indicating that Zn(II) is strongly chelated within the **1-A** complex.

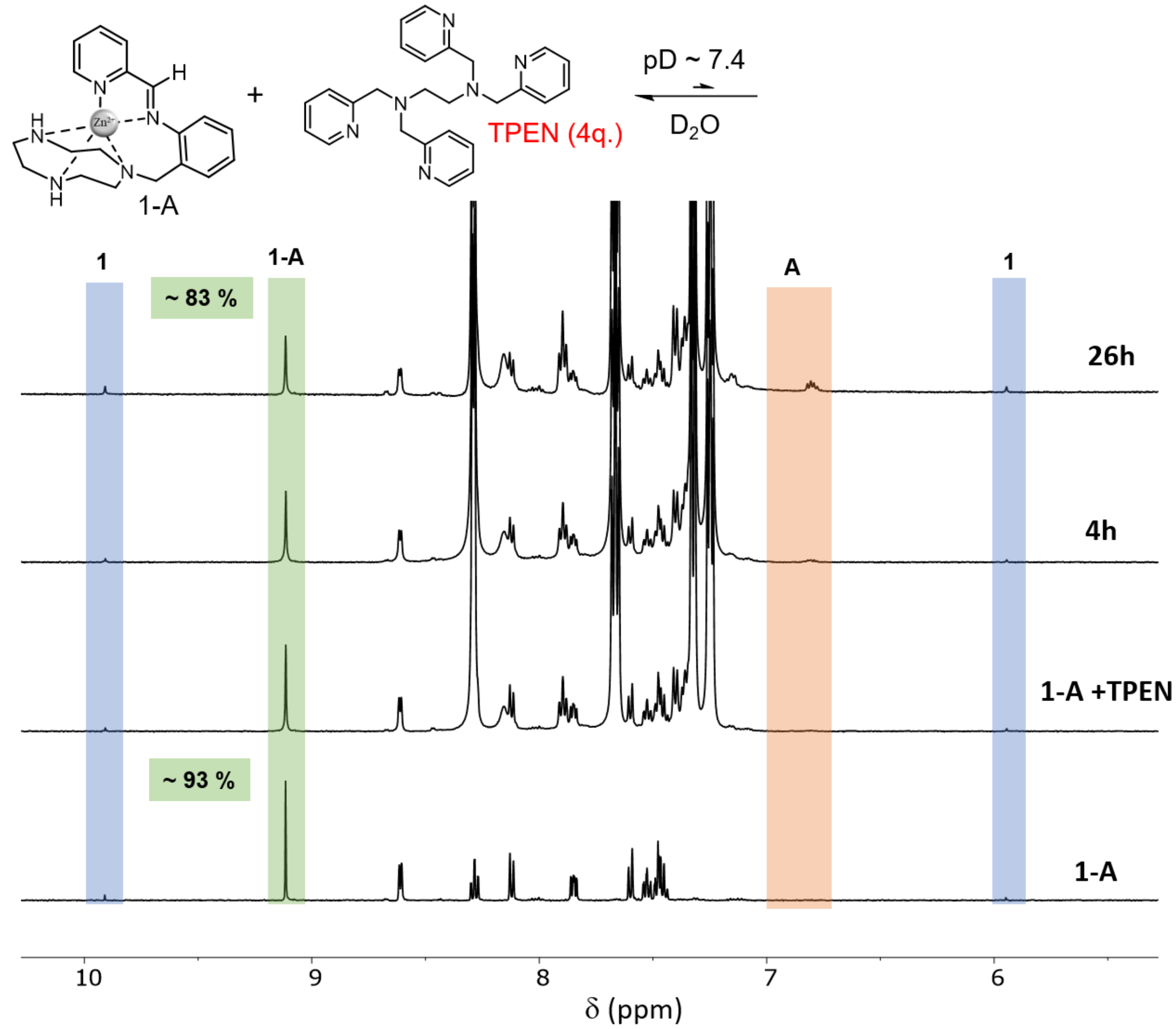

Figure S17. Parts of ^{1}H NMR spectra (500 MHz, D_2O) recorded over the indicated time intervals following mixing of **1-A** and excess of TPEN ligand ([**1-A**] = 0.5 mM, 10 mM HEPES buffer, pD = 7.4). The blue rectangles correspond to the aldehyde peak and hydrated aldehyde peak of **1.** The green rectangle showing peak corresponding to **1-A** at several time intervals.

S2.10.2. Sequestering of Zn(II) ion from **A** using TPEN

A control experiment was performed to compare the Zn(II)-binding strength of the chelated complex **1–A** with that of the precursor **A**. Upon addition of Zn(II) ions to **A** at a concentration of 0.5 mM in 10 mM HEPES buffer (D_2O, pD = 7.4), the characteristic resonances of **A** shifted downfield, consistent with Zn(II) coordination. Subsequent addition of TPEN revealed that one equivalent of the ligand was sufficient to sequester Zn(II) from the precursor **A**, as evidenced by the reshifting of the corresponding ^{1}H NMR signals (Figure 18).

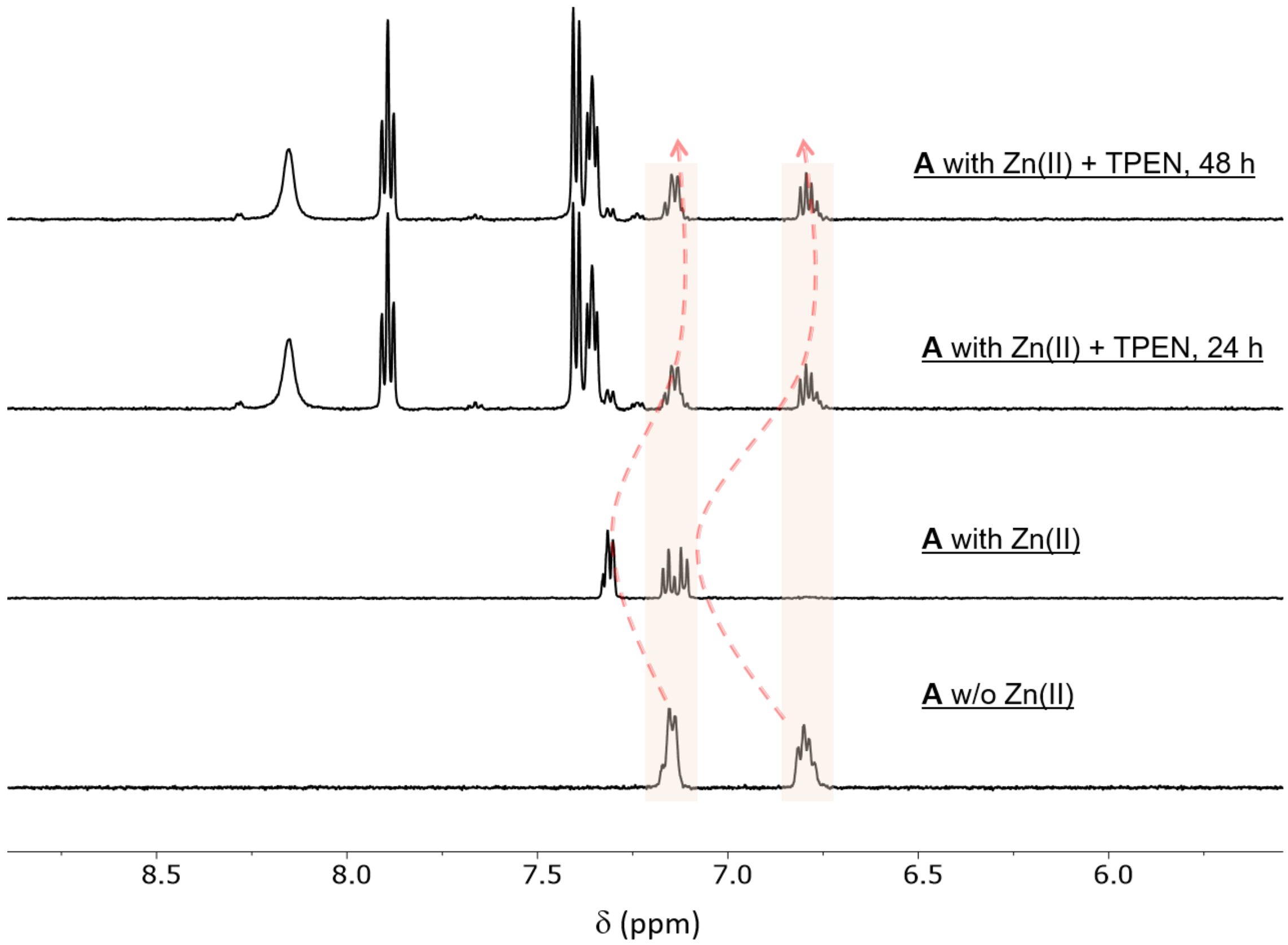

Figure S18. Parts of ^{1}H NMR spectra (500 MHz, D_2O) recorded over the indicated time intervals following mixing of **A** with Zn (II) ion and TPEN ligand.

S2.10.3. Imine and aldehyde exchange kinetics with **1-A**

To investigate the possibility of transimination and aldehyde exchange, sequential control experiments were performed in the same NMR tube using the preformed **1-A** imine complex at pD 7.4 in D_2O. First, an equimolar amount (0.5 mM) of 4-methoxyaniline was added, and no changes in the imine signal corresponding to **1-A** were observed over 48 h, indicating that transimination does not occur under these conditions. Subsequently, salicylaldehyde (0.5 mM) was added to the same sample to probe aldehyde exchange. Again, no changes in the imine signals were observed within 24 h, demonstrating that aldehyde exchange is also restricted. These results indicate that the **1-A** imine complex is resistant to both transimination and aldehyde exchange in aqueous media, consistent with the formation of a strongly stabilized, chelated structure.

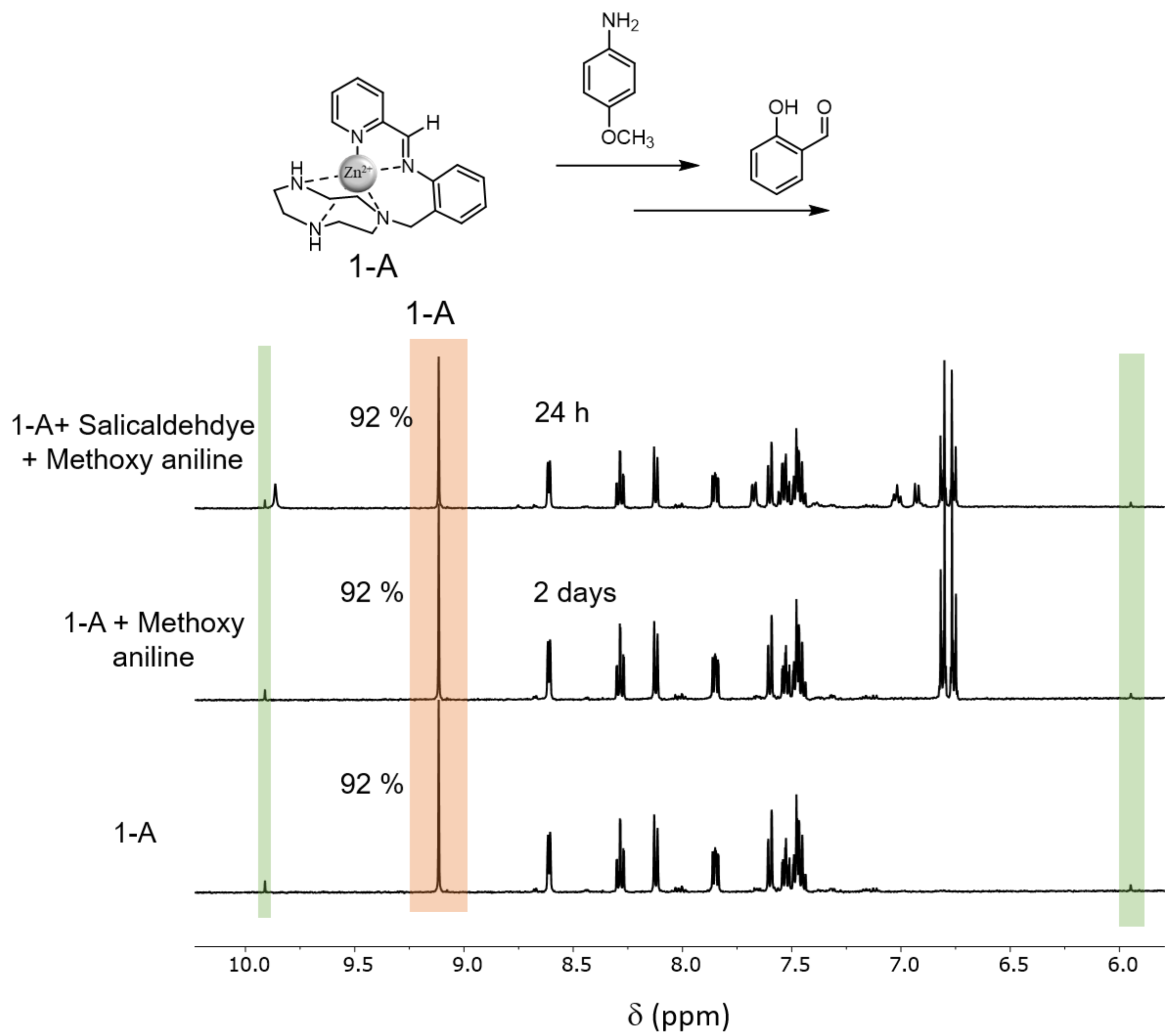

Figure S19. Parts of the ^1H NMR spectra (500 MHz, D_2O) recorded over the indicated time intervals following mixing of the preformed **1-A** imine with 4-methoxyaniline (0.5 mM) in 10 mM HEPES buffer at pD 7.4. The green and orange highlighted regions correspond to aldehyde **1** and imine **1-A**, respectively. After 48 h, salicylaldehyde (0.5 mM) was added to the same sample, and the spectra were further monitored to probe potential aldehyde exchange.

S2.10.4. Equilibration kinetics with a mixture of **1**, **A**, and **T**

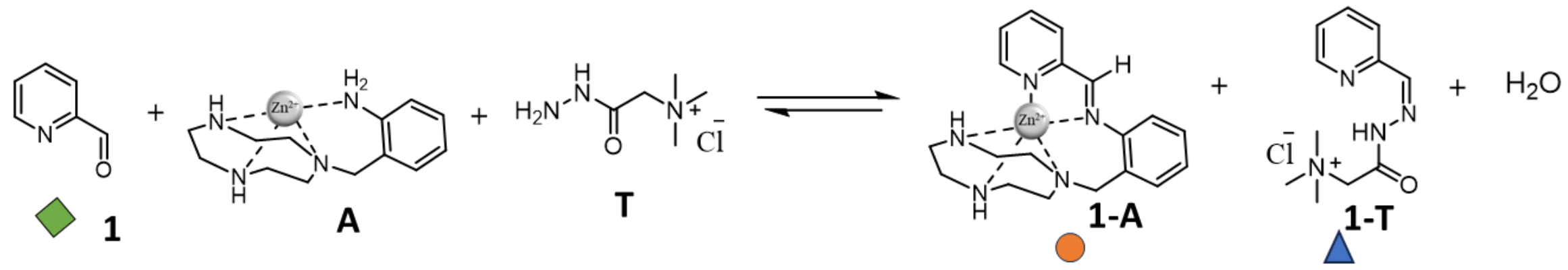

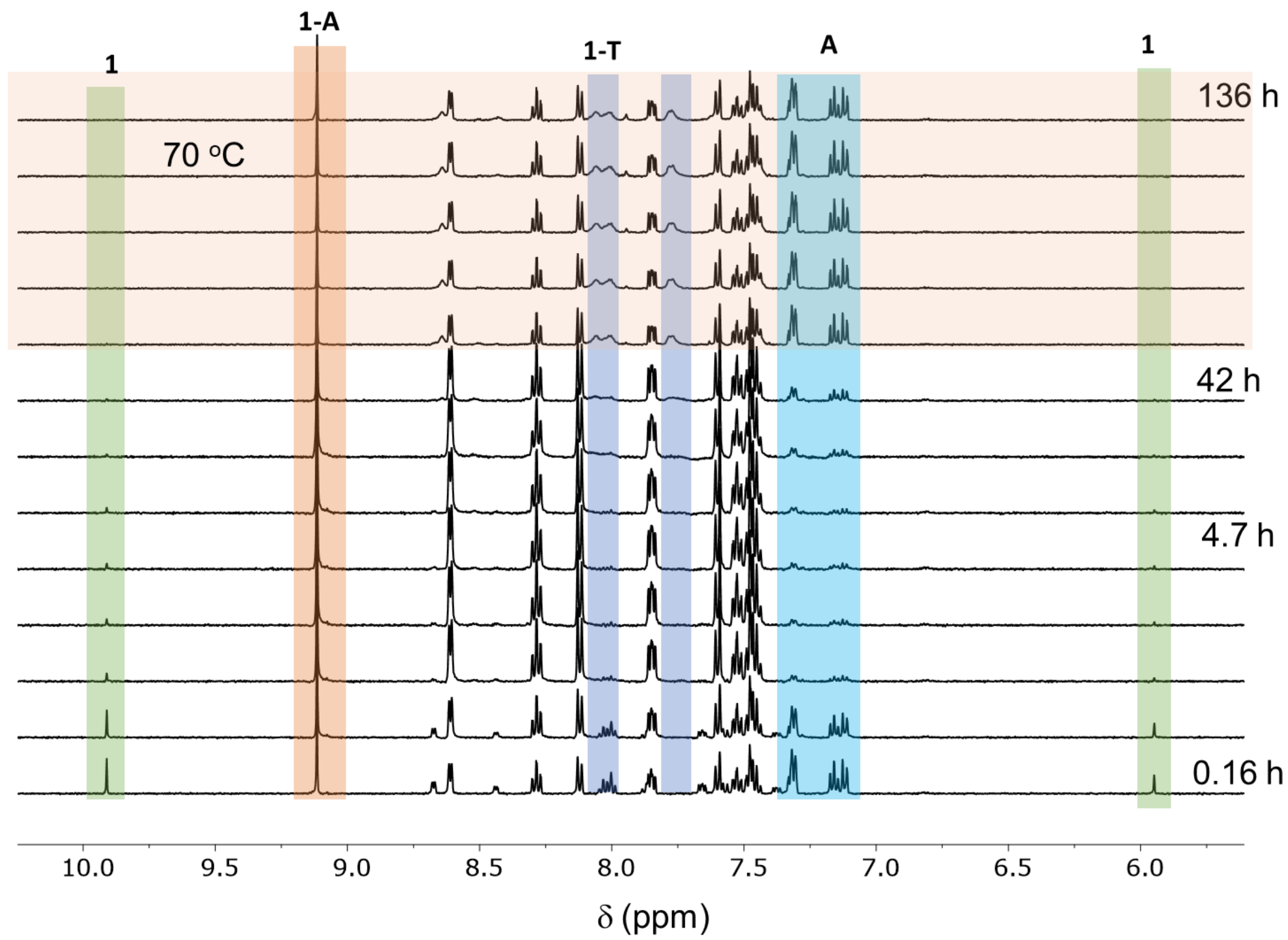

Figure 20. Parts of ^{1}H NMR spectra (500 MHz, D_2O) recorded between the indicated time intervals following mixing of **1, A** and Girard's reagent **T** (0.5 mM each) in 10 mM HEPES buffer at pD 7.4. The green, orange, blue and cyan highlighted regions correspond to aldehyde **1**, imine **1-A**, hydrazone (**1-T**), and free ligand **A**, respectively. The experiment was initially conducted at room temperature for 42 h, followed by heating at 70 °C to promote equilibration, with spectra recorded up to 196 h.

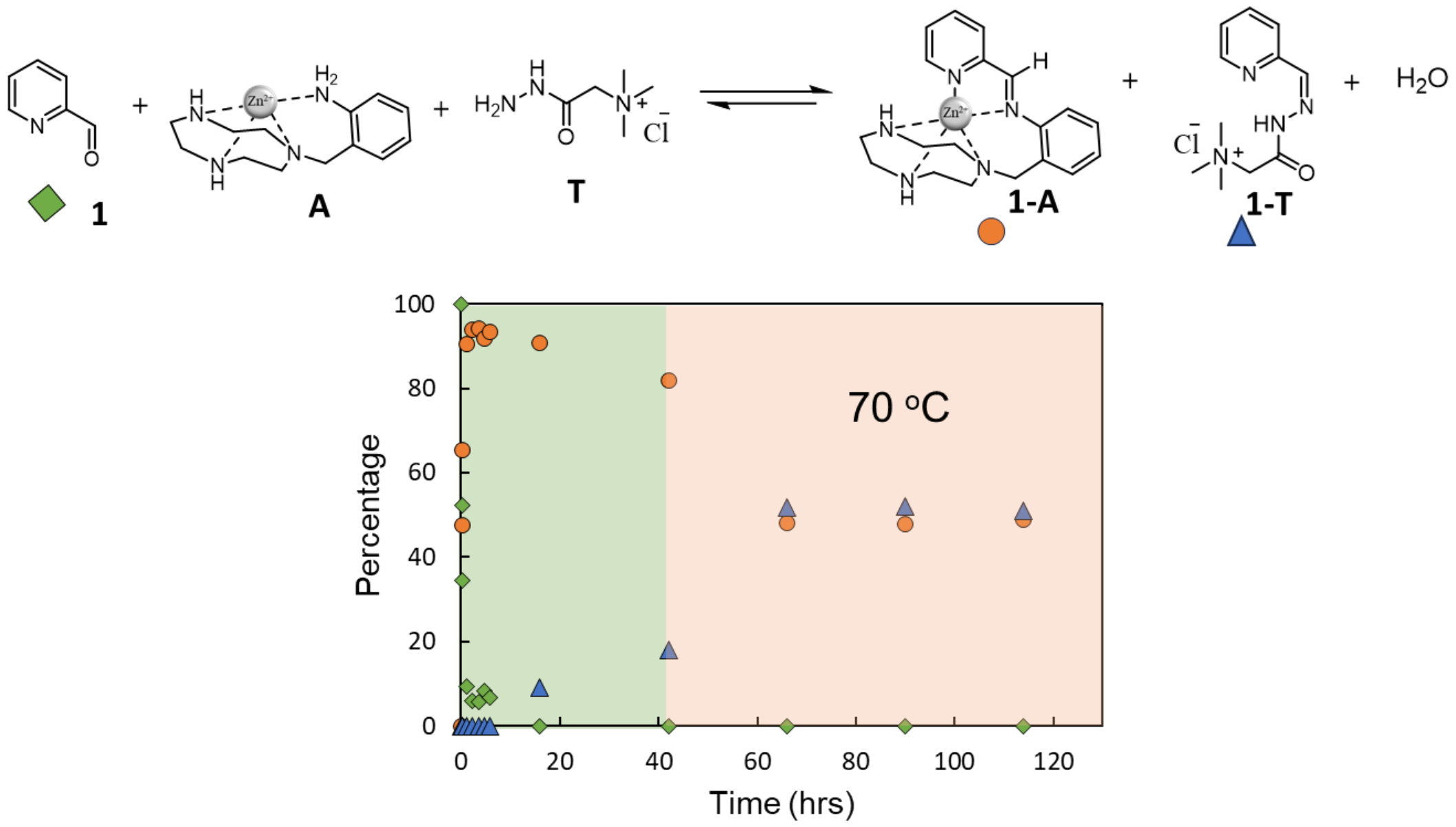

Figure S21. Time-dependent concentrations of hydrazone (**1-T**), imine (**1-A**), and aldehyde **1** following mixing of Girard's reagent **T, A,** and **1** (each 0.5 mM). The green shaded region corresponds to measurements performed at room temperature, while the red shaded region represents data collected at 70 °C. The corresponding ^{1}H NMR spectra are shown in Figure S20.

S2.10.5. Equilibration kinetics between **1-A** and Girard's reagent **T**

To investigate the potential exchange between imine and hydrazone species, the reactivity of the preformed imine **1-A** toward Girard's T reagent (**T**) was examined. Hydrazides are known to exhibit higher nucleophilicity and typically form thermodynamically more stable hydrazones with carbonyl compounds. Accordingly, the preformed imine **1-A** was treated with an equimolar amount of Girard's reagent **T** in aqueous buffer. At room temperature, the exchange process proceeded slowly, indicating a significant kinetic barrier for hydrazone formation from the coordinated imine system. Over time, the system approached an equilibrium distribution consisting of approximately 53:47 of imine (**1-A**) and hydrazone (**1-T**), respectively. The relative composition of the species was determined by quantifying the imine **(1-A)** signal using an internal standard. Due to overlap of the hydrazone (**1-G**) signals with other resonances, its concentration was estimated indirectly by subtracting the imine

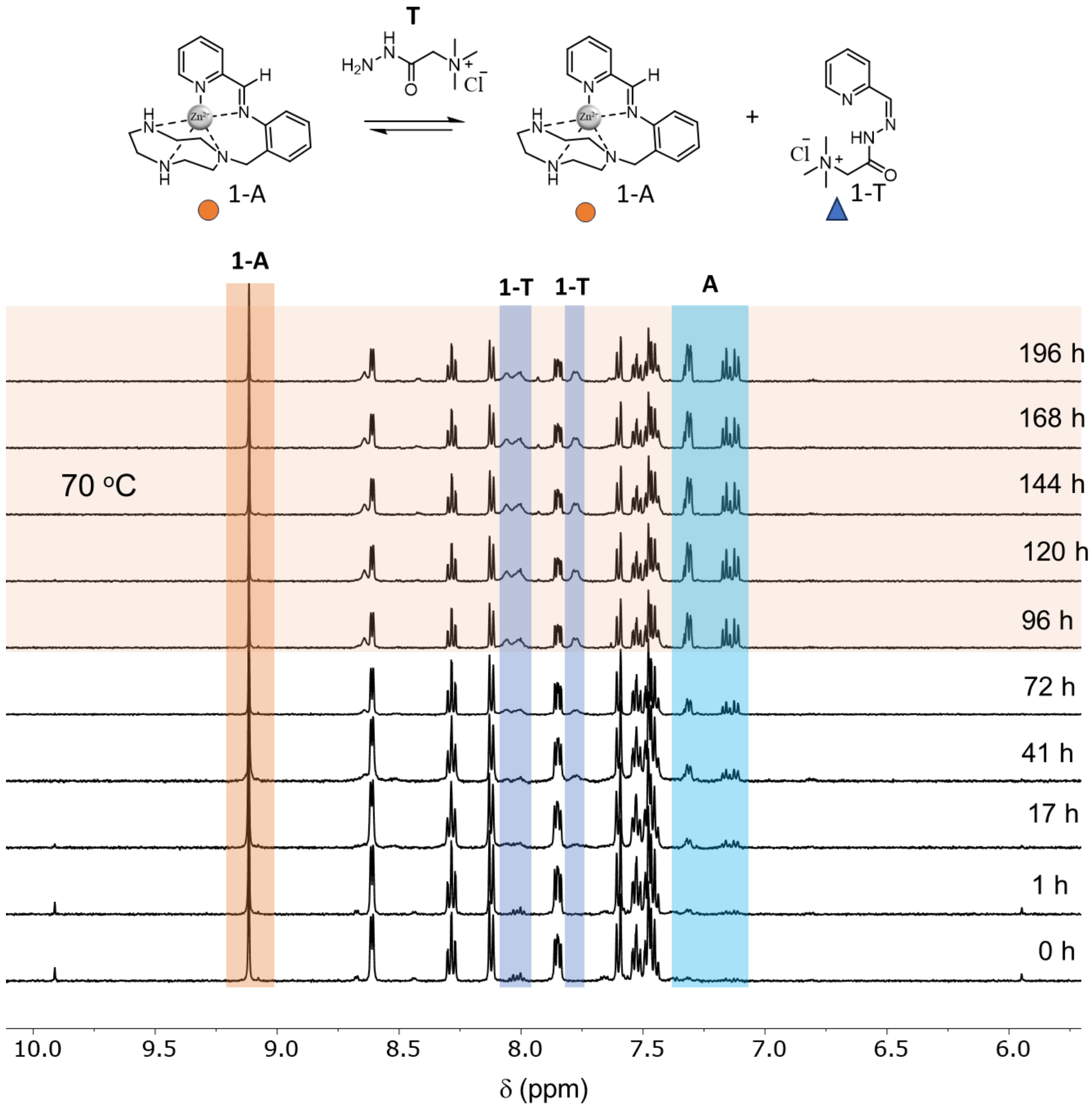

Figure S22. Parts of ^{1}H NMR spectra (500 MHz, D_2O) recorded with the indicated time intervals following mixing of **1-A** (0.5 mM) and Girard's reagent **T** (0.5 mM) in 10 mM HEPES buffer at pD 7.4. The blue, orange, and cyan highlighted regions correspond to hydrazone (**1-T**), imine (**1-A**), and free ligand **A**, respectively. The experiment was initially conducted at room temperature for 72 h, followed by heating at 70 °C to facilitate equilibration, with spectra recorded up to 196 h.

concentration from the initial total concentration (0.5 mM). The absence of aldehyde signals indicates that the decrease in imine concentration corresponds to the formation of hydrazone.

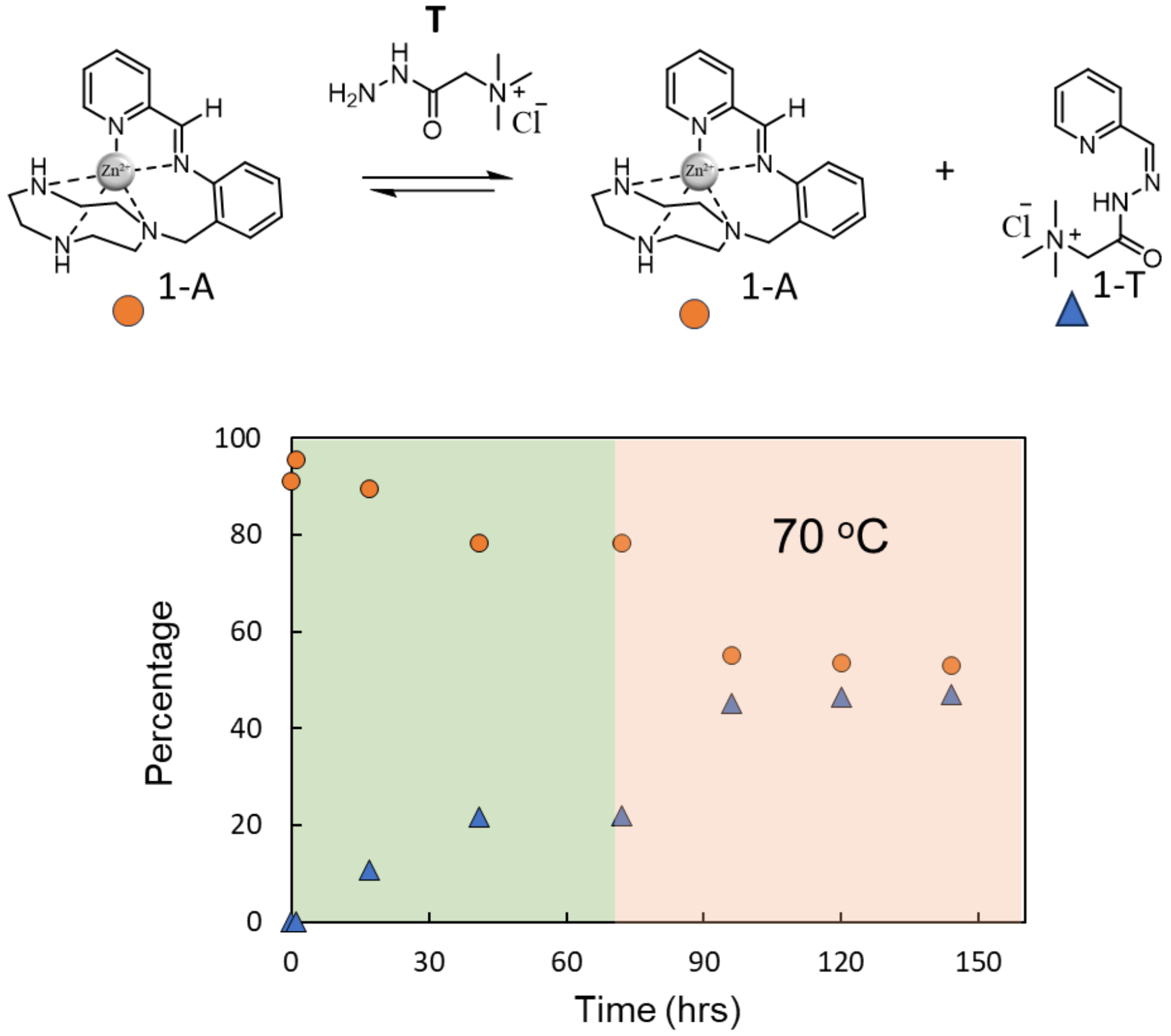

Figure S23. Time-dependent concentrations of hydrazone (**1–T**) and imine (**1–A**) (~0.48 mM) following addition of Girard's reagent **T**. The green shaded region corresponds to measurements performed at room temperature, while the red shaded region represents data collected at 70 °C. The corresponding ^{1}H NMR spectra are shown in Figure S22.

S2.11. Role of pH in the kinetic for the formation of 1-A

Kinetic experiments were performed at different pH using UV-vis spectroscopy to monitor **1-A** formation in aqueous conditions. The reactions were investigated at pD 5.9, 7.4, 7.9, 8.4 and 10.4 using 10 mM buffer solutions prepared in D_2O. To validate the kinetic traces, ^{1}H NMR measurements were conducted at the end of each experiment to quantify the imine concentration. These values were subsequently used to determine the molar extinction coefficient (ε) for each pH.

Calculation of the Molar Extinction Coefficient (ε) at pD 7.4

Absorbance at saturation (A) = 1.948

Conversion to imine determined by ^{1}H NMR = 92 %

Initial aldehyde concentration = 0.5 mM

Concentration of imine formed:

$$C_{\text{imine}} = 0.5 \times \frac{92}{100} = 0.460 \text{ mM}$$

$$\varepsilon = \frac{A}{Cl} = \frac{1.948}{0.460 \times 1} = 4.23 \text{ mM}^{-1} \text{ cm}^{-1}$$

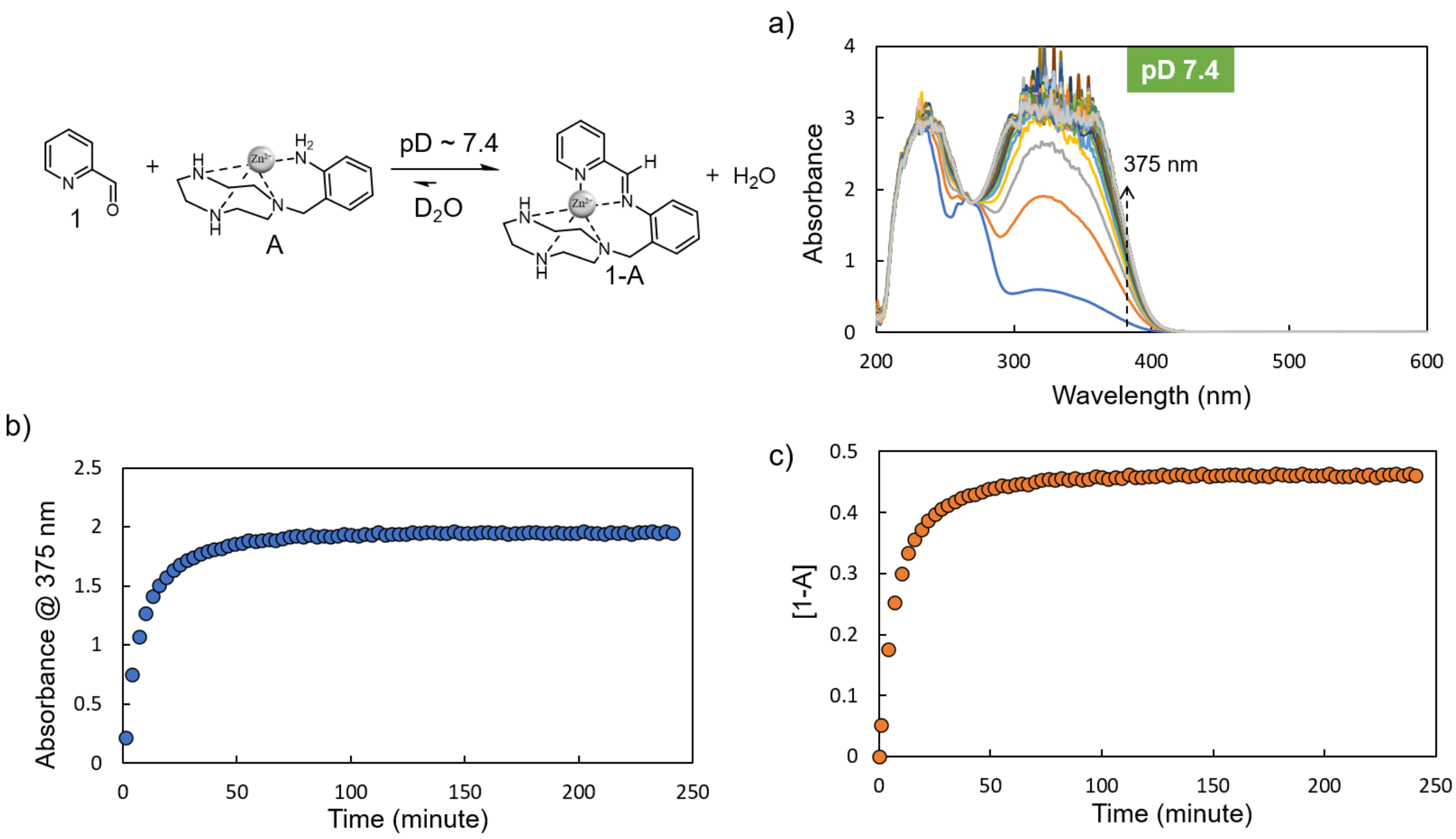

Figure S24. Kinetic analysis of **1-A** formation at pD 7.4 in 10 mM HEPES buffer (D_2O) monitored by UV-vis spectroscopy over 4 h. a) Time-dependent spectral changes showing the progression of imine formation. b) Absorbance at 375 nm as a function of time, corresponding to the formation of **1-A**. c) Concentration of **1-A** versus time, obtained from UV-vis data using molar extinction coefficients determined from ^{1}H NMR (Figure S27). The data were fitted to a second-order reversible kinetic model.

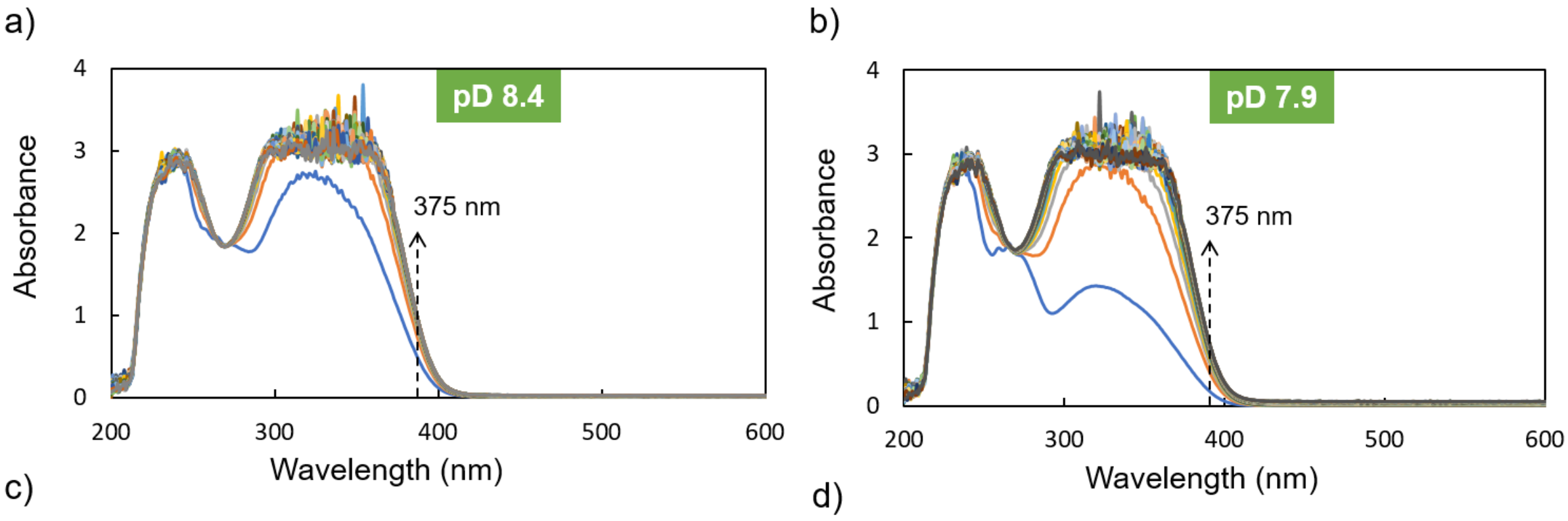

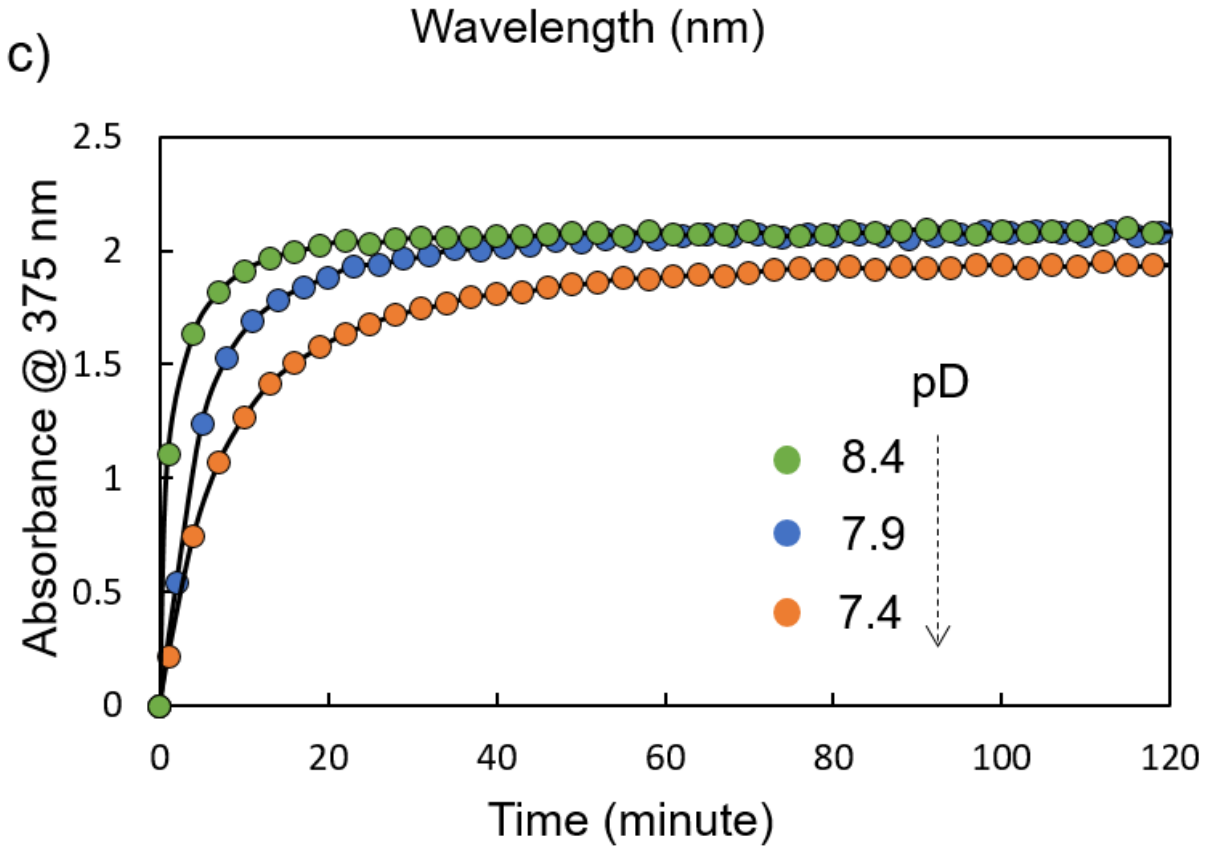

d)

pD	ε (molar extinction coefficient, $mM^{-1}cm^{-1}$)
7.4	4.23
7.9	4.33
8.4	4.32

Figure S25. Kinetic analysis of **1-A** imine formation at pD 7.9 and 8.4 in 10 mM HEPES buffer, monitored by UV-vis spectroscopy over 4 h. a, b) Time-dependent UV-vis spectra changes showing the progression of imine formation at pD 8.4 and pD 7.9. c) Comparison of absorbance at 375 nm as a function of time, corresponding to the formation of **1-A** under different pH conditions. (d) Summary of molar extinction coefficients (ε) at different pH values, determined from ^{1}H NMR (Figures S27, S28 and S29). The kinetic data were fitted to a second-order reversible model

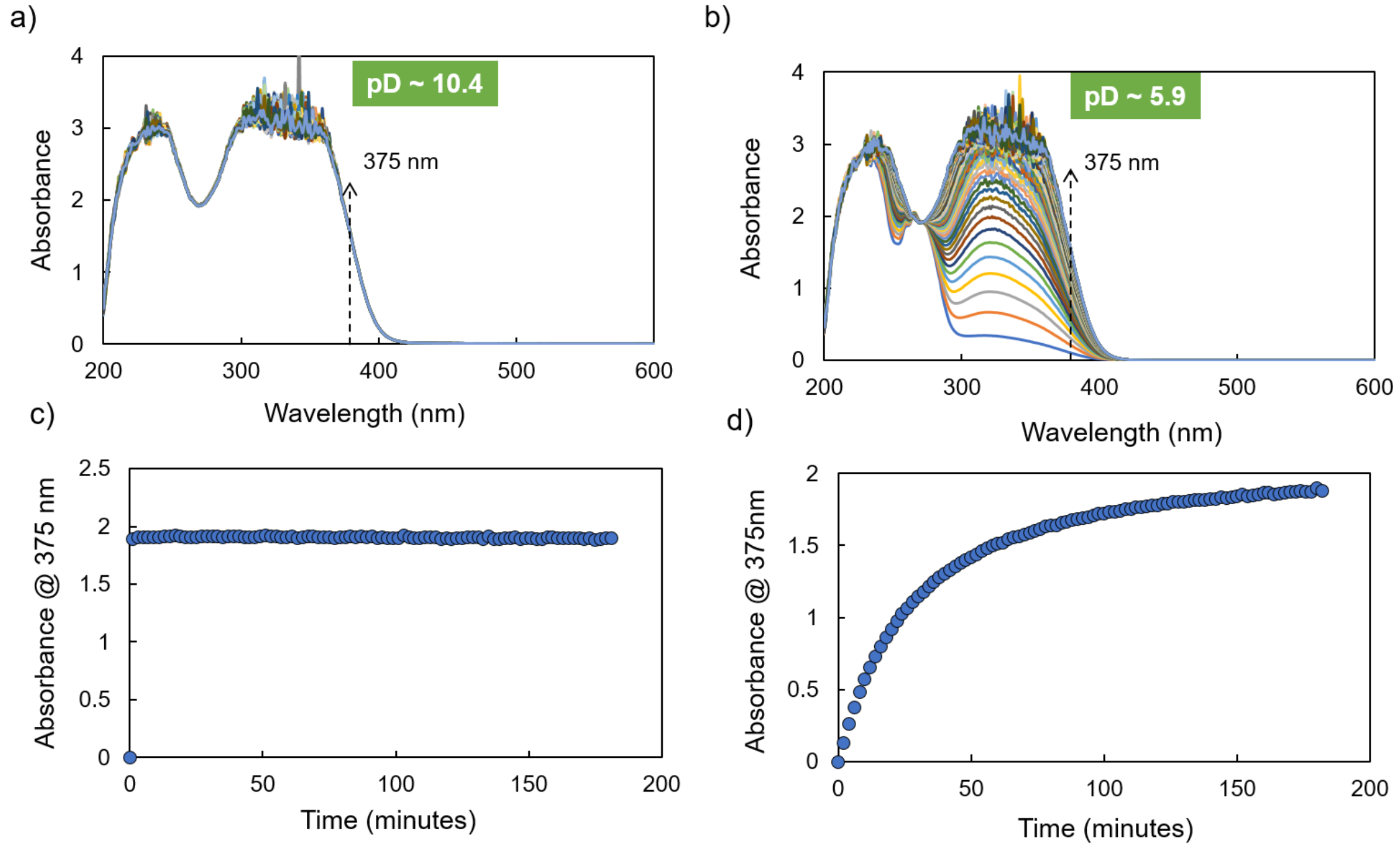

Figure S26. Kinetic analysis of **1-A** imine formation at pD 10.4 and 5.9 in 10 mM CAPS and MES buffer respectively, monitored by UV-vis spectroscopy over 3h. a, b) Time-dependent UV-vis spectra changes showing the progression of imine formation at pD 10.4 and pD 7.9. c,) Absorbance at 375 nm as a function of time, corresponding to the formation of **1-A** at pD 10.4. d,) Absorbance at 375 nm as a function of time, corresponding to the formation of **1-A** at pD 5.9. Molar extinction coefficients (ε) at determined from ^{1}H NMR, 4.16 $mM^{-1}cm^{-1}$ at pD 5.9 and 4.22 $mM^{-1}cm^{-1}$ at pD 10.4 (Figures S30 and 31). The kinetic data were fitted to a second-order reversible model.

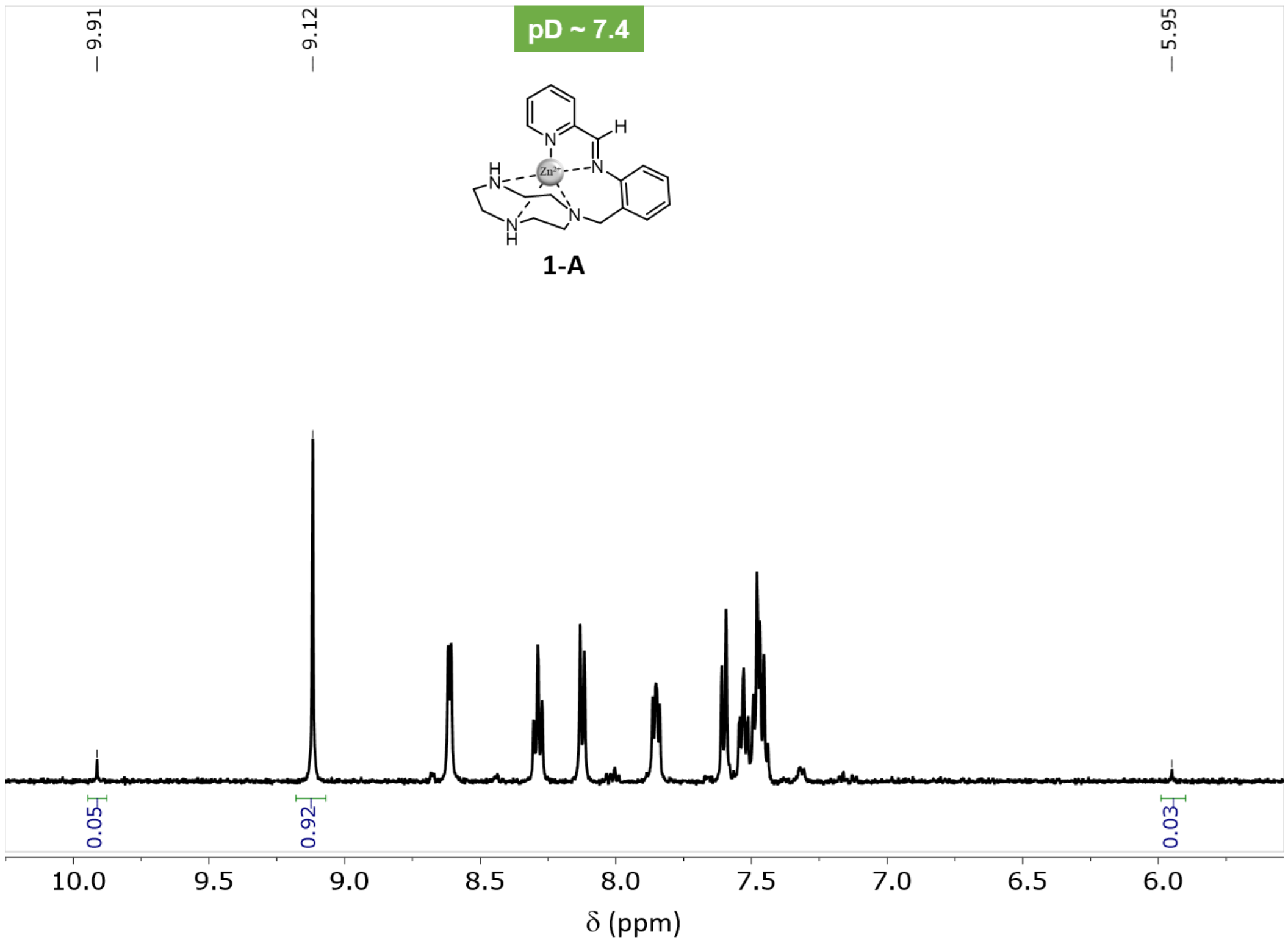

Figure S27. Part of ^1H NMR spectra (500 MHz, pD = 7.4, 10 mM HEPES in D_2O) recorded after the UV-visible kinetic experiment (pD 7.4).

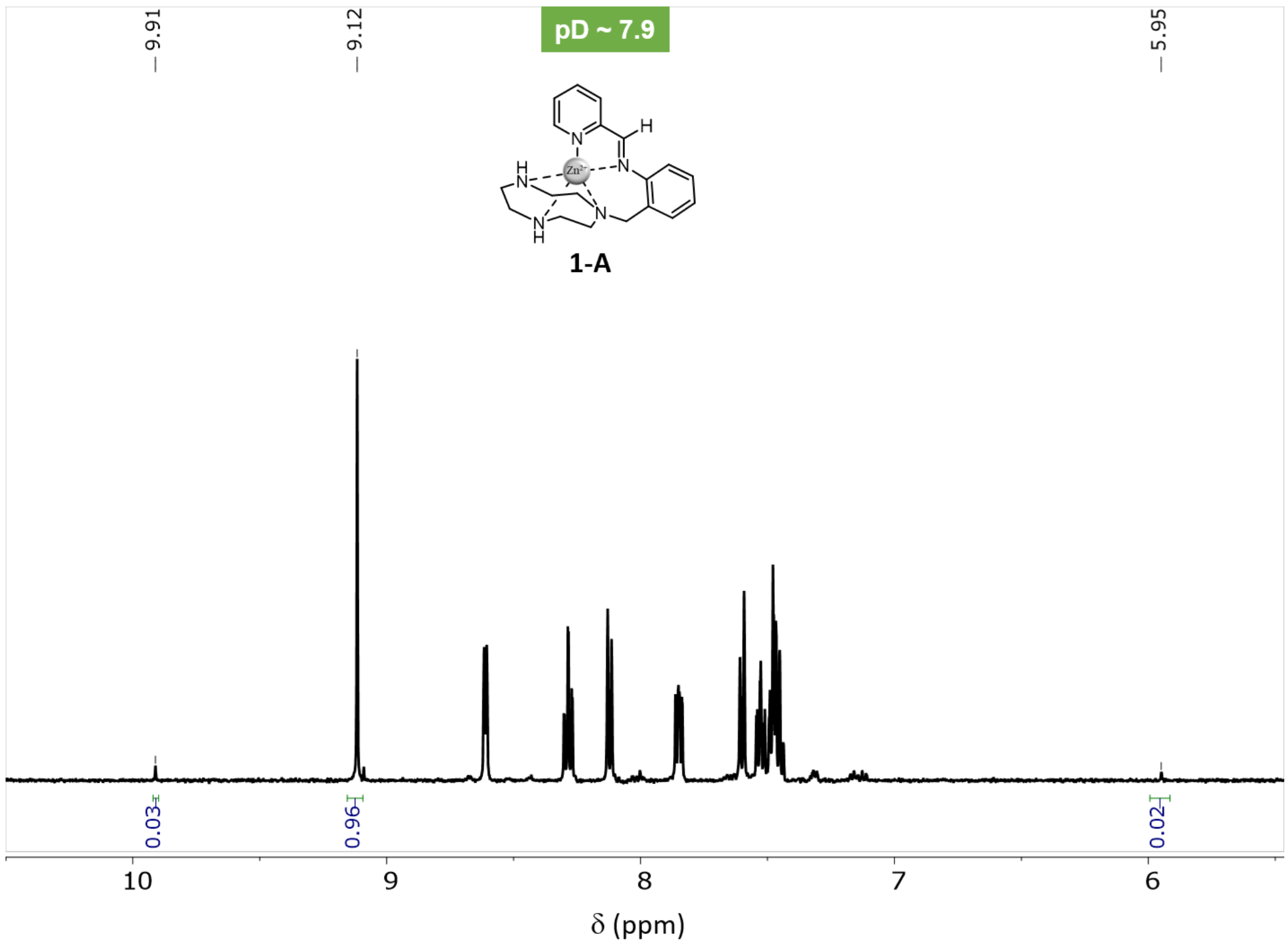

Figure S28. Part of ^1H NMR spectra (500 MHz, pD = 7.9, 10 mM HEPES in D_2O) recorded after the UV-visible kinetic experiment (pD 7.9).

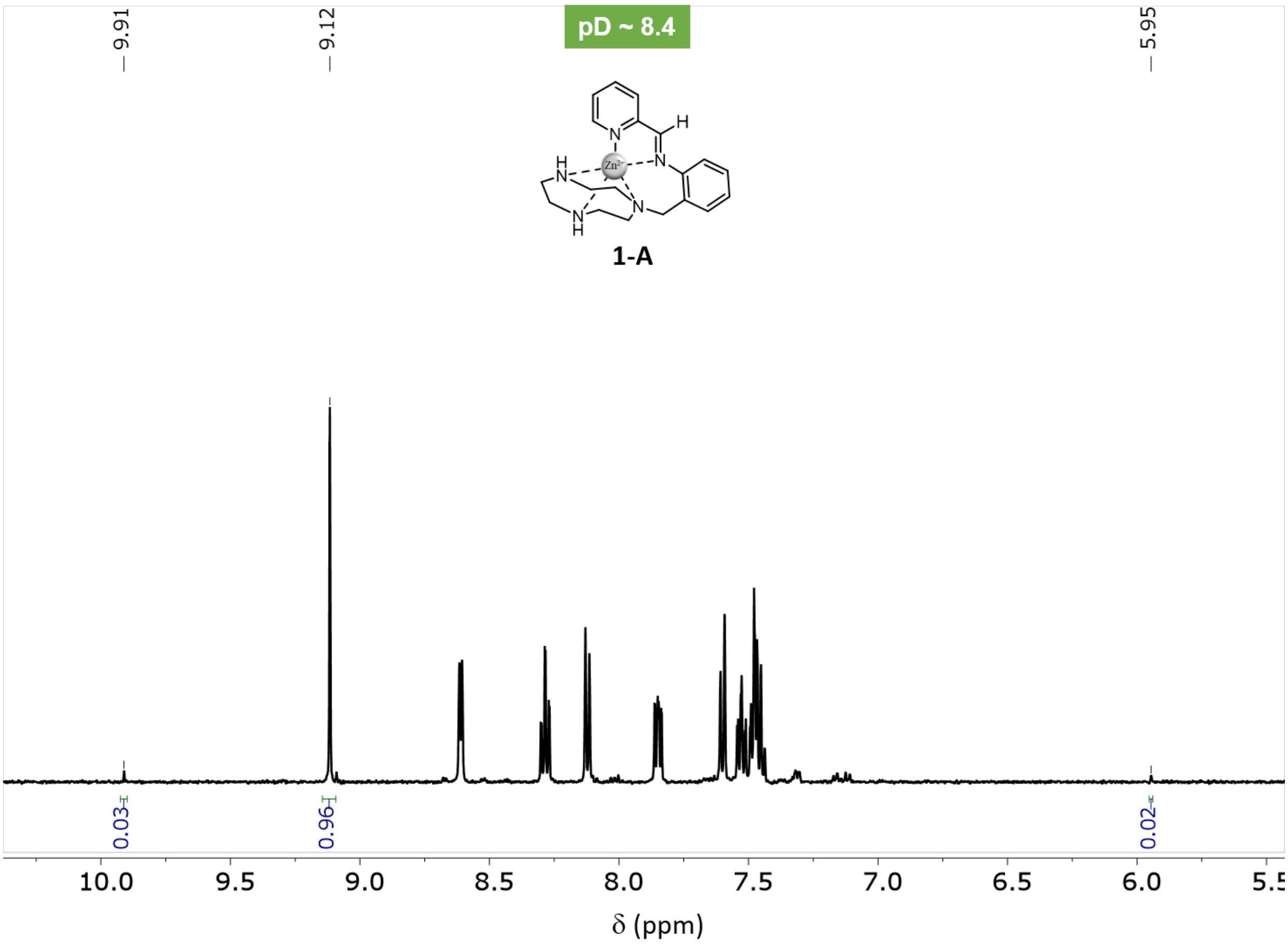

Figure S29. Part of ^{1}H NMR spectra (500 MHz, pD = 8.4, 10 mM HEPES in D_2O) recorded after the UV-Visible kinetic experiment (pD 8.4).

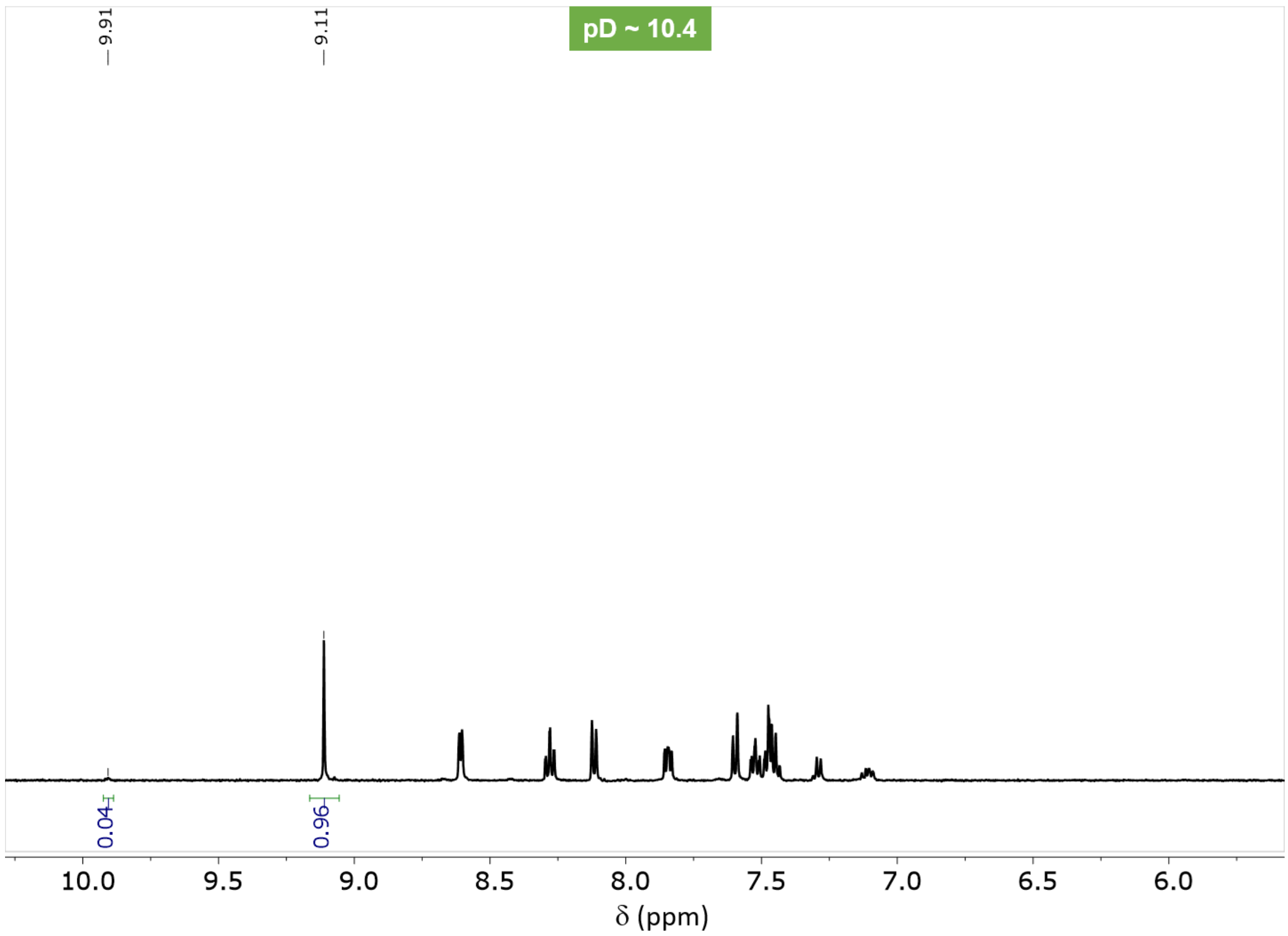

Figure S30. Part of ^{1}H NMR spectra (500 MHz, pD = 10.4, 10 mM CAPS buffered in D_2O) recorded after the UV-Visible kinetic experiment (pD 10.4).

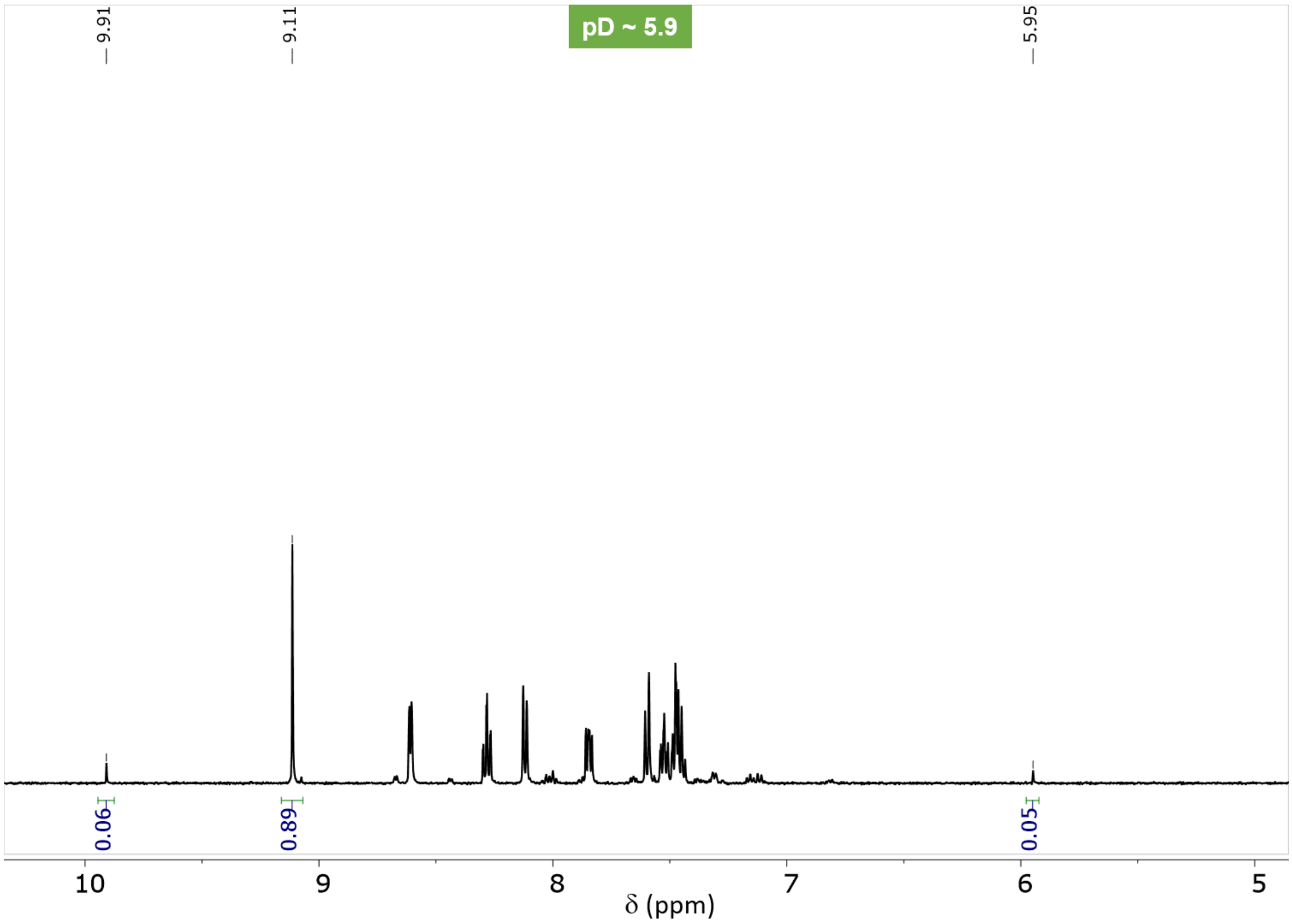

Figure S31. Part of ^1H NMR spectra (500 MHz, pD = 5.9, 10 mM MES buffered in D_2O) recorded after the UV-Visible kinetic experiment (pD 5.9).

S2.12. Formation of imine 1-A at lower concentrations

To evaluate the efficiency of imine (**1-A**) formation at reduced concentrations, kinetic experiments were conducted using UV-vis spectroscopy with different concentrations up to 50 µM. At such low concentrations, direct quantification of imine formation by ^{1}H NMR becomes unreliable due to limited signal to noise ratio. Therefore, the molar extinction coefficient (ε), determined at higher concentrations under identical experimental conditions, was used to estimate the imine concentration from the UV-vis absorption data. This approach assumes that ε remains effectively constant upon dilution, as the reaction medium, pH, and overall conditions are unchanged. The initial concentrations of the precursors were adjusted proportionally to maintain consistent stoichiometry across all experiments. While this method enables reliable estimation of imine formation at low concentrations, a minor uncertainty ~ 2-3 % is expected due to signal limitations.

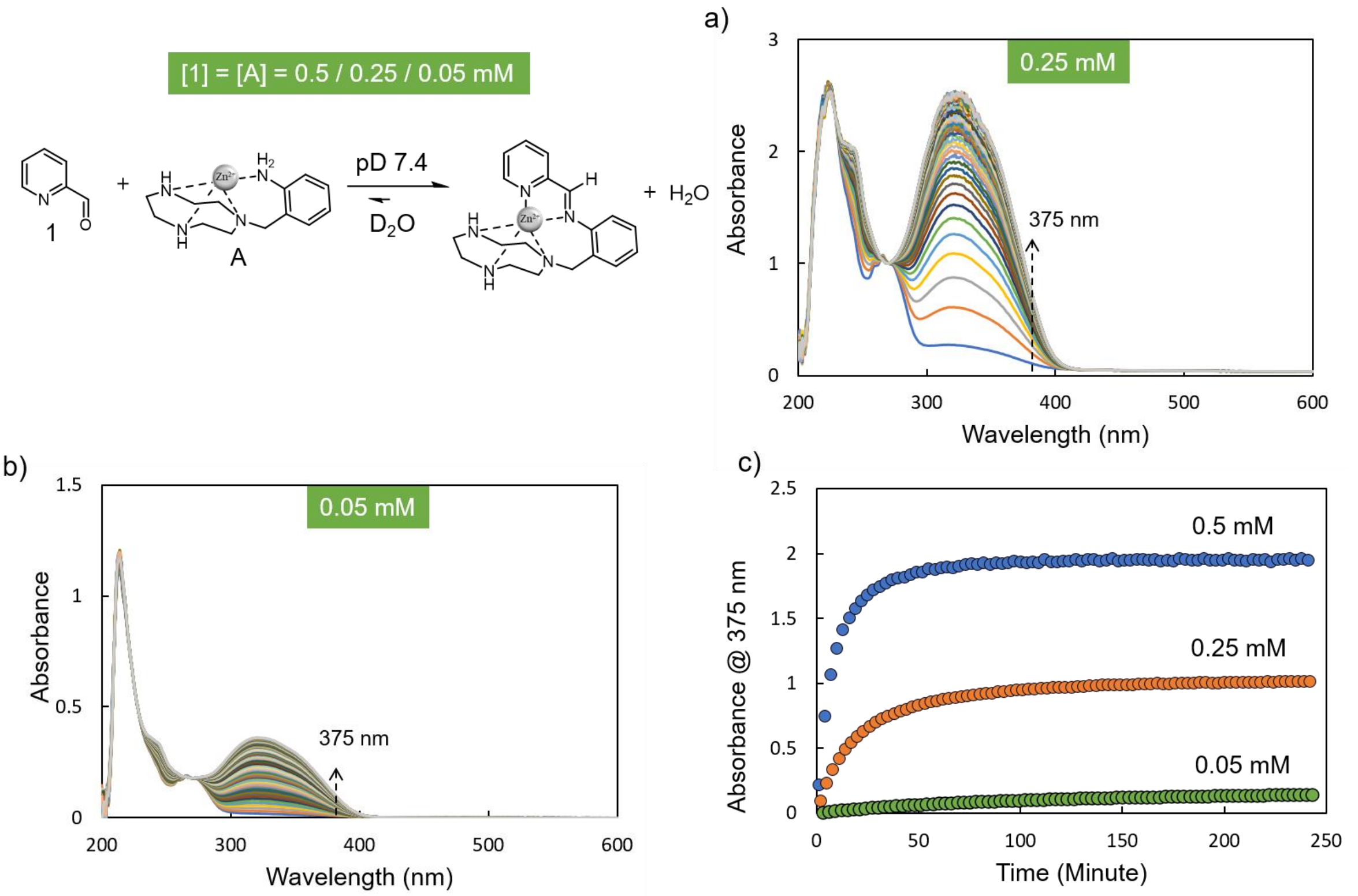

Figure S32. Kinetic analysis of **1-A** imine formation at varying concentrations in 10 mM HEPES buffer (D_2O) at pD 7.4, monitored by UV-vis spectroscopy over 4 h. a, b) Time-dependent UV-vis spectra showing the progression of imine formation at 0.25 mM and 50 µM. c) Comparison of absorbance at 375 nm as a function of time for different initial concentrations (0.5 mM, 0.25 mM, and 50 µM), corresponding to the formation of **1-A**. The kinetic data were fitted to a second-order reversible mode.

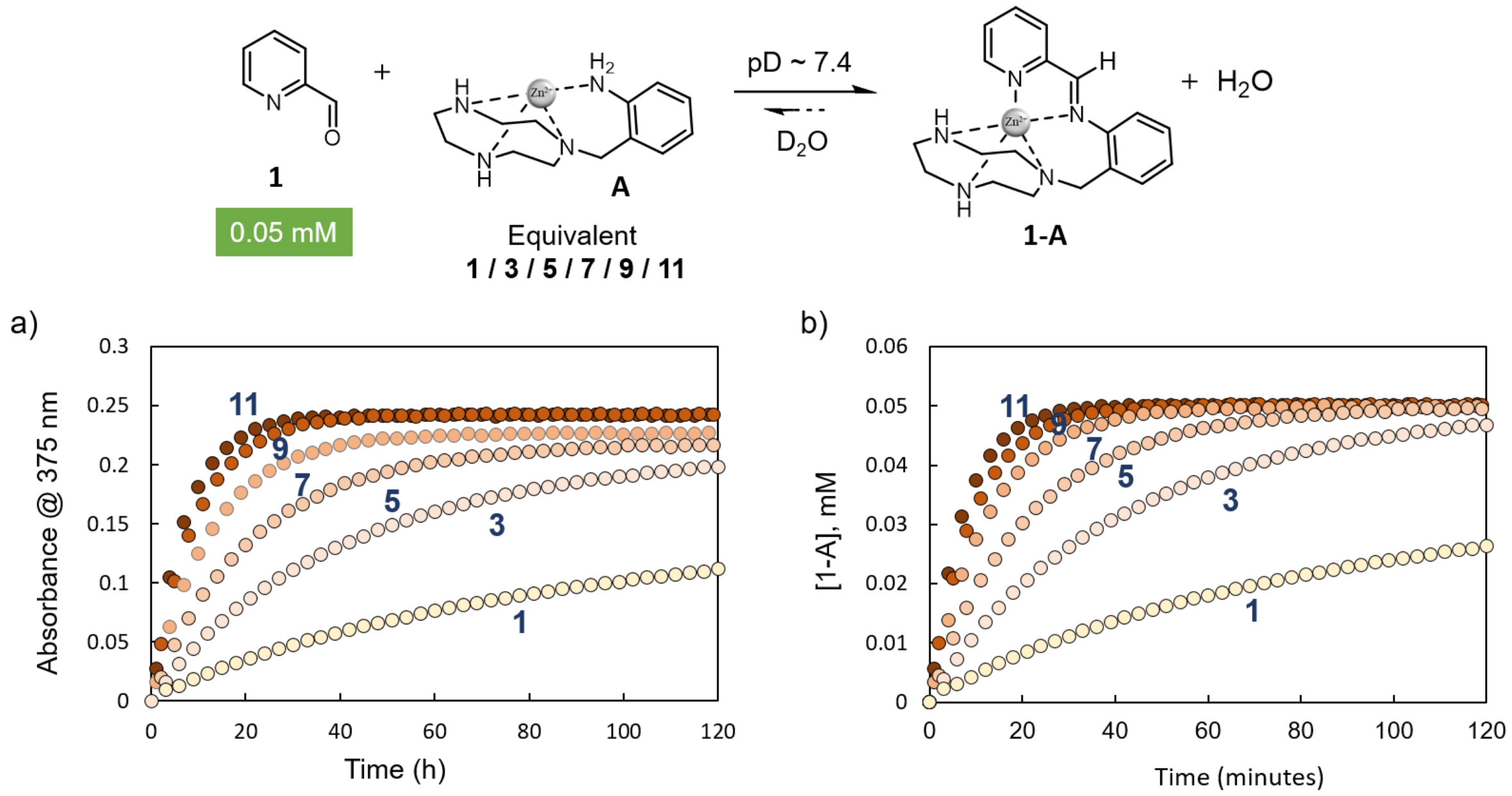

Figure S33. Kinetic analysis of **1-A** imine formation at low concentration (50 µM) with increasing equivalents of **A** (1-11 equiv), monitored by UV-vis spectroscopy at pD 7.4 in 10 mM HEPES buffer. a) Time-dependent absorbance at 375 nm corresponding to imine formation. b) Concentration profiles of **1-A** as a function of time at different equivalents of **A**. Although the contribution of excess **A** to the absorbance at 375 nm is negligible under these conditions, at low concentrations (50 µM) minor interference may still lead to an estimated uncertainty of ~ 5 % in the calculated imine concentration.

S2.13. Formation of imine 1-A in a complex biochemical matrix

To evaluate the selectivity of imine (**1-A**) formation under biologically relevant conditions, experiments were conducted in *E. coli* cell lysate. An equimolar mixture of precursors **1** and **A** was employed to access the imine formation in the presence of a complex mixture of biomolecules. The cell lysate was prepared by mechanical lysis of *E. coli* cells, followed by centrifugation to remove large macromolecules to reduce sample viscosity. Despite this treatment, the lysate contains a wide range of biomolecules, including amino acids, proteins, and lipids, which present competing nucleophilic functionalities that could potentially react with aldehyde **1**.

Experimental Procedure. Kinetic experiments were monitored by ^{1}H NMR spectroscopy in aqueous media. Due to instability of the cell lysate in pure D_2O (likely arising from disruption of H-bonding networks and subsequent aggregation), a mixed solvent system (H_2O/D_2O, 9:1 v/v) was employed. The lysate solution was first prepared in this solvent mixture and allowed to stand for 24 h, followed by an additional centrifugation step to remove aggregated macromolecular components and minimize NMR line broadening. For the kinetic measurements, a final mixture containing 25% (v/v) cell lysate and 75% (v/v) H_2O/D_2O (9:1) was used. The pH of the final solution without buffer was adjusted to 7.0. This approach ensured sufficient spectral resolution while maintaining a representative biological environment for assessing the selectivity of imine formation.

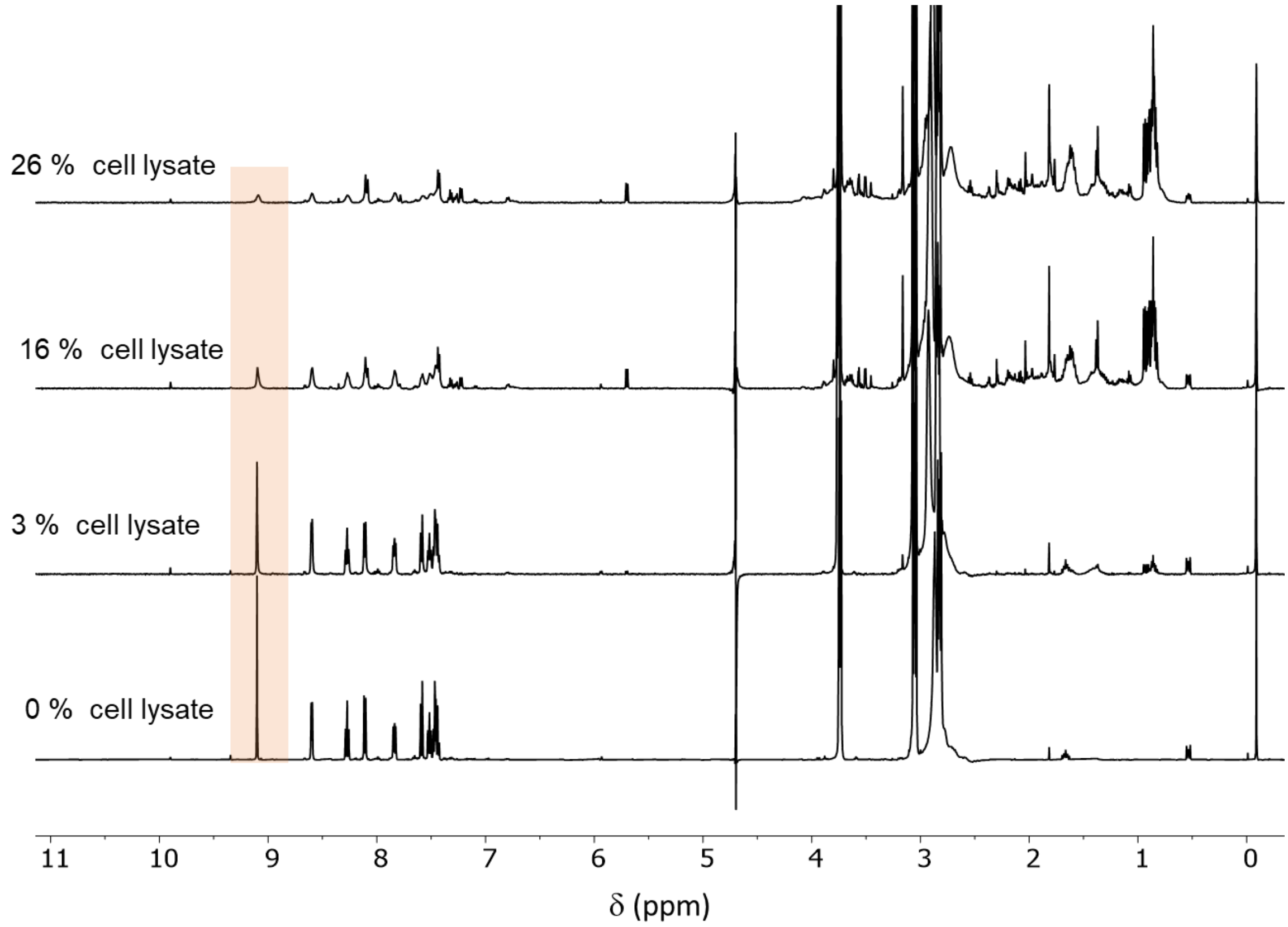

Figure S34. Parts of ^{1}H NMR spectra (500 MHz) recorded in D_2O: H_2O (9:1) with increasing proportions of *E. coli* cell lysate. The orange highlighted region corresponds to the imine **1-A**, (2.5 mM) signal) [**1-A**] = 2.5 mM, which exhibits progressive line broadening with increasing lysate content, reflecting the influence of the complex biological environment on spectral resolution.

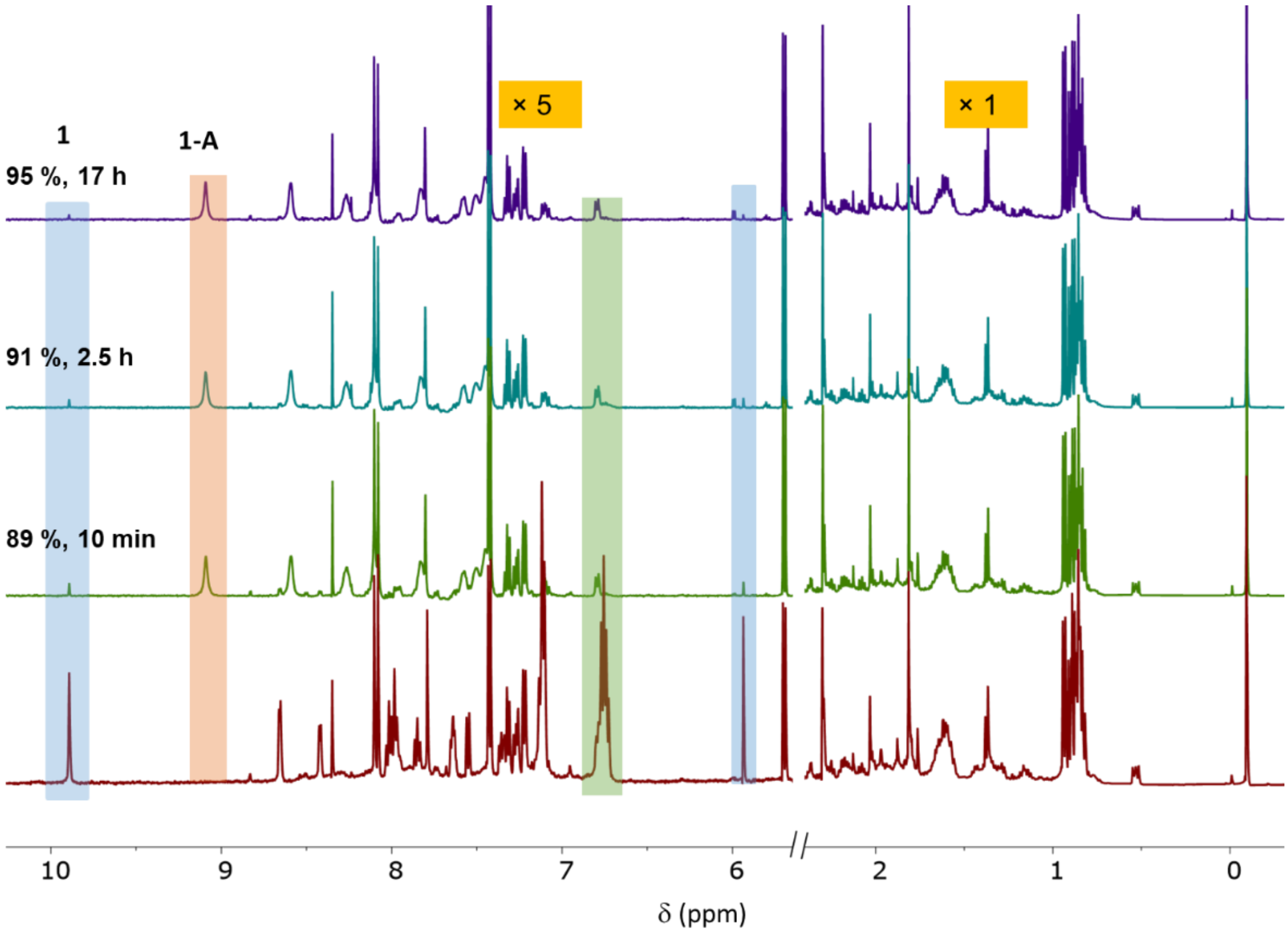

Figure S35. Parts of the ^{1}H spectra (500 MHz, D_2O) recorded with the indicated time intervals after mixing **1** and **A** (25 °C, [C] = 1.0 mM) in cell lysate in a 9:1 H_2O: D_2O-solution. The blue rectangles highlight the aldehyde and hydrated aldehyde peaks of **1**, the green rectangle highlights the peak corresponds to **A**, and the orange rectangle highlight the imine peak of **1-A**. Percentage conversion is determined using the combined integration of aldehyde, hydrated aldehyde and imine to 100 %. The left aromatic part is 5 times zoomed in comparison to aliphatic region.

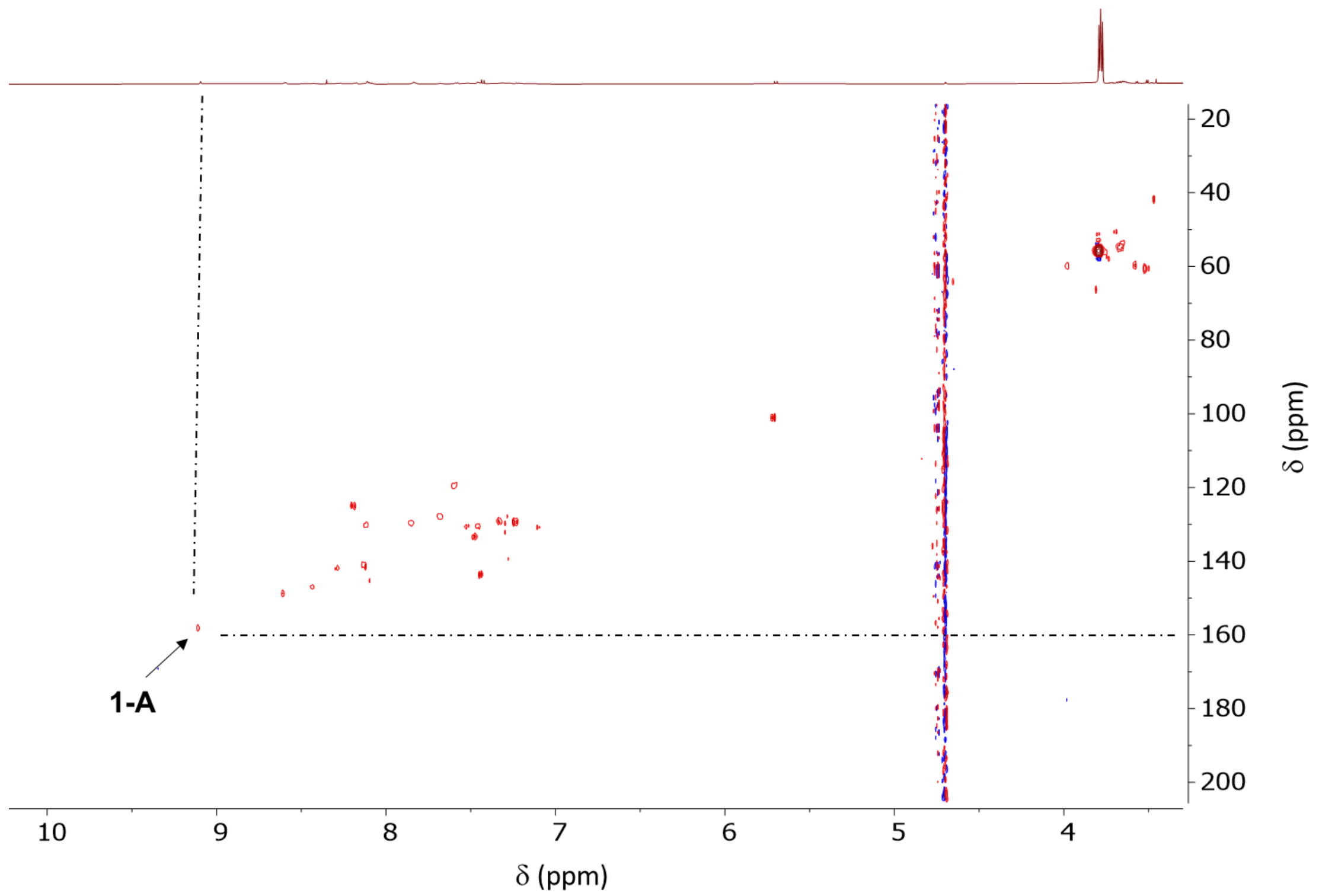

Figure S36. ^{1}H-^{13}C HSQC of **1-A** (2.5 mM) synthesized in the cell lysate in a 9:1 H_2O: D_2O mixture

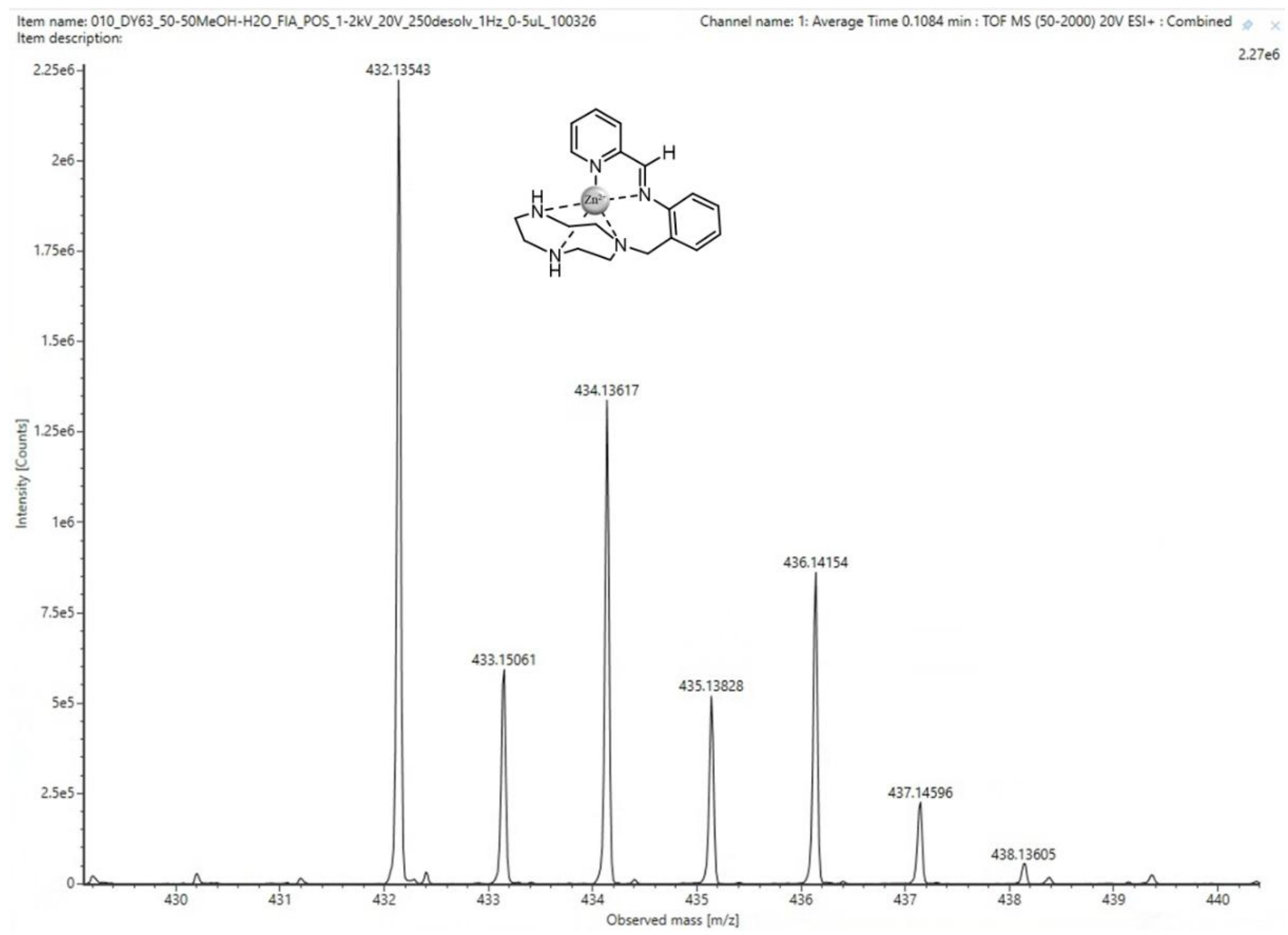

Figure S37. HRMS spectrum of **1-A** synthesized in the cell lysate matrix. Calculated mass, $[M+HCOO]^+$ = 432.1372

S3. Reversible imine bond formation in water using aldehyde 6

S3.1. Reaction kinetics for the formation of 6-B

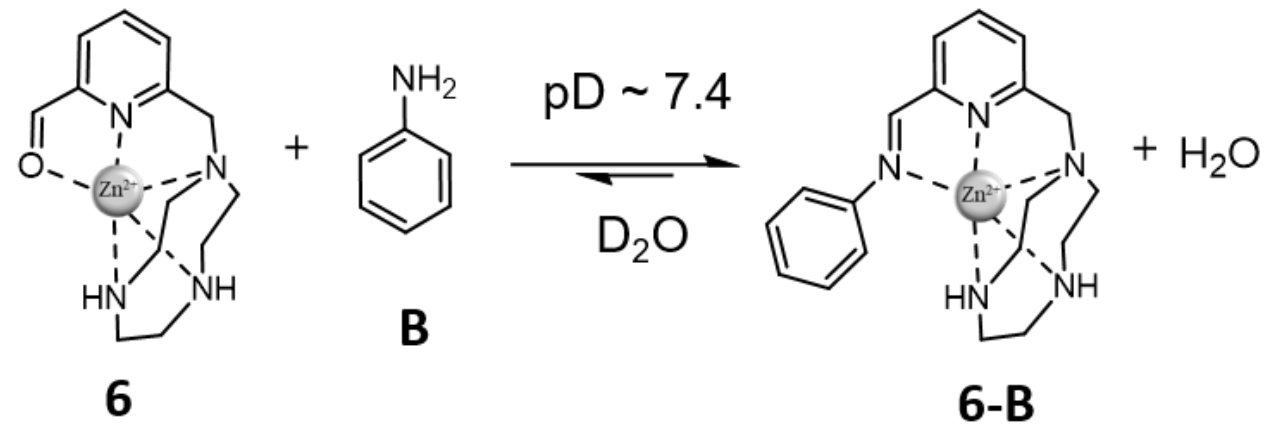

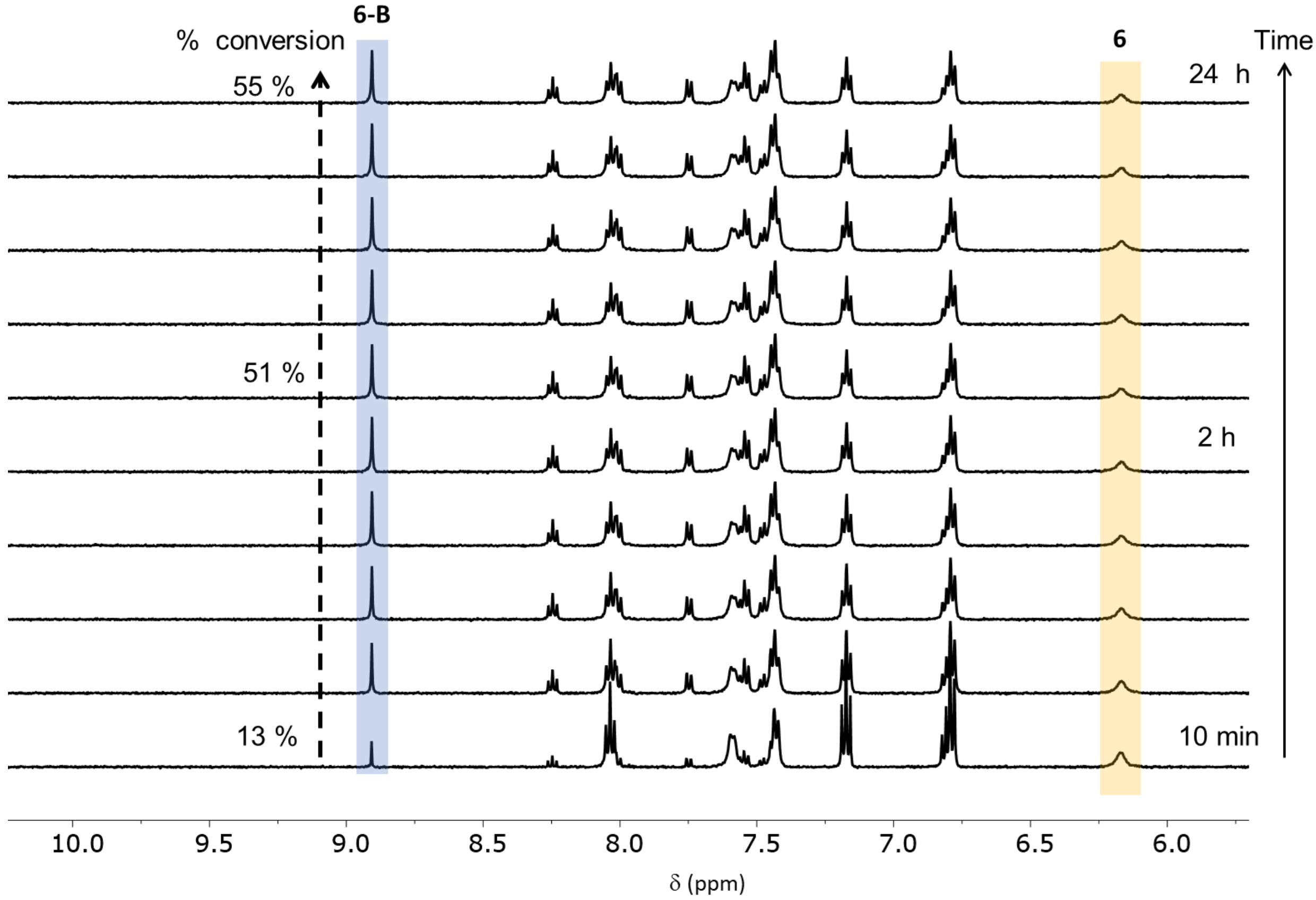

Figure S38. Parts of ^{1}H NMR spectra (500 MHz, D_2O) recorded between the indicated time intervals following mixing of **6** and **B** at 25 °C (pD = 7.4) in 10 mM HEPES buffer. The blue highlighted region corresponds to the imine **6-B**, while yellow regions indicate the hydrated aldehyde **6**.

Calculation of the Molar Extinction Coefficient (ε) for 6-B at 375 nm

Absorbance at saturation (A) = 0.460

Conversion to imine determined by ^{1}H NMR = 55 %

Initial aldehyde concentration = 0.5 mM

Concentration of imine formed:

$$C_{\text{imine}} = 0.5 \times \frac{55}{100} = 0.275 \text{ mM}$$

$$\varepsilon = \frac{A}{Cl} = \frac{0.460}{0.275 \times 1} = 1.67 \text{ mM}^{-1} \text{ cm}^{-1}$$

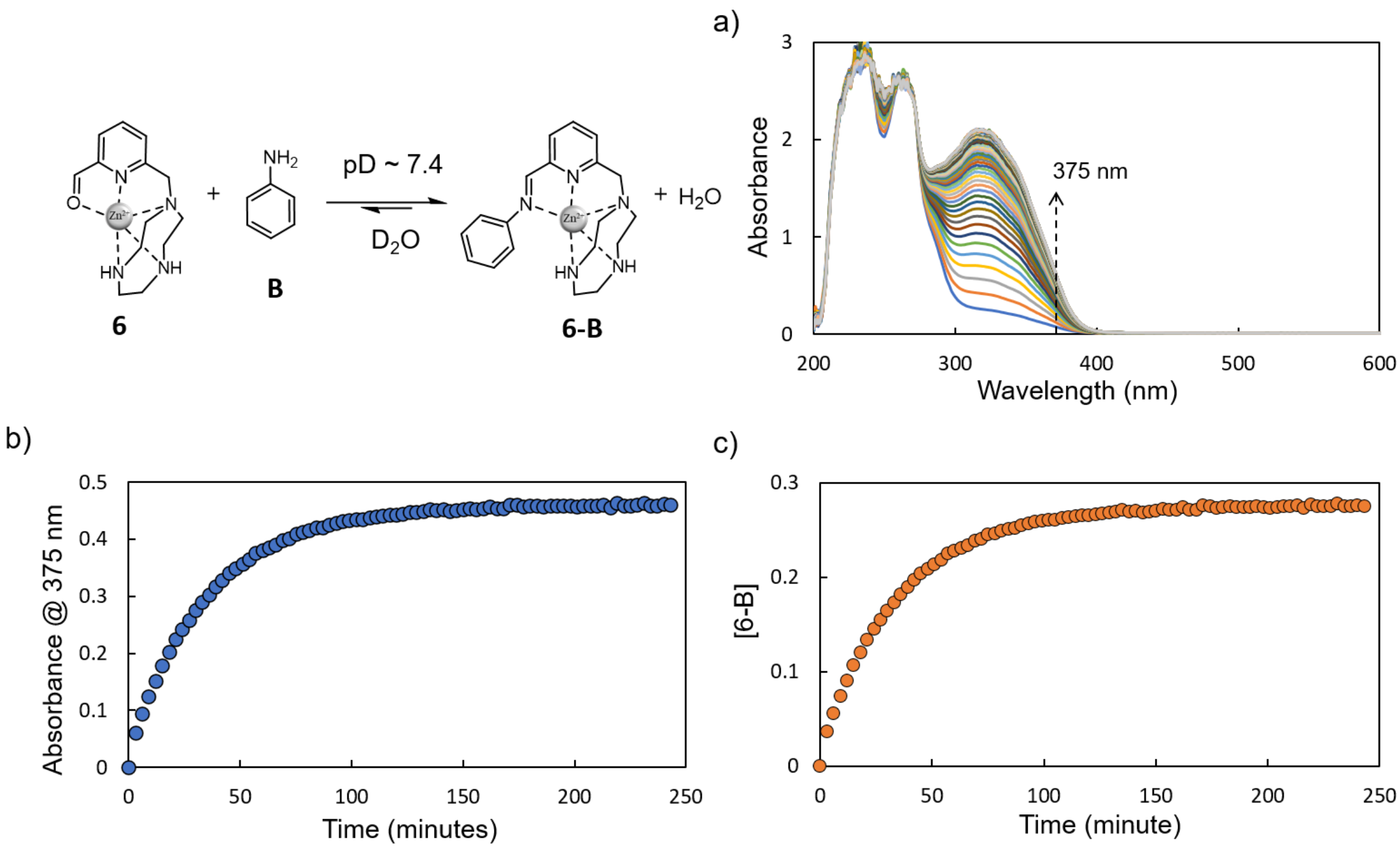

Figure S39. Kinetic analysis of imine **6-B** formation at pD 7.4 in 10 mM HEPES buffer (D_2O) monitored by UV-vis spectroscopy over 4 h. a) Time-dependent UV-vis spectra showing the progression of imine formation. b) Absorbance at 375 nm as a function of time, corresponding to the formation of **6-B**. c) Concentration of **6-B** versus time, obtained from UV-vis data using molar extinction coefficients determined from ^{1}H NMR quantification (Figure S38). The data were fitted to a second-order reversible kinetic model.

S3.2. Thermodynamic control in the formation of 6-B

To verify that the formation of **6-B** is under thermodynamic control, the experiment was conducted under conditions favoring imine formation. Aldehyde **6** and amine **B** were mixed in equimolar amounts in a reduced solvent volume (50 µL) to increase the effective concentration of **6-B**, and the mixture was allowed to equilibrate at room temperature for 24 h. Prior to NMR measurement, the sample was diluted to a final concentration of 0.5 mM by addition of the remaining solvent, while maintaining pD 7.4 using 10 mM HEPES buffer. The first spectrum was recorded approximately 10 min after dilution. Over time, the ratio of imine (**6-B**) to hydrated aldehyde evolved and reached an equilibrium composition of approximately 55 % imine.

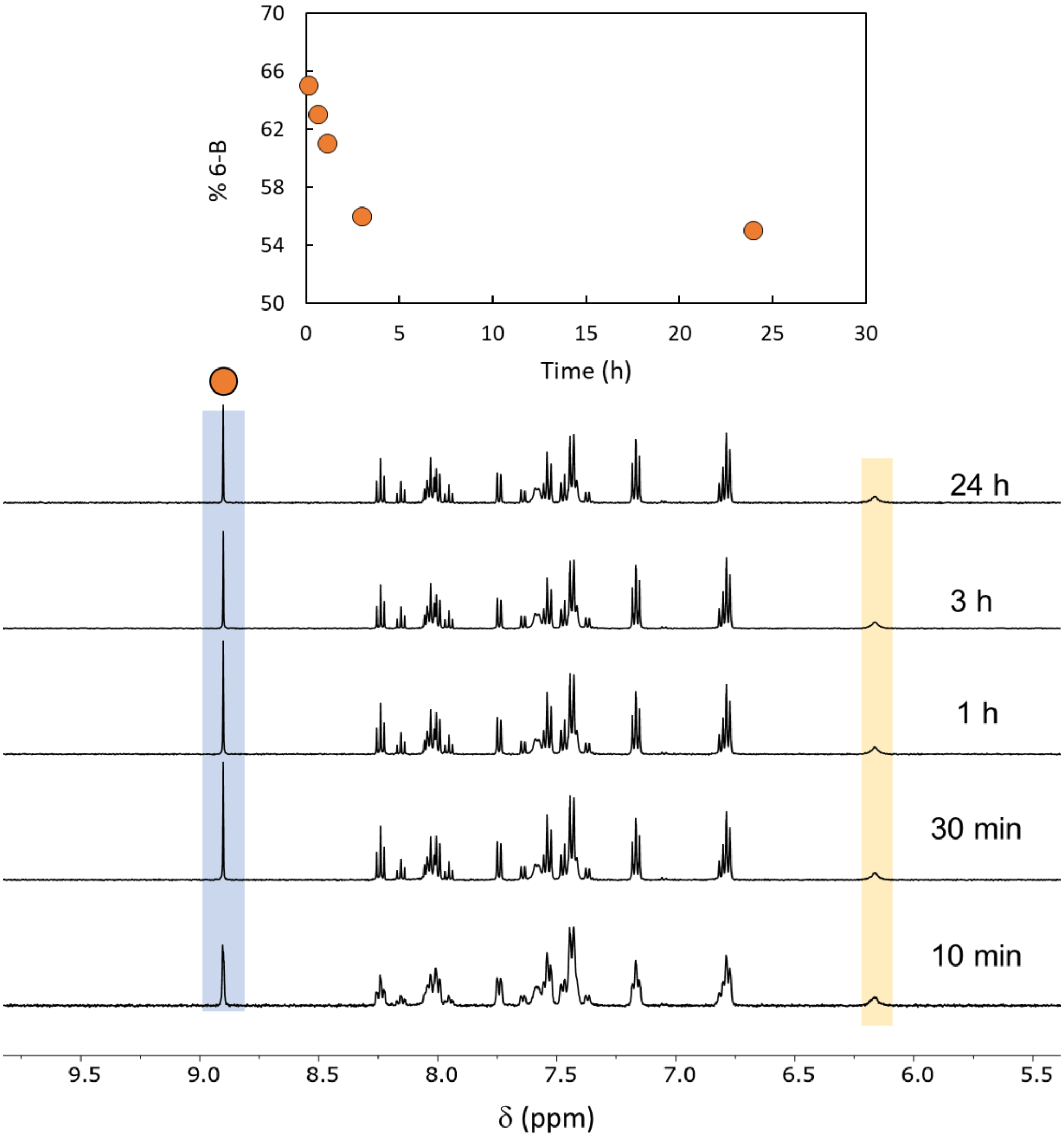

Figure S40. Parts of ^{1}H NMR spectra (500 MHz, D_2O) recorded at the indicated time intervals following dilution of **6-B** to 0.5 mM in 10 mM HEPES buffer (pD = 7.4). The blue highlighted region corresponds to the imine (**6-B**), while the yellow region indicates hydrated aldehyde **6**.

S3.3. Reaction kinetics for the formation of 6-C

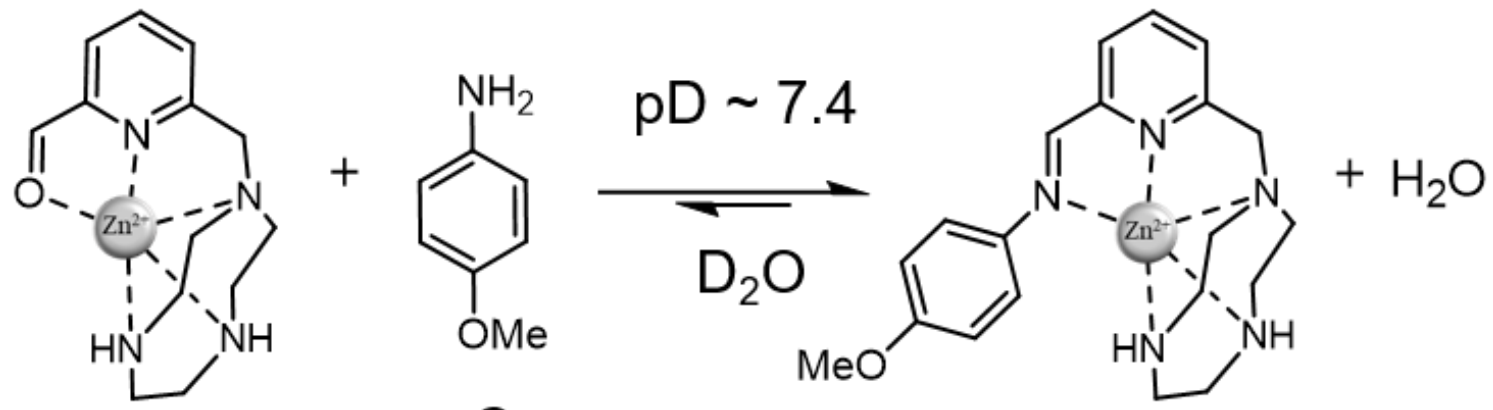

6 **C** **6-C**

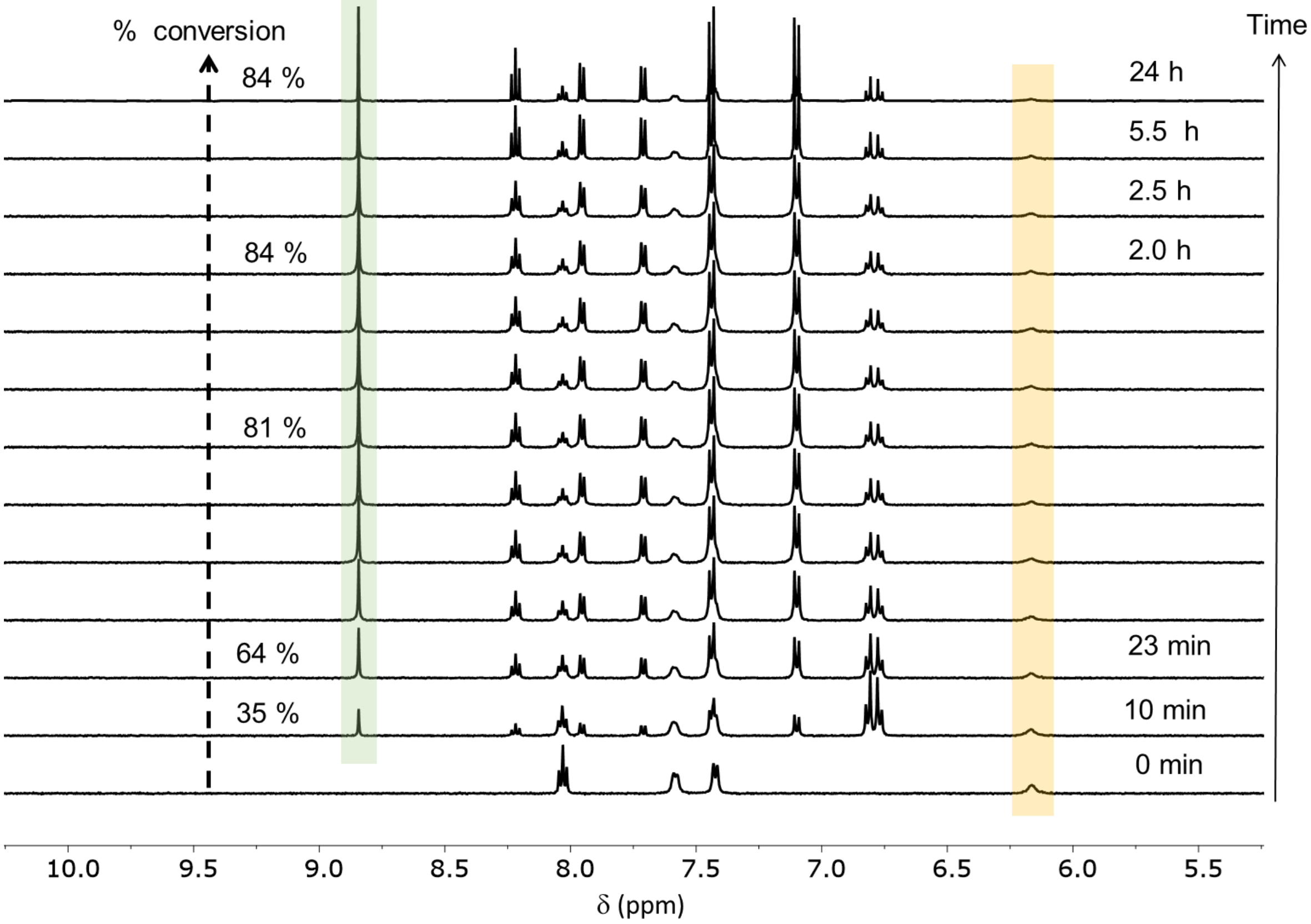

Figure S41. Parts of ^{1}H NMR spectra (500 MHz, D_2O) recorded at the indicated time intervals following mixing of **6** and **C** at 25 °C (pD = 7.4) in 10 mM HEPES buffer. The green highlighted region corresponds to the imine **6-C**, while the yellow region indicates hydrated aldehyde **6**. The percentage conversion was determined by integrating the combined signals of the hydrated aldehyde and imine, which were taken to represent 100% of the species present.

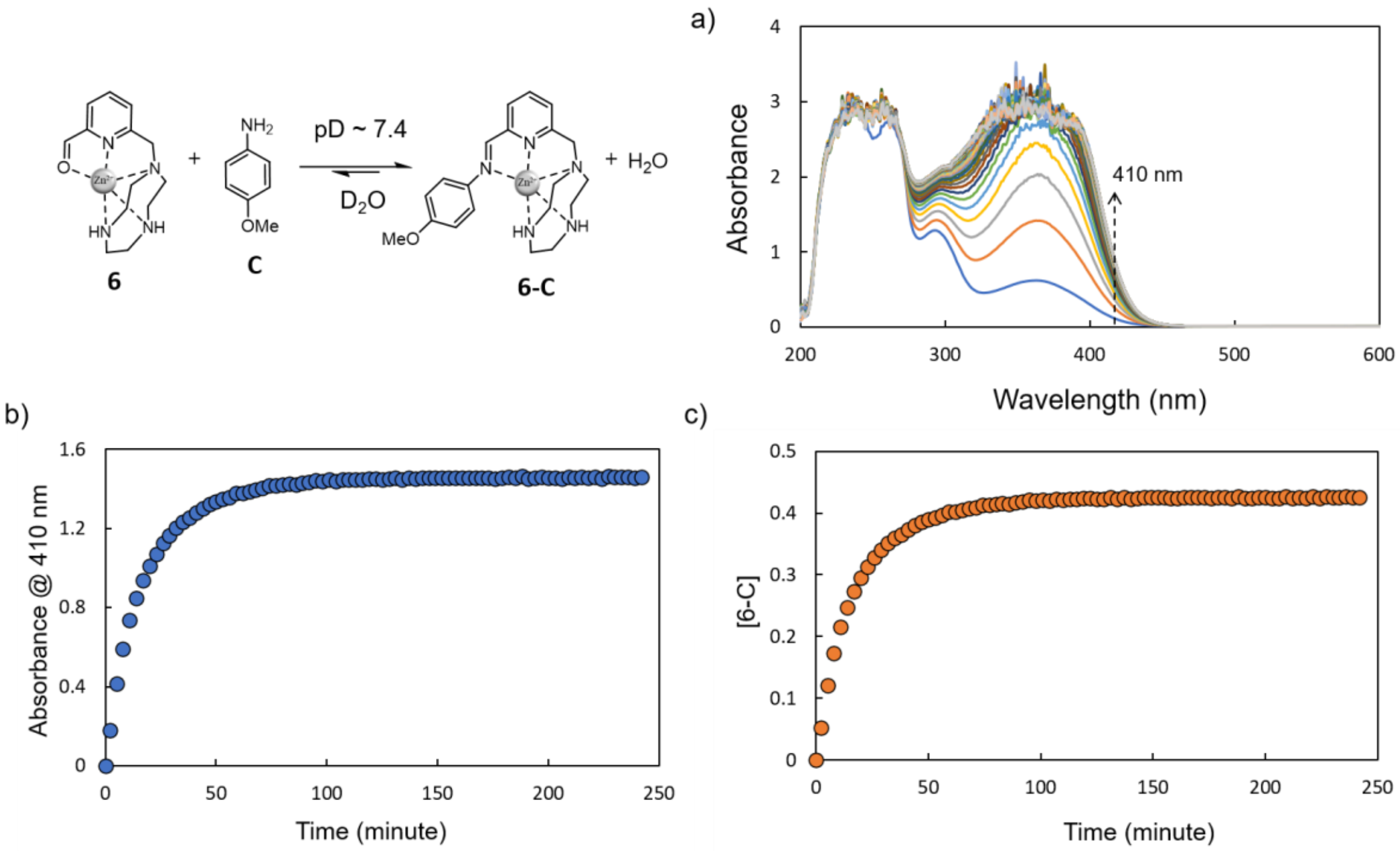

Figure S42. Kinetic analysis of **6-C** imine formation at pD 7.4 in 10 mM HEPES buffer (D_2O) monitored by UV-vis spectroscopy over 4 h. a) Time-dependent UV-vis spectra showing the progression of imine formation. b) Absorbance at 410 nm as a function of time, corresponding to the formation of **6-C**. c) Concentration of **6-C** versus time, obtained from UV-vis data using molar extinction coefficients (3.45 $mM^{-1}cm^{-1}$) determined from ^{1}H NMR quantification (Figure S41). The data were fitted to a second-order reversible kinetic model.

S3.4. Reaction kinetics for the formation of 6-D

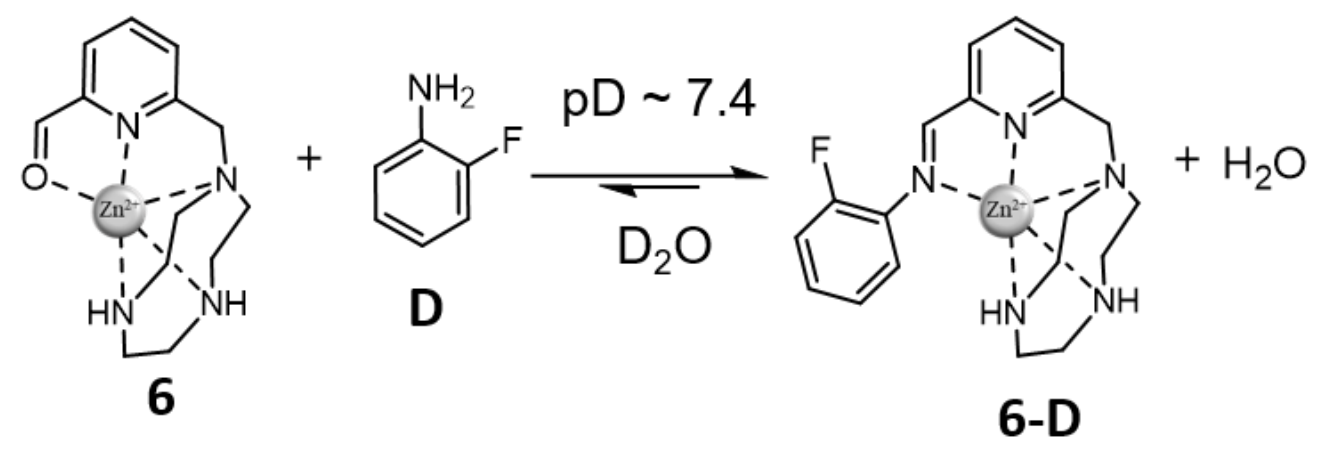

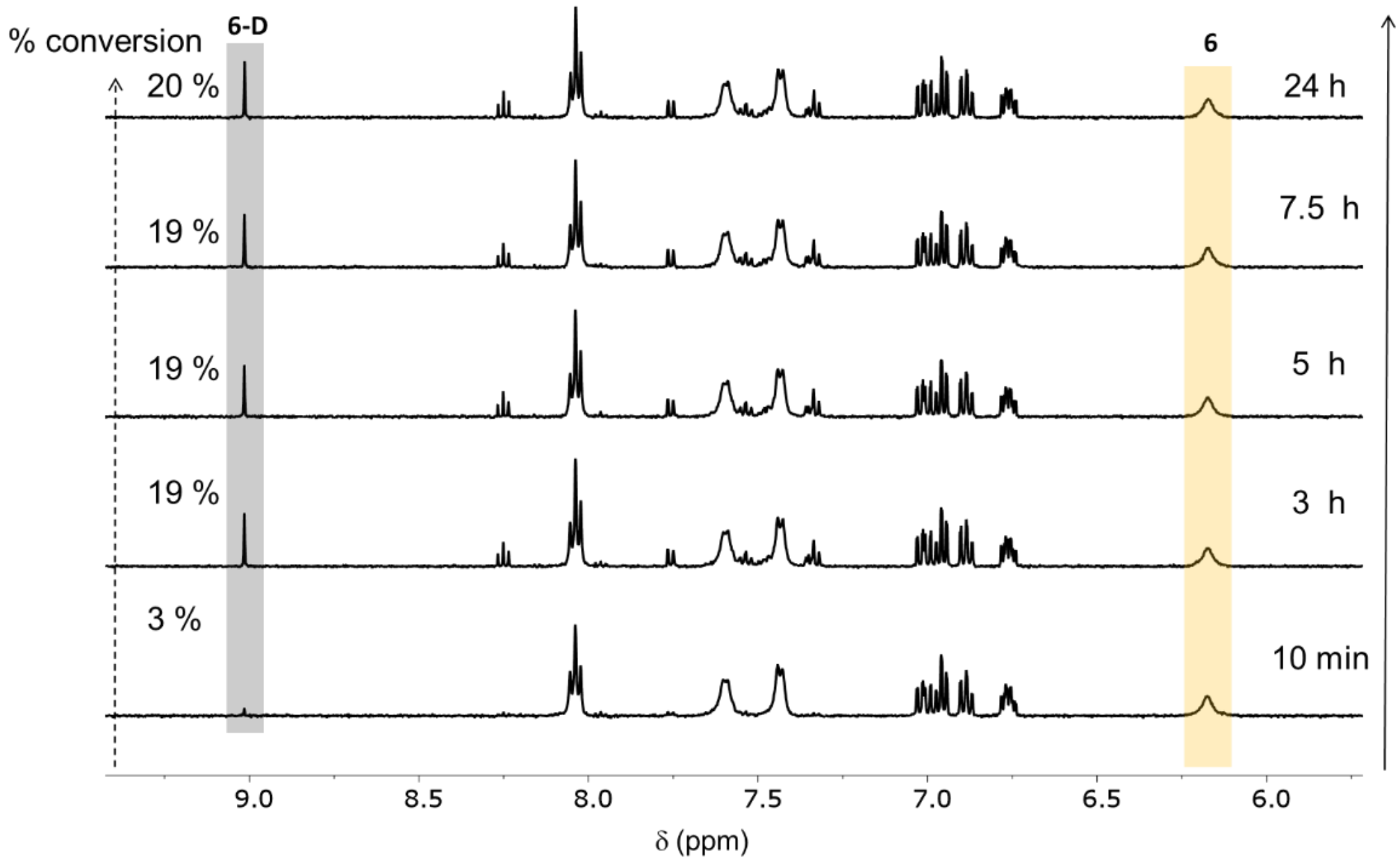

Figure S43. Part of ^1H NMR spectra (500 MHz, D_2O) recorded at the indicated time intervals following mixing of **6** and **D** at 25 °C (pD = 7.4) in 10 mM HEPES buffer. The grey highlighted region corresponds to the imine **6-D**, while the yellow region indicates hydrated aldehyde **6**.

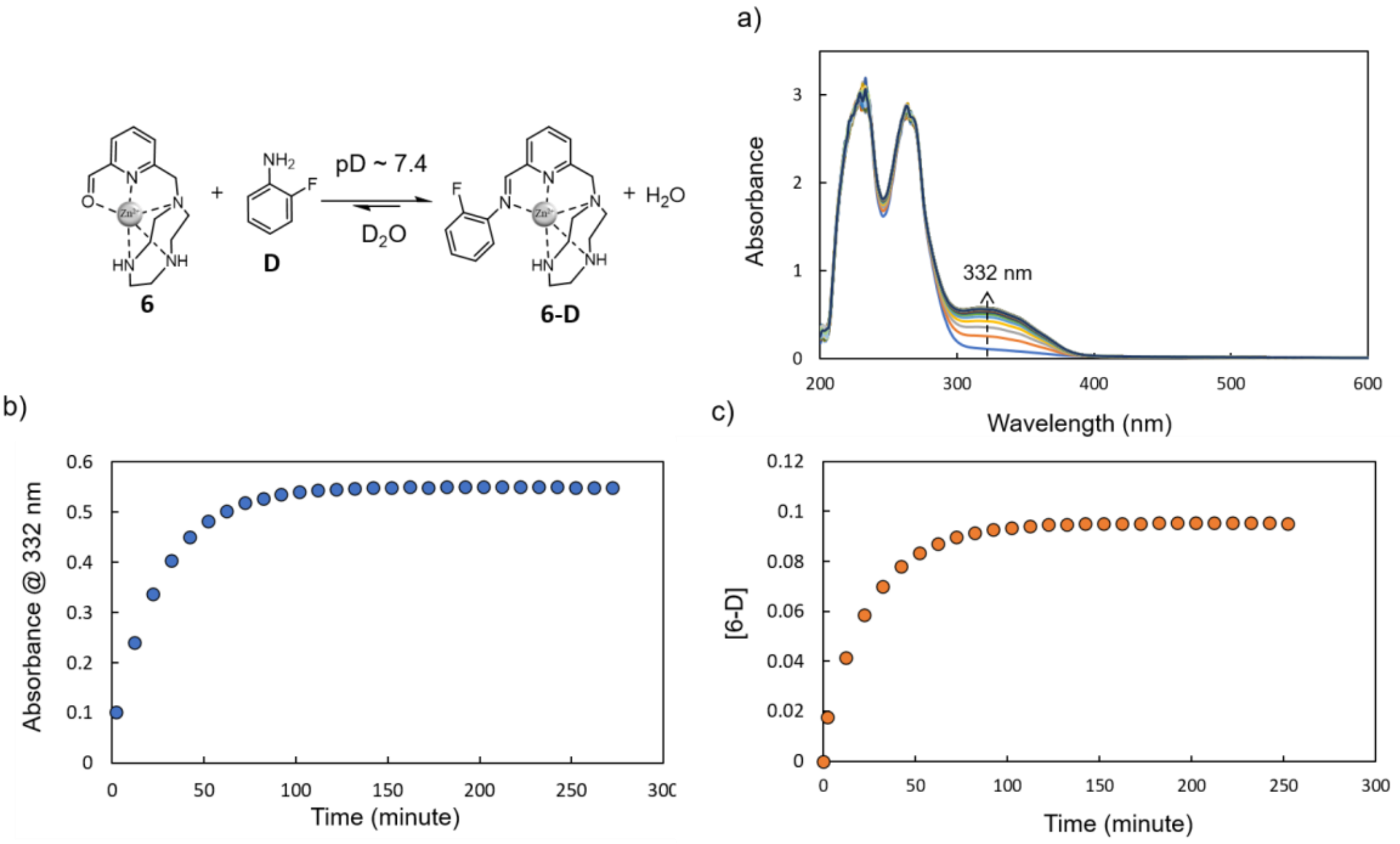

Figure S44. Kinetic analysis of **6-D** imine formation at pD 7.4 in 10 mM HEPES buffer (D_2O) monitored by UV-vis spectroscopy over 4 h. a) Time-dependent UV-vis spectra showing the progression of imine formation. b) Absorbance at 332 nm as a function of time, corresponding to the formation of **6-D**. c) Concentration of **6-D** versus time, obtained from UV-vis data using molar extinction coefficients (5.68 $mM^{-1}cm^{-1}$) determined from ^1H NMR quantification (Figure S43). The data were fitted to a second-order reversible kinetic model

S3.5. Reaction kinetics for the formation of 6-E

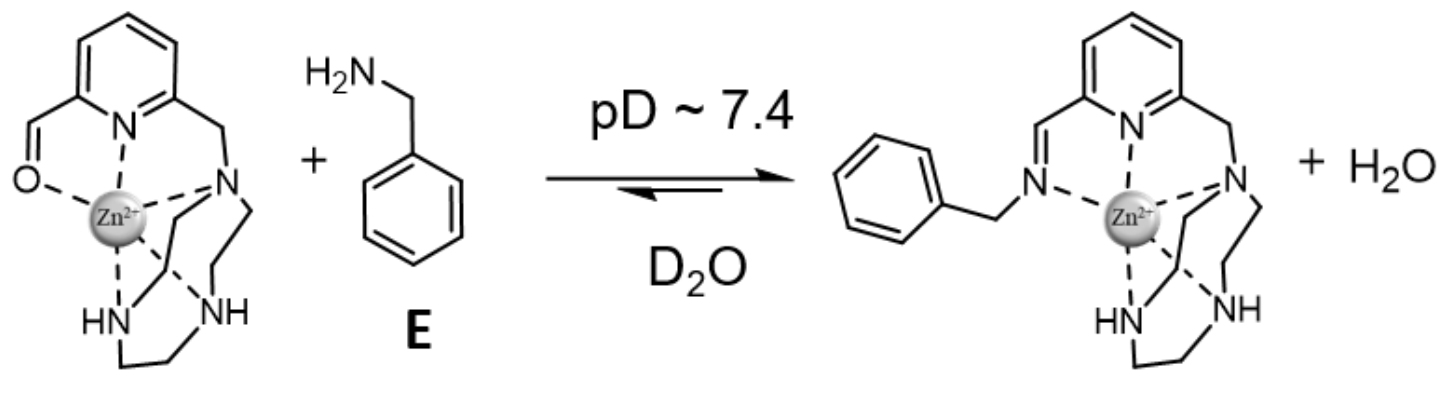

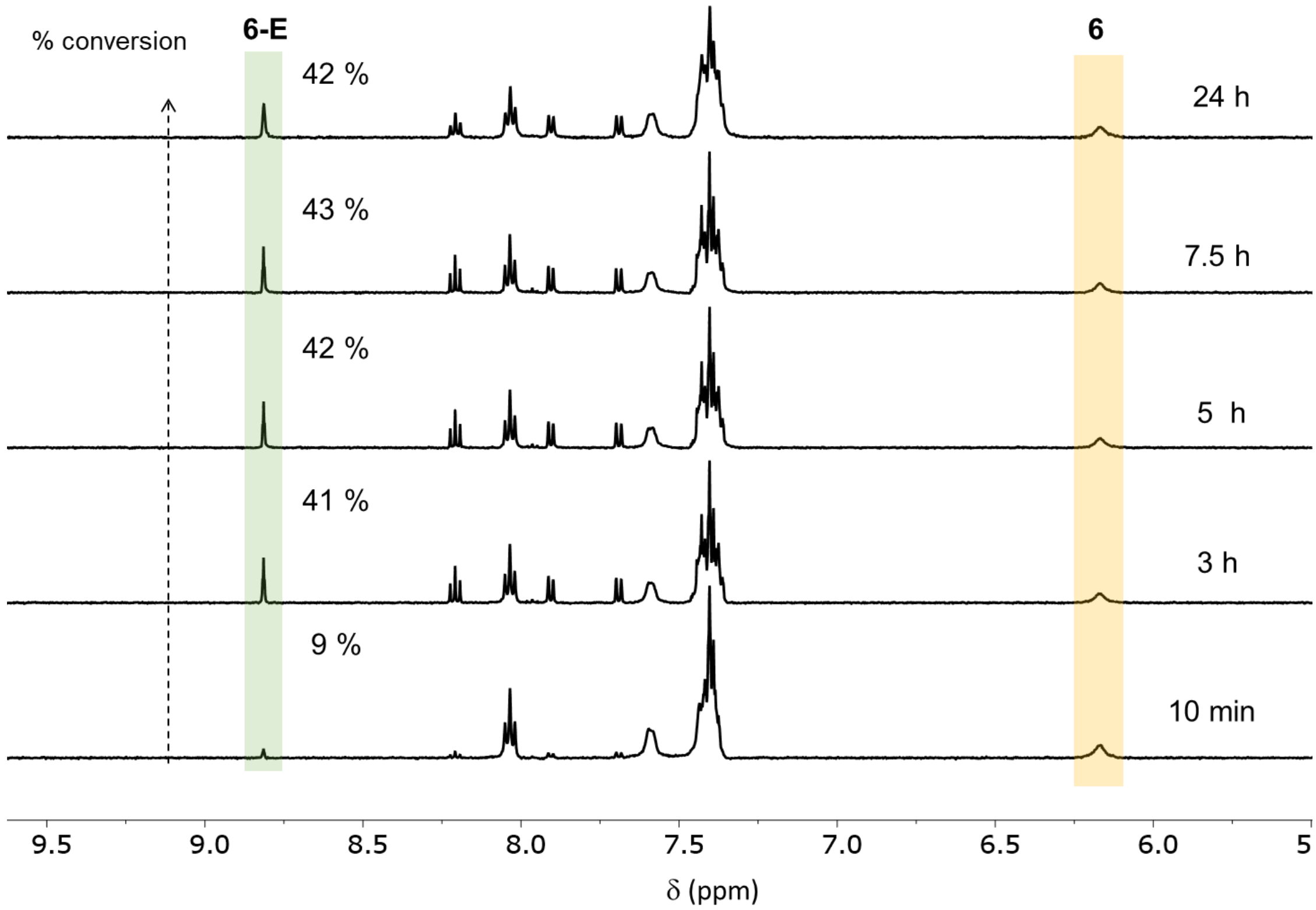

Figure S45. Part of ^1H NMR spectra (500 MHz, D_2O) recorded at the indicated time intervals following mixing of **6** and **E** at 25 °C (pD = 7.4) in 10 mM HEPES buffer. The green highlighted region corresponds to the imine **6-E**, while the yellow region indicates hydrated aldehyde **6**. The percentage conversion was determined by integrating the combined signals of the hydrated aldehyde and imine, which were taken to represent 100% of the species present.

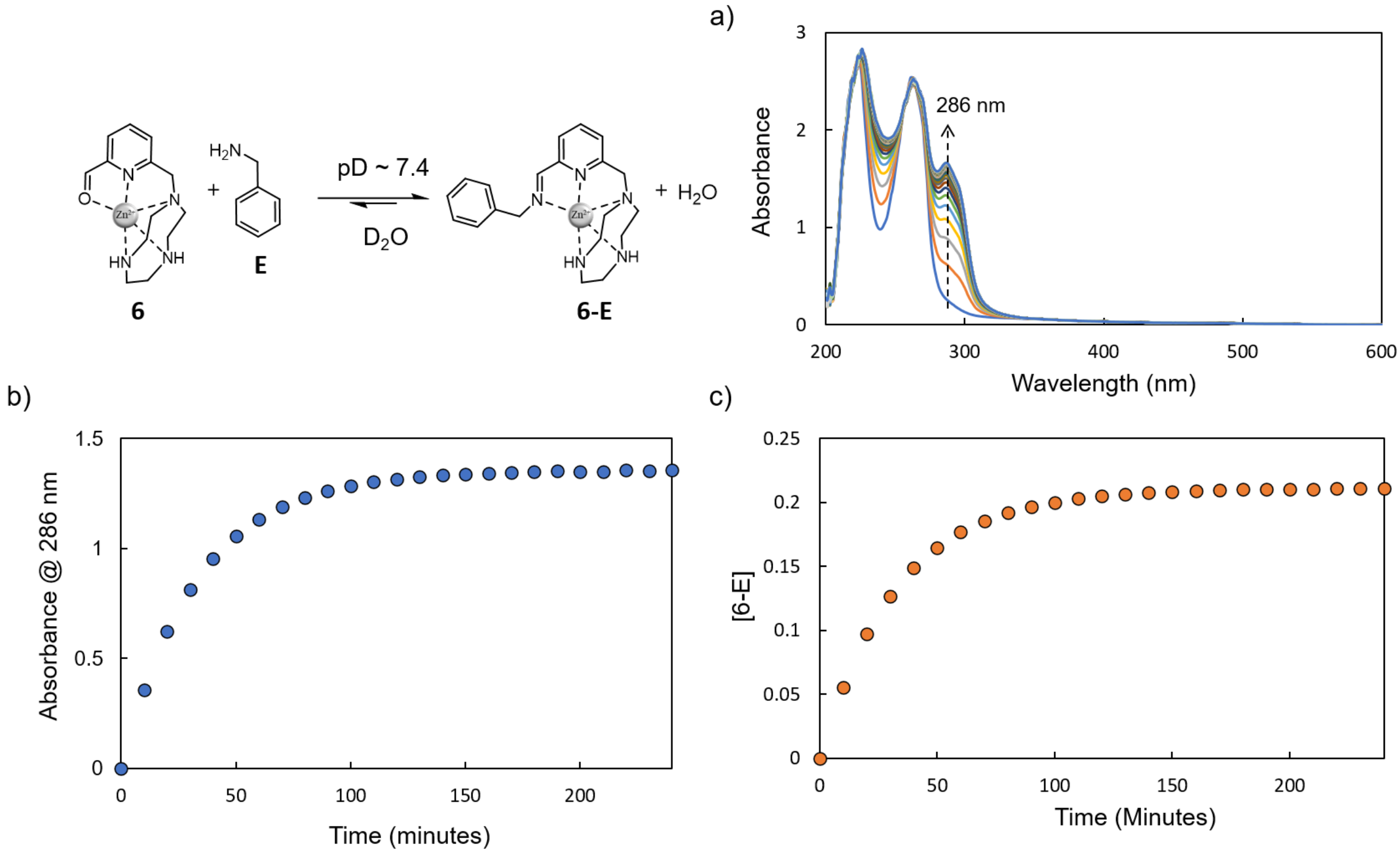

Figure S46. Kinetic analysis of **6-E** imine formation at pD 7.4 in 10 mM HEPES buffer (D_2O) monitored by UV-vis spectroscopy over 4 h. a) Time-dependent UV-vis spectra showing the progression of imine formation. b) Absorbance at 286 nm as a function of time, corresponding to the formation of **6-E**. c) Concentration of **6-E** versus time, obtained from UV–vis data using molar extinction coefficients (6.42 $mM^{-1}cm^{-1}$) determined from ^1H NMR quantification (Figure S45). The data were fitted to a second-order reversible kinetic model.

S3.6. Reaction kinetics for the formation of 6-F

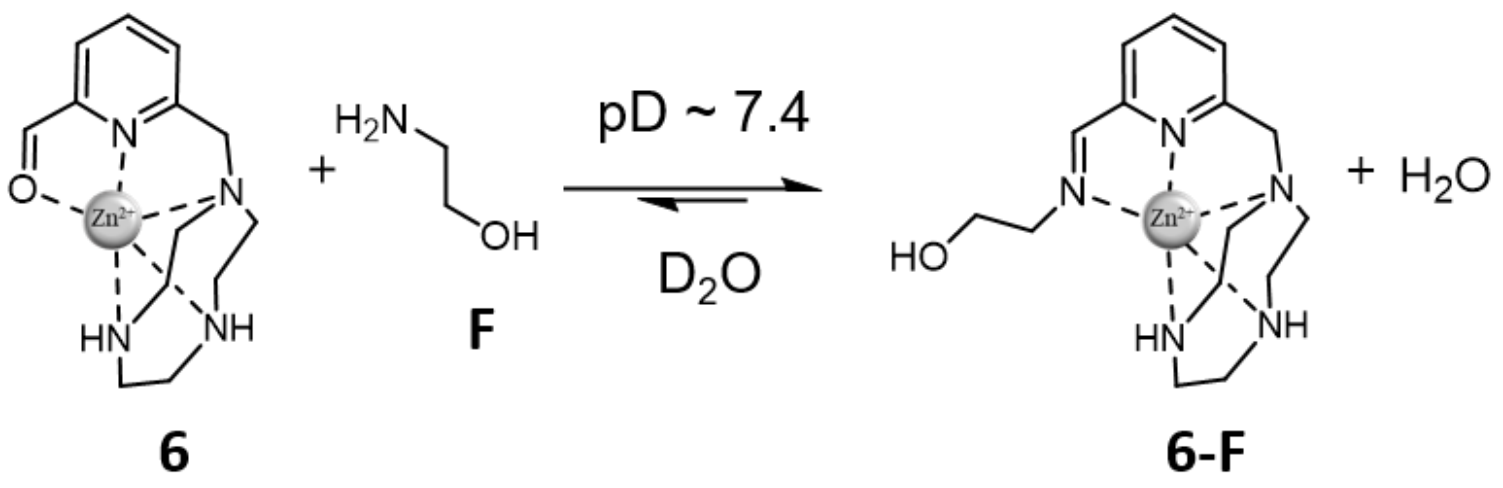

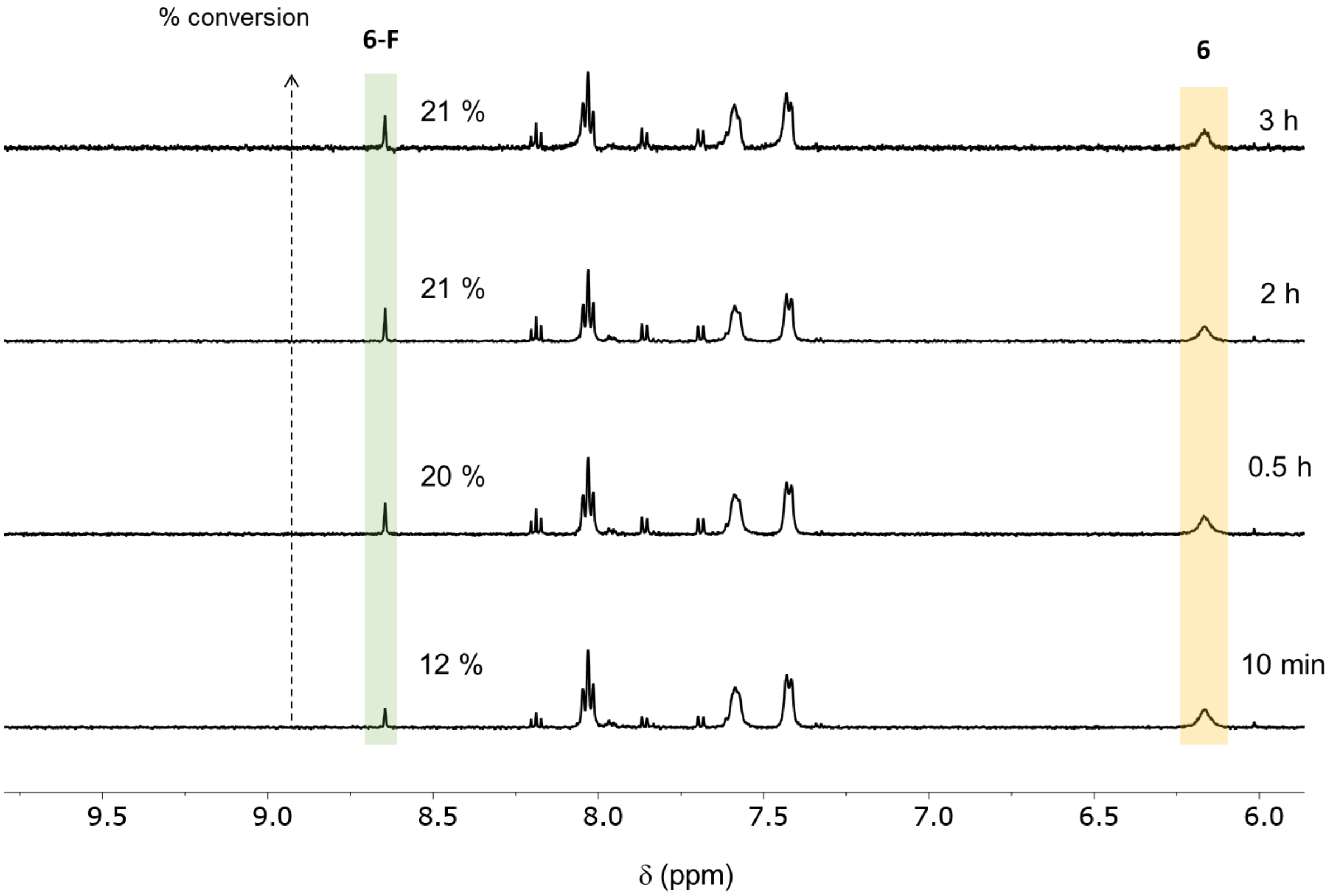

Figure S47. Parts of ^1H NMR spectra (500 MHz, D_2O) recorded at the indicated time intervals following mixing of **6** and **F** at 25 °C (pD = 7.4) in 10 mM HEPES buffer. The green highlighted region corresponds to the imine **6-F**, while the yellow region indicates hydrated aldehyde **6**. The percentage conversion was determined by integrating the combined signals of the hydrated aldehyde and imine, which were taken to represent 100% of the species present.

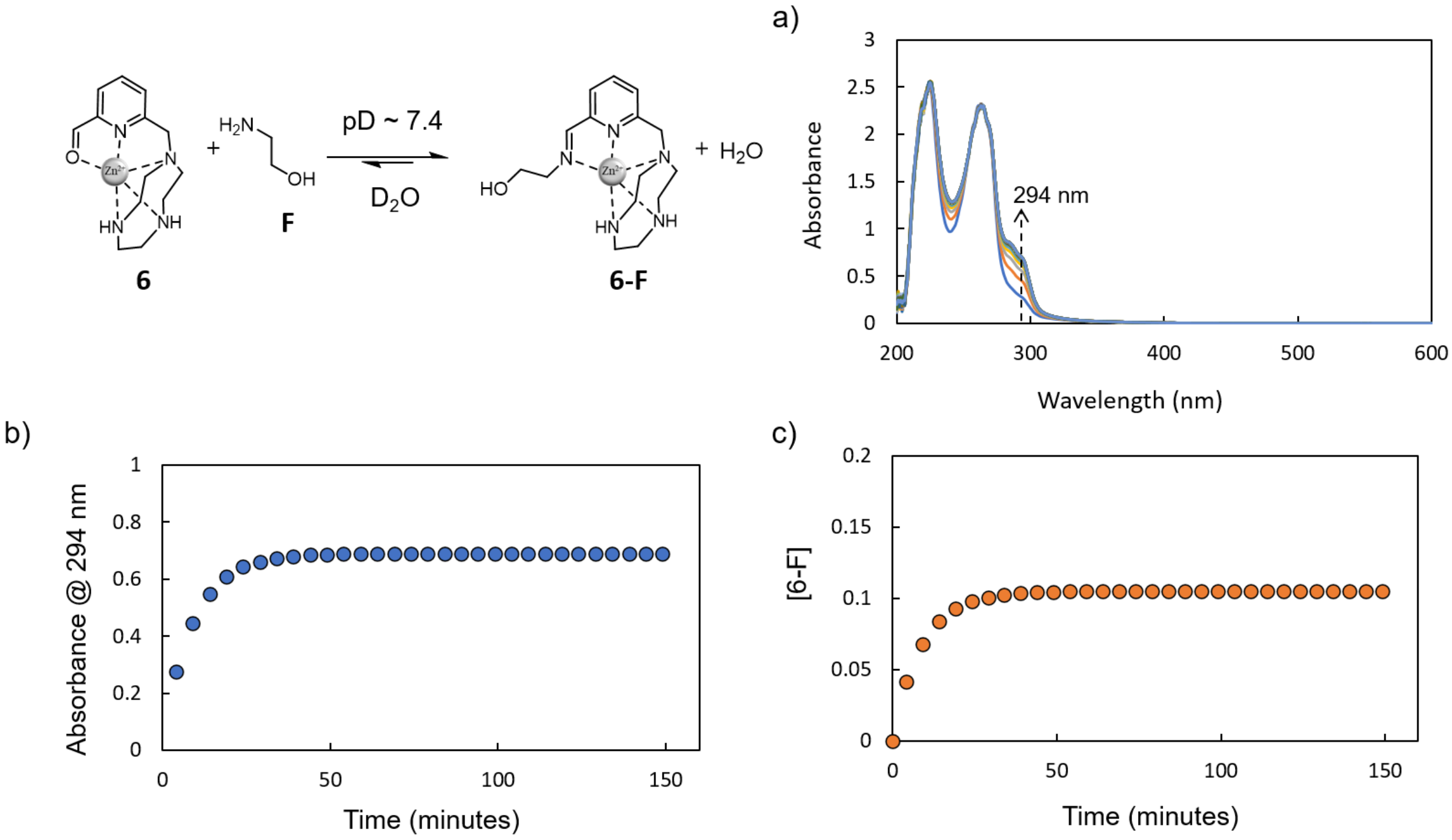

Figure S48. Kinetic analysis of **6-F** imine formation at pD 7.4 in 10 mM HEPES buffer (D_2O) monitored by UV-vis spectroscopy over 4 h. a) Time-dependent UV-vis spectra showing the progression of imine formation. b) Absorbance at 294 nm as a function of time, corresponding to the formation of **6-F**. c) Concentration of **6-F** versus time, obtained from UV-vis data using molar extinction coefficients (6.55 mM^{-1}cm^{-1}) determined from ^{1}H NMR quantification (Figure S47). The data were fitted to a second-order reversible kinetic model.

S3.7. Reaction kinetics for the formation of 6-G

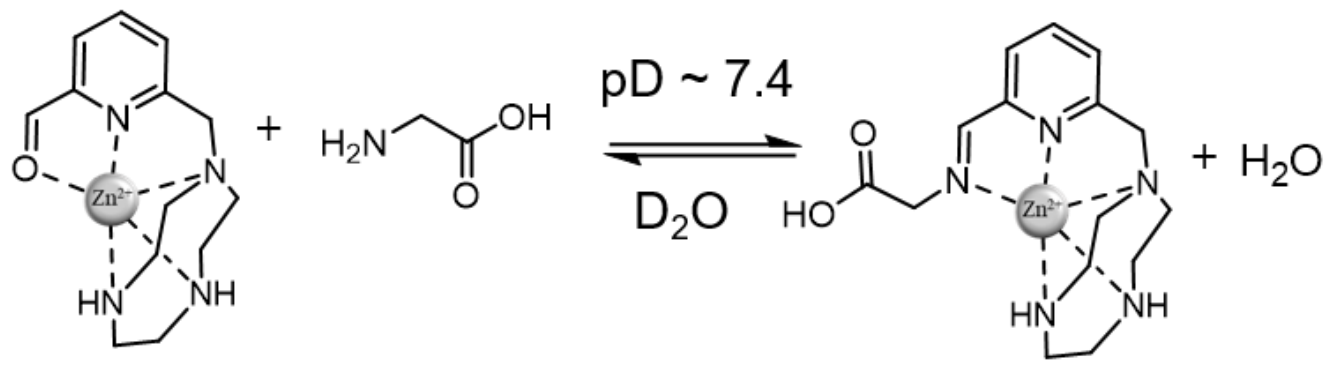

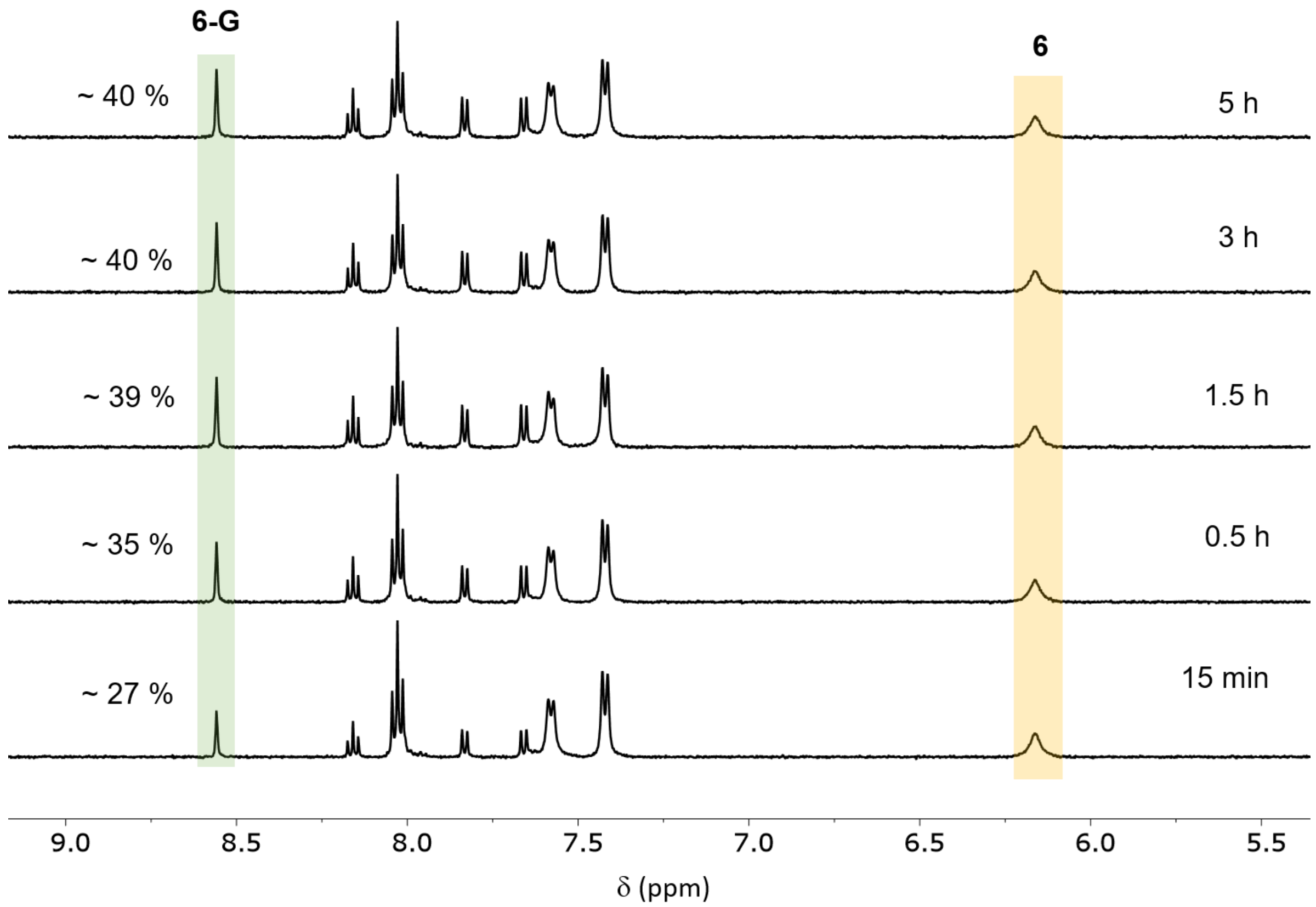

Figure S49. Parts of ^{1}H NMR spectra (500 MHz, D_2O) recorded at the indicated time intervals following mixing of **6** and **G** at 25 °C (pD = 7.4) in 10 mM HEPES buffer. The green highlighted region corresponds to the imine **6-G**, while the yellow region indicates hydrated aldehyde **6**. The percentage conversion was determined by integrating the combined signals of the hydrated aldehyde and imine, which were taken to represent 100% of the species present.

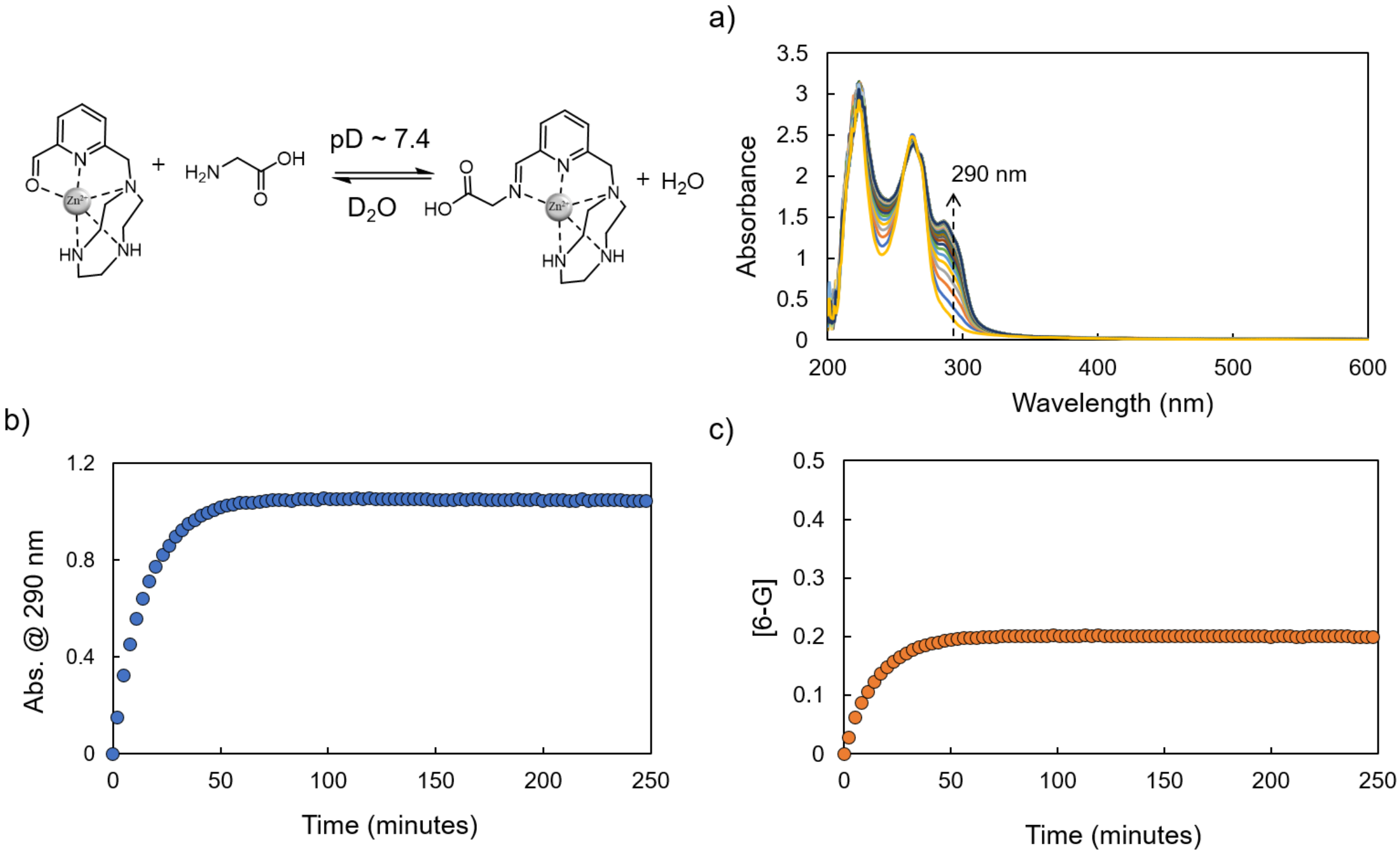

Figure S50. Kinetic analysis of **6-G** imine formation at D 7.4 in 10 mM HEPES buffer (D_2O) monitored by UV-vis spectroscopy over 4 h. a) Time-dependent UV-vis spectra showing the progression of imine formation. b) Absorbance at 294 nm as a function of time, corresponding to the formation of **6-G**. c) Concentration of **6-G** versus time, obtained from UV-vis data using molar extinction coefficients (5.23 $mM^{-1}cm^{-1}$) determined from ^{1}H NMR quantification (Figure S49). The data were fitted to a second-order reversible kinetic model.

S3.8. Role of Zn(II) in the formation of 6-C

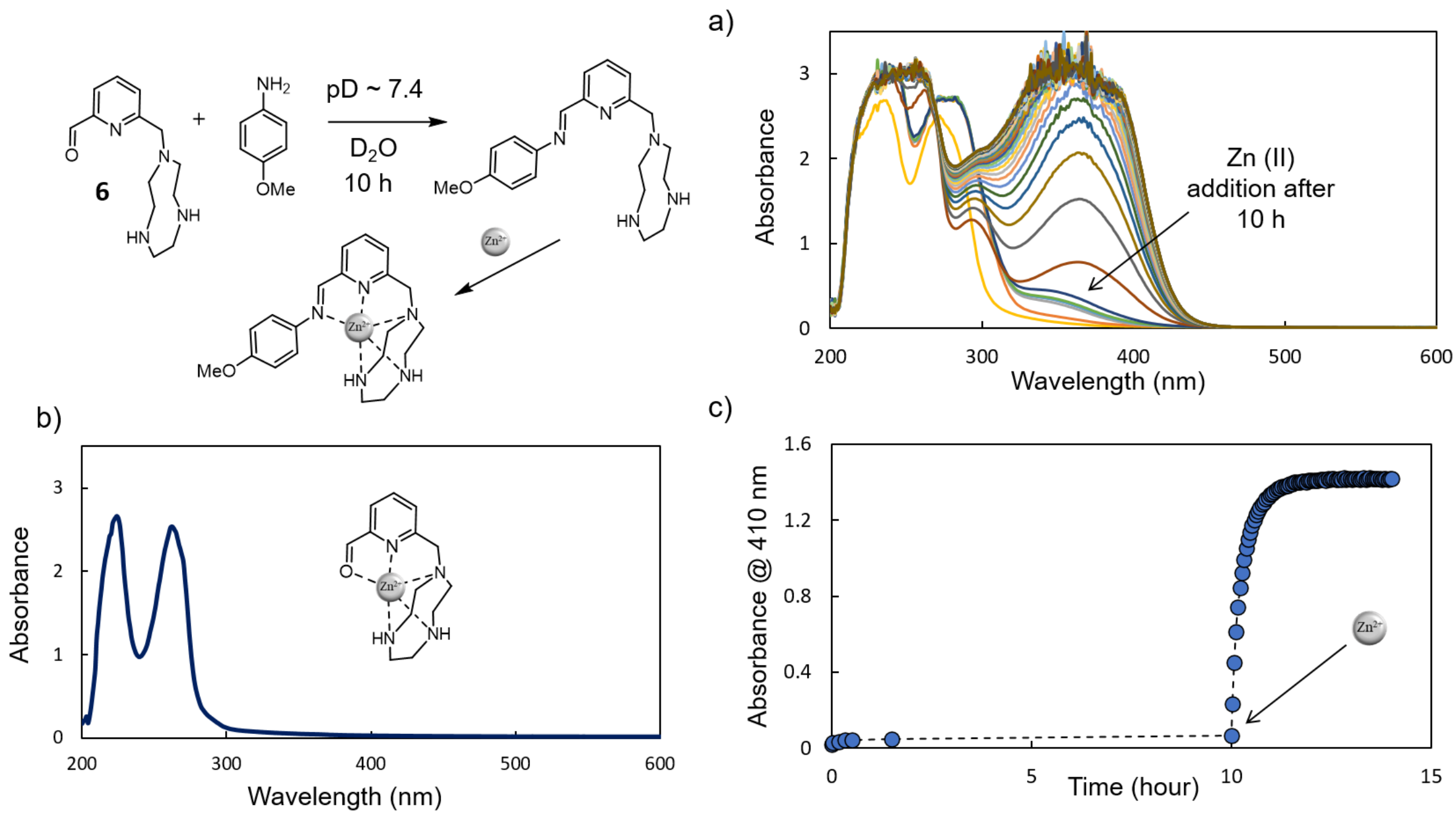

Figure S51. Role of Zn(II) in the activation of carbonyl during **6-C** imine formation monitored by UV-vis spectroscopy. a) Time-dependent UV-Vis spectra showing the kinetics of imine formation. (b) UV-vis spectrum of compound **6** with Zn(II) only to show negligible absorbance at 410 nm. (c) Absorbance at 410 nm as a function of time in the presence and absence of Zn(II). The arrow indicates the moment at which 1 equivalent of $Zn(NO_3)_2$ is added.

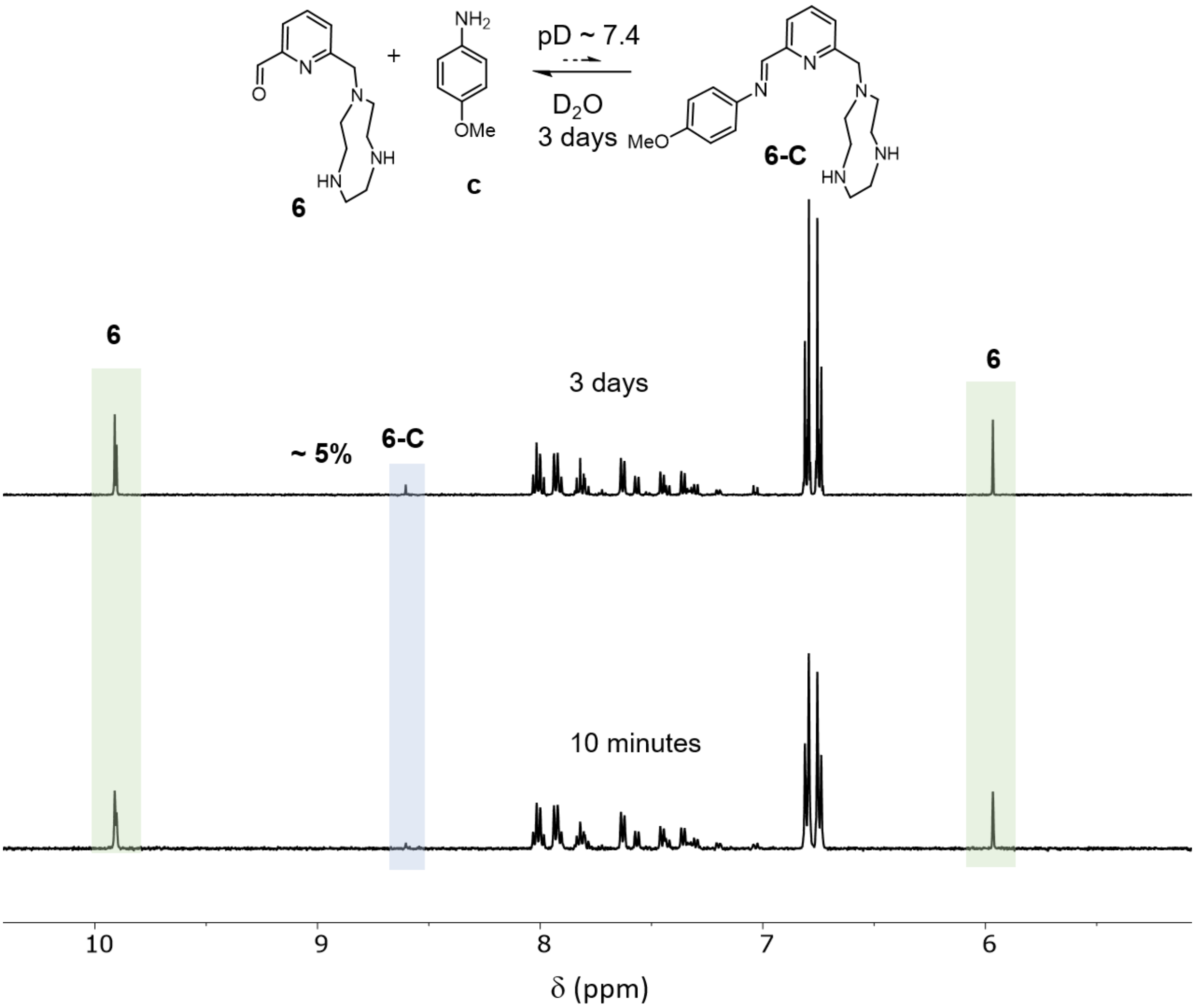

Figure S52. Parts of ^{1}H NMR spectra (500 MHz, D_2O) recorded at the indicated time intervals following mixing of **6** and **G** at 25 °C (pD = 7.4) in 10 mM HEPES buffer without Zn(II) ion. The green highlighted region corresponds to **6** aldehyde and hydrated aldehyde, while the blue region indicates imine 6-C. The percentage conversion was determined by integrating the combined signals of the hydrated aldehyde and imine, which were taken to represent 100% of the species present. Only ~ 5 % of 6-C formed after 3 days.

S3.9. Kinetics for the formation of 6-C at lower concentrations

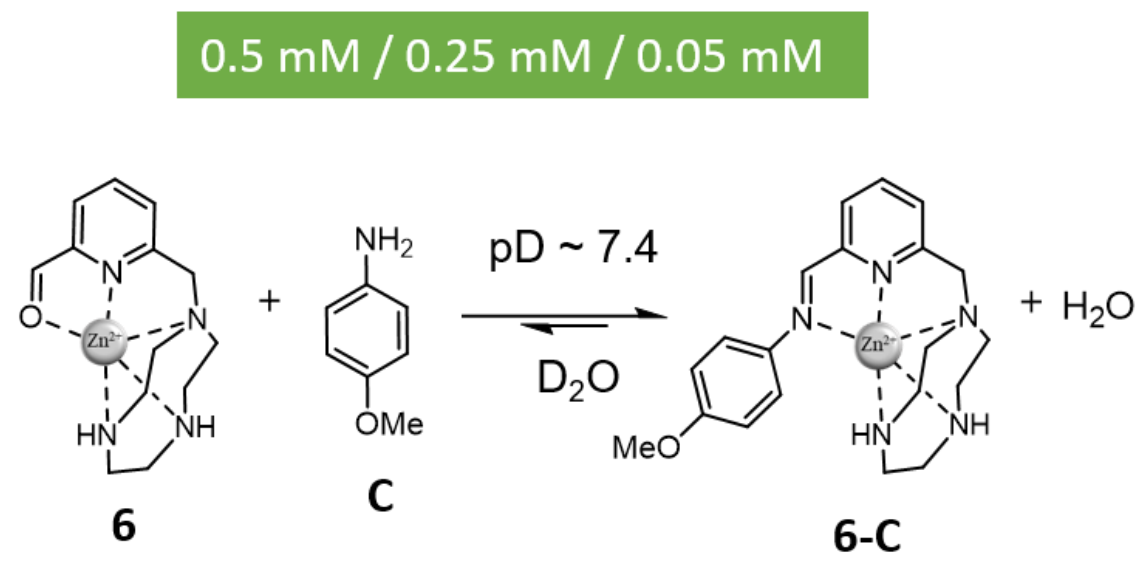

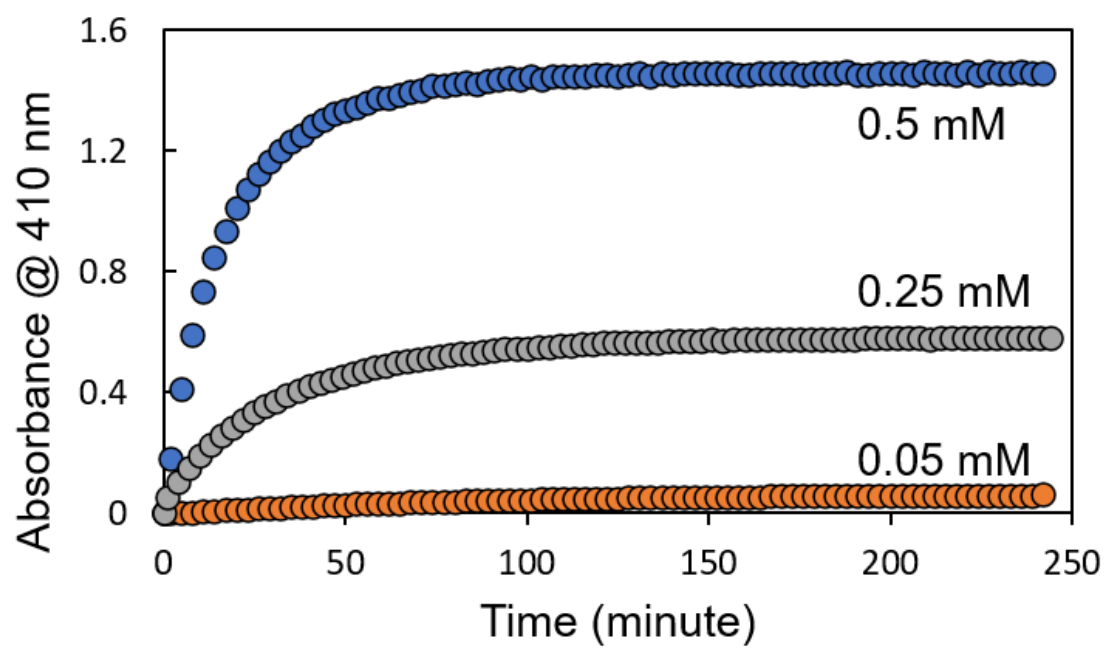

Figure S53. Kinetic analysis of **6-C** formation at varying concentrations in 10 mM HEPES buffer (D_2O) at pD 7.4, monitored by UV-vis spectroscopy over 4 h. Comparison of absorbance at 410 nm as a function of time for different initial concentrations (0.5 mM, 0.25 mM, and 50 µM), corresponding to the formation of **6-C**. The kinetic data were fitted to a second-order reversible model.

S3.10. Kinetics of 6-C formation with 3 equivalents of C

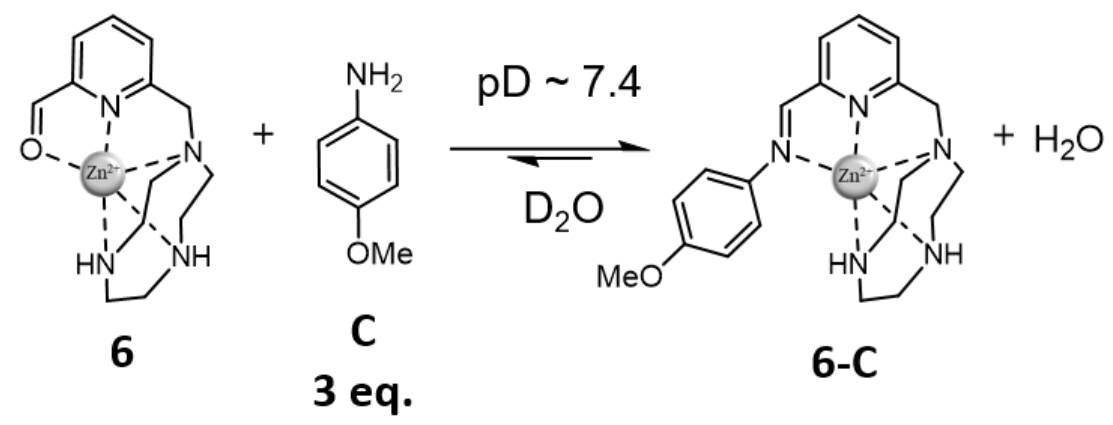

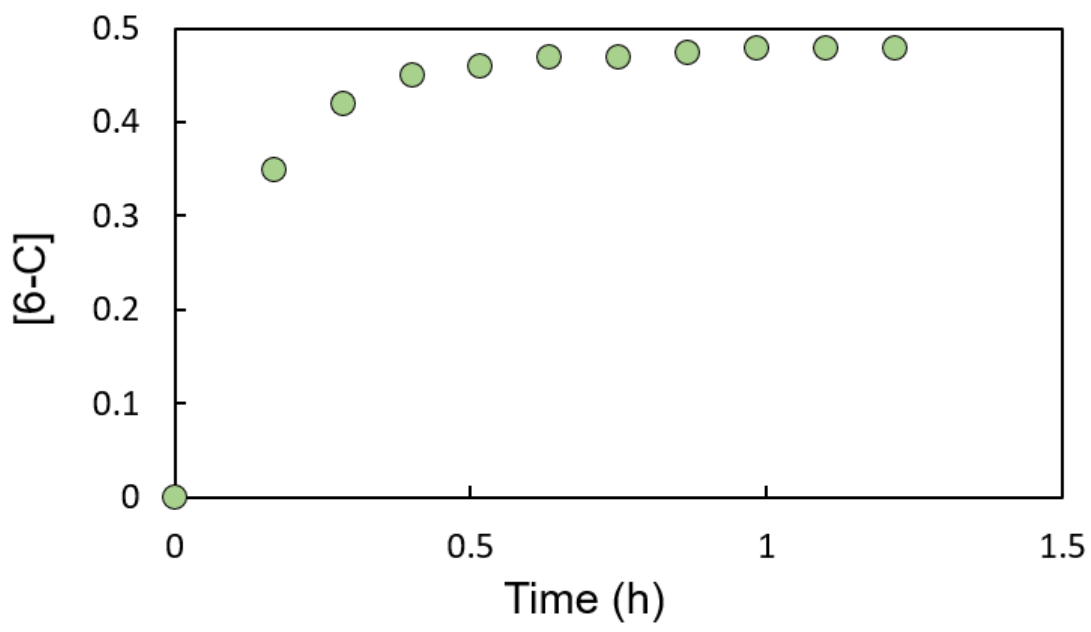

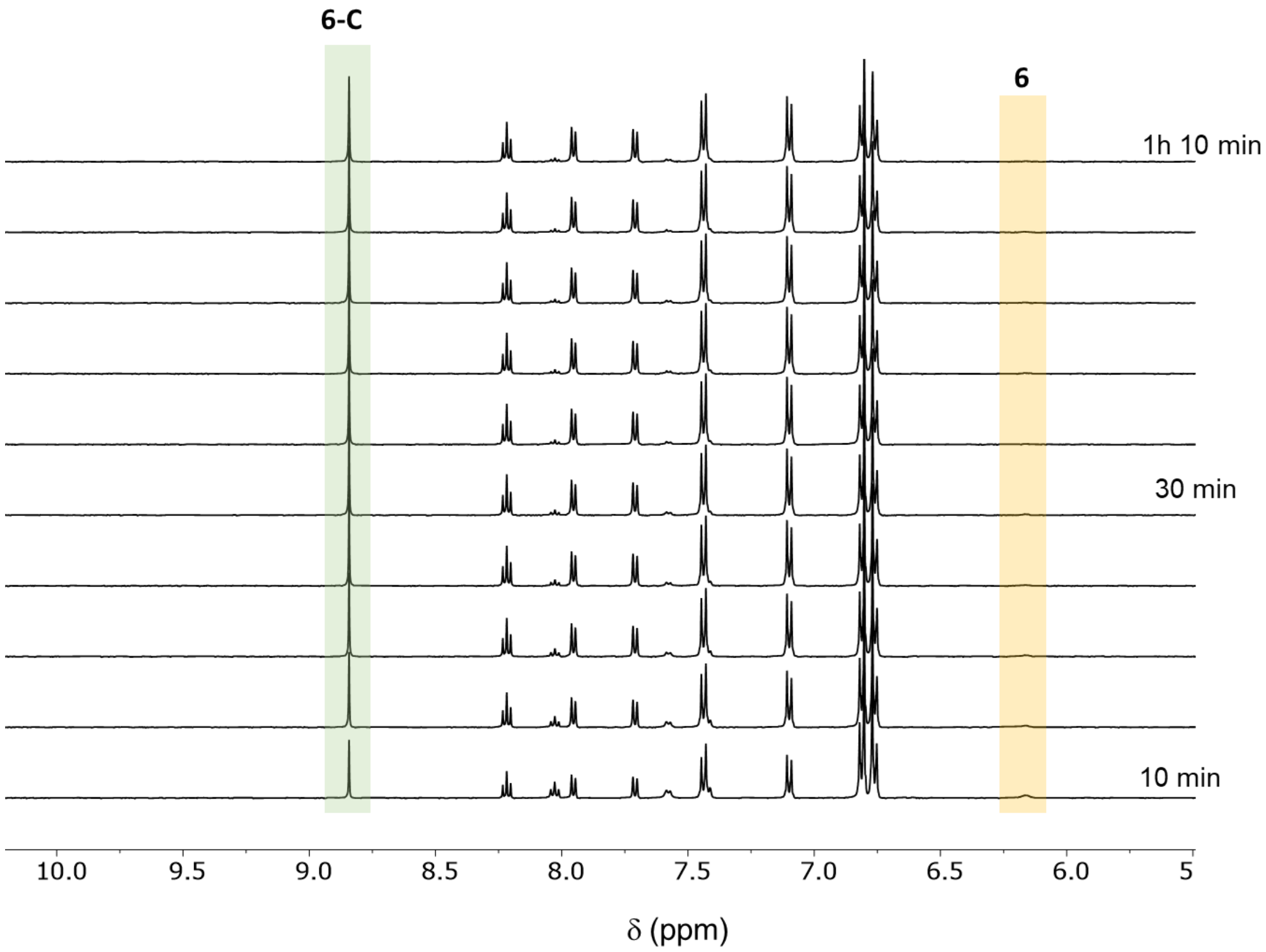

Figure S54. Parts of the ^1H spectra (500 MHz, D_2O) recorded between the indicated time intervals after mixing **6** (0.5 mM) and **C** (1.5 mM) (25 °C, pD = 7.4) with 10 mM of HEPES buffer. The yellow rectangle highlights the hydrated peak of aldehyde **6** and the green rectangles highlight the imine peaks corresponding to **6-C**. Percentage conversion is determined using the combined integration of aldehyde, hydrated aldehyde and imine to 100 %.

S3.11. Characterization of imines 6-B and 6-C

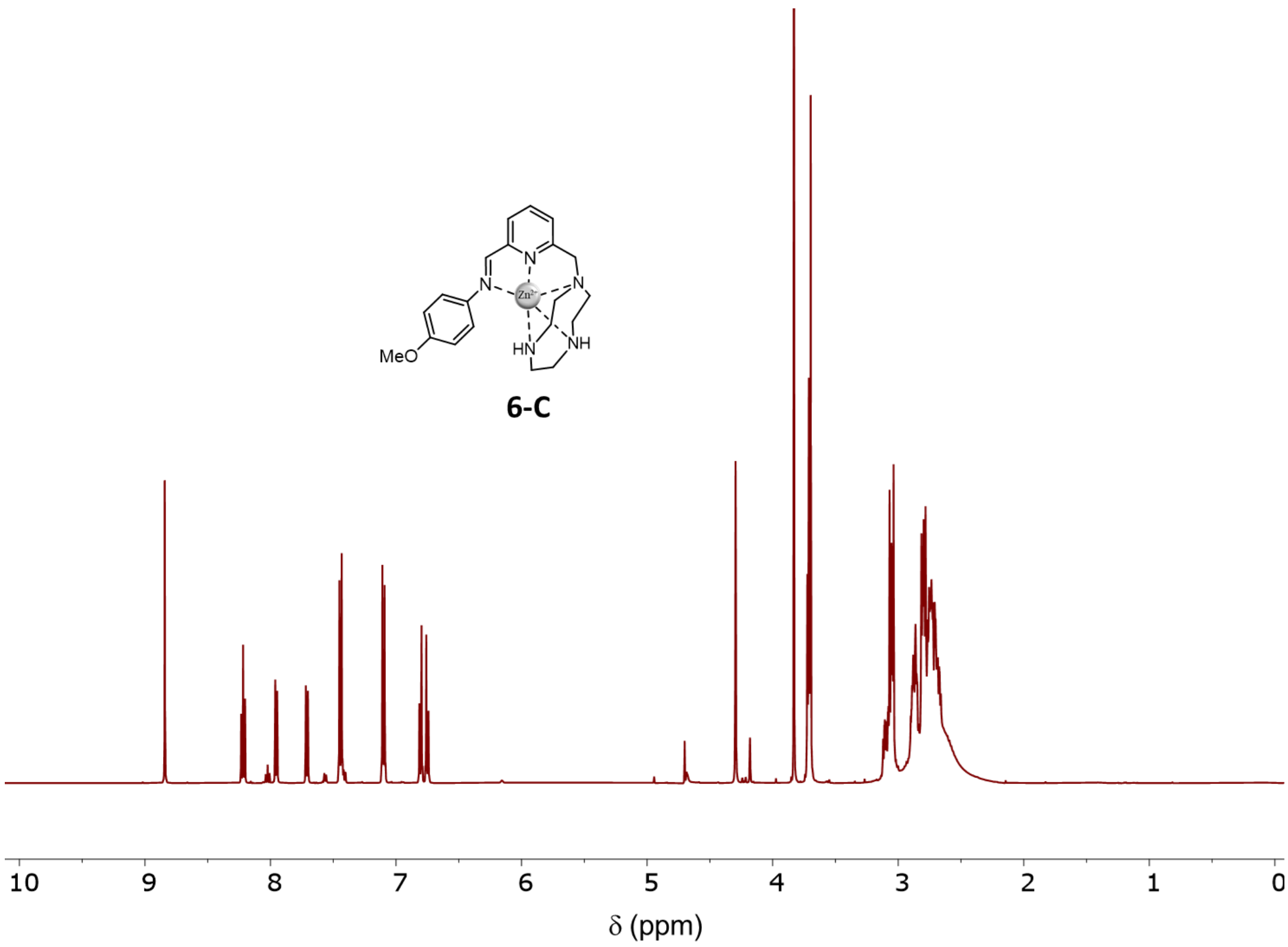

Figure S55. ^{1}H NMR spectrum of imine **6-C**, [**6-C**] = 5 mM, 10 mM HEPES in D_2O and 25 °C.

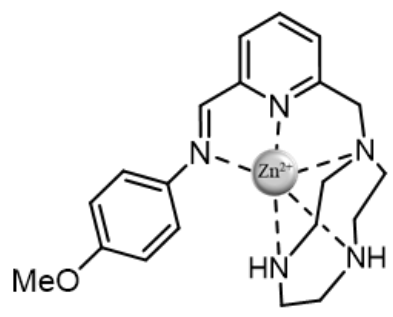

6-C

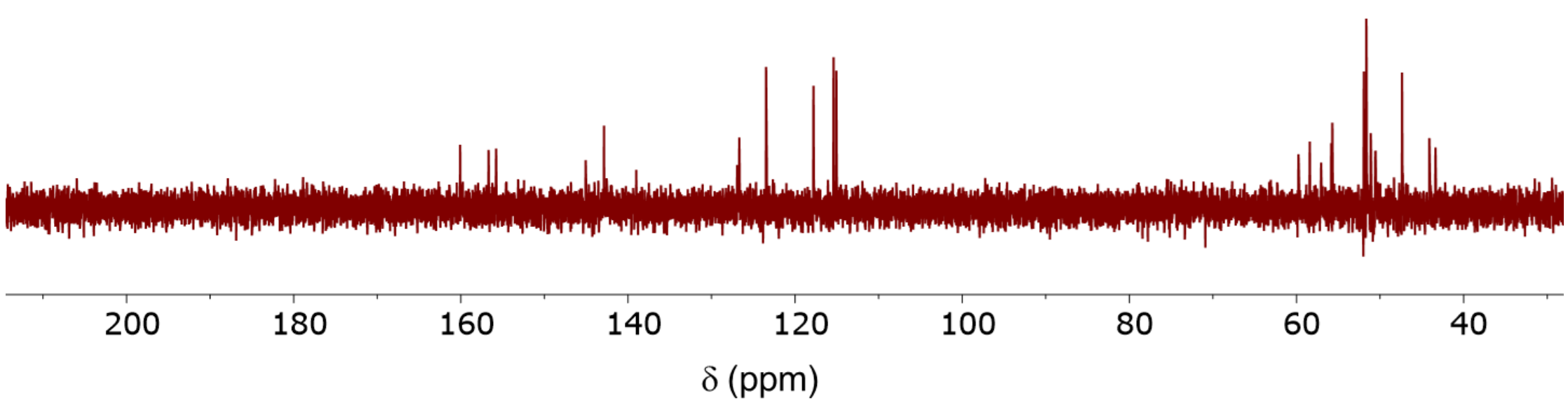

Figure S56. ^{13}C NMR spectrum of imine **6-C**, [**6-C**] = 5 mM, 10 mM HEPES in D_2O and 25 °C.

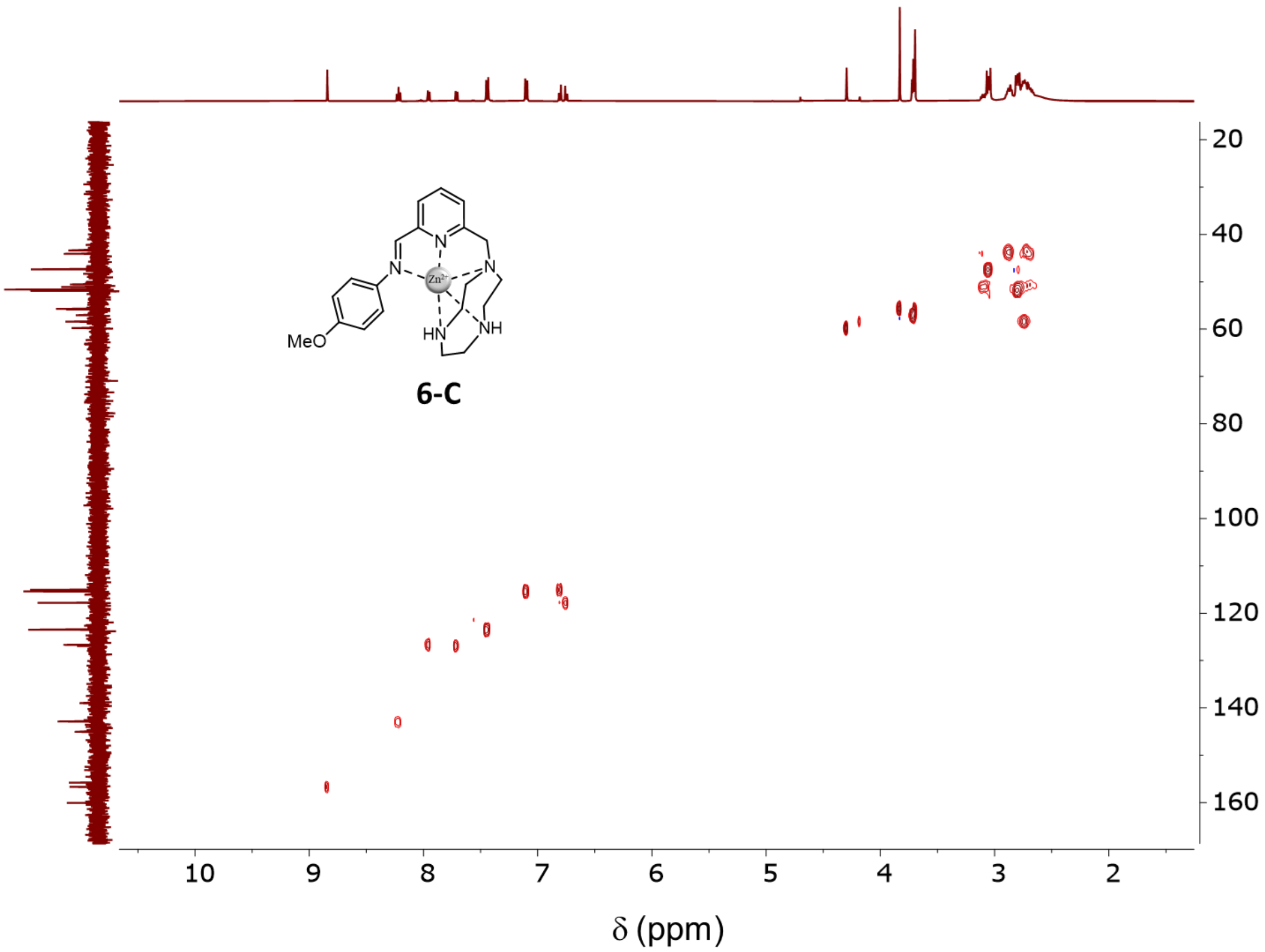

Figure 57. ^{1}H-^{13}C HSQC spectrum of imine **6-C**, [**6-C**] = 5 mM, 10 mM HEPES, D_2O and 25 ^{o}C.

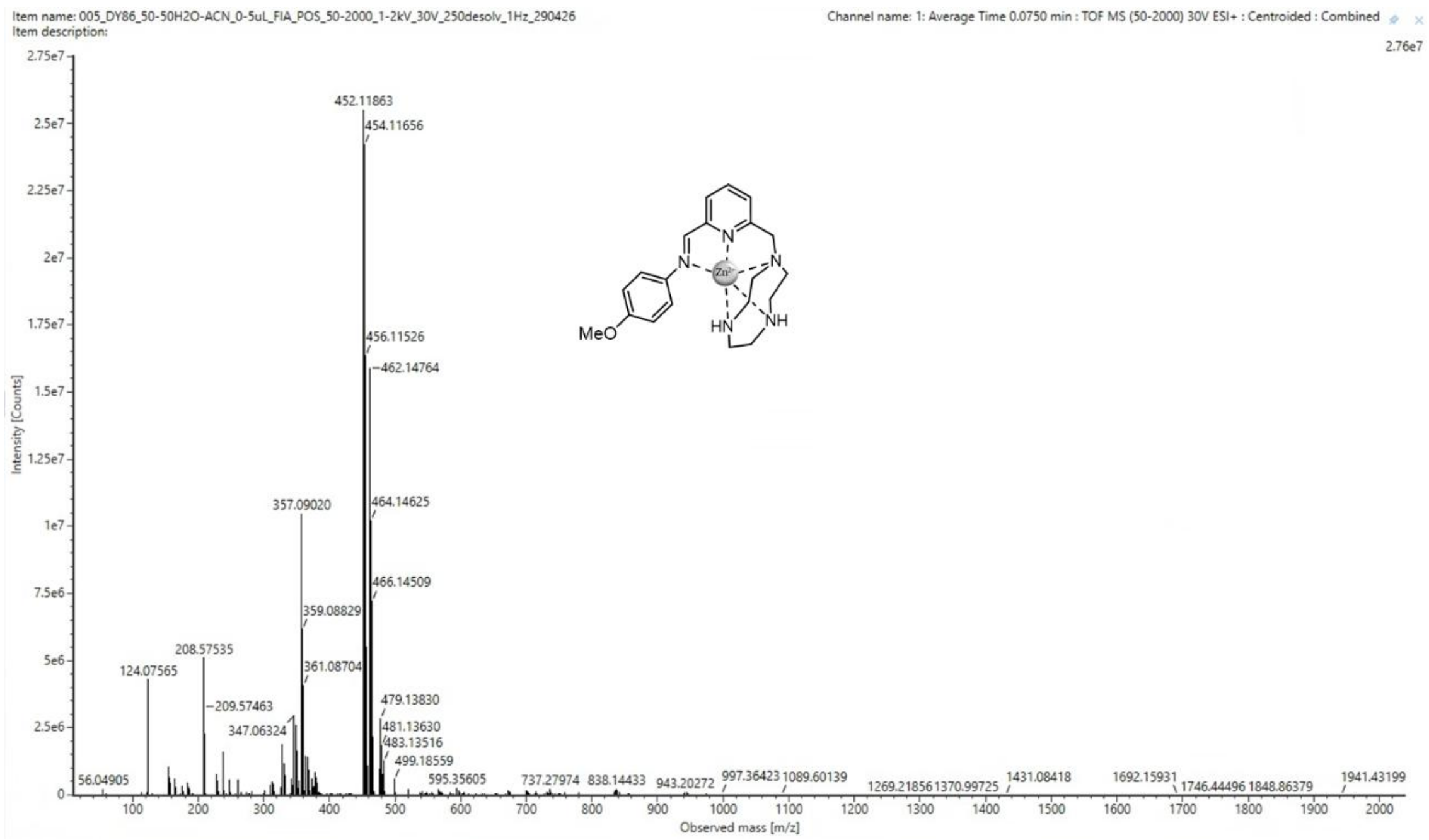

Figure S58. HRMS spectrum of imine **6-C**. Calculated Mass, $[M+Cl^{-}]$ = 452.1190

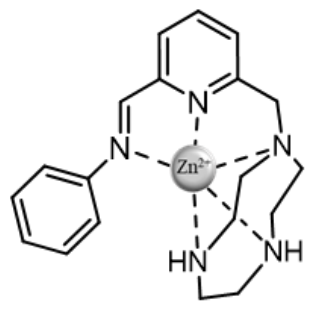

6-B

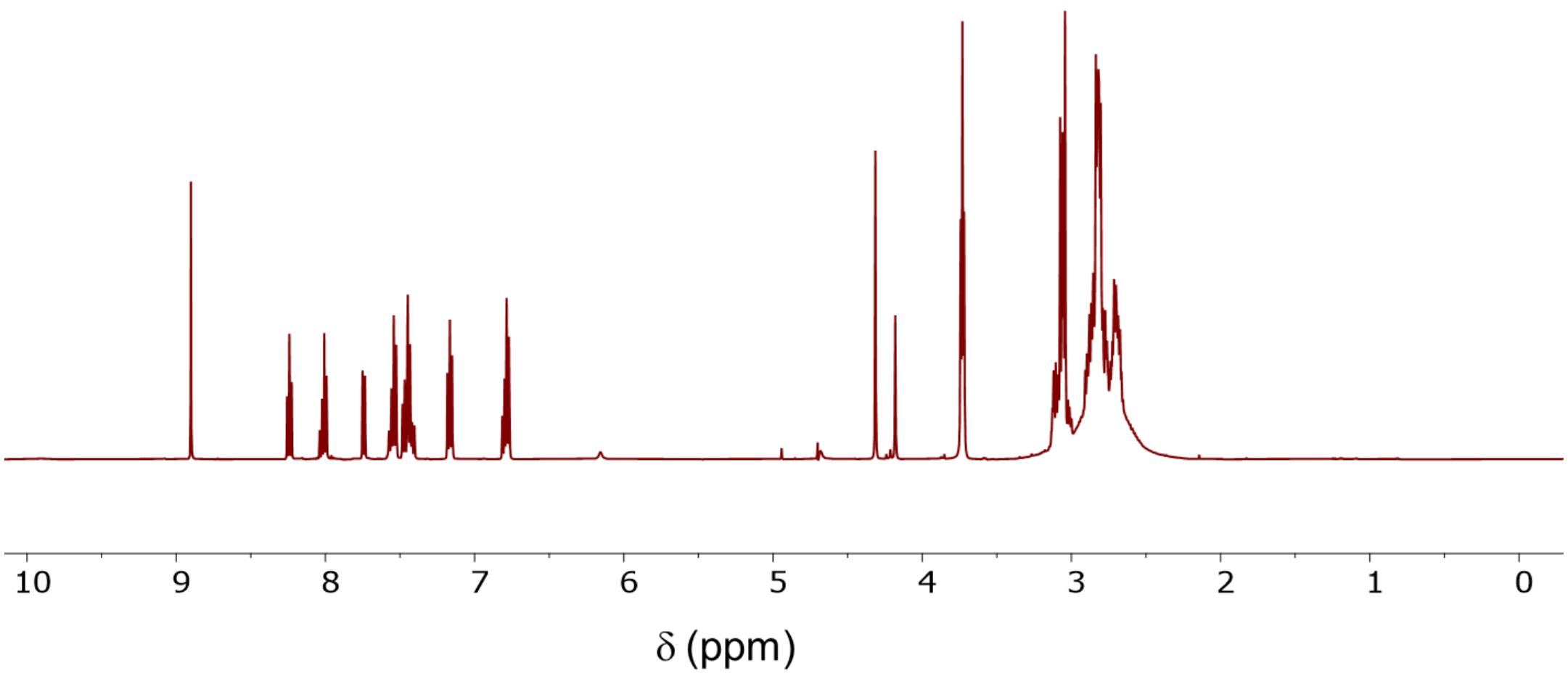

Figure S59. ^{1}H NMR spectrum of imine **6-B**, [**6-B**] = 5 mM, 10 mM HEPES in D_2O and 25°C.

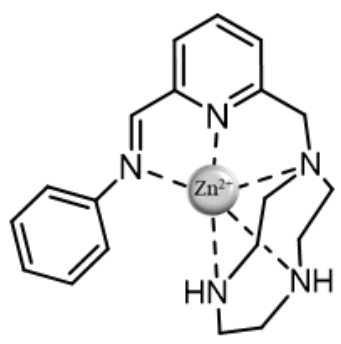

6-B

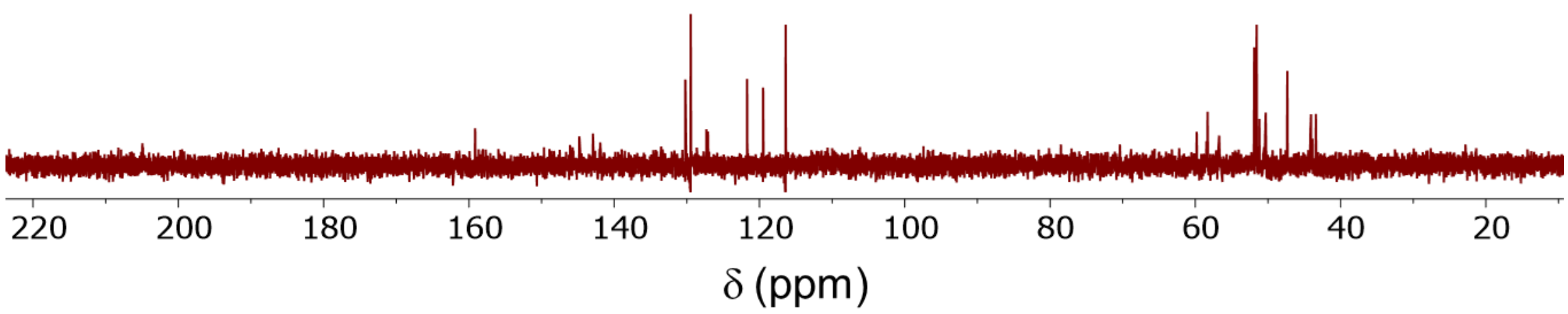

Figure S60. ^{13}C NMR spectrum of imine **6-B**, [**6-B**] = 5 mM, 10 mM HEPES in D_2O and 25 °C.

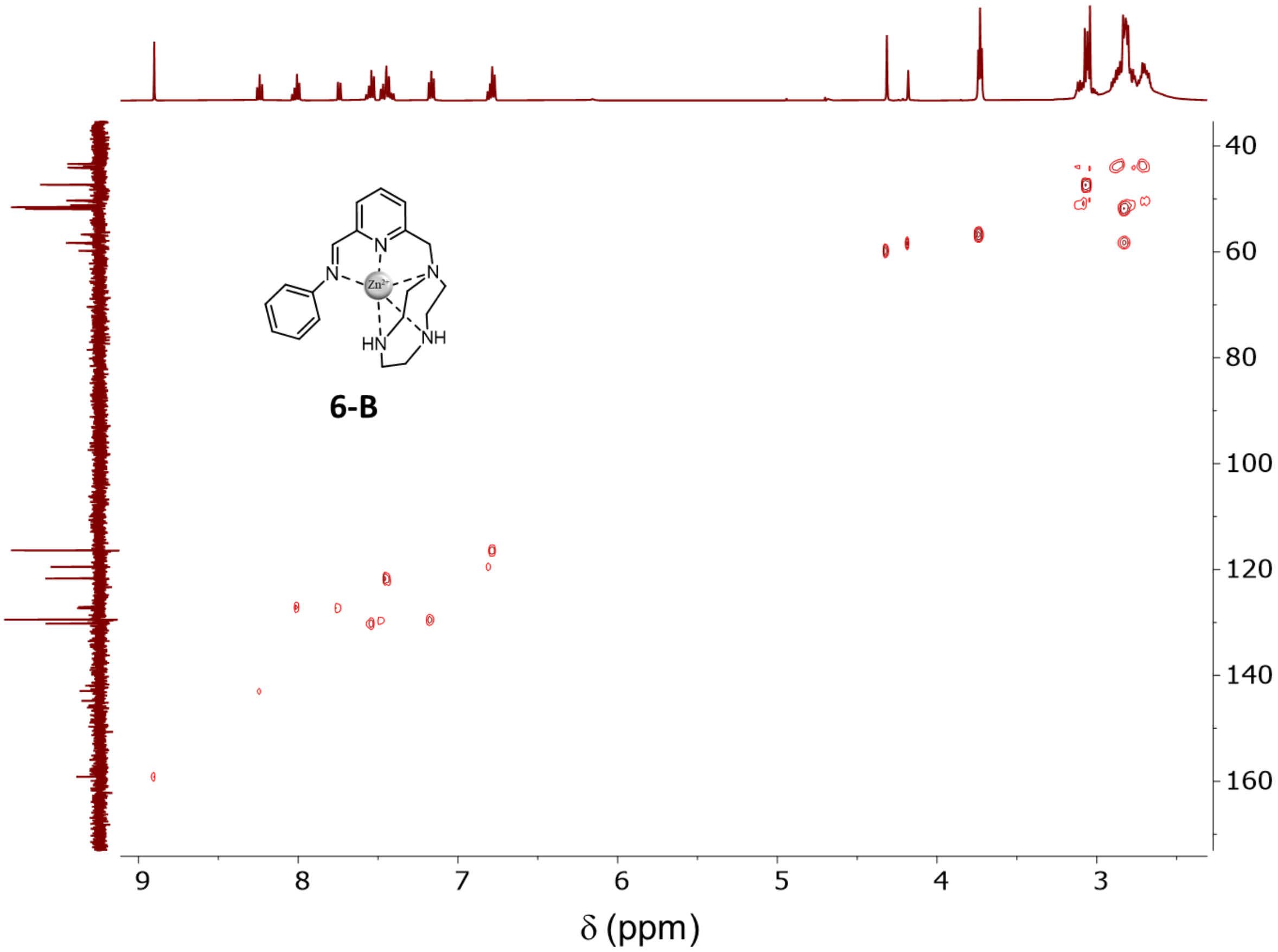

Figure 61. ^{1}H-^{13}C HSQC spectrum of imine **6-B**, [**6-B**] = 5 mM, 10 mM HEPES, D_2O, and 25 °C.

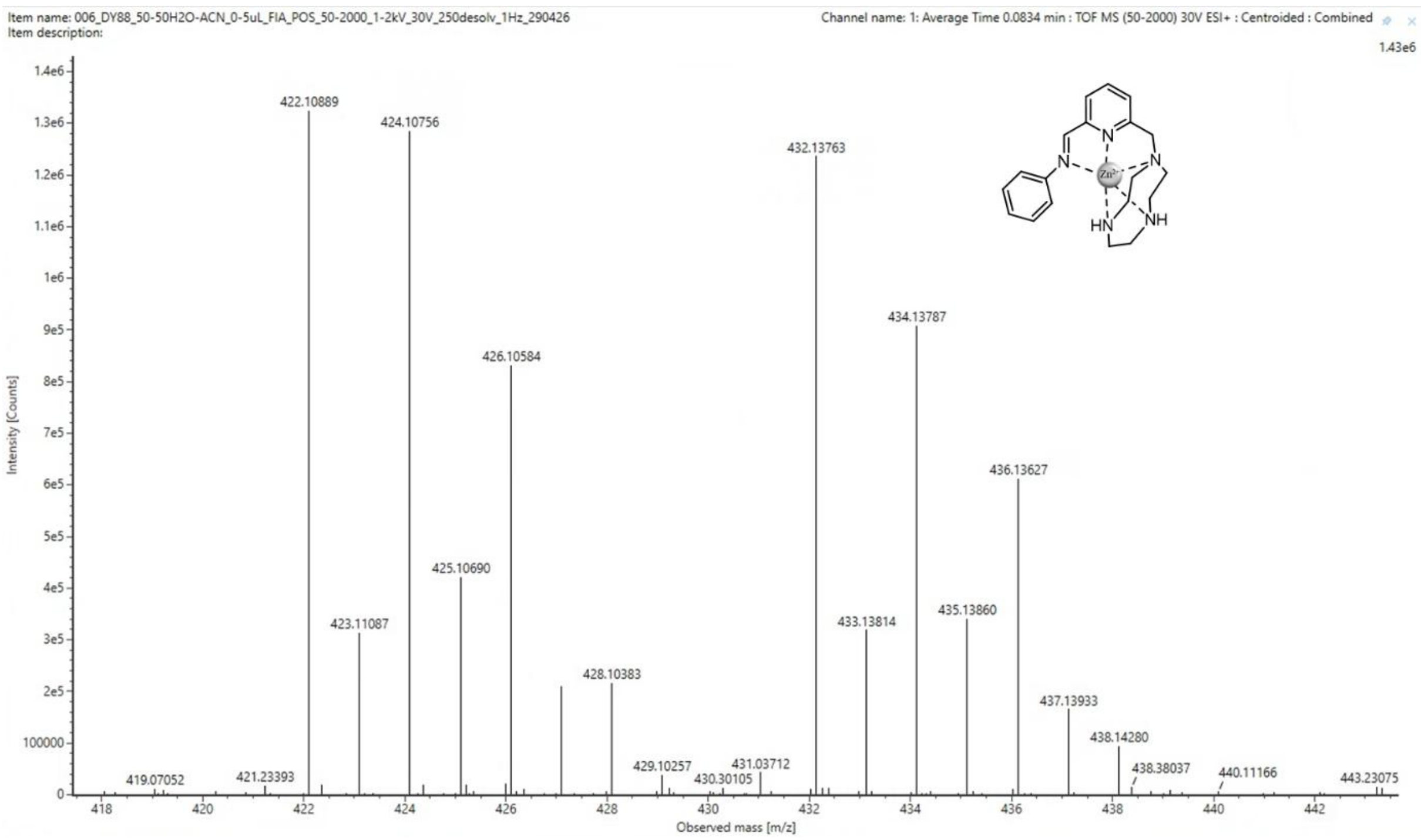

Figure S62. HRMS spectrum of imine **6-B**. Calculated Mass, $[M+Cl^-]$ = 422.1084

S4. Dynamic combinatorial library of imines in water

S4.1. Dynamic imine formation upon the stepwise addition of B, C, and D to aldehyde 6

A dynamic combinatorial library (DCL) of imines was established in aqueous solution by mixing aldehyde **6** (0.5 mM) with an equimolar amount of **D**. Upon reaching equilibrium, **B** (0.5 mM) was added, resulting in redistribution of the imine species (**6-D** and **6-B**) through transimination, and the system evolved to a new equilibrium composition. Subsequently, C (0.5 mM) was introduced, leading to further dynamic exchange and re-equilibration of the library. The system reached a final thermodynamic equilibrium within approximately 4 h. The relative distribution of imines and aldehyde was quantified by integration of the corresponding ^{1}H NMR signals, with the combined integrals normalized to 100%.

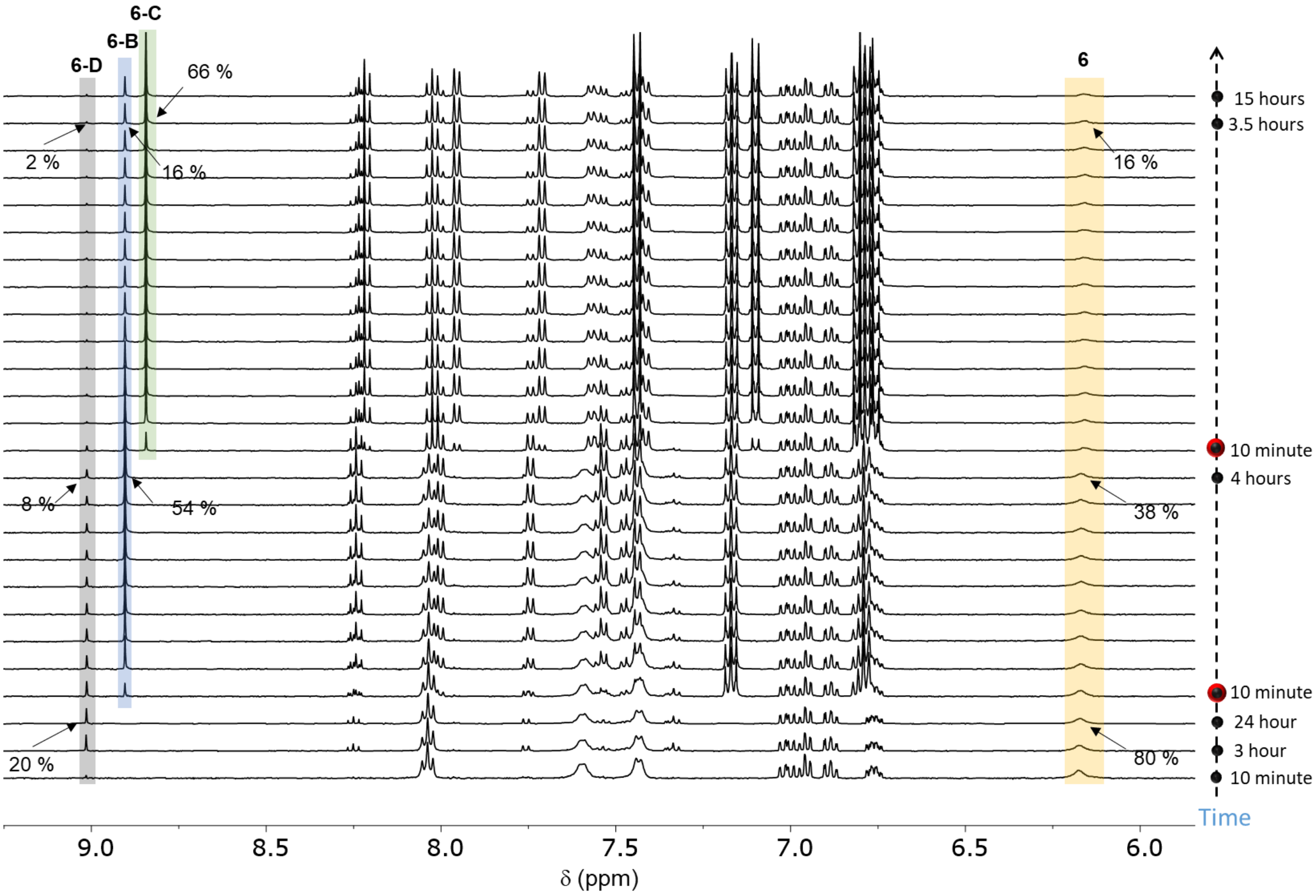

Figure S63. Parts of ^{1}H NMR spectra (500 MHz, D_2O) recorded at the indicated time intervals following sequential addition of amines D, B, and C to aldehyde **6** ([**6**] = [**D**] = [**B**] = [**C**] = 0.5 mM, 10 mM HEPES buffer, pD = 7.4). The grey, blue, and green highlighted regions correspond to imines **6-D, 6-B**, and **6-C,** respectively, while the yellow region indicates the hydrated aldehyde **6**.

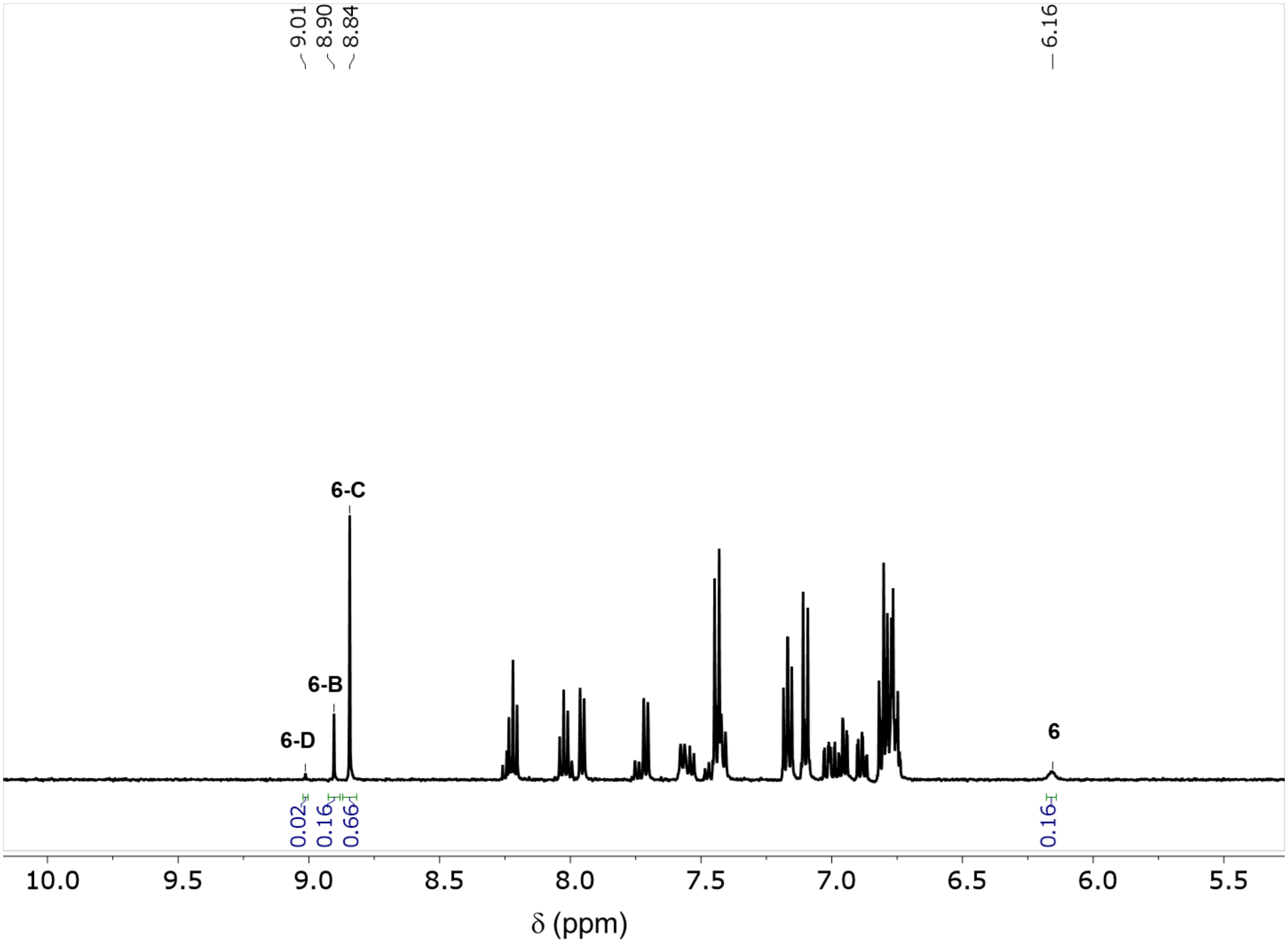

Figure S64. Parts of ^1H NMR spectrum (500 MHz, D_2O) recorded after final equilibration of the dynamic imine library generated via a stepwise approach, illustrating the distribution of imine and aldehyde species at thermodynamic equilibrium.

S4.2. Dynamic imine formation upon the simultaneous addition of B, C, and D to aldehyde 6

The same library (see section S4.1) was also obtained in a one pot approach by combining aldehyde **6** with equimolar amounts of amines **B**, **C**, and **D** at pD 7.4 in D_2O. Thermodynamic equilibrium was reached through a combination of reversible imine formation and transimination, generating a dynamic combinatorial library. In 4 hours, the system reached a thermodynamic equilibrium distribution.

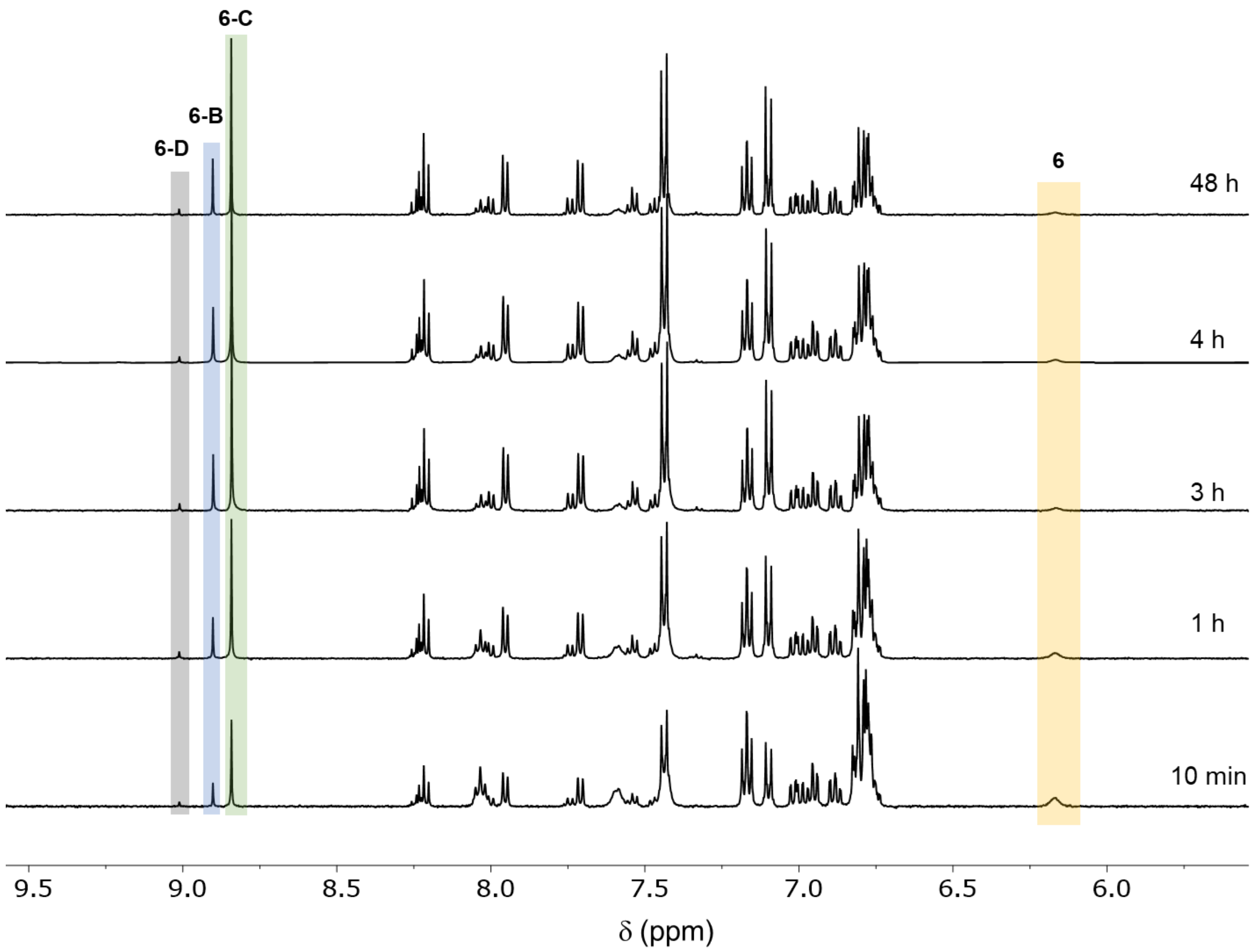

Figure S65. Parts of ^{1}H NMR spectra (500 MHz, D_2O) recorded at the indicated time intervals following one pot addition of **B, C, D,** and 6 ([**6**] = [**D**] = [**B**] = [**C**] = 0.5 mM, 10 mM HEPES buffer, pD = 7.4). The grey, blue, and green highlighted regions correspond to imines **6-D**, **6-B**, and **6-C**, respectively, while the yellow region indicates the hydrated aldehyde **6**.

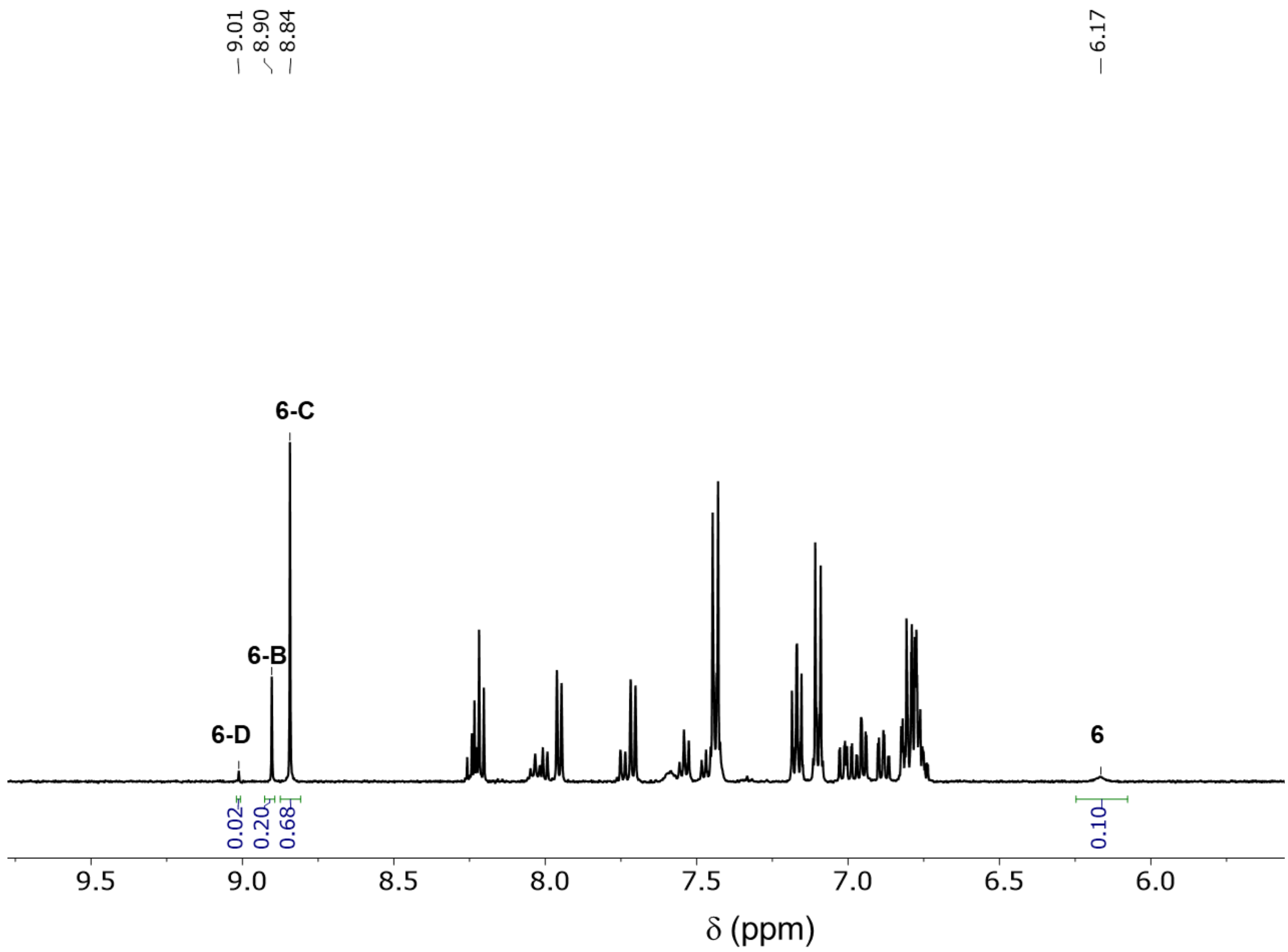

Figure S66. Parts of ^1H NMR spectrum (500 MHz, D_2O) recorded after final equilibration of the dynamic imine library generated via a one pot approach, illustrating the distribution of imine and aldehyde species at thermodynamic equilibrium.

S4.3. Distribution of dynamic combinatorial library composition based on equilibrium constants

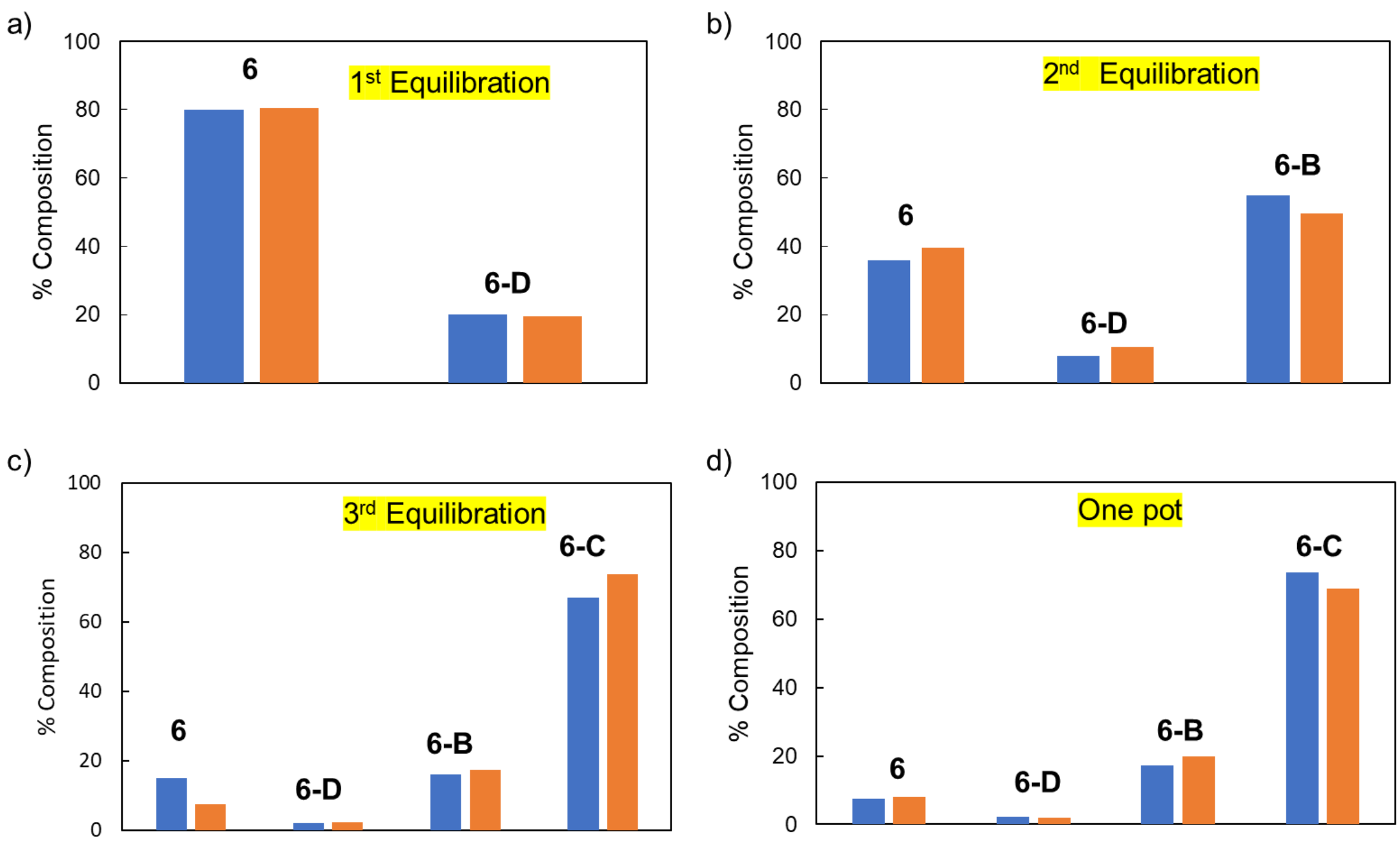

Figure S67. Comparison of the theoretically calculated from the equilibrium constant (orange) and experimentally determined (blue) percentage compositions of the dynamic combinatorial libraries (DCLs), for the stepwise approach (a-c) and the one-pot approach (d).

S4.4. Kinetic analysis of the dynamic exchange reactions regarding imines 6-B, 6-C and 6-D

We exploited the experimentally determined rate constants for formation and hydrolysis of imines **6-B**, **6-C** and **6-D** (Figure 4c) to calculate how the concentration of imines **6-B** and **6-D** would change upon the addition of amine **E** (segment 3 in Figure 5b) if the pathway for the formation of imine **6-C** would involve hydrolysis of imines **6-B** and **6-D** followed by condensation of aldehyde **6** and amine **E**. In this scenario, the rate-limiting step for the formation of imine **6-D** is the hydrolysis of either **6-B** or **6-D**. Comparison with the experimental data shows that the concentrations of **6-B** and **6-D** decrease much faster than permitted based on the hydrolysis rates of **6-B** and **6-D** suggesting that imine **6-C** forms as a result of a transimination reaction.

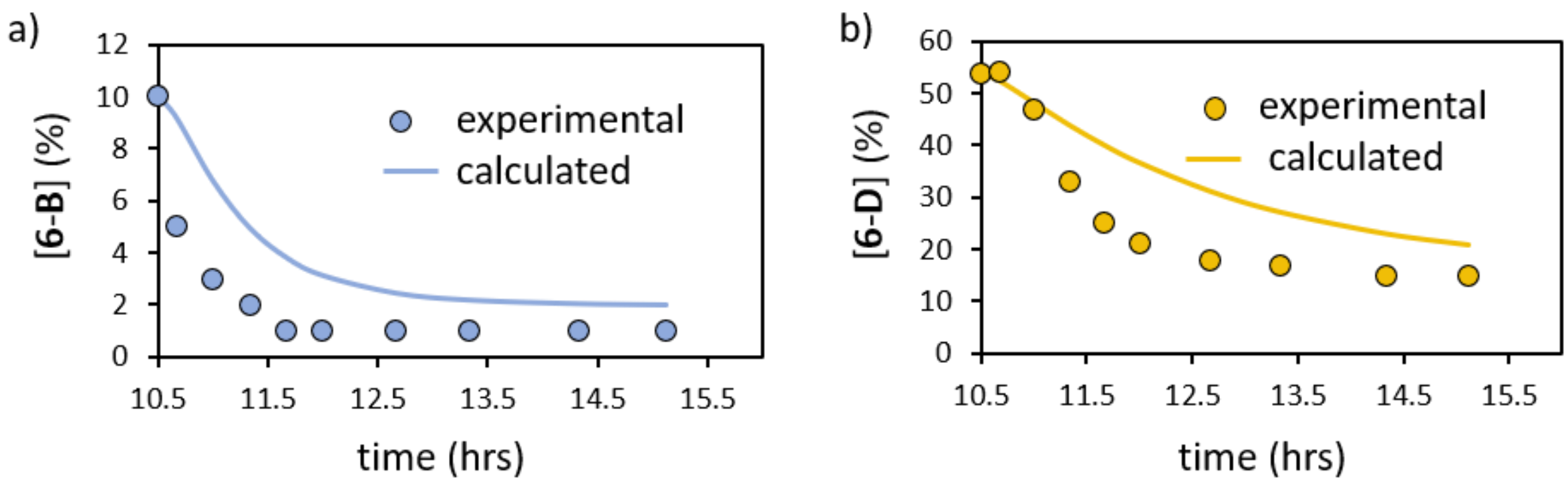

Figure S68. Plots of the changes in concentrations of imines 6-B (a) and 6-D (b) upon adding amine E (corresponding to 3° segment in Fig. 5b). The lines indicate the calculated change in concentration assuming a pathway for the formation of imine 6-C relying on hydrolysis of imines 6-B and 6-D followed by condensation of aldehyde 6 and amine E.

S5. Determination of rate constants and equilibrium constants

Kinetic data were analyzed by non-linear regression using an integrated rate equation describing a reversible second-order reaction between equimolar aldehyde **(1-6)** and amine **(A-E)** reactants. The fitting model applied to the formation of **1-A** is shown below.

$$[1-A]_t = [1]_0 - [1]_t = [1]_0 - \frac{(a-cb)}{(1-c)}$$

where

$$a = \frac{-\frac{1}{K} + \sqrt{\left(\frac{1}{K}\right)^2 + \frac{4[\mathrm{S}]_0}{K}}}{2}, \qquad b = \frac{-\frac{1}{K} - \sqrt{\left(\frac{1}{K}\right)^2 + \frac{4[\mathrm{S}]_0}{K}}}{2}, \qquad c = \frac{([\mathrm{S}]_0 - a)}{([\mathrm{S}]_0 - b)} e^{(-(a-b)k_+ t)}$$

In this equation, K denotes the equilibrium constant and k the forward rate constant for imine condensation. The subscript t indicates the concentration at time t, while subscript 0 denotes the initial concentration (t = 0). The fitted rate and equilibrium constants are reported in Figure 1a, and the corresponding non-linear regression fits for 1-A kinetics are presented in Figure 1b of the main text.

Both reactants are equal: $S_1 + S_2 \rightleftharpoons P$, $[S_1] = [S_2]$

$$= -\frac{d[\mathrm{S}_1]}{dt} = k_+[\mathrm{S}_1][\mathrm{S}_2] - k_-[\mathrm{P}] = k_+[\mathrm{S}_1]^2 - k_-[\mathrm{P}]$$

The difference emerges because of the different stoichiometry between P and S. Here,

$$[\mathrm{P}] = [\mathrm{S}_1]_0 - [\mathrm{S}_1]$$

Following the approach above, the differential can be rearranged as

$$\frac{1}{[\mathrm{S}_1]^2 - \frac{k_-}{k_+}([\mathrm{S}_1]_0 - [\mathrm{S}_1])} d[\mathrm{S}_1] = \frac{1}{[\mathrm{S}_1]^2 + \frac{1}{K}[\mathrm{S}_1] - \frac{1}{K}[\mathrm{S}_1]_0} d[\mathrm{S}_1] = -k_+ dt$$

And the roots of the (slightly different) quadratic can be found in the same way:

$$[\mathrm{S}] = \frac{-\frac{1}{K} \pm \sqrt{\left(\frac{1}{K}\right)^2 + \frac{4[\mathrm{S}]_0}{K}}}{2} = a \text{ or } b$$

Which will be denoted as *a* and *b*.

From there, the rearrangement follows identically to the case above

$$\frac{1}{([\mathrm{S}]-a)([\mathrm{S}]-b)} d[\mathrm{S}] = -k_+ dt$$

$$\frac{1}{a-b}\int_{[\mathrm{S}]_0}^{[\mathrm{S}]_t} \frac{1}{([\mathrm{S}]-a)} - \frac{1}{([\mathrm{S}]-b)} d[\mathrm{S}] = -k_+ \int_0^t dt$$

$$\frac{1}{a-b}\left[\ln\left(\frac{[\mathrm{S}]-a}{[\mathrm{S}]-b}\right)\right]_{[\mathrm{S}]_t}^{[\mathrm{S}]_0} = -k_+[t]_t^0$$

$$\frac{1}{a-b}\left(\ln\frac{([\mathrm{S}]_t-a)([\mathrm{S}]_0-b)}{([\mathrm{S}]_t-b)([\mathrm{S}]_0-a)}\right) = -k_+ t$$

$$\frac{([\mathrm{S}]_t - a)}{([\mathrm{S}]_t - b)}\frac{([\mathrm{S}]_0 - b)}{([\mathrm{S}]_0 - a)} = e^{(-(a-b)k_+t)}$$

$$\frac{([\mathrm{S}]_t - a)}{([\mathrm{S}]_t - b)} = \frac{([\mathrm{S}]_0 - a)}{([\mathrm{S}]_0 - b)}e^{(-(a-b)k_+t)} = c$$

$$[\mathrm{S}]_t - a = c([\mathrm{S}]_t - b)$$

$$[\mathrm{S}]_t - c[\mathrm{S}]_t = a - cb$$

$$\boxed{[\mathrm{S}]_t = \frac{(a - cb)}{(1 - c)}}$$

When both reactants are not equimolar

$S_1 + S_2 \rightleftharpoons P$, $[S_1] \neq [S_2]$

$$-\frac{d[\mathrm{S}_1]}{dt} = k_+[\mathrm{S}_1][\mathrm{S}_2] - k_-[\mathrm{P}]$$

Now, both $[S_2]$ and [P] need to be defined in terms of $[S_1]$.

$$[\mathrm{S}_1] + x\ = [\mathrm{S}_2] \text{ and } [\mathrm{P}] = [\mathrm{S}_1]_0 - [\mathrm{S}_1]$$

Hence

$$-\frac{d[\mathrm{S}_1]}{dt} = k_+[\mathrm{S}_1]([\mathrm{S}_1] + x\ ) - k_-([\mathrm{S}_1]_0 - [\mathrm{S}_1])$$

This can be rearranged to find the polynomial and hence the roots before taking the same approach used for the reversible reactions above.

$$\frac{1}{[\mathrm{S}_1]^2 + x[\mathrm{S}_1] + \frac{k_-}{k_+}[\mathrm{S}_1] - \frac{k_-}{k_+}[\mathrm{S}_1]_0}d[\mathrm{S}_1] = \frac{1}{[\mathrm{S}_1]^2 + \left(x + \frac{1}{K}\right)[\mathrm{S}_1] - \frac{1}{K}[\mathrm{S}_1]_0}d[\mathrm{S}_1] - k_+dt$$

And the roots of the (yet again, slightly different) quadratic can be found in the same way:

$$[\mathrm{S}] = \frac{-\left(x + \frac{1}{K}\right) \pm \sqrt{\left(x + \frac{1}{K}\right)^2 + \frac{4[\mathrm{S}]_0}{K}}}{2} = a \text{ or } b$$

Which will be denoted as *a* and *b*.

From there, the rearrangement follows identically to the cases above

$$\frac{1}{([\mathrm{S}] - a)([\mathrm{S}] - b)}d[\mathrm{S}] = -k_+dt$$

$$\frac{1}{a - b}\int_{[\mathrm{S}]_0}^{[\mathrm{S}]_t} \frac{1}{([\mathrm{S}] - a)} - \frac{1}{([\mathrm{S}] - b)}d[\mathrm{S}] = -k_+\int_0^t dt$$

$$\frac{1}{a - b}\left[\ln\left(\frac{[\mathrm{S}] - a}{[\mathrm{S}] - b}\right)\right]_{[\mathrm{S}]_t}^{[\mathrm{S}]_0} = -k_+[t]_t^0$$

$$\frac{1}{a-b}\left(\ln\frac{([\mathrm{S}]_t - a)([\mathrm{S}]_0 - b)}{([\mathrm{S}]_t - b)([\mathrm{S}]_0 - a)}\right) = -k_+ t$$

$$\frac{([\mathrm{S}]_t - a)}{([\mathrm{S}]_t - b)}\frac{([\mathrm{S}]_0 - b)}{([\mathrm{S}]_0 - a)} = e^{(-(a-b)k_+ t)}$$

$$\frac{([\mathrm{S}]_t - a)}{([\mathrm{S}]_t - b)} = \frac{([\mathrm{S}]_0 - a)}{([\mathrm{S}]_0 - b)}e^{(-(a-b)k_+ t)} = c$$

$$[\mathrm{S}]_t - a = c([\mathrm{S}]_t - b)$$

$$[\mathrm{S}]_t - c[\mathrm{S}]_t = a - cb$$

$$\boxed{[\mathrm{S}]_t = \frac{(a-cb)}{(1-c)}}$$

Where

$$a = \frac{-\left(x+\frac{1}{K}\right)+\sqrt{\left(x+\frac{1}{K}\right)^2+\frac{4[\mathrm{S}]_0}{K}}}{2}, \qquad b = \frac{-\left(x+\frac{1}{K}\right)-\sqrt{\left(x+\frac{1}{K}\right)^2+\frac{4[\mathrm{S}]_0}{K}}}{2},$$

$$c = \frac{([\mathrm{S}]_0 - a)}{([\mathrm{S}]_0 - b)}e^{(-(a-b)k_+ t)}$$

S6. DFT optimized geometry of 1-A and 6-B

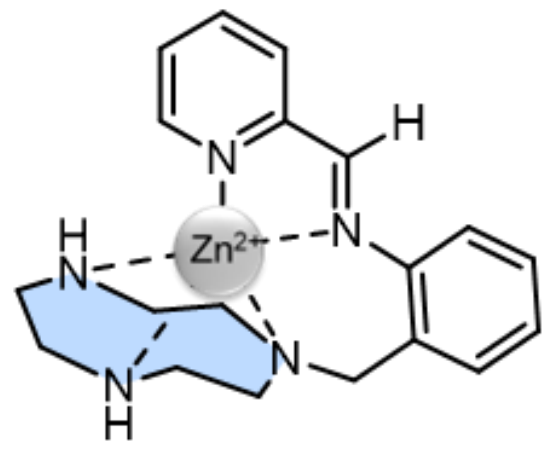

1-A

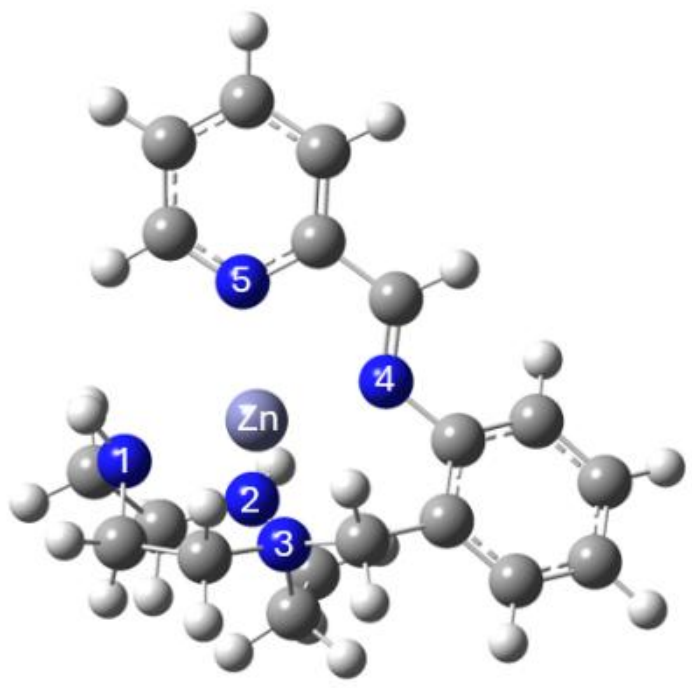

r(Zn-N1) = 2.12 Å

r(Zn-N2) = 2.08 Å

r(Zn-N3) = 2.18 Å

r(Zn-N4) = 2.10 Å

r(Zn-N5) = 2.12 Å

Optimized structure wb97xd/6-31g(d,p). E = -2791.5309

C	-1.733491000	-2.921799000	-0.883696000
N	-1.484340000	-2.280766000	0.425808000
C	-0.587284000	-3.055318000	1.316609000
C	0.471457000	-2.158879000	1.952050000
H	-0.123550000	-3.858180000	0.740370000
H	-1.160315000	-3.539117000	2.110138000
N	1.127670000	-1.279310000	0.961307000
H	1.206147000	-2.778207000	2.482189000
H	-0.008140000	-1.507863000	2.688504000
C	1.943209000	-2.032799000	-0.024025000
C	1.642198000	-1.610067000	-1.463341000
H	1.756459000	-3.100805000	0.099521000
H	3.010377000	-1.888254000	0.168085000
N	0.184192000	-1.552405000	-1.709677000
H	2.133248000	-2.303636000	-2.153822000
H	2.040976000	-0.611251000	-1.653831000
C	-0.479900000	-2.879928000	-1.761809000
H	-0.757276000	-3.125159000	-2.788443000
H	0.234746000	-3.641425000	-1.442878000
H	-2.539115000	-2.364535000	-1.369531000
H	-2.064667000	-3.959017000	-0.770601000
H	0.031245000	-1.070988000	-2.590374000
H	-2.373279000	-2.155661000	0.899010000
C	1.929857000	-0.254386000	1.669521000
H	2.716855000	-0.742103000	2.258579000
H	1.261587000	0.252740000	2.371899000
Zn	-0.575588000	-0.445505000	-0.120478000
C	2.555282000	0.760742000	0.744592000
C	1.756338000	1.622563000	-0.022661000
C	3.937691000	0.901476000	0.666290000
C	2.326614000	2.578840000	-0.859938000
C	4.517235000	1.862339000	-0.160327000
H	4.569763000	0.250038000	1.261887000
C	3.711473000	2.698715000	-0.924208000
H	5.596834000	1.949066000	-0.211755000
H	4.155380000	3.439268000	-1.580100000

N	0.354566000	1.435098000	0.029749000
H	1.690224000	3.202762000	-1.478834000
C	-0.450870000	2.421212000	0.111920000
H	-0.109959000	3.453029000	0.199652000
C	-1.896984000	2.149749000	0.107383000
C	-2.829300000	3.165278000	0.247763000
C	-4.182316000	2.830296000	0.242946000
H	-2.503358000	4.192518000	0.361506000
C	-3.541598000	0.542829000	-0.045354000
C	-4.544109000	1.500489000	0.090861000
H	-4.937009000	3.599602000	0.355715000
H	-3.791973000	-0.504345000	-0.171562000
H	-5.582583000	1.194974000	0.076889000
N	-2.247515000	0.856468000	-0.032234000

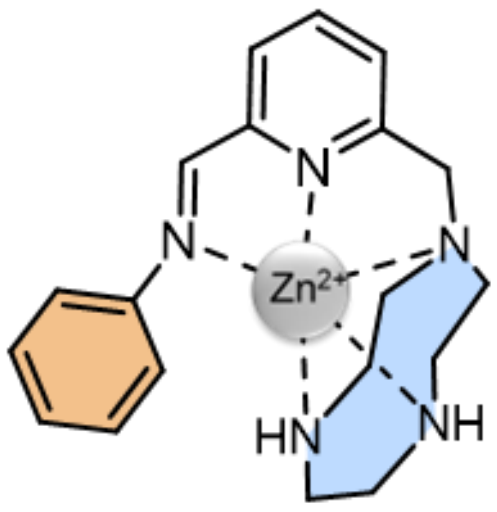

6-B

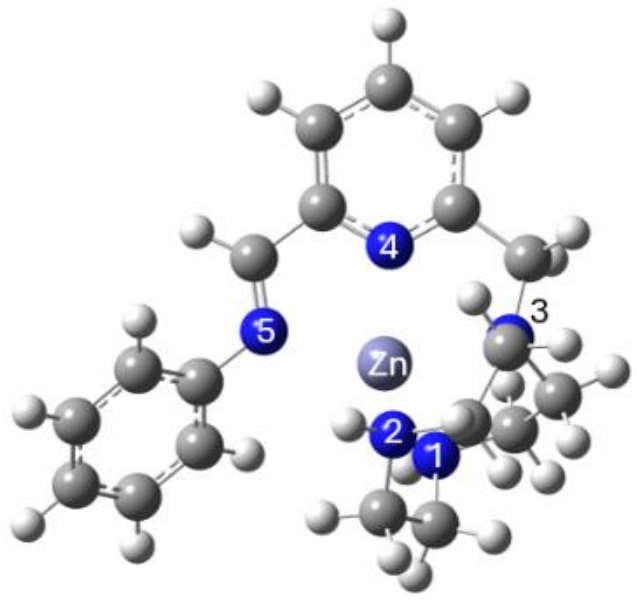

r(Zn-N1) = 2.07 Å

r(Zn-N2) = 2.08 Å

r(Zn-N3) = 2.27 Å

r(Zn-N4) = 2.04 Å

r(Zn-N5) = 2.16 Å

Optimized structure wb97xd/6-31g(d,p). E = -2791.5313

C	-0.817955000	-2.669519000	0.857729000
N	-0.058410000	-1.535139000	1.429417000
C	1.216910000	-1.918429000	2.088840000
C	2.381235000	-1.026275000	1.654006000
H	1.426744000	-2.965413000	1.860746000
H	1.113870000	-1.854346000	3.173607000
N	2.450102000	-0.875035000	0.190610000
H	3.316779000	-1.427756000	2.064281000
H	2.240790000	-0.026150000	2.076409000
C	2.740604000	-2.119904000	-0.541372000
C	1.773245000	-2.295757000	-1.712193000
H	2.664283000	-2.964322000	0.145079000
H	3.767842000	-2.135534000	-0.917436000
N	0.367066000	-2.124594000	-1.274959000
H	1.925863000	-3.278180000	-2.170438000
H	1.965023000	-1.534259000	-2.472748000
C	-0.131162000	-3.216819000	-0.397064000
H	-0.837330000	-3.846408000	-0.940716000
H	0.708616000	-3.857776000	-0.122619000
H	-1.809884000	-2.287333000	0.599766000

H	-0.946576000	-3.476061000	1.586214000
H	-0.211727000	-2.082071000	-2.108137000
H	-0.651519000	-1.069734000	2.109540000
Zn	0.283144000	-0.326773000	-0.217626000
C	3.307410000	0.242985000	-0.212059000
C	2.562346000	1.559678000	-0.160618000
C	3.191231000	2.804535000	-0.074317000
C	2.408391000	3.948633000	-0.057996000
H	4.271482000	2.864366000	-0.014319000
C	0.470600000	2.572545000	-0.181515000
C	1.015361000	3.841176000	-0.106711000
H	0.378294000	4.716505000	-0.071902000
H	2.873895000	4.925013000	0.008657000
N	1.241472000	1.476355000	-0.225402000
C	-0.975737000	2.292695000	-0.173872000
H	-1.673790000	3.129267000	-0.140452000
H	4.218281000	0.301621000	0.396180000
H	3.624067000	0.086225000	-1.247621000
N	-1.365388000	1.074836000	-0.200816000
C	-2.748684000	0.763024000	-0.127512000
C	-3.585522000	1.390253000	0.797089000
C	-3.250348000	-0.221626000	-0.980234000
C	-4.931219000	1.040868000	0.849088000
H	-3.180912000	2.122611000	1.488153000
C	-4.598124000	-0.556145000	-0.927203000
H	-2.590838000	-0.697141000	-1.698482000
C	-5.440648000	0.073069000	-0.012677000
H	-5.579974000	1.522639000	1.572459000
H	-4.989000000	-1.310723000	-1.600994000
H	-6.490089000	-0.196740000	0.033154000

S7. Synthesis of compound A and 6

Compound **A** and **di-Boc-TACN** were synthesized according to previously reported procedures.[3]

S7.1. Synthesis of compound 6

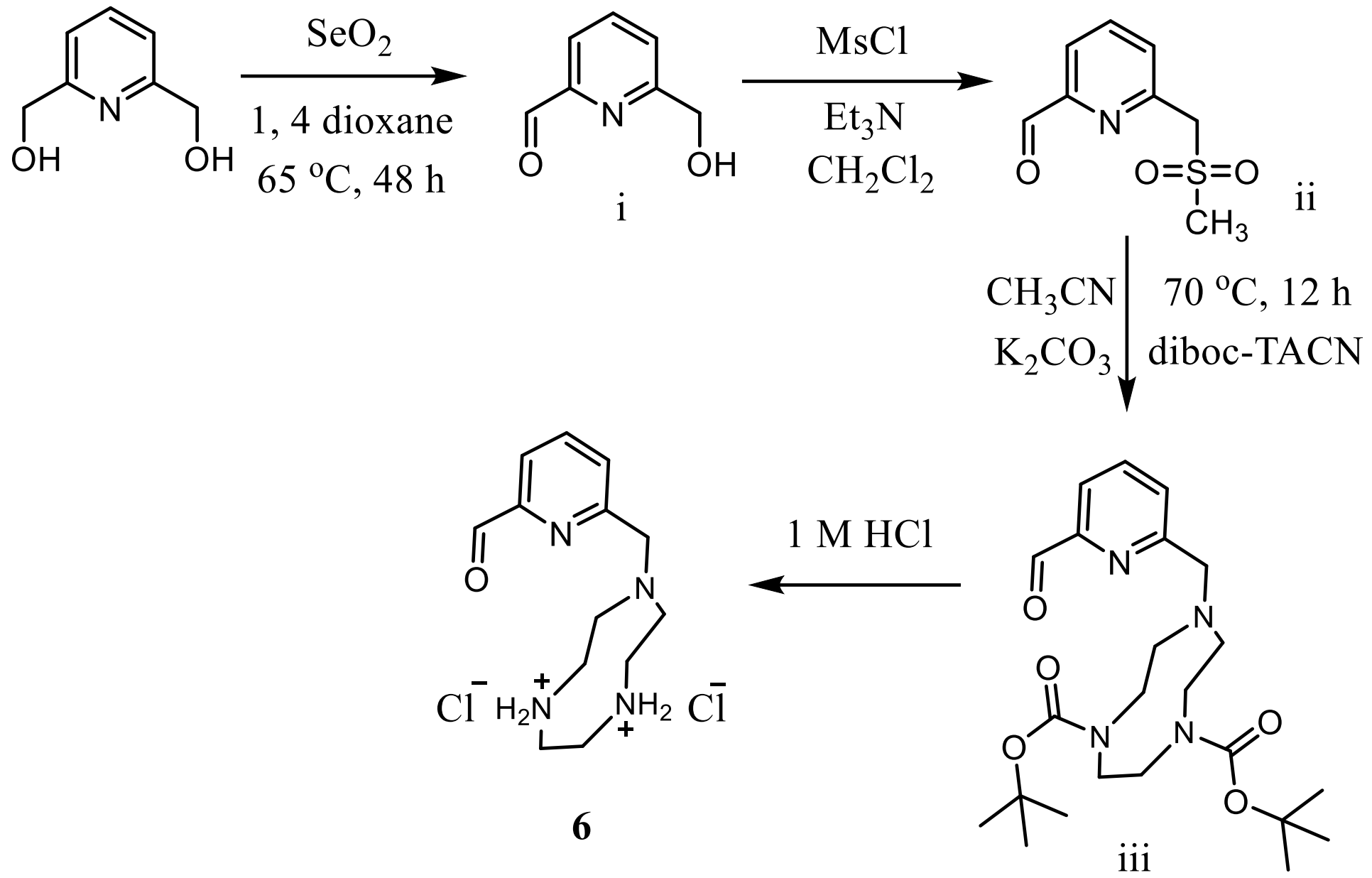

Preparation of compound i[4]

2,6-Pyridinedimethanol (3.75 g, 27.0 mmol) was dissolved in 1,4-dioxane (75 mL) under stirring. Selenium dioxide (1.50 g, 13.5 mmol, 0.5 eq) was added to the solution, and the reaction mixture was sonicated for 5 minutes to ensure complete dispersion. The mixture was then stirred at 65 °C for 24 h. After completion, the reaction was allowed to cool to room temperature and diluted with dichloromethane (125 mL). The mixture was filtered through a plug of Celite to remove insoluble materials, and the filtrate was concentrated under reduced pressure. The crude product was purified by column chromatography (1: 1 ethyl acetate: DCM) to afford the desired product as an off-white solid (2.8 g, 76%).

^{1}H NMR (500 MHz, $CDCl_3$): δ 10.1 (s, 1H), 7.92–7.85 (m, 2H), 7.57–7.51 (m, 1H), 4.90 (s, 2H)

^{13}C NMR (125 MHz, $CDCl_3$): δ 193.2, 160.1, 151.7, 137.7, 125.1, 120.6, 64.1.

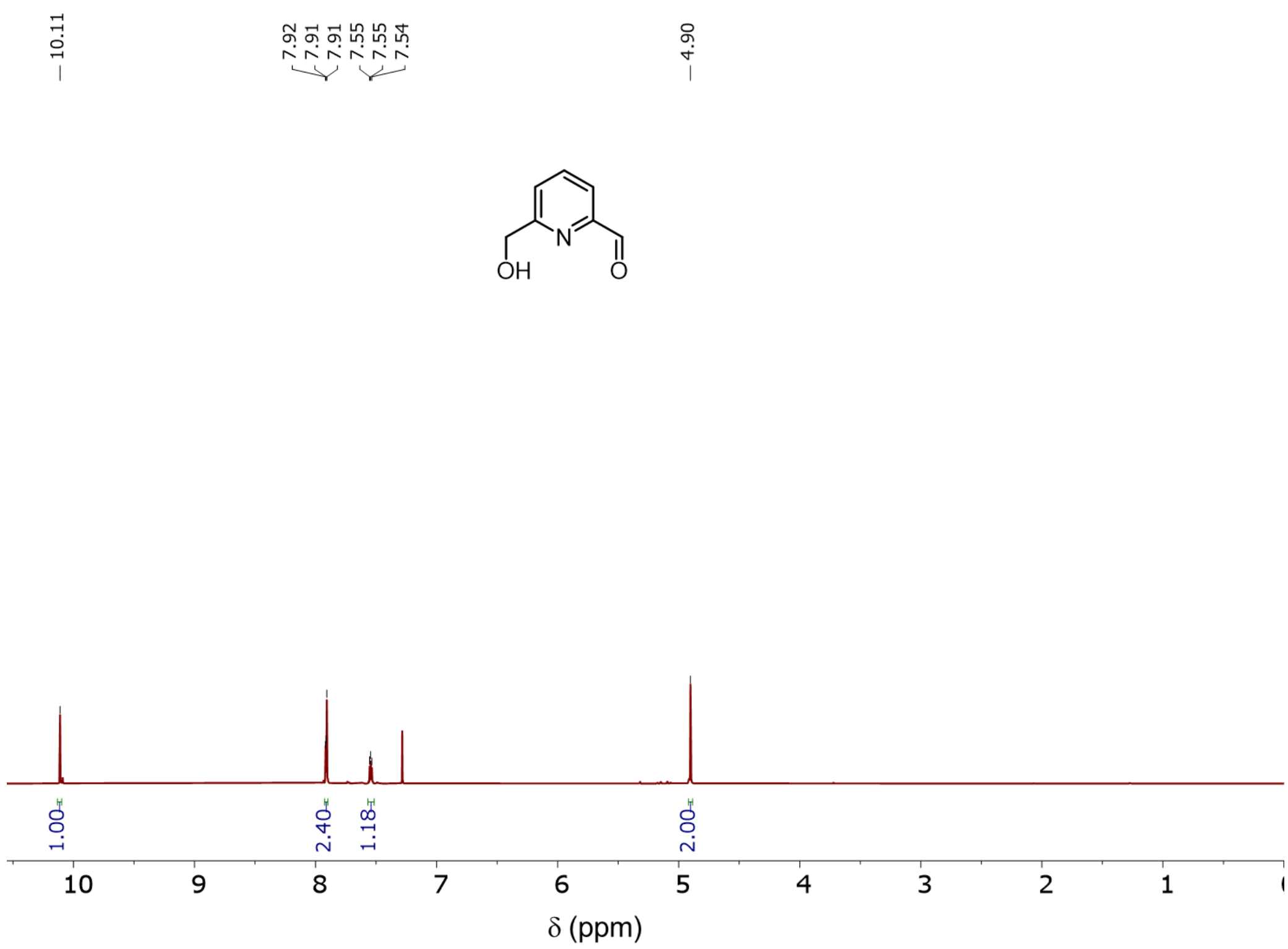

Figure S69. ^{1}H NMR spectrum of compound **i** in $CDCl_3$.

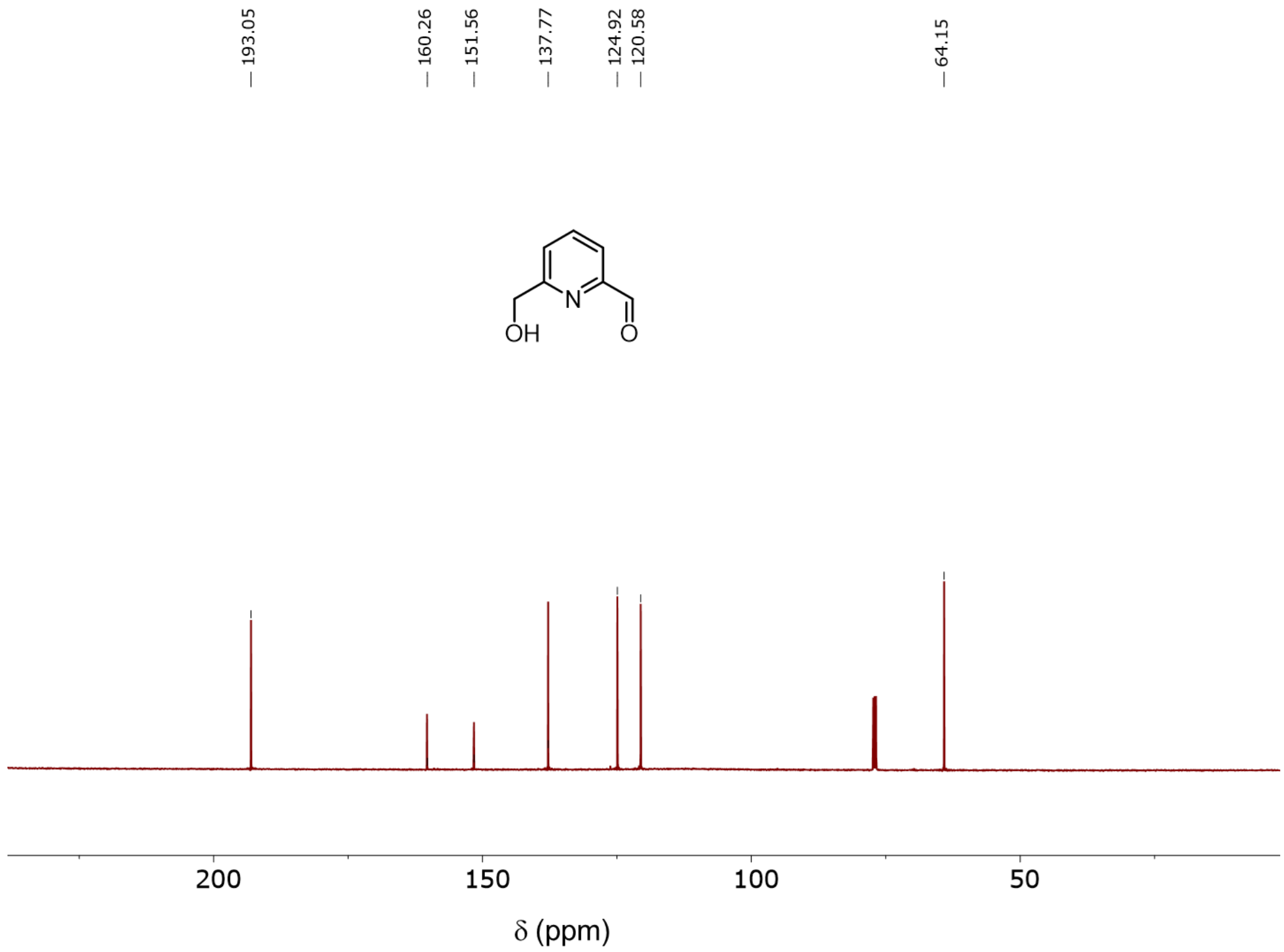

Figure S70. ^{13}C NMR spectrum of compound **i** in $CDCl_3$.

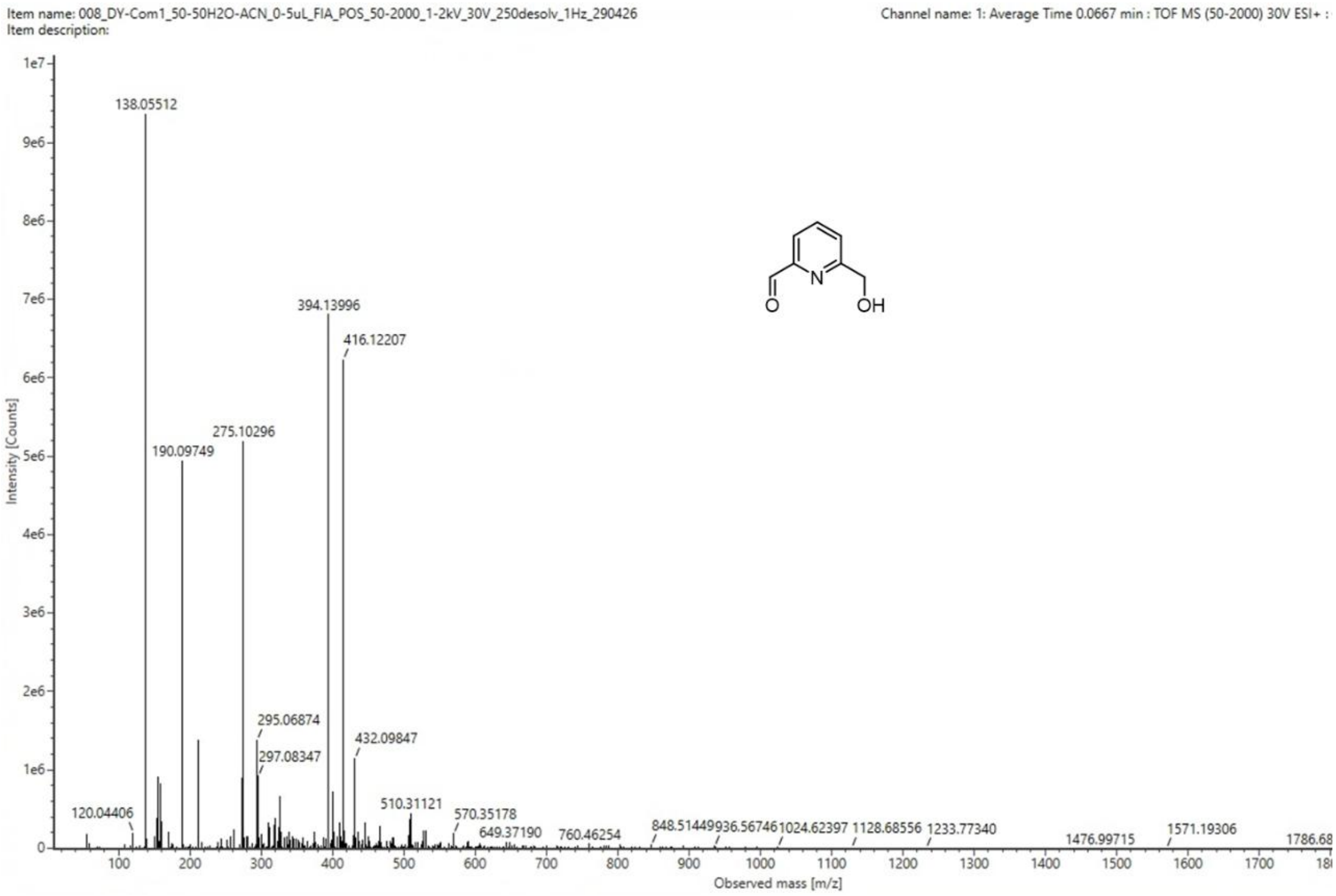

Figure S71. HRMS spectrum of compound **i,** Calculated mass [M+H] = 138.055

<u>Preparation of compound ii</u>[4]

Methanesulfonyl chloride (0.69 mL, 8.75 mmol, 1.2 equiv) was added dropwise at 0 °C to a stirred solution of 6-(hydroxymethyl)-2-pyridinecarboxaldehyde (1.0 g, 7.29 mmol, 1.0 equiv) and triethylamine (2.07 mL, 21.9 mmol, 3.0 equiv) in dry dichloromethane (30 mL). The reaction mixture was stirred at 0 °C for 1 h. The reaction was quenched by the addition of saturated aqueous sodium bicarbonate solution. The aqueous layer was separated and extracted with dichloromethane (3 × 20 mL). The combined organic layers were dried over anhydrous sodium sulfate, filtered, and concentrated under reduced pressure to afford the crude product. The product was obtained as a brown oil (1.2 g, 82 % yield).

^{1}H NMR (500 MHz, $CDCl_3$): δ 10.05 (s, 1H), 7.99–7.96 (m, 2H), 7.74–7.72 (m, 1H), 5.40 (s, 2H), 3.13 (s, 3H).
^{13}C NMR (125 MHz, $CDCl_3$): δ 192.6, 154.5, 152.6, 138.6, 126.4, 121.9, 70.6, 38.1.

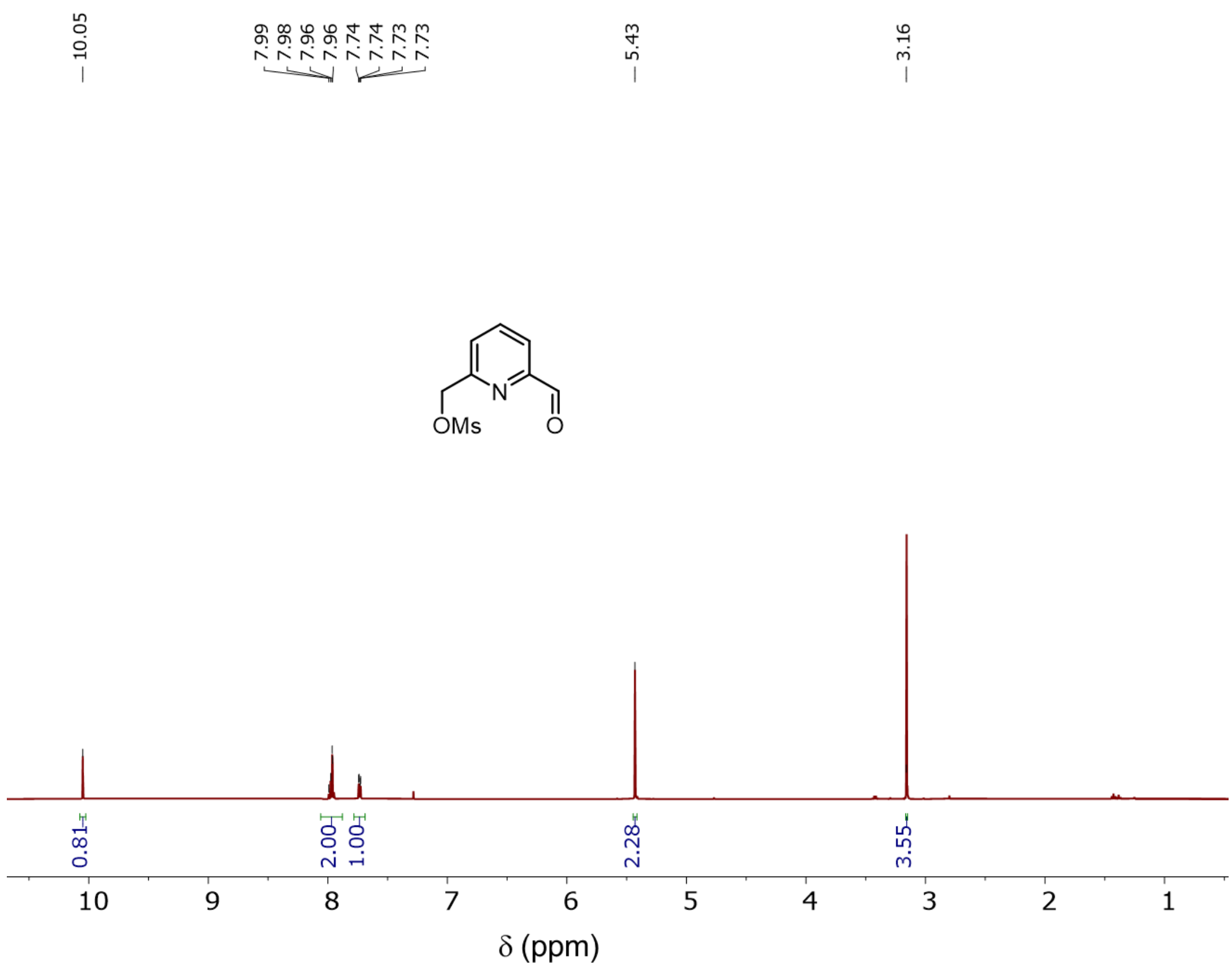

Figure S72. ^{1}H NMR spectrum of compound **ii** in $CDCl_3$.

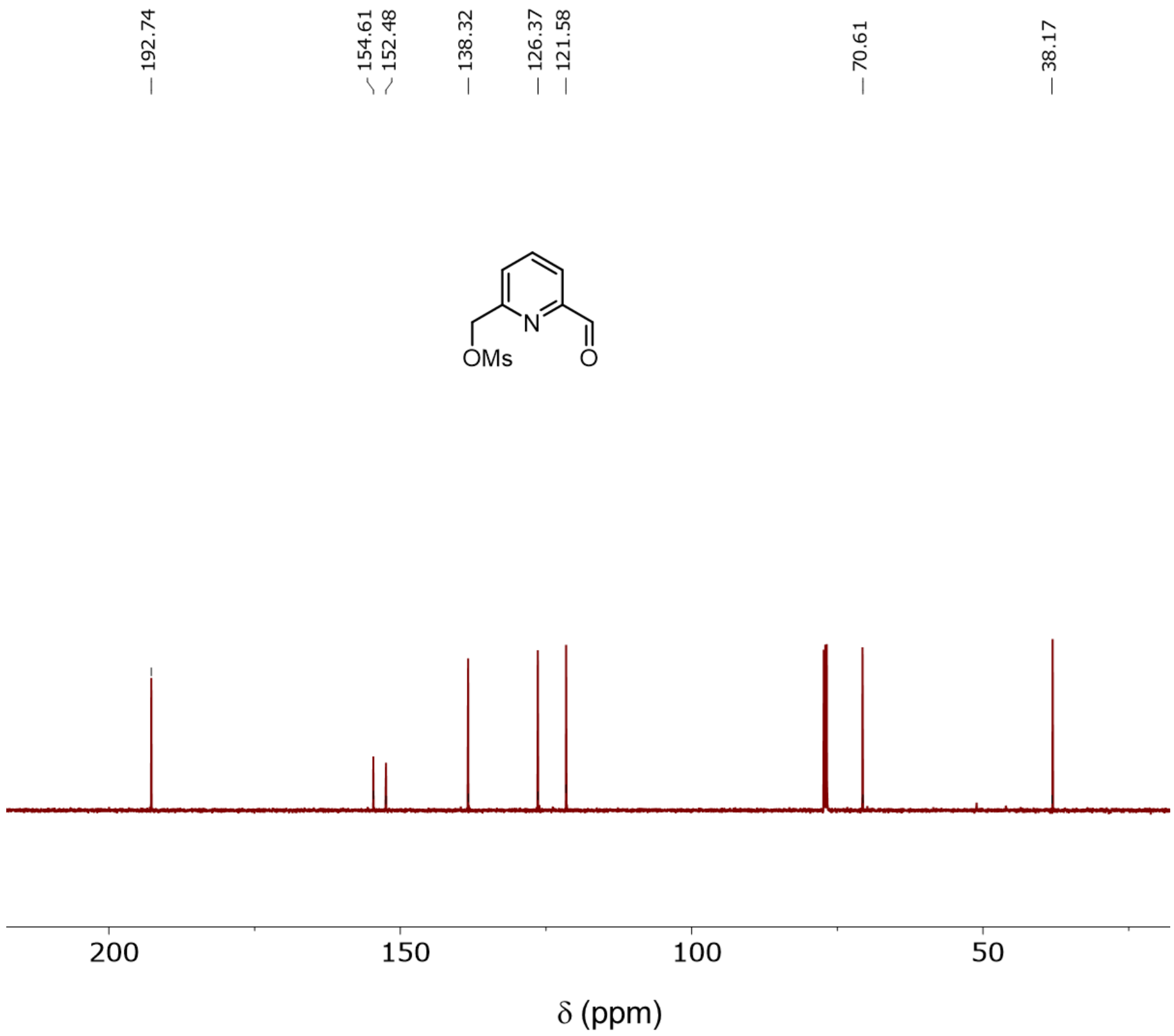

Figure S73. ^{13}C NMR spectrum of compound **ii** in $CDCl_3$.

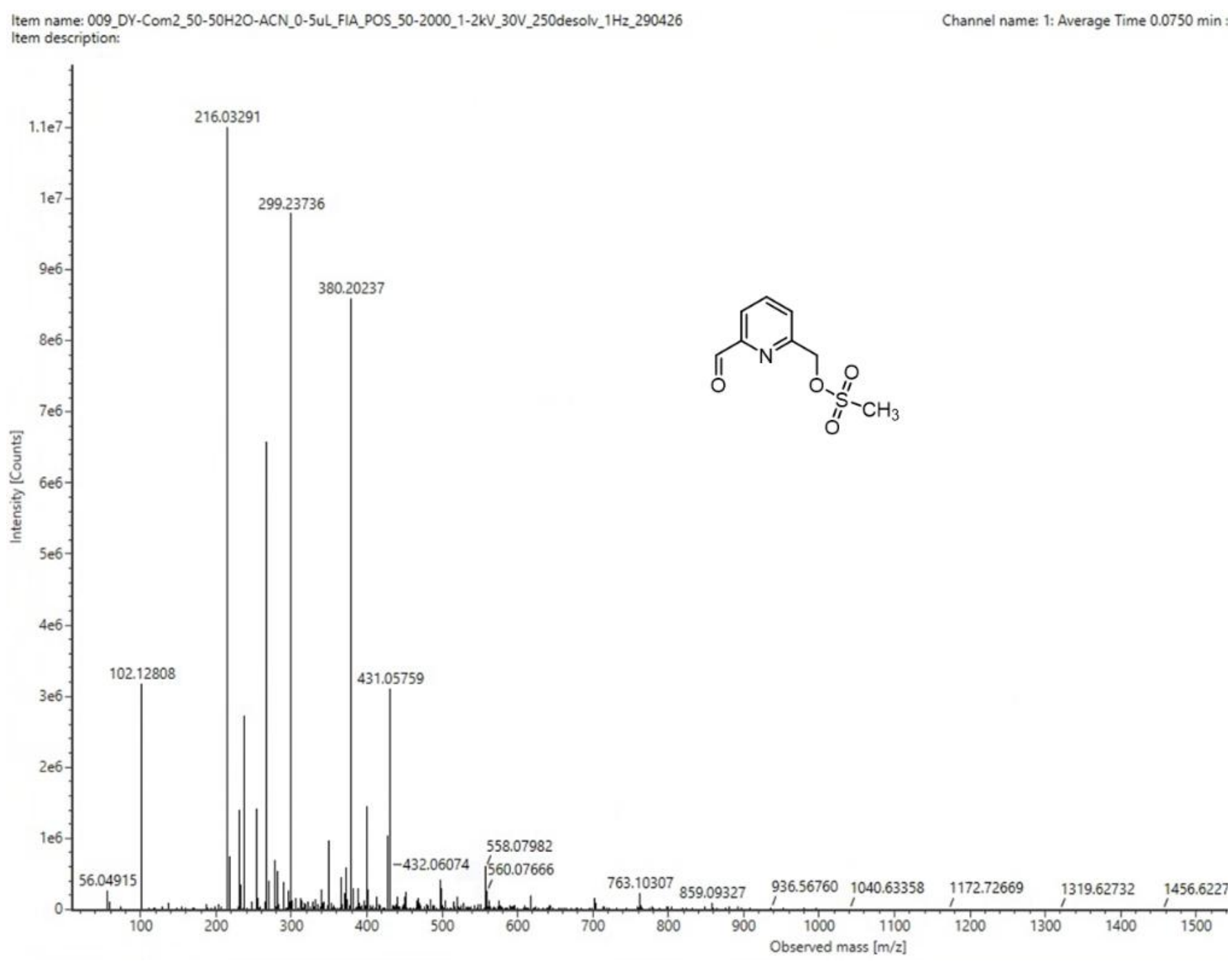

Figure S74. HRMS spectra of compound **ii**, Calculated mass [M+H] = 216.033

Preparation of compound iii

To a stirred solution of **compound ii** (0.25 g, 1.15 mmol, 1.0 equiv) in dry acetonitrile (30 mL) was added di-Boc-protected 1,4,7-triazacyclononane (0.42 g, 0.65 mmol, 1.1 equiv), followed by potassium carbonate (0.4 g, 2.6 mmol, 2.9 equiv). The reaction mixture was stirred at 60 °C for 12 h under an inert atmosphere. After completion, the solvent was removed under reduced pressure. The residue was washed with saturated aqueous sodium bicarbonate solution and extracted with dichloromethane (3 × 50 mL). The combined organic layers were dried over anhydrous sodium sulfate and concentrated under reduced pressure. Purification by flash column chromatography afforded the desired product as a viscous brown solid (0.37 g, 70% yield).

^{1}H NMR (500 MHz, $CDCl_3$): δ 10.03 (s, 1H), 7.72–7.90 (m, 3H), 3.48 (s, 1H), 3.1–3.45 (m, 8H), 2.71–2.23 (m, 2H), 1.47–1.51 (d, 18H)
^{13}C NMR (125 MHz, $CDCl_3$): δ 193.6, 155.8, 151.8, 137.1, 127.9, 125.3, 120.3, 79.6, 61.9, 53.2, 50.6, 49.1, 28.6

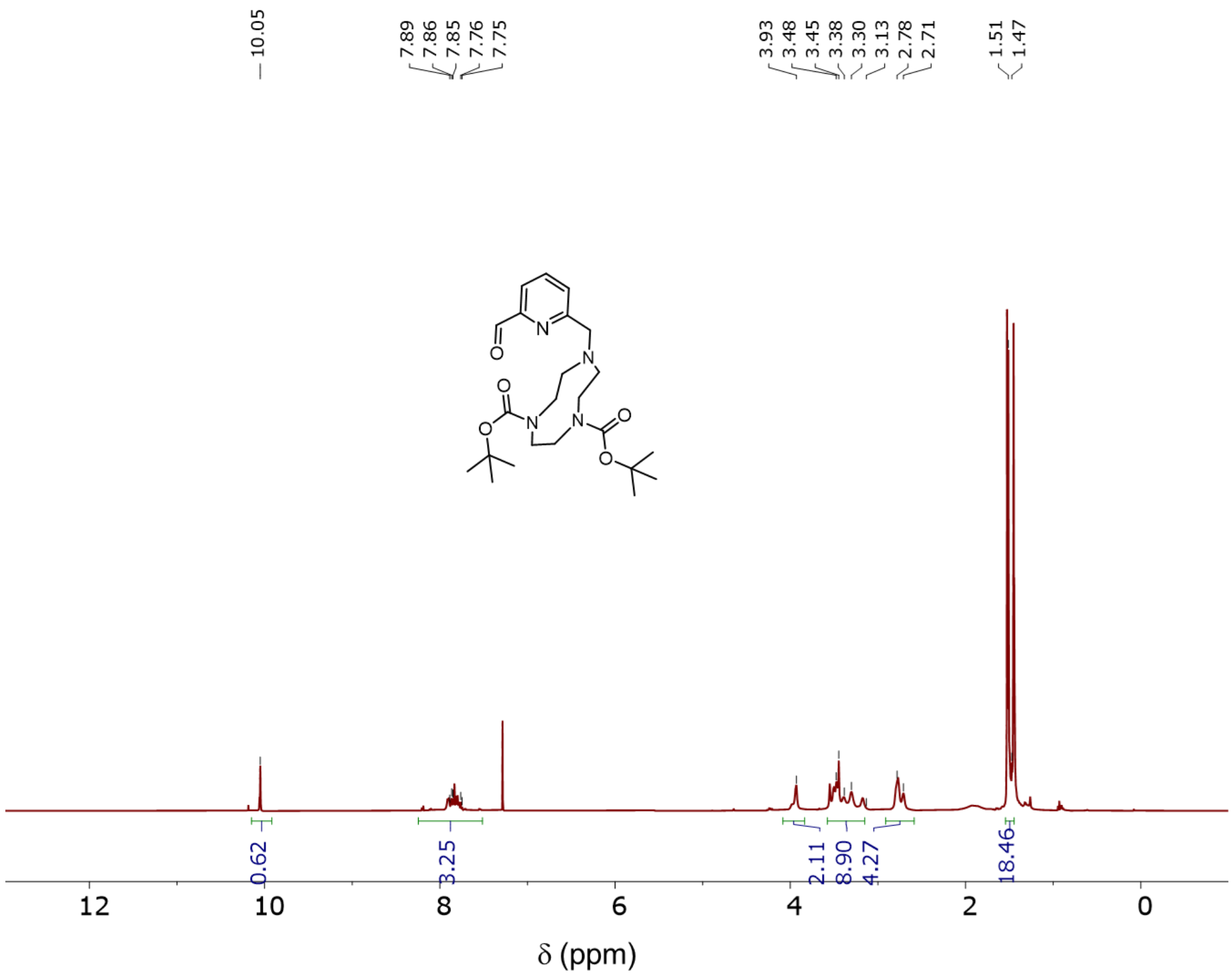

Figure S75. ^{1}H NMR spectrum of compound **iii** in $CDCl_3$.

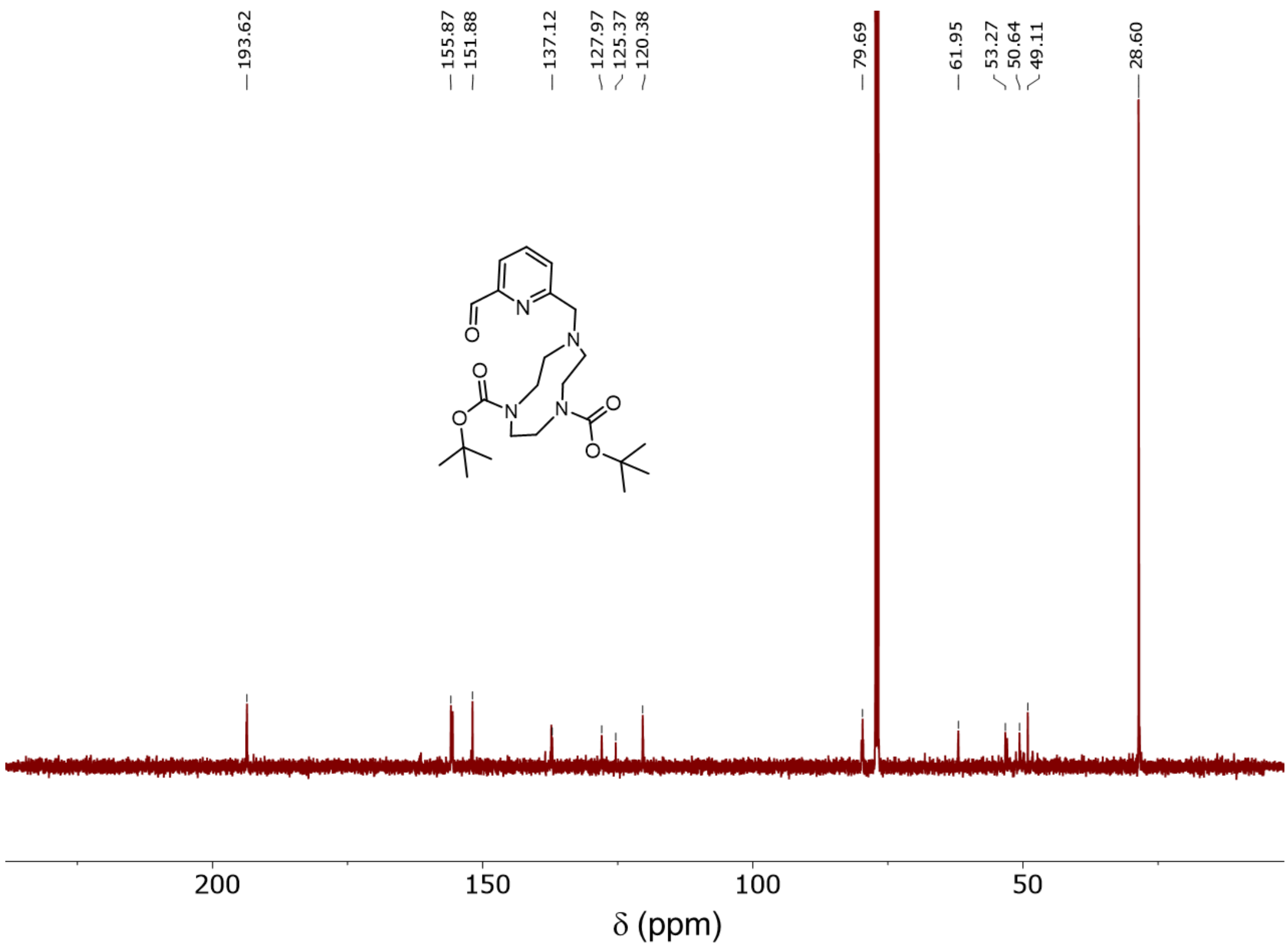

Figure S76. ^{13}C NMR spectrum of compound **iii** in $CDCl_3$.

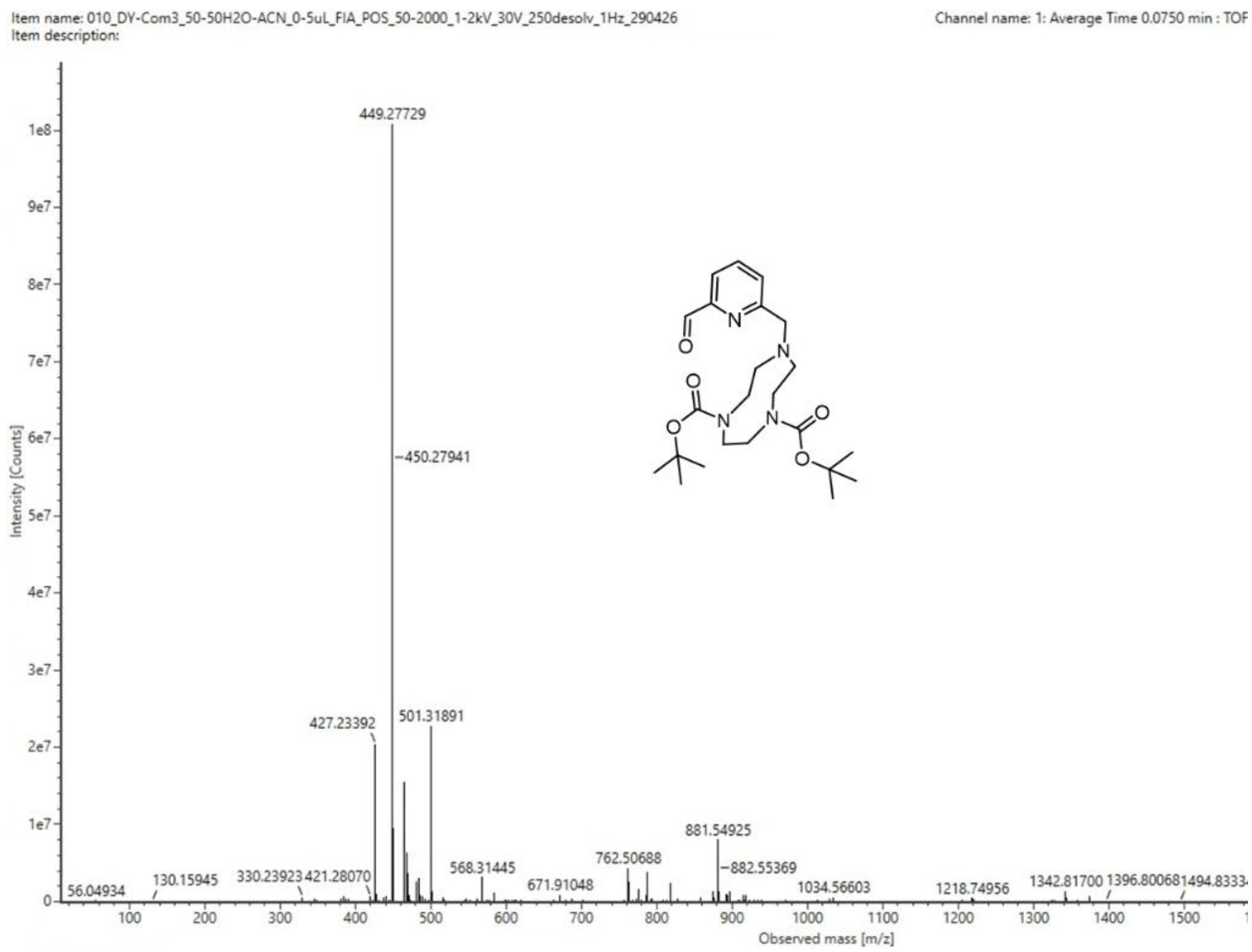

Figure S77. HRMS specta of compound **iii,** Calculated mass [M+H] = 449.276

Preparation of compound 6

Compound iii (100 mg) was dissolved in ethyl acetate (5 mL). A freshly prepared 3 M HCl solution was added, and the reaction mixture was stirred at room temperature overnight. The solvent was removed under reduced pressure to afford the pure product in quantitative yield.

^{1}H NMR (500 MHz, D_2O): δ 9.93 (s, 1H), 8.11 – 8.03 (m, 1H), 7.98 (d, *J* = 7.8 Hz, 1H), 7.62 (d, *J* = 7.8 Hz, 1H), 7.56 (d, *J* = 7.8 Hz, 1H), 7.52 (d, *J* = 7.8 Hz, 1H), 4.19 (s, 1H), 4.15 (s, 1H), 3.68 (s, 2H), 3.31 – 3.21 (m, 5H), 3.13 – 3.00 (m, 5H).

^{13}C NMR (125 MHz, D_2O): 194.05, 160.6, 157.06, 156.3, 150.2, 142.2, 139.9, 129.1, 128.1, 124.7, 121.4, 88.3, 57.1, 56.6, 48.7, 48.4, 44.6, 44.2, 43.2, 42.6

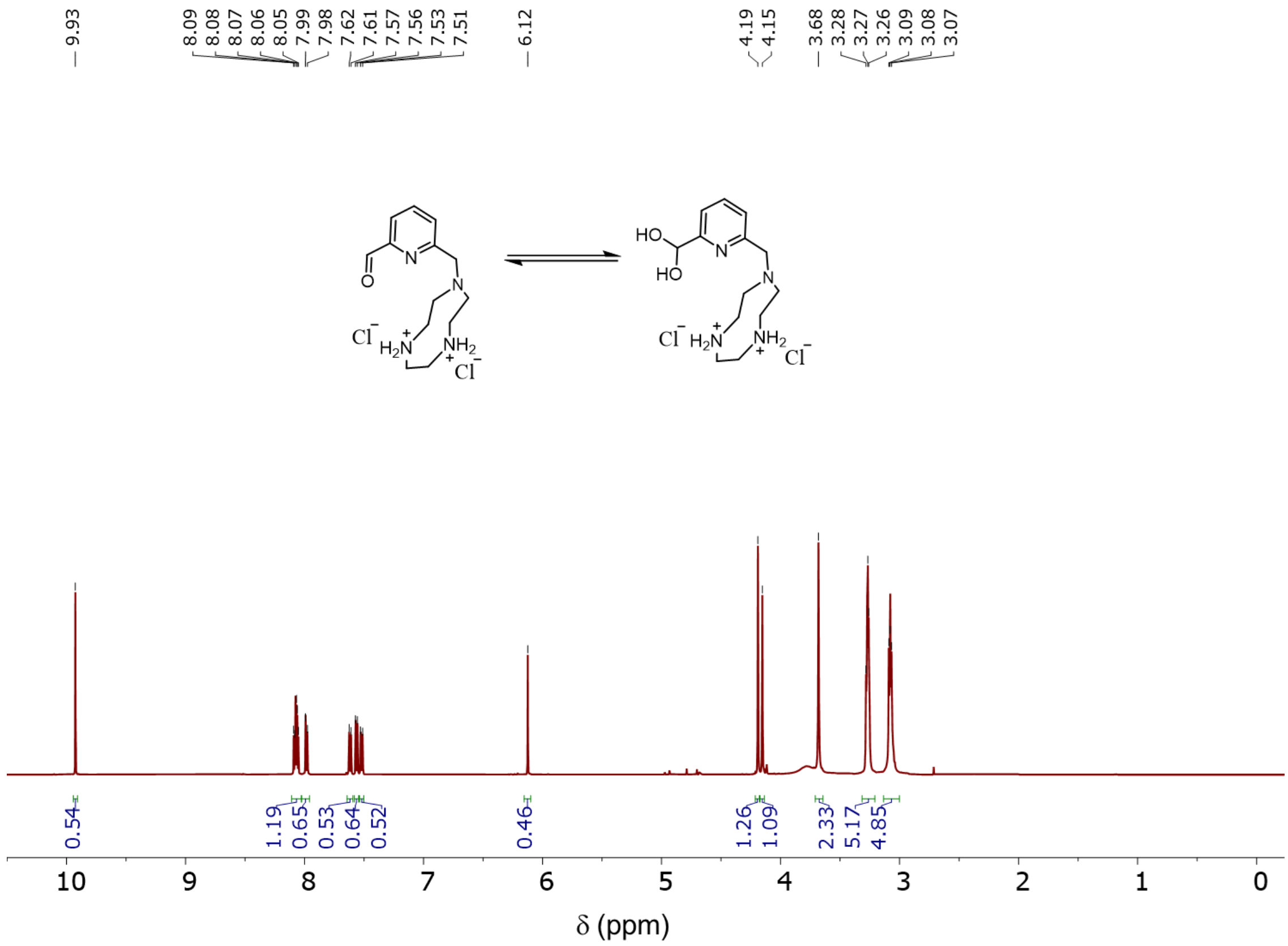

Figure S78. ^{1}H NMR spectrum of compound **6** in D_2O.

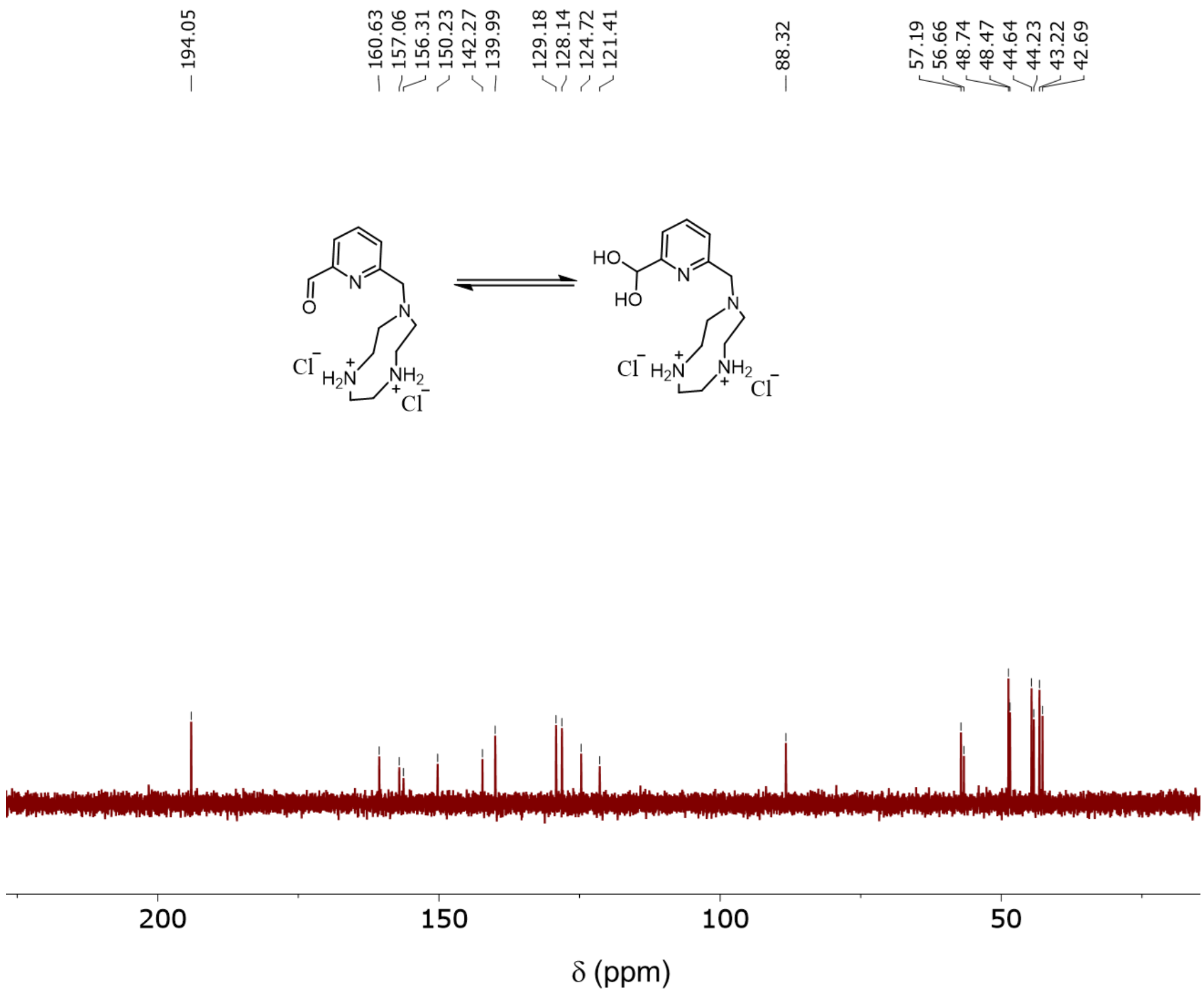

Figure S79. ^{13}C NMR spectrum of compound **6** in D_2O.

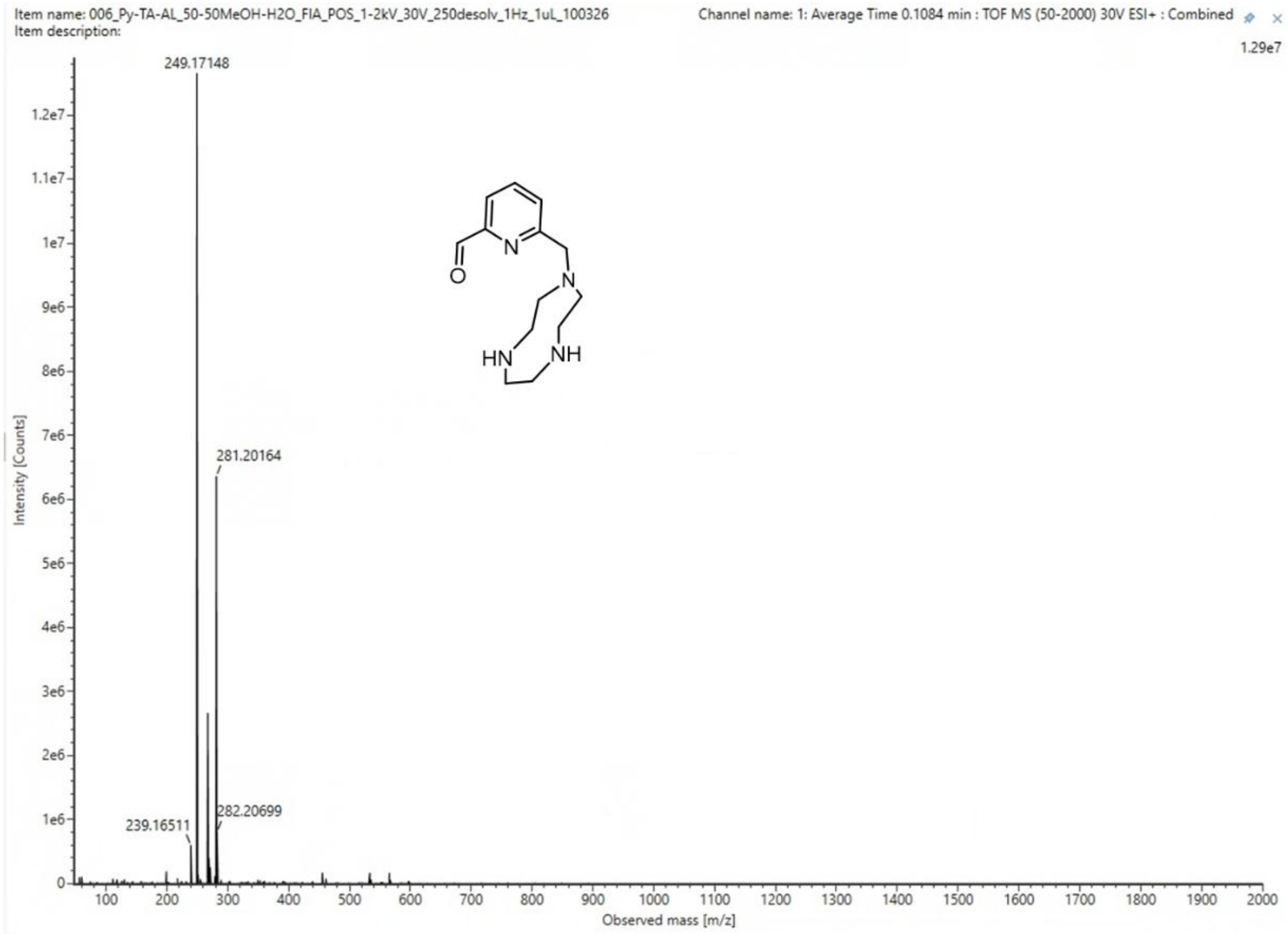

Figure S80. HRMS specta of compound **6,** Calculated mass [M+H] = 249.1720

S8. Formation of 1-A imine in PBS buffer

To evaluate the formation and stability of **1-A** in PBS buffer, aldehyde **1** (0.5 mM) and **TACN-aniline A** (0.5 mM) were mixed in 10 mM PBS (pD ~ 7.4) and monitored by NMR spectroscopy. The reaction initially proceeded efficiently, with the characteristic resonances of **1-A** clearly observed. After 24 h, turbidity appeared in the NMR tube, indicating the formation of aggregates. Importantly, the characteristic resonances corresponding to **1-A** remained detectable, demonstrating that the formed imine remains stable in the PBS environment.

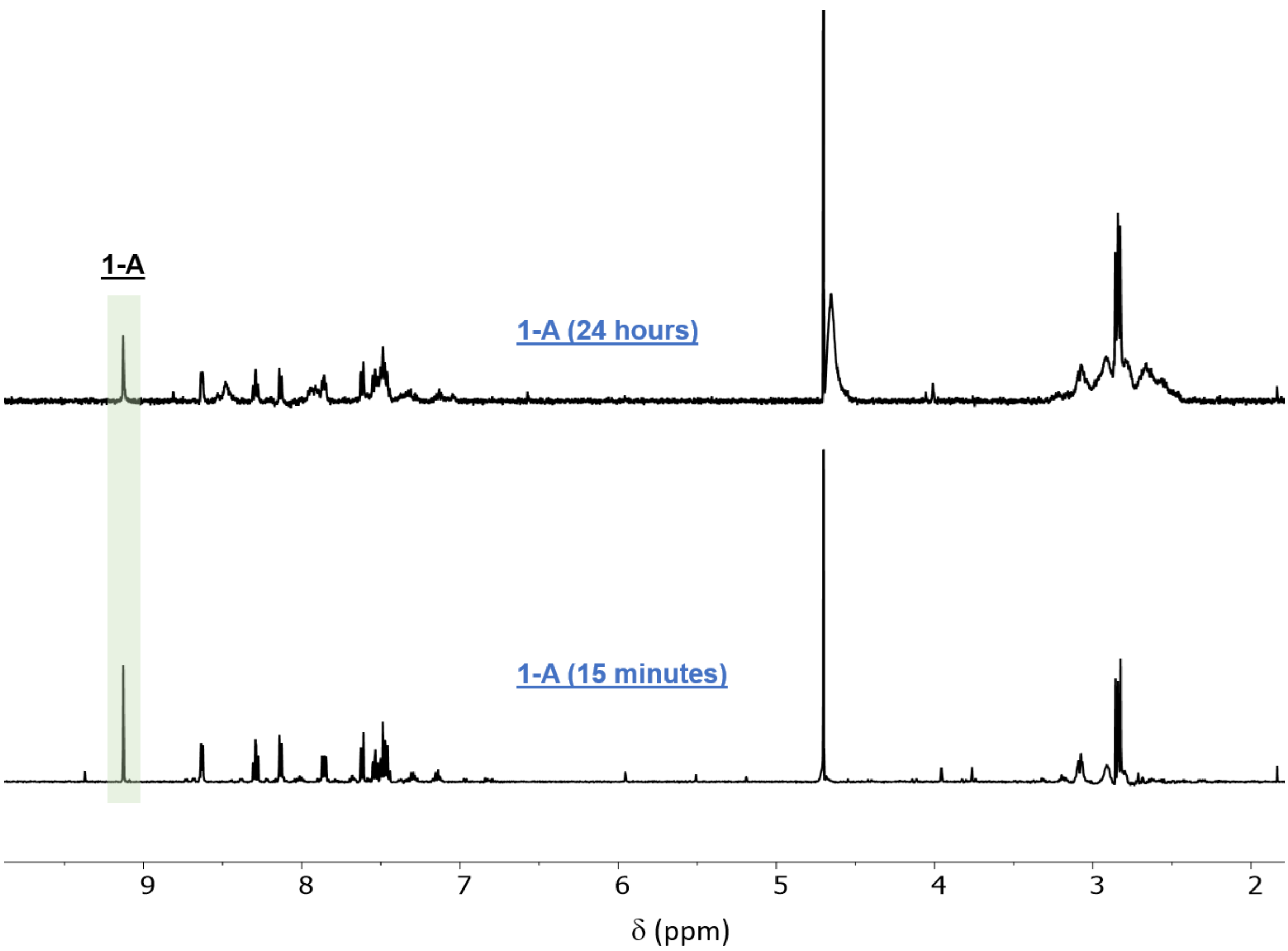

Figure S81. Parts of the ^{1}H spectra (500 MHz, D_2O) recorded between the indicated time intervals after mixing **1** and **A** (25 °C, pD = 7.4, [C] = 0.5 mM) in PBS buffer (10 mM).

S9. References

1. Gaussian 16, Revision B.01, M. J. Frisch, G. W. Trucks, H. B. Schlegel, G. E. Scuseria, M. A. Robb, J. R. Cheeseman, G. Scalmani, V. Barone, G. A. Petersson, H. Nakatsuji, X. Li, M. Caricato, A. V. Marenich, J. Bloino, B. G. Janesko, R. Gomperts, B. Mennu, and D. J. F. Gaussian Inc. Wallingford, CT, USA. at (2016).

2. Tomasi, J., Mennucci, B. & Cammi, R. Quantum Mechanical Continuum Solvation Models. *Chem. Rev.* **105**, 2999–3094 (2005).

3. Marchetti, T. *et al.* Near-Quantitative Formation of Imines in Water with Allosteric Control. *J. Am. Chem. Soc.* **148**, 4615–4621 (2026).

4. Griswold, A. R. *et al.* A Chemical Strategy for Protease Substrate Profiling. *Cell Chem. Biol.* **26**, 901–907 (2019).
